# N‐Heterocyclic Carbene/Carboxylic Acid Co‐Catalysis Enables Oxidative Esterification of Demanding Aldehydes/Enals, at Low Catalyst Loading

**DOI:** 10.1002/anie.202104712

**Published:** 2021-07-20

**Authors:** Wacharee Harnying, Panyapon Sudkaow, Animesh Biswas, Albrecht Berkessel

**Affiliations:** ^1^ Department of Chemistry (Organic Chemistry) University of Cologne Greinstraße 4 50939 Cologne Germany

**Keywords:** acylation, carbenes, cooperative catalysis, esterification, oxidation

## Abstract

We report the discovery that simple carboxylic acids, such as benzoic acid, boost the activity of N‐heterocyclic carbene (NHC) catalysts in the oxidative esterification of aldehydes. A simple and efficient protocol for the transformation of a wide range of sterically hindered α‐ and β‐substituted aliphatic aldehydes/enals, catalyzed by a novel and readily accessible N‐Mes‐/N‐2,4,6‐trichlorophenyl 1,2,4‐triazolium salt, and benzoic acid as co‐catalyst, was developed. A whole series of α/β‐substituted aliphatic aldehydes/enals hitherto not amenable to NHC‐catalyzed esterification could be reacted at typical catalyst loadings of 0.02–1.0 mol %. For benzaldehyde, even 0.005 mol % of NHC catalyst proved sufficient: the lowest value ever achieved in NHC catalysis. Preliminary studies point to carboxylic acid‐induced acceleration of acyl transfer from azolium enolate intermediates as the mechanistic basis of the observed effect.

## Graphical abstract

While N‐heterocyclic carbene (NHC) catalyzed oxidative esterification of aldehydes/enals is synthetically highly attractive, it is plagued by poor conversions/yields for sterically demanding substrates. The triazolium catalyst shown, with benzoic acid as co‐catalyst, overcomes this deficiency. A variety of hitherto recalcitrant aldehydes/enals are efficiently converted to esters with very low catalyst loadings (down to 50 ppm).

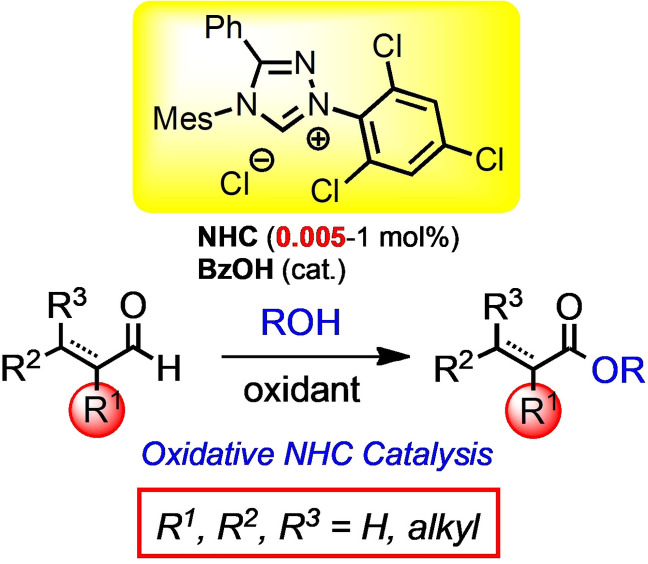

The ester functional group is encountered ubiquitously in organic molecules.^[1]^ As a consequence, the development of mild and efficient strategies for the synthesis of esters continues to be an important objective. Classical esterification methods involve the stoichiometric activation of carboxylic acids (as acid halides, anhydrides, or activated esters) amenable to subsequent coupling with alcohol nucleophiles.^[1]^ Over the past decade, the direct oxidative coupling of aldehydes with alcohols as a one‐pot procedure (oxidative esterification) has emerged as a conceptually and economically attractive alternative approach.^[2]^ In this context, various metal‐based and metal‐free catalytic systems employing mild oxidants have been developed. Among those, as an elegant organocatalytic process, the use of N‐heterocyclic carbenes (NHCs) has gained considerable interest as a mild acylation strategy. The latter transformation can be achieved either by incorporating a redox‐active functionality into the substrates, such as α‐reducible aldehydes or enals (NHC‐redox catalysis, Scheme 1 a),[^3^, ^4^] or by using external stoichiometric oxidants (oxidative NHC catalysis, Scheme 1 b).^[5]^ For the latter, various oxidants, including MnO_2_,^[6]^ organic oxidants (nitrobenzene, PhSSPh, TEMPO, diphenoquinone, flavins, azobenzene, phenazine, CCl_3_CN),^[7]^ electrochemistry,^[8]^ and O_2_/air in the presence/absence of transition metal catalysts,^[9]^ have been reported.

**Scheme 1:**
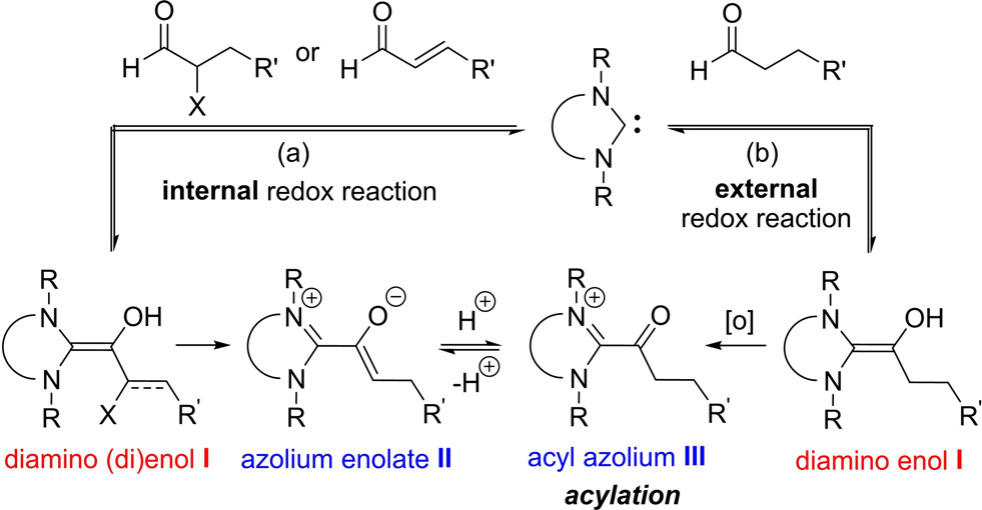
Acyl donor intermediates formed in NHC catalysis via a) internal redox reaction and b) external redox reaction.

Mechanistically, oxidative NHC catalysis relies on a two‐electron oxidation of the Breslow intermediate (diamino enol **I**)[^10^, ^11^] (Scheme 1) with an external oxidant to afford the acyl azolium intermediate **III**,^[12]^ which has been exploited in numerous useful new reactions.^[5]^ The most frequently used oxidant for this transformation is the Kharasch reagent **O1** (Scheme 2),^[13]^ pioneered by Studer et al. for NHC catalysis.^[7l]^ The oxidation process with **O1** works most efficiently for aromatic aldehydes/ enals as substrates.[^7l^, ^7m^, ^7n^, ^7o^, ^7p^, ^7q^, ^7r^, ^7s^, ^7t^] With aliphatic aldehydes, the transformation is comparatively sluggish, requires 7.5 mol % of the triazolium catalyst **A** (Scheme 2 a) at elevated temperature, and 2 equiv of Rb_2_CO_3_ as base.^[7p]^ Scheidt et al. reported the use of MnO_2_ (5 equiv) as oxidant in combination with the triazolium iodide **A** (10 mol %). In the presence of DBU (1.1 equiv), oxidative esterification of unbranched aliphatic aldehydes, and of some α‐ and β‐methyl substituted aldehydes was achieved (Scheme 2 a).[^6a^, ^6b^] It is a general observation that sterically demanding aliphatic aldehydes and α,β‐alkyl enals are challenging substrates for NHC catalysis. On the other hand, we had shown earlier[^4j^, ^14^] that NHC catalysts with low basicity, and carrying dispersion energy donors^[15]^ can overcome the above limitations. Key to success is superior catalyst stability, together with the ability to promote the formation of the Breslow intermediate. We reasoned that NHCs of the above type may just as efficiently catalyze aldehyde esterifications under oxidative conditions. Herein, we introduce the novel and readily accessible triazolium salt **C1** as a new and highly efficient catalyst for oxidative esterification (Scheme 2 b; see the Supporting Information for synthesis details). A whole series of sterically demanding aliphatic aldehydes and alkyl enals, hitherto not amenable to the known NHC‐catalyzed transformation using **O1** as oxidant, could smoothly be converted to esters (Scheme 2 b). We furthermore discovered that simple benzoic acid (BzOH) co‐catalyzes this transformation, allowing NHC catalyst loadings as low as 0.005 mol %.^[16]^

**Scheme 2:**
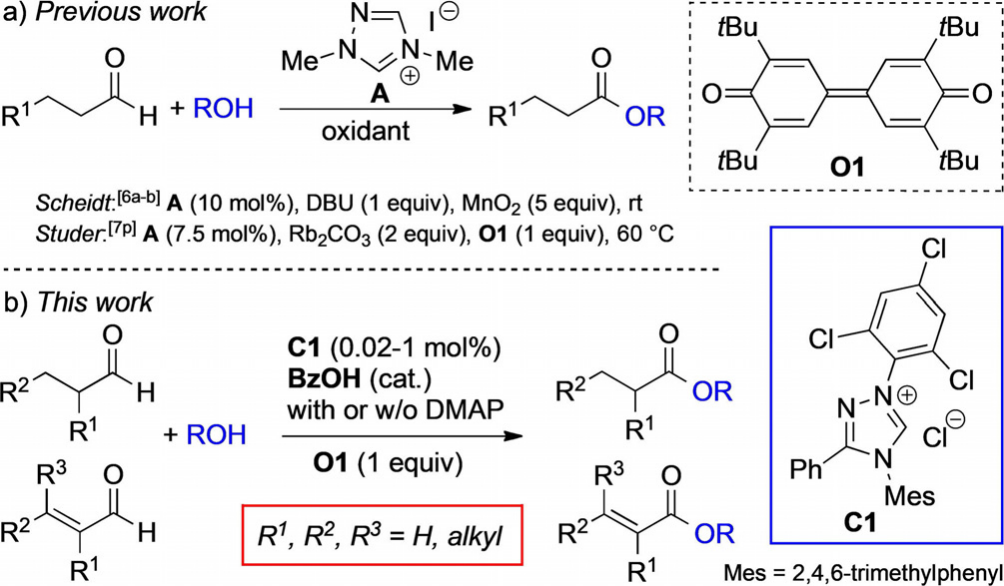
NHC‐catalyzed oxidative esterification of aliphatic aldehydes/enals.

As a touchstone substrate, we chose 2‐ethyl hexanal (**1 a**) which is unreactive in the presence of known NHC catalysts. **O1** was chosen as oxidant, as it can easily be recovered and recycled.^[14]^ Key results from extensive optimization (see the Supporting Information) are shown in Figure 1. Among the NHC catalysts examined (Supporting Information, Figure S3), the new triazolium salt **C1** was identified as the most effective one. Initially, treatment of **1 a** with benzyl alcohol (BnOH, **2 a**, 1.5 equiv) in the presence of **C1** (1 mol %), DIPEA (1 mol %), and **O1** (1.1 equiv) in tetrahydrofuran (THF) afforded only trace amounts of **3 aa** (8 % yield) after 24 h at rt. We discovered that BzOH (10 mol %) as co‐catalyst significantly enhances the catalytic efficiency of **C1** (1 mol %), even in the absence of added base, affording the desired ester **3 aa** in 96 % yield after a reaction time of only 5 h (Figure 1 A, line a).^[17]^ The reaction was further accelerated upon addition of a catalytic amine base (2 mol %), including DIPEA, DABCO, N‐methylmorpholine (NMM), N,N‐dimethylaminopyridine (DMAP), and pyridine (Figure 1 A). Among those, DMAP gave the best performance, completing the esterification within 3 h (Figure 1 A, line d), presumably promoting NHC turnover as proton shuttle and as an acyl‐transfer catalyst. No reaction occurred in the absence of the triazolium salt, regardless of whether or not base/BzOH was present. The loading of **C1** in the presence of BzOH could be decreased further to 0.5 mol % without compromising the product yield, albeit at somewhat longer reaction time (5 h) for full conversion. The efficiency of co‐catalysis was found to be strongly dependent on the type of acid. Different carboxylic acids were evaluated and simple BzOH provided best activity (Supporting Information, Figure S2).

**Figure 1:**
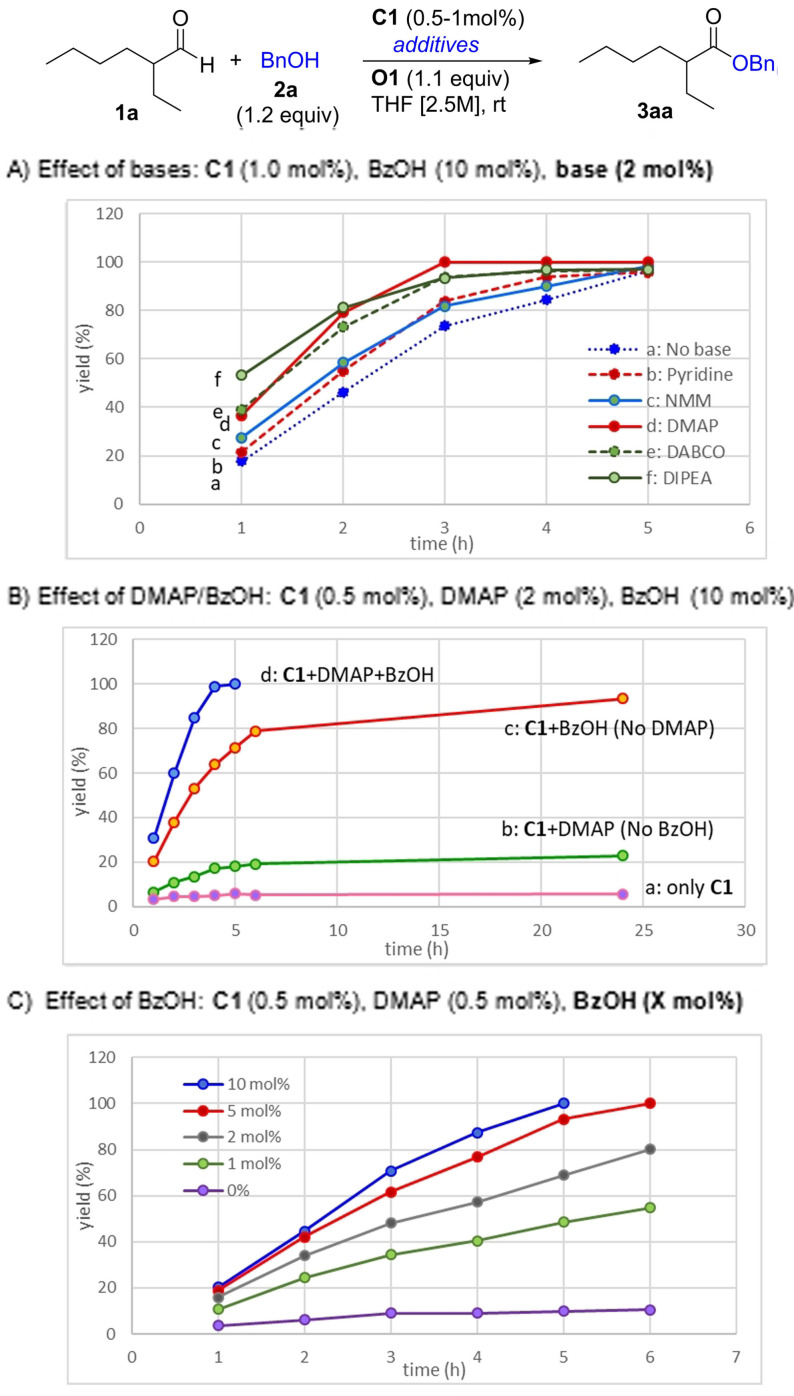
Kinetic studies on the effect of (a) base, (b) DMAP/BzOH, and (c) BzOH, as the co‐catalysts of **C1** in the reaction of **1 a** and **2 a**. Yields were determined by GC using dodecane as internal standard. A) Effect of bases: **C1** (1.0 mol %), BzOH (10 mol %), base (2 mol %). B) Effect of DMAP/BzOH: **C1** (0.5 mol %), DMAP (2 mol %), BzOH (10 mol %). C) Effect of BzOH: **C1** (0.5 mol %), DMAP (0.5 mol %), **BzOH (X mol %)**.

The effect of BzOH and DMAP on the kinetics of the reaction is compared in Figure 1 B. The initial rates of reactions with added BzOH (lines c,d) were significantly higher than those without (lines a,b). In the absence of BzOH as co‐catalyst, the reactions stalled at very low conversion (lines a,b). Varying the loadings of the BzOH and DMAP co‐catalysts revealed that higher BzOH concentrations accelerate the reactions further, with the accelerating effect approaching saturation at some point (around 10 mol %, Figure 1 C). At the same time, increasing the ratio of DMAP to **C1** above ca. 4:1 resulted in a less pronounced acceleration by BzOH (Supporting Information, Figure S4). These results indicate that external BzOH is crucial for promoting the NHC catalyst turnover, and/or preventing catalyst deactivation. Clearly, BzOH may also play a role in assisting the reduction of diquinone **O1** by H‐bonding/protonation.^[18]^ In summary, the use of **C1** (0.5 mol %), DMAP (1 mol %) and BzOH (10 mol %) proved most practical for the co‐catalyzed transformation of **1 a** and **2 a** to **3 aa**.

We next investigated the practicality and generality of this transformation (Figure 2). The oxidative esterification of the aldehydes **1** with the alcohol components **2** was performed on gram scale (5–15 mmol of substrate) under operationally simple conditions, that is, at room temperature and under air, unless otherwise stated. Simple Kugelrohr distillation was applied to isolate the ester from the crude product after evaporation of the THF solvent. Under these experimental conditions, **O1**‐H_2_/**O1** could be readily recovered, recycled to **O1** (97 % purity),^[14]^ and reused. To our delight, in the presence of catalyst **C1** (0.5 mol %), a variety of benzylic, heterobenzylic, allylic and primary alcohols with different chain lengths could be reacted with unbranched, α‐ and β‐branched aliphatic aldehydes **1 a**–**f**, affording the corresponding esters **3 aa**–**3 fa** in excellent yields (Figure 2). This method proved compatible with various substrate types and functional groups, such as furan, thiophene, acetyl‐, and Boc‐protected amines, pyridine, and indole. The reaction rates were rather sensitive to steric effects. For example, while the reaction of ethanol and less nucleophilic trifluoroethanol with **1 a** was completed within 5 h and 32 h, respectively (giving the esters **3 ag**–**3 ah**), trichloroethanol and *neo*‐pentanol required 9–10 days for complete conversion (products **3 ai**–**3 aj**). Similarly, the reaction of **1 a** with secondary alcohols required higher catalyst loadings (**C1**/DMAP/BzOH=1:2:20 mol %) to complete the esterification within reasonable time (products **3 ar**–**3 av**). The tertiary alcohol *t*‐BuOH failed to give the ester **3 aw**, only starting material was recovered.

**Figure 2:**
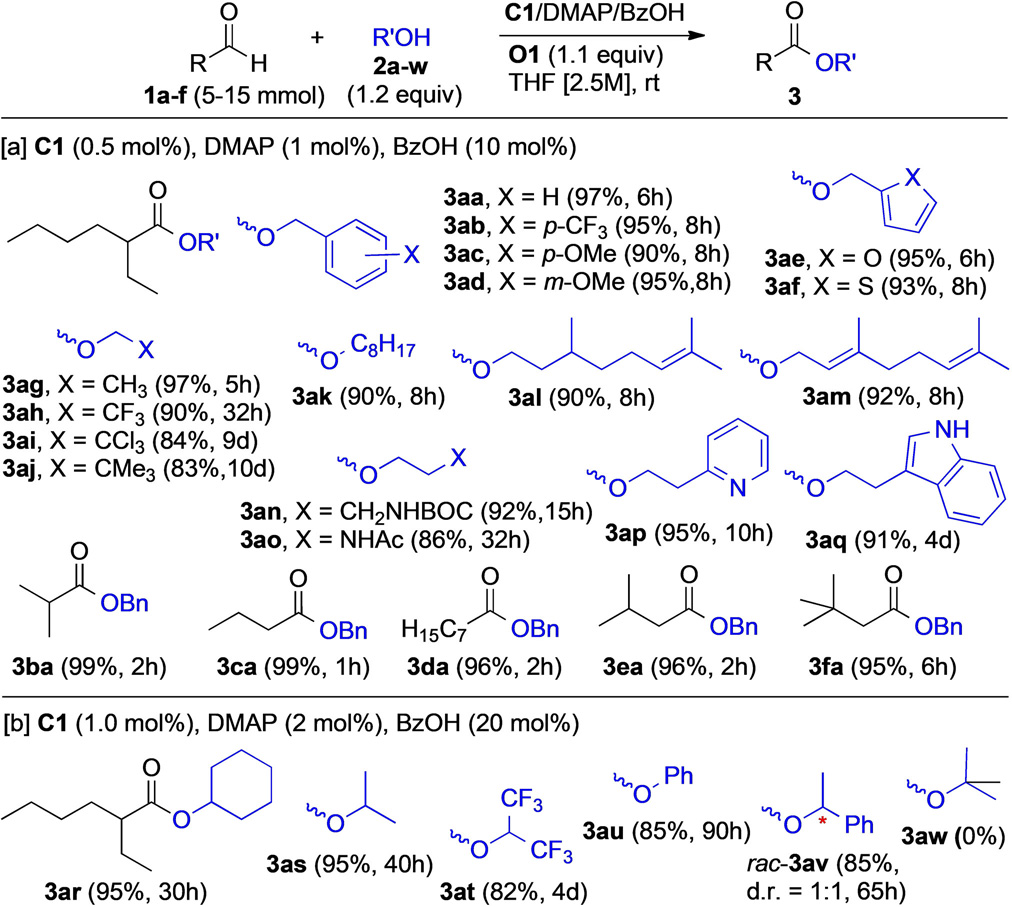
Substrate scope of the oxidative esterification of aliphatic aldehydes and alcohol nucleophiles. Yields refer to isolated products.

When methanol (**2 x**) was used as the nucleophile, the esterification proceeded very fast, and the addition of DMAP was not necessary (Figure 3). We found that the catalyst loading could be drastically reduced to only 0.05 mol % of **C1** and 2 mol % of BzOH for co‐catalyzing the esterification of **1 a** to give **3 ax** in 94 % isolated yield. The same conditions were applied to the synthesis of esters **3 dx** and **3 gx**–**3 lx**. For the aldehydes **1 d**, **1 g**, and **1 i**, the reaction with MeOH stalled after 2–3 h at room temperature, even at higher catalyst loadings. Nevertheless, full conversion could be accomplished by either performing the reaction at 50 °C, or at room temperature by adding an extra batch of **C1** (0.05 mol %) after 2–3 h. For small unbranched aldehydes, such as hexanal (**3 m**), 0.02 mol % loading of **C1** was sufficient to complete the esterification within 3 h (product **3 mx**). As an aldehyde substrate with an α‐stereogenic center, prone to racemization, enantiopure **1 n** (99 % *ee*) was esterified in the presence of 0.1 mol % of the catalyst **C1**. Complete conversion to the ester **3 nx** (64 % *ee*) occured within 4 h. The partial loss of stereochemical integrity, also observed under the conditions reported by Scheidt[^6a^, ^6b^] and Studer,^[7p]^ can be accounted for by the formation of the azolium enolate intermediate (**II**, Scheme 1) under oxidative conditions.^[12a]^ Sterically more demanding pivaldehyde (**1 o**) remained unchanged and did not react to yield the desired methyl pivalate (**3 ox**), even at catalyst loadings up to 15 mol %.

**Figure 3:**
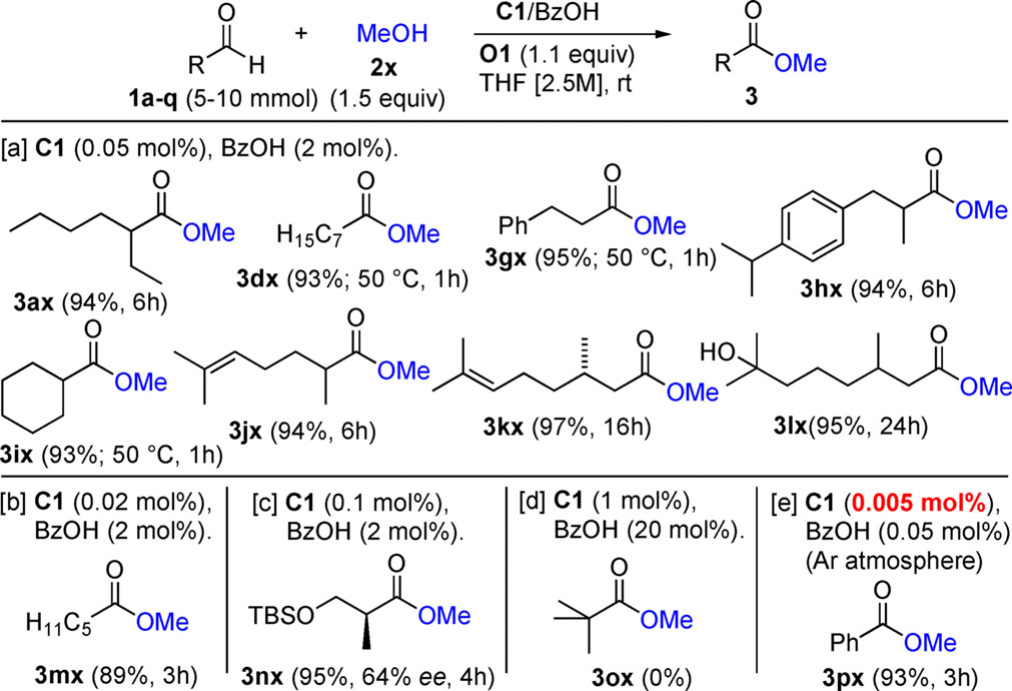
Scope of the oxidative esterification of aldehydes with methanol as alcohol component. Yields refer to isolated products.

We next addressed the question of what the minimal catalyst loading required for the oxidative esterification of more reactive aromatic aldehydes might be. To our delight, as little as 0.005 mol % (50 ppm) of **C1** and 0.05 mol % of BzOH were sufficient to transform benzaldehyde (**1 p**) to methyl benzoate (**3 px**) in 93 % yield, at room temperature, within 3 h (Figure 3 e). Note that for operating at such extremely low NHC concentration, inert atmosphere had to be applied. Although several successful examples of NHC‐acid co‐catalysis have been reported,^[19]^ the 50 ppm of **C1** disclosed here represent the lowest‐ever NHC loading in the presence of an external Brønsted acid co‐catalyst.

We finally evaluated the oxidative esterification of α,β‐enals **4**, with MeOH (**2 x**) as the alcohol component, using the substrate library shown in Figure 4. We were delighted to find that a diverse array of enals, including α‐ and β‐ substituted aromatic and aliphatic ones, reacted smoothly to give the corresponding esters **5** (Figure 4). Gratifyingly, even α‐substitued acroleins were successfully oxidized to the esters **5 fx**–**5 hx** in yields ranging from 80 % to 98 %, indicating the absence of NHC deactivation by Michael addition.^[4j]^ In the case of citral (**5 d**; *E*/*Z=*50:50), we observed *E*/*Z*‐isomerization, as the dienoate **5 dx** was obtained with an *E*/*Z* ratio of 68:32. Additionally, partial isomerization of the α,β‐unsaturation to the β,γ‐position was observed with **5 j** and **5 k,** giving the products **5 jx** and **5 kx**, respectively. Sterically congested β‐cyclocitral (**5 l**) was unreactive when the same experimental conditions were applied, and no product **5 lx** was observed.

**Figure 4:**
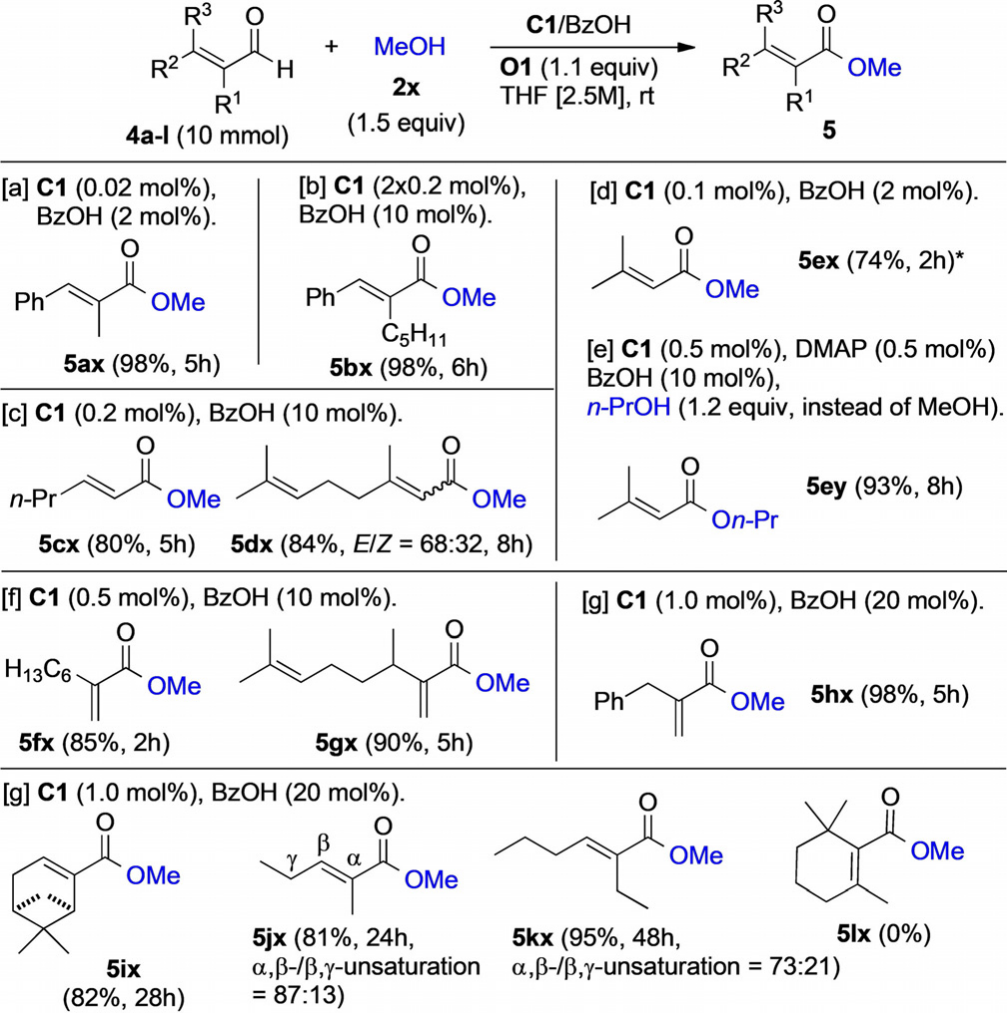
Scope of the oxidative esterification of enals. Yields refer to isolated products. *The lower yield of the isolated methyl ester **5 ex** (74 %) is due to its volatility, as the prenal substrate **5 e** was completely consumed with clean conversion. When changing to *n*‐propanol, the resulting ester **5 ey** could be isolated in 93 % yield.

Initial kinetic experiments were performed to shed light on the mechanism of the BzOH co‐catalysis. We have recently suggested that ester formation from aliphatic aldehydes proceeds via the azolium enolate state (such as **7**, Scheme 3), while esterification of aromatic aldehydes must proceed through an acyl azolium intermediate (such as **6 a**,**b**, Scheme 3).^[12a]^ The SIPr‐derived acetyl azolium triflate **6 a**, the analogous benzoyl azolium chloride **6 b** ^[20]^ and the acetyl azolium enolate **7** were exposed to BnOH (**2 a**) or methanol (**2 x**) in the presence of BzOH (1 equiv), and the reaction progress was monitored by ^1^H NMR (see the Supporting Information, Figures S10–S16, Tables S1,S2). In the presence of BzOH, the azolium enolate **7** reacted with BnOH to benzyl acetate ca. 11 times faster than in the absence of BzOH. This result suggests that for aliphatic aldehyde substrates (such as **1 a**), BzOH co‐catalysis involves interaction with the azolium enolate intermediate.^[21]^ In contrast, neither the acetyl azolium triflate **6 a** nor the benzoyl azolium chloride **6 b** reacted with BnOH (**2 a**) or methanol (**2 x**), regardless of whether or not BzOH was present. This result is in line with our observation that BzOH effects at best a slight acceleration in the case of benzaldehyde (**1 p**), where ester formation can proceed only via an acyl azolium intermediate, and not an azolium enolate (Supporting Information, Figure S9). While rate acceleration may be negligible, the presence of the BzOH co‐catalyst is still necessary for achieving extremely low catalyst loadings in benzaldehyde esterification (Figure 3 e). We conclude that BzOH also prevents, or at least retards, deactivation of the NHC catalyst. An obvious mechanistic option for this may be reversible protonation to the azolium cation.

**Scheme 3:**
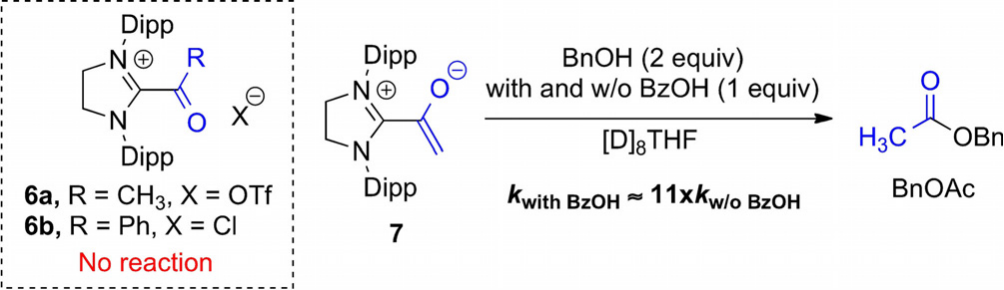
SIPr‐derived acetyl azolium triflate **6 a**, benzoyl azolium chloride **6 b**, acetyl azolium enolate **7**, and the effect of BzOH on the reaction of **7** with BnOH (**2 a**) or methanol (**2 x**) [Dipp: 2,6‐di(2‐propyl)phenyl].

In summary, we disclose an efficient and practical method for the NHC‐catalyzed oxidative coupling of alcohols with a wide range of sterically hindered α/β‐substituted aliphatic aldehydes and enals, hitherto recalcitrant to this type of transformation. Our method hinges on the novel and readily accessible triazolium salt **C1**, with low‐basicity and carrying dispersion energy donors. Furthermore, the introduction of benzoic acid as co‐catalyst was the key to success. The resulting cooperative catalytic system effects oxidative esterification at very low catalyst loadings (down to 50 ppm for benzaldehyde).

## Conflict of interest

The authors declare no conflict of interest.

## Supporting information

As a service to our authors and readers, this journal provides supporting information supplied by the authors. Such materials are peer reviewed and may be re‐organized for online delivery, but are not copy‐edited or typeset. Technical support issues arising from supporting information (other than missing files) should be addressed to the authors.

Supporting Information
