# Supplementary material for: N‐Heterocyclic Carbene/Carboxylic Acid Co‐Catalysis Enables Oxidative Esterification of Demanding Aldehydes/Enals, at Low Catalyst Loading
Source: Angew Chem Int Ed Engl. 2021 Jul 20;60(36):19631–6. doi: 10.1002/anie.202104712 (PMC8457137; doi:10.1002/anie.202104712)
Supplement: Supplementary file 1 — Supporting Information [file ANIE-60-19631-s001.pdf]

Angewandte Chemie
Eine Zeitschrift der Gesellschaft Deutscher Chemiker

Supporting Information

N-Heterocyclic Carbene/Carboxylic Acid Co-Catalysis Enables Oxidative Esterification of Demanding Aldehydes/Enals, at Low Catalyst Loading

Wacharee Harnying, Panyapon Sudkaow, Animesh Biswas, and Albrecht Berkessel**

anie_202104712_sm_miscellaneous_information.pdf

Table of Contents

1. General 2

2. Reaction optimization for the NHC-catalysed oxidative esterification of **1a** with **2a** 2

3. Synthesis of the triazolium salt **C1** 5

4. General procedure for the NHC-catalyzed oxidative esterification of aldehydes/enals 6

5. Gas chromatograms for the determination of the enantiomeric composition of 3-(*tert*-butyldimethylsilyloxy)-2-methylpropanal 13

6. Gas chromatograms for the determination of the enantiomeric composition of methyl 3-(*tert*-butyldimethylsilyloxy)-2-methylpropanoate (**3nx**) 14

7. Investigations on the effect of BzOH on acyl azolium and azolium enolate intermediates in the esterification 15

8. NMR spectra 21

9. References 72

1. General

Nuclear magnetic resonance (NMR) spectra were recorded on a Bruker Avance 300 instrument (^{1}H: 300.13 MHz, ^{13}C: 75.46 MHz, ^{19}F: 282.40 MHz), on a Bruker AV 400 instrument (^{1}H: 400.13 MHz, ^{13}C: 100.61 MHz, ^{19}F: 376 MHz), on a Bruker AV 500 instrument (^{1}H: 500.13 MHz, ^{13}C: 125.76 MHz), or a Bruker Avance II 600 instrument (^{1}H: 600.20 MHz, ^{13}C: 150.92 MHz) at ambient temperature. Chemical shifts (δ) are reported in parts per million (ppm) relative to tetramethylsilane (TMS) or solvent residual signals. The following abbreviations were used for chemical shift multiplicities in ^{1}H NMR spectra: br = broad, s = singlet, d = doublet, t = triplet, q = quartet, quint = quintet, sext = sextet, sept = septet, m = multiplet. IR spectra were recorded on a Shimadzu IR Affinity-1 FT-IR spectrometer. GC-MS analyses were done on a Hewlett Packard HP 6890 Series Plus gas chromatograph, injector and autosampler, HP 5973 Series mass-selective detector operated in electron-impact (EI) mode with an ionization energy of 75 eV and all other parameters set at autotune values, using H_2 as carrier gas and a HP-5 MS column (30 m x 0.25 mm ID, 0.25 μm film thickness): temperature program (Std 50): 50 °C, 5 min; 20 °C/min to 280 °C, 280 °C, 10 min, unless otherwise stated. ESI-mass spectra were measured on an Agilent 1100 Series LC/MSD instrument with a G1312A binary pump, G1313A autosampler and G1956A mass-selective detector. GC analyses were done on an Agilent 6890N Network gas chromatograph, injector and autosampler, and flame ionization detector, using N_2 as carrier gas. Elemental analyses were performed using an Elementar Vario MICRO cube elemental analyzer. Melting points were determined on a Büchi apparatus and are uncorrected.
Reagents were purchased from suppliers and used either as received or distilled prior to use. Anhydrous solvents were purchased from Acros. The oxidant **O1,**[1] the aldehyde (*S*)-**1n**,[2] and α-methylene substituted aldehydes[3] were synthesized according to literature procedures.

2. Reaction optimization for the NHC-catalysed oxidative esterification of 1a with 2a

General procedure: *For optimal standardization of the reaction conditions, the reactions were run in parallel in a glovebox.*
In a GC vial, the reaction was performed with 0.5 mmol of **1a** (78 μL), benzyl alcohol (BnOH, **2a**, 62/78 μL, 1.2/1.5 equiv), **O1** (225 mg, 0.55 mmol, 1.1 equiv), NHC salt (0.5–1.0 mol%) and dodecane (20 μL, 15 mg, as the internal standard) in dry THF (0.2–0.25 mL). The reaction mixture was stirred at ambient temperature. For the determination of aldehyde conversion and yield, an aliquot (5 μL) was withdrawn, filtered through a plug of silica gel and washed with EtOAc for GC analysis (Figures S1-S4).

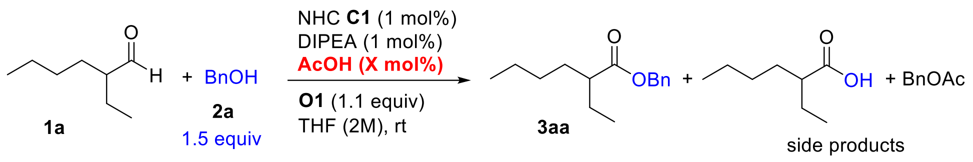

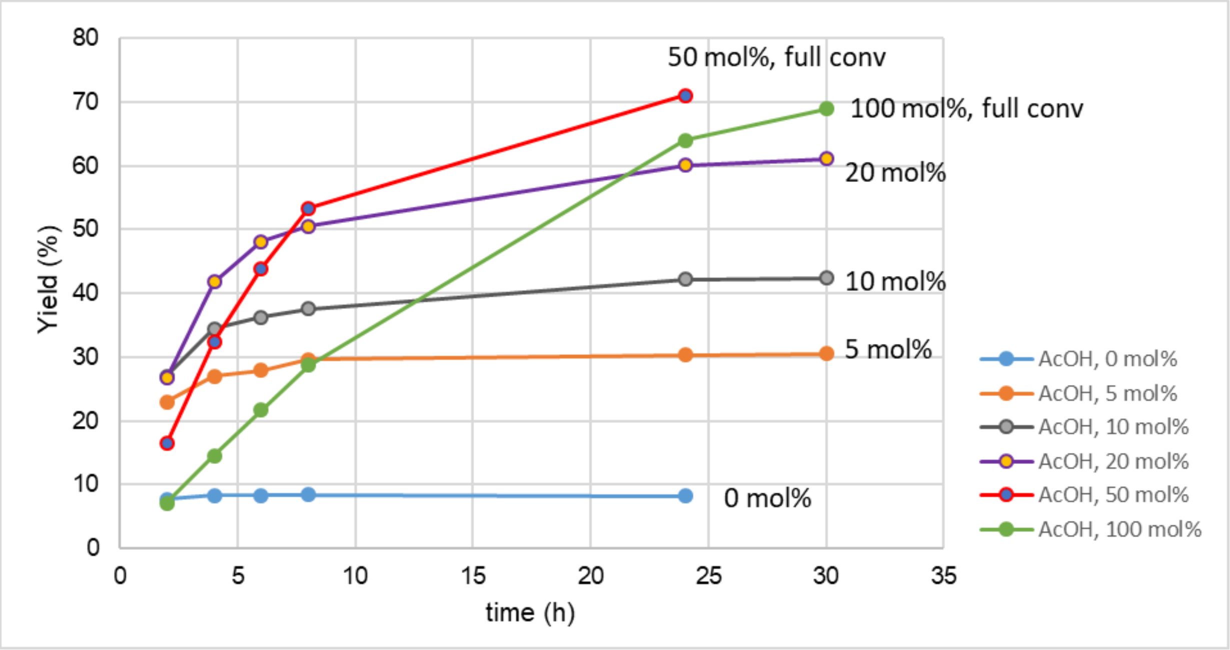

Figure S1. Acetic acid (AcOH) as the additive: effect of additive loadings.

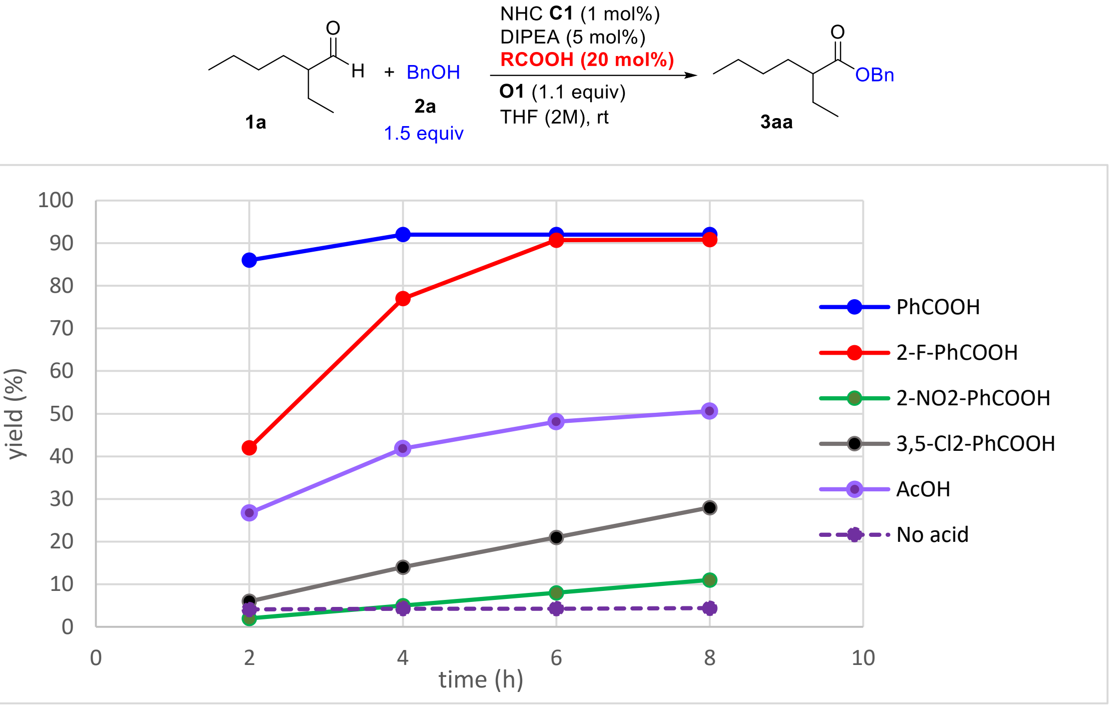

Figure S2. Effect of carboxylic acids (RCOOH) as additives.

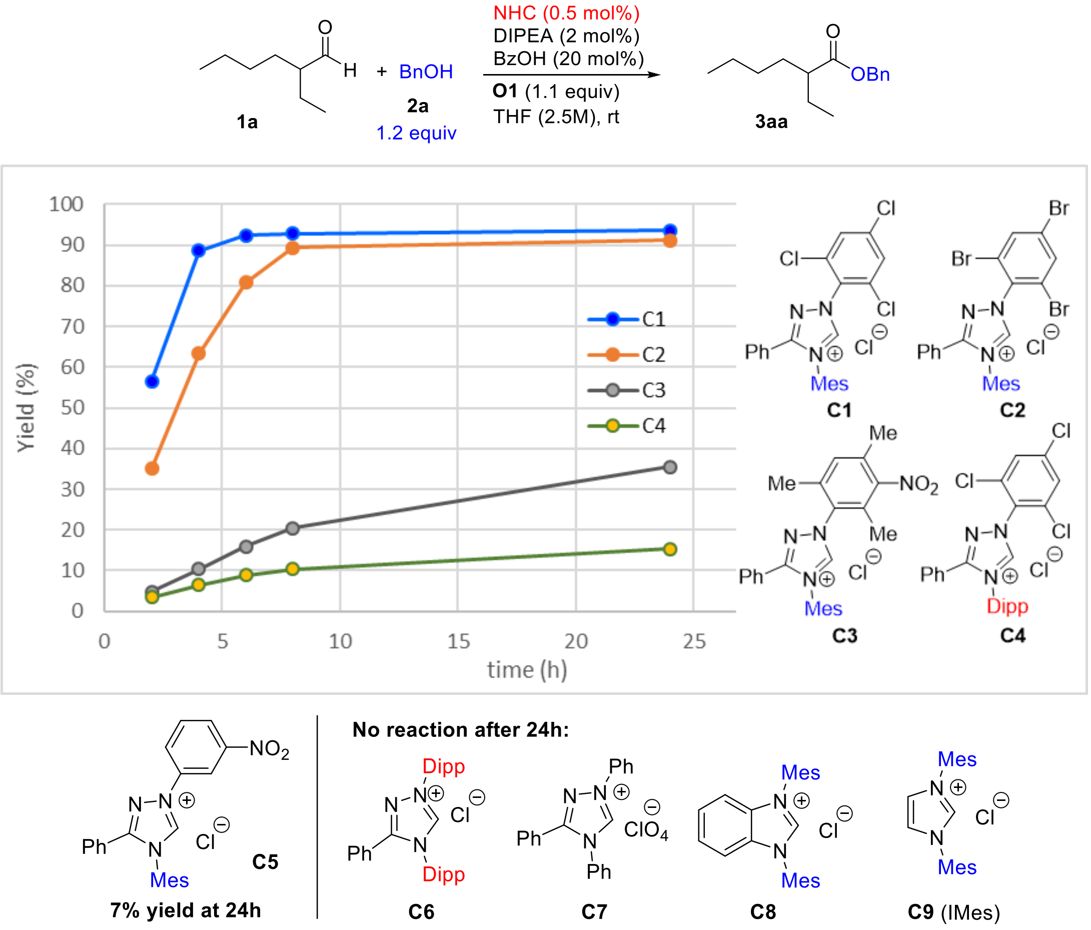

Figure S3. Effect of triazolium salts as catalysts.

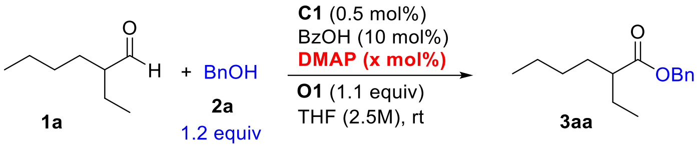

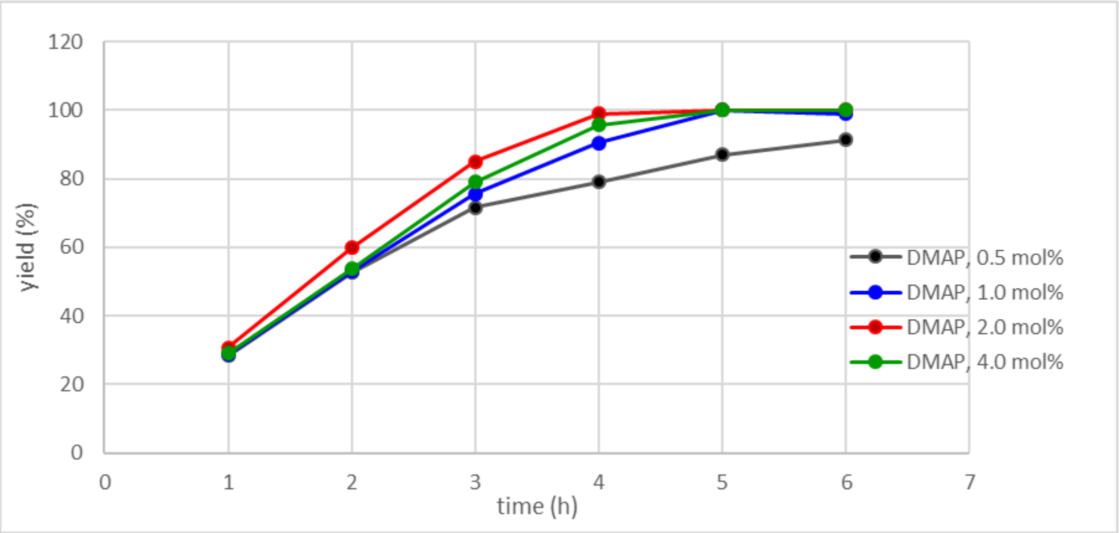

Figure S4. Effect of DMAP loadings.

3. Synthesis of the triazolium salt C1

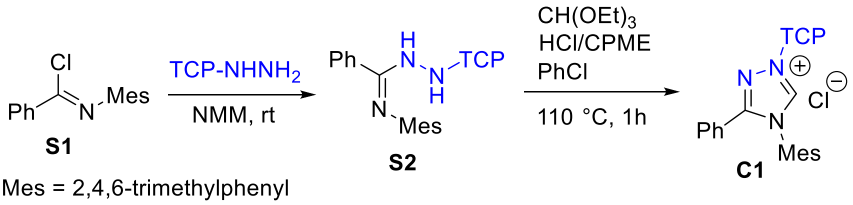

Mes = 2,4,6-trimethylphenyl
TCP = 2,4,6-trichlorophenyl

The benzimidoyl chloride **S1** (5.2 g, 20.0 mmol, 1.0 equiv), prepared according to the literature,[4] was treated with 2,4,6-trichlorophenyl hydrazine (4.2 g, 20.0 mmol, 1.0 equiv) in dry THF (50 mL) under Ar atmosphere. The mixture was cooled in an ice bath, followed by the addition of *N*-methylmorpholine (NMM, 2.4 mL, 22.0 mmol, 1.1 equiv). The reaction mixture was stirred at 0 °C for 30 min and then at rt for 24 h. The volatiles were removed on a rotary evaporator. The residue was diluted with water and extracted with DCM. The combined organic layers were washed with 1N HCl, water, brine, dried over Na_2SO_4, and filtered. After removal of the solvent, the crude product **S2** was obtained as an orange solid which was used in the next step without further purification.
To the crude **S2**, in a round-bottom flask, was successively added chlorobenzene (8.0 mL), triethyl orthoformate (10.0 mL, 60.0 mmol, 3.0 equiv), and anhydrous HCl in cyclopentyl methyl ether (CPME, 3M, 9.0 mL, 30.0 mmol, 1.5 equiv). The reaction flask was immersed in an oil bath at 110 °C, and the mixture was stirred open to air for 1 h. The reaction mixture was then concentrated on a rotary evaporator at 60°C. The residue was washed with Et_2O by vigorous stirring for 1 h and the Et_2O phase was removed. The residue was further triturated with EtOAc with vigorous stirring for at least 2 h. The resulting solid was collected by suction filtration and washed with EtOAc, furnishing the desired triazolium salt **C1** as a cream colored solid (2.0 g, 4.2 mmol, 21%).

1-(2,4,6-Trichlorophenyl)-4-(2,4,6-trimethylphenyl)-3-phenyl-1*H*-1,2,4-triazol-4-ium chloride (C1). Cream colored solid; mp 205–208 °C; ^{1}H NMR (300 MHz, DMSO-d_6): δ (ppm) = 11.99 (s, 1H, NCHN), 8.29 (s, 2H, ArH), 7.76–7.63 (m, 1H, ArH), 7.62–7.44 (m, 4H, ArH), 7.26 (s, 2H, ArH), 2.38 (s, 3H, CH_3), 2.10 (s, 6H, 2xCH_3); ^{13}C NMR (75 MHz, DMSO-d_6): δ (ppm) = 154.3 (C_q), 149.6 (NCHN), 142.1 (C_q), 138.7 (C_q), 134.4 (C_q), 133.41 (C_q), 133.36 (ArCH), 130.2 (2xArCH), 129.7 (4xArCH), 129.1 (C_q), 128.1 (2xArCH), 127.3 (C_q), 121.3 (C_q), 20.7 (CH_3), 17.2 (2xCH_3); IR (ATR): $\tilde{\nu}$ [cm^{-1}] = 1556 (s), 1460 (m), 855 (m), 828 (m), 779 (w), 702 (s), 622 (s), 608 (s), 577 (s); ESI-MS (positive): *m/z* = 442 (M^+-Cl, 100%); elemental analysis calcd (%) for $C_{23}H_{19}N_3Cl_4$•H_2O: C, 55.56; H, 4.26; N, 8.45; found: C, 55.31; H, 4.01; N, 8.37.

4. General procedure for the NHC-catalyzed oxidative esterification of aldehydes/enals

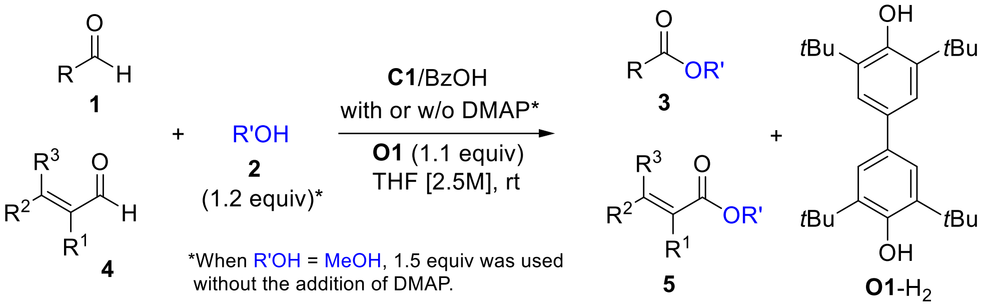

For reactions on 10 mmol scale: A 25 mL round-bottom flask was charged with **C1**, bezoic acid and **O1** (97% purity for the recycled **O1**, 4.5 g, 11 mmol, 1.1 equiv) and dry THF (4.0 mL). Alcohol **2** (12 mmol, 1.2 equiv) or methanol (0.6 mL, 15 mmol, 1.5 equiv) and aldehyde **1**/**4** (10 mmol, 1.0 equiv) were added successively. The flask was closed with a stopper and stirred at room temperature. After the reaction was complete (by GC), hexane was added and the solvent was evaporated under reduced pressure. This process was repeated 2-3 times for the removal of THF. The residue was subjected to *Kugelrohr*-distillation *via* a short *Vigreux* column filled with glass wool to give the desired ester **3**/**5**. If the product was contaminated with benzoic acid and/or alcohol **2**, it was diluted with hexanes, treated with Na_2CO_3 (solid) and filtered over a short pad of SiO_2 using hexanes and 5% Et_2O/hexanes as the eluent. For polar compounds, purification by short column chromatography on silica gel (EtOAc/cyclohexane as the eluent) was applied.

For recovering of **O1**-H_2/**O1**, the solid residue from the distillation was suspended in MeOH/H_2O (2:1), stirred for 30 min and collected by suction filtration, affording **O1**-H_2/**O1** (quant.) as a light brown solid. Of the recovered **O1**-H_2/**O1**, 30-50 g were collected and subjected to the re-oxidation to **O1**, according to the literature procedure.[4]

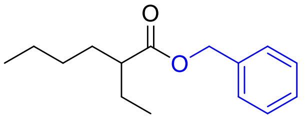
3aa

Benzyl 2-ethylhexanoate (3aa). Colorless liquid; ^{1}H NMR (300 MHz, $CDCl_3$): δ (ppm) = 7.45 – 7.26 (m, 5H, ArH), 5.13 (s, 2H, OCH_2), 2.32 (m, 1H, CH), 1.74 – 1.38 (m, 4H, 2xCH_2), 1.35–1.18 (m, 4H, 2xCH_2), 0.90–0.83 (m, 6H, 2xCH_3); ^{13}C NMR (75 MHz, $CDCl_3$): δ (ppm) = 176.3 (C=O), 136.3 (ArC), 128.5 (2xArCH), 128.1 (2xArCH), 128.1 (ArCH), 65.9 (OCH_2), 47.3 (CH), 31.8 (CH_2), 29.6 (CH_2), 25.5 (CH_2), 22.6 (CH_2), 13.9 (CH_3), 11.8 (CH_3); IR (ATR): $\tilde{\nu}$ [cm^{-1}] = 2959 (m), 2932 (m), 1732 (s), 1456 (m), 1381 (w), 1263 (m), 1213 (m), 1163 (s), 1139 (s), 1119 (m), 1092 (m), 975 (m), 748 (m), 733 (m); GC-MS: t_R (Std 50) = 12.4 min, *m/z* = 234 (M^+), 178, 143, 127, 108, 91 (100%), 77, 57.

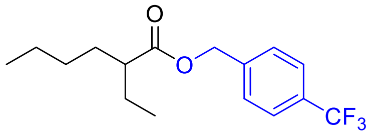

3ab

4-(Trifluoromethyl)benzyl 2-ethylhexanoate (3ab). Colorless liquid; ^{1}H NMR (300 MHz, $CDCl_3$): δ (ppm) = 7.62 (d, *J* = 8.2 Hz, 2H, ArH), 7.47 (d, *J* = 8.1 Hz, 2H, ArH), 5.18 (s, 2H, OCH_2), 2.35 (m, 1H, CH), 1.74–1.42 (m, 4H, 2xCH_2), 1.38–1.11 (m, 4H, 2xCH_2), 0.91–0.83 (m, 6H, 2xCH_3); ^{13}C NMR (75 MHz, $CDCl_3$): δ (ppm) = 176.0 (C=O), 140.3 (ArC), 128.1 (2xArCH), 125.5 (q, J = 3.6 Hz, 2xArCH), 124.4 (q, *J* = 271.7 Hz, CF_3), 64.87 (OCH_2), 47.3 (CH), 31.7 (CH_2), 29.6 (CH_2), 25.4 (CH_2), 22.6 (CH_2), 13.9 (CH_3), 11.8 (CH_3); ^{19}F NMR (376 MHz, $CDCl_3$) δ (ppm) = –62.6 (CF_3); IR (ATR): $\tilde{\nu}$ [cm^{-1}] = 2961 (m), 2935 (m), 1734 (s), 1460 (w), 1381 (w), 1323 (s), 1267 (m), 1213 (m), 1163 (s), 1124 (s), 1111 (s), 1018 (m), 1003 (m), 822 (m); GC-MS: t_R (Std 50) = 12.3 min, *m/z* = 302 (M^+), 283, 246, 217, 200, 173, 159 (100%), 140, 127, 109, 97, 83, 69, 57.

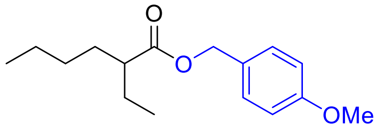

3ac

4-(Methoxy)benzyl 2-ethylhexanoate (3ac). Colorless liquid; ^{1}H NMR (500 MHz, $CDCl_3$): δ (ppm) = 7.32 (d, *J* = 8.7 Hz, 2H, ArH), 6.91 (d, *J* = 8.7 Hz, 2H, ArH), 5.08 (s, 2H, OCH_2), 3.83 (s, 3H, OCH_3), 2.31 (m, 1H, CH), 1.71–1.58 (m, 2H), 1.57–1.43 (m, 2H), 1.36–1.19 (m, 4H), 0.88 (t, *J* = 7.4 Hz, 3H, CH_3), 0.87 (t, *J* = 7.1 Hz, 3H, CH_3); ^{13}C NMR (126 MHz, $CDCl_3$): δ (ppm) = 176.5 (C=O), 159.6 (ArC), 130.1 (2xArCH), 128.6 (ArC), 114.0 (2xArCH), 65.9 (OCH_2), 55.4 (OCH_3), 47.4 (CH), 31.9 (CH_2), 29.7 (CH_2), 25.6 (CH_2), 22.8 (CH_2), 14.1 (CH_3), 12.0 (CH_3); IR (ATR): $\tilde{\nu}$ [cm^{-1}] = 2959 (m), 2933 (m), 1728 (s), 1612 (m), 1514 (s), 1458 (m), 1381 (w), 1358 (w), 1246 (s), 1163 (s), 1140 (s), 1033 (m), 970 (m), 819 (s), 808 (m); GC-MS: t_R (Std 50) = 13.8 min, *m/z* = 264 (M^+), 138, 121 (100%), 107, 91, 71, 67, 57.

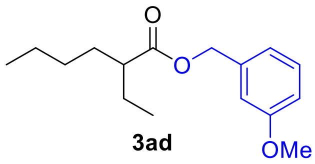

3ad

3-(Methoxy)benzyl 2-ethylhexanoate (3ad). Colorless liquid; ^{1}H NMR (300 MHz, $CDCl_3$): δ (ppm) = 7.26 (t, *J* = 7.8 Hz, 1H, ArH), 6.93 (d, *J* = 7.6 Hz, 1H, ArH), 6.91–6.80 (m, 2H, ArH), 5.10 (s, 2H, OCH_2), 3.80 (s, 3H, OCH_3), 2.33 (m, 1H, CH), 1.76–1.40 (m, 4H, 2xCH_2), 1.37–1.15 (m, 4H, 2xCH_2), 0.91–0.84 (m, 6H, 2xCH_3); ^{13}C NMR (75 MHz, $CDCl_3$): δ (ppm) = 176.2 (C=O), 159.7 (ArC), 137.9 (ArC), 129.5 (ArCH), 120.3 (ArCH), 113.6 (ArCH), 113.4 (ArCH), 65.7 (OCH_2), 55.2 (OCH_3), 47.3 (CH), 31.8 (CH_2), 29.6 (CH_2), 25.5 (CH_2), 22.6 (CH_2), 13.9 (CH_3), 11.9 (CH_3); IR (ATR): $\tilde{\nu}$ [cm^{-1}] = 2959 (m), 2933 (m), 1730 (s), 1603 (m), 1587 (m), 1490 (m), 1456 (m), 1437 (m), 1265 (s), 1163 (s), 1157 (s), 1139 (s), 1043 (m), 779 (m), 692 (m); GC-MS: t_R (Std 50) = 13.7 min, *m/z* = 264 (M^+), 209, 195, 163, 138 (100%), 121, 107, 91, 77, 57.

Furan-2-ylmethyl 2-ethylhexanoate (3ae). Pale yellow liquid; ^{1}H NMR (300 MHz, $CDCl_3$): δ (ppm) = 7.40 (dd, J = 1.8, 0.8 Hz, 1H, ArH), 6.39 (d, J = 3.1 Hz, 1H, ArH), 6.34 (dd, J = 3.2, 1.9 Hz, 1H, ArH), 5.08 (s, 2H, OCH_2), 2.29 (m, 1H, CH), 1.74–1.36 (m, 4H, $2xCH_2$), 1.35–1.13 (m, 4H, $2xCH_2$), 0.85 (t, J = 7.5 Hz, 6H, $2xCH_3$); ^{13}C NMR (75 MHz, $CDCl_3$): δ (ppm) = 176.0 (C=O), 149.9 (ArC), 143.0 (ArCH), 110.4 (ArCH), 110.3 (ArCH), 57.6 (OCH_2), 47.1 (CH), 31.7 (CH_2), 29.5 (CH_2), 25.4 (CH_2), 22.6 (CH_2), 13.9 (CH_3), 11.7 (CH_3); IR (ATR): $\tilde{\nu}$ [cm^{-1}] = 2961 (m), 2934 (m), 2874 (w), 2860 (w), 1732 (s), 1503 (w), 1458 (m), 1381 (w), 1227 (m), 1161 (s),1152 (s), 1136 (s), 1016 (m), 974 (m), 920 (m), 741 (s); GC-MS: t_R (Std 50) = 11.3 min, m/z = 224 (M^+), 127, 97, 81 (100%), 57.

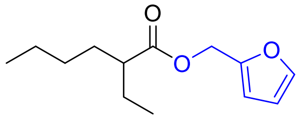

3ae

Thiophen-2-ylmethyl 2-ethylhexanoate (3af). Pale yellow liquid; ^{1}H NMR (300 MHz, $CDCl_3$): δ (ppm) = 7.29 (dd, J = 5.1, 1.2 Hz, 1H, ArH), 7.12–7.04 (m, 1H, ArH), 6.97 (dd, J = 5.1, 3.5 Hz, 1H, ArH), 5.28 (s, 2H, OCH_2), 2.29 (m, 1H, CH), 1.75–1.36 (m, 4H, $2xCH_2$), 1.35–1.13 (m, 4H, $2xCH_2$), 0.86 (t, J = 7.4 Hz, 3H, CH_3), 0.85 (t, J = 7.0 Hz, 3H, CH_3); ^{13}C NMR (75 MHz, $CDCl_3$): δ (ppm) = 176.0 (C=O), 138.4 (ArC), 127.9 (ArCH), 126.7 (ArCH), 126.6 (ArCH), 60.1 (OCH_2), 47.2 (CH), 31.7 (CH_2), 29.5 (CH_2), 25.4 (CH_2), 22.6 (CH_2), 13.9 (CH_3), 11.8 (CH_3); IR (ATR): $\tilde{\nu}$ [cm^{-1}] = 2956 (m), 2932 (m), 2872 (w), 2860 (w), 1732 (s), 1458 (m), 1381 (w), 1225 (m), 1161 (s), 1136 (s), 968 (m), 831 (m), 700 (s); GC-MS: t_R (Std 50) = 12.4 min, m/z = 240 (M^+), 127, 114, 97 (100), 57.

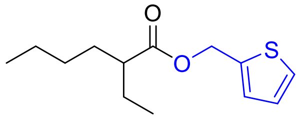

3af

Ethyl 2-ethylhexanoate (3ag). Colorless liquid; ^{1}H NMR (300 MHz, $CDCl_3$): δ (ppm) = 4.14 (q, J = 7.1 Hz, 2H, OCH_2), 2.24 (m, 1H, CH), 1.67–1.40 (m, 4H, $2xCH_2$), 1.37–1.16 (m, 4H, $2xCH_2$),1.26 (t, J = 7.1 Hz, 3H, CH_3), 0.97– 0.81 (m, 6H, $2xCH_3$); ^{13}C NMR (75 MHz, $CDCl_3$): δ (ppm) = 176.4 (C=O), 59.9 (OCH_2), 47.3 (CH), 31.8 (CH_2), 29.6 (CH_2), 25.5 (CH_2), 22.6 (CH_2), 14.3 (CH_3), 13.9 (CH_3), 11.8 (CH_3); IR (ATR): $\tilde{\nu}$ [cm^{-1}] = 2961 (m), 2934 (m), 2876 (w), 1732 (s), 1458 (m), 1375 (m), 1263 (m), 1175 (s), 1144 (m), 1094 (m), 1032 (m), 862 (w); GC-MS: t_R (Std 50) = 8.5 min, m/z = 144, 127, 116 (100%), 101, 88, 73, 57.

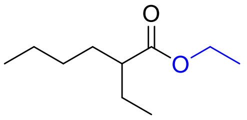

3ag

2,2,2-Trifluoroethyl 2-ethylhexanoate (3ah). Colorless liquid; ^{1}H NMR (300 MHz, $CDCl_3$): δ (ppm) = 4.48 (q, J = 8.5 Hz, 2H, OCH_2), 2.39 (m, 1H, CH), 1.79–1.40 (m, 4H, $2xCH_2$), 1.38–1.19 (m, 4H, $2xCH_2$), 1.00–0.79 (m, 6H, $2xCH_3$); ^{13}C NMR (75 MHz, $CDCl_3$): δ (ppm) = 174.7 (C=O), 123.4 (q, J = 276.9 Hz, CF_3), 50.8 (q, J = 36.5 Hz, OCH_2), 46.9 (CH), 31.5 (CH_2), 29.4 (CH_2), 25.3 (CH_2), 22.5 (CH_2), 13.8 (CH_3), 11.5 (CH_3); ^{19}F NMR (282 MHz, $CDCl_3$) δ (ppm) = –74.0 (CF_3); IR (ATR): $\tilde{\nu}$ [cm^{-1}] = 2965 (w), 2938 (w), 2864 (w), 1753 (m), 1458 (w), 1408 (w), 1281 (m), 1163 (s), 1132 (s), 1098 (m), 956 (m), 841 (w), 667 (w); GC-MS: t_R (Std 50) = 7.4 min, m/z = 198, 183, 170, 155 (100%), 135, 127, 83, 69, 55.

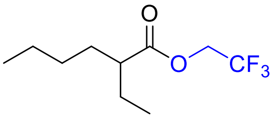

3ah

2,2,2-Trichloroethyl 2-ethylhexanoate (3ai). Pale yellow liquid; ^{1}H NMR (300 MHz, $CDCl_3$): δ (ppm) = 4.75 (s, 2H, OCH_2), 2.43 (m, 1H, CH), 1.81–1.46 (m, 4H, $2xCH_2$), 1.38–1.24 (m, 4H, $2xCH_2$), 0.97–0.86 (m, 6H, $2xCH_3$); ^{13}C NMR (75 MHz, $CDCl_3$): δ (ppm) = 174.6 (C=O), 95.1 (CCl_3), 73.80 (OCH_2), 47.1 (CH), 31.4 (CH_2), 29.5 (CH_2), 25.2 (CH_2), 22.6 (CH_2), 13.9 (CH_3), 11.8 (CH_3); IR (ATR): $\tilde{\nu}$ [cm^{-1}] = 2961 (m), 2934 (m), 2862 (w), 1749 (s), 1458 (m), 1385 (w), 1263 (w), 1198 (w), 1130 (s), 1059 (m), 939 (w), 758 (s), 716 (s); GC-MS: t_R (Std 80) = 9.8 min, m/z = 248, 218, 205, 167, 127, 115, 99, 88, 73, 57.

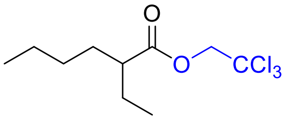

3ai

Neopentyl 2-ethylhexanoate (3aj). Pale yellow liquid; ^{1}H NMR (300 MHz, $CDCl_3$): δ (ppm) = 3.77 (s, 2H, OCH_2), 2.29 (m, 1H, CH), 1.74–1.39 (m, 4H, $2xCH_2$), 1.39–1.18 (m, 4H, $2xCH_2$), 0.95 [s, 9H, $C(CH_3)_3$], 1.93–1.86 (m, 6H, $2xCH_3$); ^{13}C NMR (75 MHz, $CDCl_3$): δ (ppm) = 176.4 (C=O), 73.4 (OCH_2), 47.5 (CH), 31.8 (CH_2), 31.2 (C_q), 29.6 (CH_2), 26.5 ($3xCH_3$), 25.5 (CH_2), 22.6 (CH_2), 13.9 (CH_3), 11.9 (CH_3);n IR (ATR): $\tilde{\nu}$ [cm^{-1}] = 2959 (m), 2934 (m), 2874 (w), 1732 (s), 1464 (w), 1379 (w), 1366 (w), 1263 (w), 1169 (m), 1144 (m), 997 (m); GC-MS: t_R (Std 50) = 10.0 min, m/z = 199, 186, 158, 144, 127, 116, 99, 88, 71, 57 (100%).

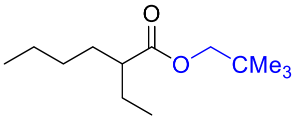

3aj

Octyl 2-ethylhexanoate (3ak). Colorless liquid; ^{1}H NMR (500 MHz, $CDCl_3$): δ (ppm) = 4.70 (t, J = 6.7 Hz, 2H, OCH_2), 2.25 (m, 1H, CH), 1.66–1.56 (m, 4H, $2xCH_2$), 1.54–1.39 (m, 2H, CH_2), 1.39–1.19 (m, 14H, $7xCH_2$), 0.92–0.83 (m, 9H, $3xCH_3$); ^{13}C NMR (126 MHz, $CDCl_3$): δ (ppm) = 176.7 (C=O), 64.3 (OCH_2), 47.6 (CH), 32.0 (CH_2), 31.9 (CH_2), 29.8 (CH_2), 29.4, 29.3 (CH_2), 28.9 (CH_2), 26.1 (CH_2), 25.7 (CH_2), 22.8 ($2xCH_2$), 14.2 (CH_3), 14.1 (CH_3), 12.0 (CH_3); IR (ATR): $\tilde{\nu}$ [cm^{-1}] = 2957 (m), 2926 (m), 2857 (m), 1732 (s), 1460 (m), 1263 (w), 1169 (s), 1144 (m), 1094 (s); GC-MS: t_R (Std 50) = 12.6 min, m/z = 256 (M^+), 200, 157, 145 (100%), 127, 112, 99, 88, 70, 57.

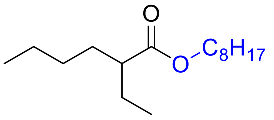

3ak

3,7-Dimethyloct-6-en-1-yl 2-ethylhexanoate (3al). Pale yellow liquid; ^{1}H NMR (500 MHz, $CDCl_3$): δ (ppm) = 5.09 (tt, J = 7.1, 1.3 Hz, 1H, CH=C), 4.16–4.06 (m, 2H, OCH_2), 2.24 (m, 1H, CH), 2.06–1.90 (m, 2H, CH_2), 1.68 (s, 3H, CH_3), 1.60 (s, 3H, CH_3), 1.71–1.14 (m, 13H, CH + $6xCH_2$), 0.91 (d, J = 6.6 Hz, 3H, CH_3), 0.88 (t, J = 7.4 Hz, 3H, CH_3), 0.88 (t, J = 7.1 Hz, 3H, CH_3); ^{13}C NMR (126 MHz, $CDCl_3$): δ (ppm) = 176.7 (C=O), 131.5 (C_q), 124.7 (CH), 62.6 (OCH_2), 47.6 (CH), 37.1 (CH_2), 35.8 (CH_2), 32.0 (CH_2), 29.8 (CH_2), 29.6 (CH), 25.9 (CH_3), 25.7 (CH_2), 25.6 (CH_2), 22.8 (CH_2), 19.5 (CH_3), 17.8 (CH_3), 14.1 (CH_3), 12.0 (CH_3); IR (ATR): $\tilde{\nu}$ [cm^{-1}]

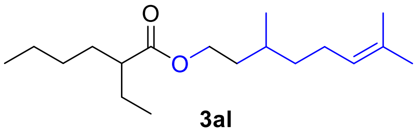

3al

= 2960 (m), 2928 (m), 2860 (w) 1732 (s), 1458 (m), 1379 (m), 1263 (m), 1169 (s), 1144 (m), 1094 (m), 984 (w); GC-MS: t_R (Std 50) = 13.3 min, *m/z* = 283 (M^+), 267, 254, 239, 227, 209, 183, 171, 138, 123, 109, 95, 81 (100%), 69, 57.

(*E*)-3,7-Dimethylocta-2,6-dien-1-yl 2-ethylhexanoate (3am). Pale yellow liquid; ^{1}H NMR (500 MHz, $CDCl_3$): δ (ppm) = 5.35 (td, *J* = 7.1, 1.2 Hz, 1H, CH=C), 5.35 (tt, *J* = 6.9, 1.3 Hz, 1H, CH=C), 4.60 (d, *J* = 7.1 Hz, 2H, OCH_2), 2.25 (m, 1H, CH), 2.13–2.01 (m, 4H, 2xCH_2), 1.70 (s, 3H, CH_3), 1.68 (s, 3H, CH_3), 1.60 (s, 3H, CH_3), 1.64–1.54 (m, 2H, CH_2), 1.53–1.39 (m, 2H, CH_2), 1.36–1.20 (m, 4H, 2xCH_2), 0.88 (t, *J* = 7.4 Hz, 3H, CH_3), 0.87 (t, *J* = 7.1 Hz, 3H, CH_3); ^{13}C NMR (126 MHz, $CDCl_3$): δ (ppm) = 176.6 (C=O), 142.2 (C_q), 131.9 (C_q), 123.9 (CH), 118.7 (CH), 61.1 (OCH_2), 47.5 (CH), 39.7 (CH_2), 32.0 (CH_2), 29.8 (CH_2), 26.4 (CH_2), 25.8 (CH_3), 25.7 (CH_2), 22.8 (CH_2), 17.8 (CH_3), 16.6 (CH_3), 14.1 (CH_3), 12.0 (CH_3); IR (ATR): $\tilde{\nu}$ [cm^{-1}] = 2961 (m), 2930 (m), 2860 (w), 1730 (s), 1456 (m), 1379 (m), 1263 (w), 1165 (s), 1142 (s), 1117 (m), 970 (m); GC-MS: t_R (Std 50) = 13.5 min, *m/z* = 280 (M^+), 265, 252, 237, 224, 211, 181, 169, 154, 136, 121, 107, 93, 80, 69 (100%), 57.

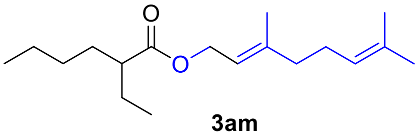

3am

Boc-aminopropyl 2-ethylhexanoate (3an). Pale yellow liquid; ^{1}H NMR (300 MHz, $CDCl_3$): δ (ppm) = 4.81 (br s, 1H, NH), 4.15 (t, *J* = 6.2 Hz, 2H, OCH_2), 3.20 (q, *J* = 6.3 Hz, 2H, NCH_2), 2.37–2.17 (m, 1H, CH), 1.93–1.74 (m, 2H, CH_2), 1.70–1.38 (m, 4H, 2xCH_2), 1.44 [s, 9H, $C(CH_3)_3$], 1.37–1.17 (m, 4H, 2xCH_2), 0.91–0.86 (m, 6H, 2xCH_3); ^{13}C NMR (75 MHz, $CDCl_3$): δ (ppm) = 176.5 (C=O), 155.9 (C=O), 61.6 (OCH_2), 47.3 (CH), 37.5 (CH_2), 31.7 (CH_2), 29.6 (CH_2), 29.2 (CH_2), 28.4 (3xCH_3), 25.4 (CH_2), 22.6 (CH_2), 13.9 (CH_3), 11.8 (CH_3); IR (ATR): $\tilde{\nu}$ [cm^{-1}] = 3352 (br w), 2961 (m), 2934 (m), 2862 (w), 1715 (s), 1697 (s), 1516 (m), 1456 (m), 1366 (m), 1248 (m), 1165 (s), 1040 (w), 974 (w), 779 (w); GC-MS: t_R (Std 50) = 13.9 min, *m/z* = 245, 228, 145, 127, 118, 102, 74, 57 (100%).

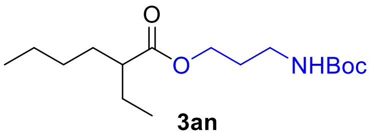

3an

2-Acetamidoethyl 2-ethylhexanoate (3ao). Colorless liquid; ^{1}H NMR (300 MHz, $CDCl_3$): δ (ppm) = 6.20 (s, 1H, NH), 4.19 (t, *J* = 5.5 Hz, 2H, OCH_2), 3.51 (q, *J* = 5.6 Hz, 2H, NCH_2), 2.29 (m, 1H, CH), 1.99 (s, 3H, $COCH_3$), 1.70–1.40 (m, 4H, 2xCH_2), 1.38–0.78 (m, 4H, 2xCH_2), 0.98–0.78 (m, 6H, 2xCH_3); ^{13}C NMR (75 MHz, $CDCl_3$): δ (ppm) = 176.6 (C=O), 170.4 (C=O), 62.6 (OCH_2), 47.2 (CH), 39.0 (CH_2), 31.6 (CH_2), 29. 6 (CH_2), 25.4 (CH_2), 23.1 (CH_3), 22.5 (CH_2), 13.9 (CH_3), 11.8 (CH_3); IR (ATR): $\tilde{\nu}$ [cm^{-1}] = 3285 (br w), 3082 (br w), 2959 (m), 2934 (m), 2862 (w), 1732 (s), 1655 (s), 1550 (m), 1458 (m), 1373 (m), 1263 (m), 1169 (s), 1144 (s), 1040 (m), 729 (w); GC-MS: t_R (Std 50) = 12.7 min, *m/z* = 186, 173, 127, 99, 85, 72, 57 (100%).

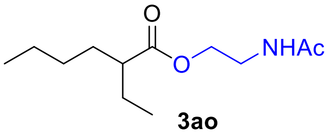

3ao

Pyridin-2-ylethyl 2-ethylhexanoate (3ap). Pale yellow liquid; ^{1}H NMR (300 MHz, $CDCl_3$): δ (ppm) = 8.56 (m, 1H, ArH), 7.61 (td, *J* = 7.7, 1.8 Hz, 1H, ArH), 7.23–7.11 (m, 2H, ArH), 4.49 (t, *J* = 6.7 Hz, 2H, OCH_2), 3.13 (t, *J* = 6.7 Hz, 2H, $NCCH_2$), 2.22 (m, 1H, CH), 1.65–1.32 (m, 4H, 2xCH_2), 1.30–1.06 (m, 4H, 2xCH_2), 0.84 (t, *J* = 7.1 Hz, 3H, CH_3), 0.81 (t, *J* = 7.5 Hz, 3H, CH_3); ^{13}C NMR (75 MHz, $CDCl_3$): δ (ppm) = 176.2 (C=O), 158.0 (PyC), 149.3 (PyCH), 136.3 (PyCH), 123.4 (PyCH), 121.6 (PyCH), 63.1 (OCH_2), 47.2 (CH), 37.3 (CH_2), 31.7 (CH_2), 29.5 (CH_2), 25.4 (CH_2), 22.6 (CH_2), 13.9 (CH_3), 11.7 (CH_3); IR (ATR): $\tilde{\nu}$ [cm^{-1}] = 2959 (m), 2932 (m), 2860 (w), 1730 (s), 1591 (m), 1458 (m), 1437 (m), 1263 (m), 1169 (s), 1144 (s), 1094 (m), 991 (m), 750 (m); GC-MS: t_R (Std 50) = 13.2 min, *m/z* = 234, 220, 206, 193, 178, 122 (100%), 106, 93, 78, 57.

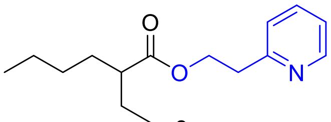

3ap

Indol-3-ylethyl 2-ethylhexanoate (3aq). Pale yellow liquid; ^{1}H NMR (300 MHz, $CDCl_3$): δ (ppm) = 8.16 (s, 1H, NH), 7.63 (d, *J* = 7.6 Hz, 1H, ArH), 7.29 (d, *J* = 7.7 Hz, 1H, ArH), 7.23–7.05 (m, 2H, ArH), 6.94 (d, *J* = 2.3 Hz, 1H, ArH), 4.37 (t, *J* = 7.3 Hz, 2H, OCH_2), 3.09 (t, *J* = 7.2 Hz, 2H, CH_2), 2.28 (m, 1H, CH), 1.70–1.37 (m, 4H, 2xCH_2), 1.34–1.12 (m, 4H, 2xCH_2), 0.90–0.81 (m, 6H, 2xCH_3); ^{13}C NMR (75 MHz, $CDCl_3$): δ (ppm) = 176.8 (C=O), 136.3 (ArC), 127.5 (ArC), 122.2 (ArCH), 122.0 (ArCH), 119.4 (ArCH), 118.8 (ArCH), 111.9 (ArC), 111.3 (ArCH), 64.3 (OCH_2), 47.5 (CH), 31.9 (CH_2), 29.7 (CH_2), 25.6 (CH_2), 25.0 (CH_2), 22.7 (CH_2), 14.0 (CH_3), 11.9 (CH_3); IR (ATR): $\tilde{\nu}$ [cm^{-1}] = 3402 (br w), 2959 (w), 2932 (w), 2859 (w), 1711 (m), 1456 (m), 1337 (w), 1263 (w), 1175 (m), 1094 (m), 989 (w), 804 (w), 737 (s); GC-MS: t_R (Std 50) = 16.1 min, *m/z* = 287 (M^+), 143 (100%), 130, 115, 103, 89, 77, 57.

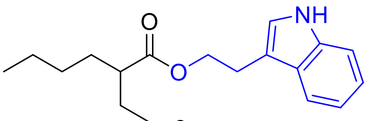

3aq

Benzyl 2-methylpropanoate (3ba). Pale yellow liquid; ^{1}H NMR (300 MHz, $CDCl_3$): δ (ppm) = 7.36–7.30 (m, 5H, ArH), 5.11 (s, 2H, OCH_2), 2.60 (hept, *J* = 7.0 Hz, 1H, CH), 1.19 (d, *J* = 7.0 Hz, 6H, 2xCH_3); ^{13}C NMR (75 MHz, $CDCl_3$): δ (ppm) = 176.9 (C=O), 136.3 (ArC), 128.5 (2xArCH), 128.1 (ArCH), 128.0 (2xArCH), 66.0 (OCH_2), 34.0 (CH), 19.0 (2xCH_3); IR (ATR): $\tilde{\nu}$ [cm^{-1}] = 2974 (w), 2936 (w), 2878 (w), 1732 (s), 1497 (w), 1456 (m), 1389 (w), 1343 (w), 1258 (m), 1188 (m), 1146 (s), 1069 (m), 966 (m), 746 (m), 735 (m), 696 (s); GC-MS: t_R (Std 50) = 10.2 min, *m/z* = 178 (M^+), 108, 91 (100%), 77, 65, 51.

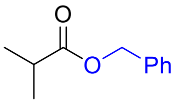

3ba

Benzyl butanoate (3ca). Pale yellow liquid; ^{1}H NMR (300 MHz, $CDCl_3$): δ (ppm) = 7.40–7.23 (m, 5H, ArH), 5.12 (s, 2H, OCH_2), 2.34 (t, *J* = 7.4 Hz, 2H, CH_2), 1.68 (q, *J* = 7.4 Hz, 2H, CH_2), 0.95 (t, *J* = 7.4 Hz, 3H, CH_3); ^{13}C NMR (75 MHz, $CDCl_3$): δ (ppm) = 173.5 (C=O), 136.2 (ArC), 128.5 (2xArCH), 128.2 (3xArCH), 66.0 (OCH_2), 36.2 (CH_2), 18.5 (CH_2), 13.7 (CH_3); IR (ATR): $\tilde{\nu}$ [cm^{-1}] = 3034 (w), 2965 (w), 2876 (w), 1732 (s), 1456 (m), 1383 (w), 1256 (m),

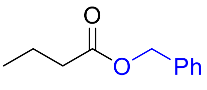

3ca

1167 (s), 1088 (m), 972 (m), 735 (m), 696 (s); GC-MS: t_R (Std 50) = 10.5 min, *m/z* = 178 (M^+), 108, 91 (100%), 77, 71, 65, 51.

Benzyl octanoate (3da). Pale yellow liquid; ^{1}H NMR (300 MHz, $CDCl_3$): δ (ppm) = 7.49–7.20 (m, 5H, ArH), 5.11 (s, 2H, OCH_2), 2.35 (t, *J* = 7.5 Hz, 2H, CH_2), 1.64 (qunit, *J* = 7.2 Hz, 2H, CH_2), 1.35 – 1.20 (m, 8H, 4xCH_2), 0.94 – 0.78 (m, 3H, CH_3); ^{13}C NMR (75 MHz, $CDCl_3$): δ (ppm) = 173.7 (C=O), 136.2 (ArC), 128.5 (2xArCH), 128.2 (3xArCH), 66.0 (OCH_2), 34.3 (CH_2), 31.7 (CH_2), 29.1 (CH_2), 28.9 (CH_2), 25.0 (CH_2), 22.6 (CH_2), 14.1 (CH_3); IR (ATR): $\tilde{\nu}$ [cm^{-1}] = 2955 (w), 2926 (m), 2857 (w), 1736 (s), 1497 (w), 1456 (m), 1379 (w), 1258 (m),1213 (m), 1155 (s), 1103 (m), 733 (m), 696 (s); GC-MS: t_R (Std 50) = 13.0 min, *m/z* = 234 (M^+), 143, 125, 108, 91 (100%), 77, 65, 57.

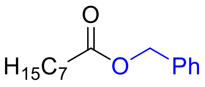

3da

Benzyl isovalerate (3ea). Pale yellow liquid; ^{1}H NMR (300 MHz, $CDCl_3$): δ (ppm) = 7.40– 7.25 (s, 5H, ArH), 5.11 (s, 2H, OCH_2), 2.24 (m, 2H, CH_2), 2.12 (m, 1H, CH), 0.95 (d, *J* = 6.5 Hz, 6H, 2xCH_3); ^{13}C NMR (75 MHz, $CDCl_3$): δ (ppm) = 172.9 (C=O), 136.2 (ArC), 128.5 (2xArCH), 128.2 (2xArCH), 66.0 (OCH_2), 43.4 (CH_2), 25.7 (CH), 22.4 (2xCH_3); IR (ATR): $\tilde{\nu}$ [cm^{-1}] = 3034 (w), 2959 (m), 2872 (w), 1732 (s), 1458 (m), 1377 (w), 1292 (m), 1254 (m), 1182 (m), 1165 (s), 1119 (m), 1094 (m), 999 (m), 737 (m), 696 (s); GC-MS: t_R (Std 50) = 10.9 min, *m/z* = 192 (M^+), 108, 91 (100%), 85, 77, 65, 57, 51.

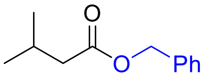

3ea

Benzyl 3,3-dimethylbutanoate (3fa). Pale yellow liquid; ^{1}H NMR (300 MHz, $CDCl_3$): δ (ppm) = 7.42–7.25 (m, 5H, ArH), 5.11 (s, 2H, OCH_2), 2.26 (s, 2H, CH_2), 1.04 (s, 9H, 3xCH_3); ^{13}C NMR (75 MHz, $CDCl_3$): δ (ppm) = 172.4 (C=O), 136.2 (ArC), 128.5 (2xArCH), 128.3 (2xArCH), 128.1 (ArCH), 65.9 (OCH_2), 48.0 (OCH_2), 30.8 (C_q), 30.0 (CH_3); IR (ATR): $\tilde{\nu}$ [cm^{-1}] = 3034 (w), 2957 (m),2870 (w), 1732 (s), 1456 (w), 1368 (m), 1321 (m), 1223 (s), 1125 (s), 995 (m), 737 (m), 696 (s); GC-MS: t_R (Std 50) = 11.1 min, *m/z* = 206 (M^+), 131, 108, 99, 91 (100%), 77, 65, 57.

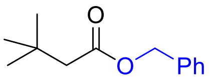

3fa

Cyclohexyl 2-ethylhexanoate (3ar). Pale yellow liquid; ^{1}H NMR (300 MHz, $CDCl_3$): δ (ppm) = 4.79 (m, 1H, OCH), 2.22 (m, 1H, CH), 1.86–1.19 (m, 18H, 9xCH_2), 0.89 (t, *J* = 7.4 Hz, 3H, CH_3), 0.88 (t, *J* = 7.0 Hz, 3H, CH_3); ^{13}C NMR (75 MHz, $CDCl_3$): δ (ppm) = 175.8 (C=O), 71.9 (OCH), 47.5 (CH), 31.9 (CH_2), 31.7 (2xCH_2), 29.6 (CH_2), 25.6 (CH_2), 25.4 (CH_2), 23.7 (2xCH_2), 22.6 (CH_2), 13.9 (CH_3), 11.8 (3xCH_3); IR (ATR): $\tilde{\nu}$ [cm^{-1}] = 2934 (m), 2859 (m), 1728 (s), 1452 (m), 1385 (w), 1261 (m), 1173 (s), 1144 (m), 1123 (w), 1040 (w), 1017 (w), 968 (w); GC-MS: t_R (Std 50) = 11.7 min, *m/z* = 170, 145 (100%), 127, 99, 88, 83, 73, 67, 55.

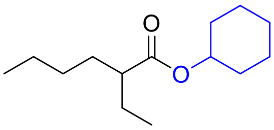

3ar

Isopropyl 2-ethylhexanoate (3as). Colorless liquid; ^{1}H NMR (300 MHz, $CDCl_3$): δ (ppm) = 5.04 (sept, *J* = 6.3 Hz, 1H, OCH), 2.21 (m, 1H, CH), 1.68–1.38(m, 4H, 2xCH_2), 1.37–1.22 (m, 4H, 2xCH_2), 1.23 (d, *J* = 6.3 Hz, 6H, 2xCH_3), 0.91–0.85 (m, 6H, 2xCH_3); ^{13}C NMR (75 MHz, $CDCl_3$): δ (ppm) = 175.9 (C=O), 67.0 (OCH), 47.5 (CH), 31.8(CH_2), 29.6 (CH_2), 25.5 (CH_2), 22.6 (CH_2), 21.9 (2xCH_3), 13.92 (CH_3), 11.8 (CH_3); IR (ATR): $\tilde{\nu}$ [cm^{-1}] = 2963 (m), 2934 (m), 2876 (w), 2862 (w), 1728 (s), 1458 (m), 1373 (m), 1265 (m), 1177 (s), 1146 (m), 1107 (s), 959 (w), 822 (w); GC-MS: t_R (Std 50) = 8.8 min, *m/z* = 158, 145, 130, 116, 99, 88, 73, 57 (100%).

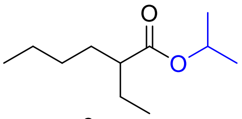

3as

1,1,1,3,3,3-Hexafluoropropan-2-yl 2-ethylhexanoate (3at). Colorless liquid; ^{1}H NMR (300 MHz, $CDCl_3$): δ (ppm) = 5.80 (sept, *J* = 6.2 Hz, 1H, OCH), 2.49 (m, 1H, CH), 1.78–1.49 (m, 4H, 2xCH_2), 1.40–1.20 (m, 4H, 2xCH_2), 0.92 (t, *J* = 7.5 Hz, 3H, CH_3), 0.89 (t, *J* = 7.0 Hz, 3H, CH_3); ^{13}C NMR (75 MHz, $CDCl_3$): δ (ppm) = 172.9, 120.5 (q, *J* = 281.8 Hz, CF_3), 66.1 [sept, *J* = 34.6 Hz, $CH(CF_3)_2$], 46.9 (CH), 31.4 (CH_2), 29.1 (CH_2), 25.3 (CH_2), 22.4 (CH_2), 13.7 (CH_3), 11.3 (CH_3); ^{19}F NMR (282 MHz, $CDCl_3$): δ (ppm) = –73.4 (CF_3); IR (ATR): $\tilde{\nu}$ [cm^{-1}] = 2967 (w), 2940 (w), 2866 (w), 1775 (m), 1464 (w), 1387 (m), 1354 (w), 1288 (m), 1225 (m), 1198 (s), 1101 (s), 907 (m), 729 (w), 691 (m); GC-MS: t_R (Std 50) = 6.1 min, *m/z* = 294 (M^+), 266, 251, 238 (100%), 223, 203, 183, 127, 99, 83, 69, 55.

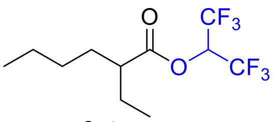

3at

Phenyl 2-ethylhexanoate (3au). Colorless liquid; ^{1}H NMR (300 MHz, $CDCl_3$): δ (ppm) = 7.44–7.30 (m, 2H, ArH), 7.28–7.14 (m, 1H, ArH), 7.14–7.00 (m, 2H, ArH), 2.51 (m, 1H, CH), 1.90–1.50 (m, 4H, 2xCH_2), 1.46–1.29 (m, 4H, 2xCH_2), 1.02 (t, *J* = 7.4 Hz, 3H, CH_3), 0.95–0.90 (m, 3H, CH_3); ^{13}C NMR (75 MHz, $CDCl_3$): δ (ppm) = 174.8 (C=O), 150.9 (ArC), 129.4 (2xArCH), 125.7 (ArCH), 121.6 (2xArCH), 47.4 (CH), 31.8 (CH_2), 29.7 (CH_2), 25.6 (CH_2), 22.7 (CH_2), 14.0 (CH_3), 11.9 (CH_3); IR (ATR): $\tilde{\nu}$ [cm^{-1}] = 2961 (m), 2934 (m), 2874 (w), 2860 (w), 1755 (s), 1593 (m), 1493 (m), 1456 (m), 1375 (w), 1192 (s), 1159 (s), 1111 (s), 1070 (m), 820 (w), 743 (m), 689 (s); GC-MS: t_R (Std 50) = 11.1 min, *m/z* =

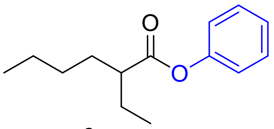

3au

1-Phenylethyl 2-ethylhexanoate (3av). Pale yellow liquid; d.r. = 1:1; ^{1}H NMR (300 MHz, $CDCl_3$): δ (ppm) = δ 7.40–7.22 (m, 5H, ArH), 5.92 (q, *J* = 6.6 Hz, 1H, OCH), 2.28 (m, 1H, CH), 1.72–1.39 (m, 4H, 2xCH_2), 1.53 (d, *J* = 6.7 Hz, 3H, CH_3), 1.35–1.10 (m, 4H, 2xCH_2), 0.91 – 0.80 (m, 6H, 2xCH_3); ^{13}C NMR (75 MHz, $CDCl_3$): δ (ppm) = 175.6 (C=O), 141.9 (ArC), 128.4 (2xArCH), 127.7 (ArCH), 126.1 (2xArCH), 71.74 (OCH), 47.5 and 47.4 (CH, 2 diastereomers), 31.8 (CH_2), 29.6 and 29.5 (CH_2, 2 diastereomers), 25.5 (CH_2), 22.6 (CH_2), 22.2 (CH_3), 13.9 (CH_3), 11.81 and 11.78 (CH_3, 2 diastereomers); IR (ATR): $\tilde{\nu}$ [cm^{-1}] = 2961 (m), 2932 (m), 2860 (w), 1730 (s), 1495 (w), 1456 (m), 1375 (m), 1263 (m), 1169 (s), 1142 (m), 1063 (m), 953 (w), 758 (m), 696 (s); GC-MS: t_R (Std 80) = 10.86 and 10.90 min, *m/z* = 248 (M^+), 143, 122, 105 (100%), 91, 77, 57.

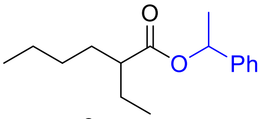

3av

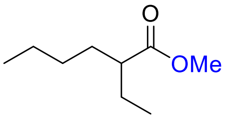

3ax

Methyl 2-ethylhexanoate (3ax). Colorless liquid; ^{1}H NMR (300 MHz, $CDCl_3$): δ (ppm) = 3.67 (s, 3H, OCH_3), 2.27 (m, 1H, CH), 1.69–1.39 (m, 4H, 2xCH_2), 1.36–1.18 (m, 4H, 2xCH_2), 0.88 (t, J = 7.3 Hz, 6H, 2xCH_3); ^{13}C NMR (75 MHz, $CDCl_3$): δ (ppm) = 176.9 (C=O), 51.2 (OCH_3), 47.3 (CH), 31.8 (CH_2), 29.6 (CH_2), 25.5 (CH_2), 22.6 (CH_2), 13.9 (CH_3), 11.8 (CH_3); IR (ATR): $\tilde{\nu}$ [cm^{-1}] = 2959 (m), 2934 (m), 2862 (w), 1736 (s), 1458 (m), 1435 (m), 1375 (w), 1263 (m), 1192 (m), 1167 (m), 1146 (m), 999 (w), 799 (w); GC-MS: t_R (Std 50) = 7.7 min, m/z = 130, 115, 102, 87 (100%), 69, 57.

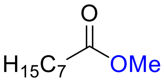

3dx

Methyl octanoate (3dx). Pale yellow liquid; ^{1}H NMR (300 MHz, $CDCl_3$): δ (ppm) = 3.66 (s, 3H, OCH_3), 2.30 (t, J = 7.5 Hz, 2H, CH_2), 1.62 (m, 2H, CH_2), 1.40–1.20 (m, 8H, 4xCH_2), 0.95–0.82 (m, 3H, CH_3); ^{13}C NMR (75 MHz, $CDCl_3$): δ (ppm) = 174.3 (C=O), 51.4 (OCH_3), 34.1 (CH_2), 31.6 (CH_2), 29.1 (CH_2), 28.9 (CH_2), 24.9 (CH_2), 22.6 (CH_2), 14.0 (CH_3); IR (ATR): $\tilde{\nu}$ [cm^{-1}] = 2928 (m), 2857 (w), 1740 (s), 1435 (m), 1196 (m), 1165 (s), 1107 (m), 725 (w); GC-MS: t_R (Std 50) = 8.7 min, m/z = 127, 115, 101, 87, 74 (100%), 69, 55.

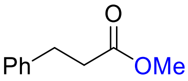

3gx

Methyl 3-phenylpropanoate (3gx). Pale yellow liquid; ^{1}H NMR (300 MHz, $CDCl_3$): δ (ppm) = 7.34–7.24 (m, 2H, ArH), 7.24–7.15 (m, 3H, ArH), 3.65 (s, 3H, OCH_3), 2.94 (t, J = 7.8 Hz, 2H, CH_2), 2.62 (t, J = 7.8 Hz, 2H, CH_2); ^{13}C NMR (75 MHz, $CDCl_3$): δ (ppm) = 173.3 (C=O), 140.5 (ArC), 128.5 (2xArCH), 128.3 (2xArCH), 126.3 (ArCH), 51.6 (OCH_3), 35.7 (CH_2), 31.0 (CH_2); IR (ATR): $\tilde{\nu}$ [cm^{-1}] = 3028 (w), 2951 (w), 1734 (s), 1497 (w), 1435 (m), 1364 (w), 1194 (m), 1161 (s), 1028 (m), 986 (m), 750 (m), 698 (s); GC-MS: t_R (Std 50) = 10.0 min, m/z = 164 (M^+), 133, 104 (100%), 91, 77, 65, 51.

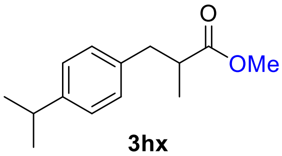

3hx

Methyl 3-(4-isopropylphenyl)-2-methylpropanoate (3hx). Pale yellow liquid; ^{1}H NMR (300 MHz, $CDCl_3$): δ (ppm) = 7.19–7.02 (m, 4H, ArH), 3.63 (s, 3H, OCH_3), 3.00 (dd, J = 13.0, 6.3 Hz, 1H, CHH), 2.87 [sept, J = 6.9 Hz, 1H, CH(CH_3)$_2$], 2.78–2.58 (m, 2H, CHH+$CH$$CH_3$), 1.23 (d, J = 6.9 Hz, 6H, CH(CH_3)$_2$), 1.14 (d, J = 6.8 Hz, 3H, CHCH_3); ^{13}C NMR (75 MHz, $CDCl_3$): δ (ppm) = 176.6 (C=O), 146.8 (ArC), 136.6 (ArC), 128.9 (2xArCH), 126.4 (2xArCH), 51.5 (OCH_3), 41.4 (CH), 39.3 (CH_2), 33.7 (CH), 24.0 (2xCH_3), 16.8 (CH_3); IR (ATR): $\tilde{\nu}$ [cm^{-1}] = 2959 (m), 2872 (w), 1736 (s), 1514 (w), 1460 (m), 1362 (w), 1281 (w), 1209 (m), 1161 (s), 1119 (m), 1055 (m), 988 (w), 814 (m), 762 (w); GC-MS: t_R (Std 50) = 11.8 min, m/z = 220 (M^+), 205, 160, 145, 133 (100%), 117, 105, 91, 77, 59.

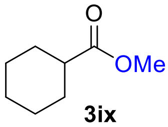

3ix

Methyl cyclohexanecarboxylate (3ix). Pale yellow liquid; ^{1}H NMR (300 MHz, $CDCl_3$): δ (ppm) = 3.66 (s, 3H), 2.30 (m, 1H, CH), 1.90 (m, 2H, CH_2), 1.80–1.68 (m, 2H, CH_2), 1.68–1.58 (m, 1H, CHH), 1.54–1.36 (m, 2H, CH_2), 1.35–1.09 (m, 3H, CHH+ CH_2); ^{13}C NMR (75 MHz, $CDCl_3$): δ (ppm) = 176.5 (C=O), 51.4 (OCH_3), 43.1 (CH), 29.0 (2xCH_2), 25.7 (CH_2), 25.4 (2xCH_2); IR (ATR): $\tilde{\nu}$ [cm^{-1}] = 2932 (m), 2855 (m), 1732 (s), 1449 (m), 1435 (m), 1314 (m), 1246 (m), 1196 (m), 1169 (s), 1134 (m), 1040 (m), 893 (w), 756 (w); GC-MS: t_R (Std 50) = 8.0 min, m/z = 142 (M^+), 127, 110, 101, 87, 83, 74, 67, 59, 55 (100%).

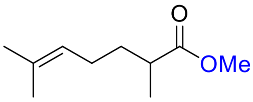

3jx

Methyl 2,6-dimethylhept-5-enoate (3jx). Colorless liquid; ^{1}H NMR (300 MHz, $CDCl_3$): δ (ppm) = 5.08 (m, 1H, C=CH), 3.67 (s, 3H, OCH_3), 2.45 (m, 1H, CH), 1.98 (q, J = 7.5 Hz, 2H, CH_2), 1.68 (m, 1H, CHH), 1.59 (s, 3H, CH_3), 1.59 (s, 3H, CH_3), 1.51–1.35 (m, 1H, CHH), 1.15 (d, J = 7.0 Hz, 3H, CH_3); ^{13}C NMR (75 MHz, $CDCl_3$): δ (ppm) = 177.2 (C=O), 132.1 (C_q), 123.6 (=CH), 51.4 (OCH_3), 38.9 (CH), 33.8 (CH_2), 25.67 (CH_2), 25.64 (CH_3), 17.6 (CH_3), 17.0 (CH_3); IR (ATR): $\tilde{\nu}$ [cm^{-1}] = 2970 (m), 2932 (m), 2856 (w), 1736 (s), 1435 (m), 1377 (m), 1196 (m), 1157 (s), 1121 (m), 1063 (m), 837 (w), 750 (w); GC-MS: t_R (Std 50) = 8.9 min, m/z = 170 (M^+), 138, 110, 95, 88 (100%), 83, 69, 57.

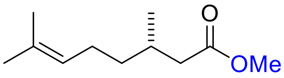

3kx

Methyl (*S*)-(-)-citronellate (3kx). Colorless liquid; ^{1}H NMR (300 MHz, $CDCl_3$): δ (ppm) = 5.09 (m, 1H, C=CH), 3.67 (s, 3H, OCH_3), 2.32 (dd, J = 14.6, 5.9 Hz, 1H, CHCHH), 2.12 (dd, J = 14.6, 8.2 Hz, 1H, CHCHH), 2.00–1.90 (m, 3H, CH+CH_2), 1.68 (s, 3H, CH_3), 1.60 (s, 3H, CH_3), 1.44–1.16 (m, 2H, CH_2), 0.94 (d, J = 6.6 Hz, 3H, CH_3); ^{13}C NMR (75 MHz, $CDCl_3$): δ (ppm) = 173.9 (C=O), 131.7 (C_q), 124.4 (=CH), 51.5 (OCH_3), 41.7 (CH_2), 36.9 (CH_2), 30.2 (CH), 25.8 (CH_2), 25.6 (CH_3), 19.7 (CH_3), 17.8 (CH_3).
IR (ATR): $\tilde{\nu}$ [cm^{-1}] = 2955 (w), 2916 (m), 2853 (w), 1738 (s), 1435 (m), 1377 (w), 1366 (w), 1288 (w), 1258 (w), 1221 (w), 1194 (m), 1179 (w), 1153 (m), 1111 (w), 1082 (w), 1011 (w), 879 (w), 835 (w), 740 (w), 710 (w);
GC-MS: t_R (Std 50) = 9.8 min, m/z = 184 (M^+), 152, 110, 95, 82, 69 (100%), 55.

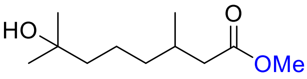

3lx

Methyl 7-hydroxycitronellate (3lx). Pale yellow liquid; ^{1}H NMR (300 MHz, $CDCl_3$): δ (ppm) = 3.67 (s, 3H, OCH_3), 2.32 (dd, J = 14.7, 6.1 Hz, 1H, CHCHH), 2.13 (dd, J = 14.7, 7.9 Hz, 1H, CHCHH), 1.97 (m, 1H, CH), 1.84 (s, 1H, OH), 1.51–1.13 (m, 6H, 3xCH_2), 1.21 (s, 6H, 2xCH_3), 0.94 (d, J = 6.6 Hz, 3H, CH_3); ^{13}C NMR (75 MHz, $CDCl_3$): δ (ppm) = 173.7 (C=O), 70.8 (C_q), 51.3 (OCH_3), 43.9 (CH_2), 41.6 (CH_2), 37.1 (CH_2), 30.3 (CH), 29.3 (CH_3), 29.1 (CH_3), 21.6 (CH_2), 19.7 (CH_3); IR (ATR): $\tilde{\nu}$ [cm^{-1}] = 3456 (br), 2963 (m), 2938 (m), 1736 (s), 1462 (m), 1437 (m), 1366 (m), 1285 (m), 1161 (s), 1089 (m), 1007 (m), 937 (m), 908 (m), 714 (w); GC-MS: t_R (Std 50) = 10.8 min, m/z = 187, 155, 144, 129, 109, 101 (100%), 95, 87, 74, 69, 59.

Methyl hexanoate (3mx). Colorless liquid; ^{1}H NMR (300 MHz, $CDCl_3$): δ (ppm) = 3.67 (s, 3H, OCH_3)), 2.30 (t, J = 7.5 Hz, 2H, CH_2), 1.63 (m, 2H, CH_2), 1.40–1.22 (m, 4H, 2xCH_2), 0.90 (t, J = 6.9 Hz, 3H, CH_3); ^{13}C NMR (75 MHz, $CDCl_3$): δ (ppm) = 174.2 (C=O), 51.3 (OCH_3), 34.0 (CH_2), 31.3 (CH_2), 24.6 (CH_2), 22.3 (CH_2), 13.8 (CH_3); IR (ATR): $\tilde{\nu}$ [cm^{-1}] = 2955 (m), 2932 (m), 2862 (w), 1738 (s), 1435 (m), 1362 (w), 1246 (m), 1169 (s), 1101 (m), 1013 (w), 860 (w), 735 (w); GC-MS: t_R (Std 50) = 5.8 min, m/z = 99, 87, 74 (100%), 59.

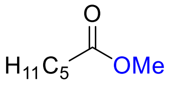

3mx

Methyl (*S*)-3-(*tert*-butyldimethylsilyloxy)-2-methylpropanoate (3nx). Colorless liquid; 66% ee (determined by HPLC on Chiralcel OD-H, 0.1% 2-propanol in hexanes; flow rate, 0.5 mL/min; oven temp, 18.0 °C; detector, UV @ 220 nm; t_R of (*S*)-enantiomer = 9.3 min and t_R of (*R*)-enantiomer = 9.9 min); ^{1}H NMR (300 MHz, $CDCl_3$): δ (ppm) = 3.78 (dd, J = 9.7, 6.9 Hz, 1H, OC*H*H), 3.68 (s, 3H, OCH_3), 3.65 (dd, J = 9.7, 6.0 Hz, 1H, OCH*H*), 2.65 (m, 1H, CH), 1.14 (d, J = 7.0 Hz, 3H, CHC*H*$_3$), 0.87 [s, 9H, C(C*H*$_3$)$_3$], 0.04 (m, 6H, 2xCH_3); ^{13}C NMR (75 MHz, $CDCl_3$): δ (ppm) = 175.4 (C=O), 65.2 (OCH_2), 51.5 (OCH_3), 42.5 (CH), 25.8 [C(*C*H$_3$)$_3$], 18.2 [*C*(CH$_3$)$_3$], 13.4 (CH_3), -5.5 (2xSiCH_3); IR (ATR): $\tilde{\nu}$ [cm^{-1}] = 3125 (w), 2988 (w), 2951 (m), 2930 (m), 2857 (m), 1740 (s), 1584(s), 1462 (m), 1435 (m), 1389 (m), 1362 (m), 1256 (s), 1198 (s), 1175 (m), 1092 (s), 1061 (s), 937 (w), 833 (s); GC-MS: t_R (Std 50) = 9.7 min, m/z = 217, 201, 175 (100%), 147, 119, 89, 75, 59.

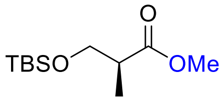

3nx

Methyl benzoate (3px). Pale yellow liquid; ^{1}H NMR (300 MHz, $CDCl_3$): δ (ppm) = 8.10–8.00 (m, 2H, ArH), 7.59–7.51 (m, 1H, ArH), 7.47–7.39 (m, 2H, ArH), 3.91 (s, 3H, OCH_3); ^{13}C NMR (75 MHz, $CDCl_3$): δ (ppm) = 167.1 (C=O), 132.9 (ArCH), 130.2 (ArC), 129.6 (2xArCH), 128.3 (2xArCH), 52.1 (OCH_3); IR (ATR): $\tilde{\nu}$ [cm^{-1}] = 3065 (w), 2999 (w), 2951 (w), 1717 (s), 1601 (w), 1452 (m), 1435 (m), 1315 (m), 1271 (s), 1177 (m), 1109 (s), 1070 (m), 1026 (m), 964 (w), 822 (w), 706 (s), 687 (m), 675 (m); GC-MS: t_R (Std 50) = 8.4 min, m/z = 136 (M^+), 105 (100%), 71, 51.

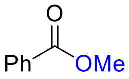

3px

Methyl 2-methylcinnamate (5ax). Yellow solid; m.p. 38–39 °C (Lit.[5]: 40–41 °C); ^{1}H NMR (300 MHz, $CDCl_3$): δ (ppm) = 7.69 (q, J = 1.4 Hz, 1H, CH), 7.42–7.37 (m, 4H, ArH), 7.37–7.29 (m, 1H, ArH), 3.82 (s, 3H, OCH_3), 2.12 (d, J = 1.4 Hz, 3H, CH_3); ^{13}C NMR (75 MHz, $CDCl_3$): δ (ppm) = 169.3 (C=O), 139.1 (CH), 136.0 (ArC), 129.8 (2xArCH), 128.5 (2xArH), 128.5 (ArCH), 52.1 (OCH_3), 14.2 (CH_3); IR (ATR): $\tilde{\nu}$ [cm^{-1}] = 2994 (w), 2947 (w), 2843 (w), 1701 (s), 1632 (w), 1489 (w), 1449 (m), 1435 (m), 1393 (w), 1358 (w), 1325 (w), 1314 (w), 1296 (m), 1254 (s), 1215 (m), 1194 (m), 1163 (w), 1115 (s), 1078 (w), 1026 (w), 1016 (w), 999 (w), 951 (m), 934 (m), 907 (w), 854 (w), 818 (w), 766 (s), 739 (m), 708 (s), 692 (s); GC-MS: t_R (Std 50) = 11.1 min, m/z = 176.1 (M^+), 145, 115 (100%), 91, 63, 51.

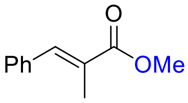

5ax

Methyl 2-*n*-pentylcinnamate (5bx). Yellow liquid; ^{1}H NMR (300 MHz, $CDCl_3$): δ (ppm) = 7.65 (s, 1H, CH), 7.43–7.26 (m, 5H, ArH), 3.81 (s, 3H, OCH_3), 2.51 (m, 2H, CH_2), 1.61–1.50 (m, 2H, CH_2), 1.40–1.26 (m, 4H, 2xCH_2), 0.95–0.83 (m, 3H, CH_3); ^{13}C NMR (75 MHz, $CDCl_3$): δ (ppm) = 169.0 (C=O), 138.7 (CH), 135.9 (ArC), 133.7 (ArC), 129.2 (2xArCH), 128.4 (2xArCH), 128.3 (ArCH), 51.9 (OCH_3), 31.9 (CH_2), 29.0 (CH_2), 27.5 (CH_2), 22.4 (CH_2), 14.0 (CH_3); IR (ATR): $\tilde{\nu}$ [cm^{-1}] = 2953 (w), 2928 (w), 2859 (w), 1711 (s), 1630 (w), 1498 (w), 1447 (w), 1433 (m), 1381 (w), 1317 (w), 1288 (w), 1279 (w), 1246 (s), 1215 (m), 1198 (s), 1159 (w), 1128 (s), 1063 (w), 928 (w), 819 (w), 766 (m), 748 (w), 700 (s); GC-MS: t_R (Std 50) = 12.9 min, m/z = 232 (M+), 201, 172, 143, 129, 115 (100%), 91, 77.

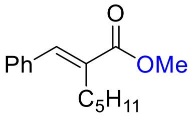

5bx

Methyl hex-2-enoate (5cx). Colorless liquid; ^{1}H NMR (300 MHz, $CDCl_3$): δ (ppm) = 6.97 (dt, J = 15.6, 7.0 Hz, 1H, CH_2C*H*=CH), 5.82 (dt, J = 15.6, 1.6 Hz, 1H, CH_2CH=C*H*), 3.73 (s, 3H, OCH_3), 2.18 (m, 2H, CH_2), 1.49 (sext, J = 7.4 Hz, 2H, CH_2), 0.94 (t, J = 7.4 Hz, 3H, CH_3); ^{13}C NMR (75 MHz, $CDCl_3$): δ (ppm) = 167.3 (C=O), 149.7 (CH), 121.1 (CH), 51.5 (OCH_3), 34.3 (CH_2), 21.4 (CH_2), 13.8 (CH_3); IR (ATR): $\tilde{\nu}$ [cm^{-1}] = 2961 (w), 2933 (w), 2874 (w), 1722 (s), 1657 (m), 1458 (w), 1435 (m), 1339 (w), 1321 (w), 1312 (w), 1269 (m), 1225 (m), 1192 (m), 1173 (s), 1124 (m), 1049 (w), 1024 (m), 978 (m), 920 (w), 882 (w), 837 (w), 716 (w); GC-MS: t_R (Std 50) = 6.6 min, m/z = 128 (M^+), 113, 97, 87, 68, 55 (100%).

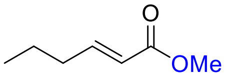

5cx

Methyl 3,7-dimethylocta-2,6-dienoate (5dx). Colorless liquid; *E*/*Z* ~ 1:1; ^{1}H NMR (300 MHz, $CDCl_3$): δ (ppm) = 5.66 (m, 1H, C=CH), 5.20–5.01 (m, 1H, C=CH), 3.67 and 3.68 (each s, 3H, OCH_3), 2.69–2.59 (m, 2H, CH_2 of *Z*-isomer), 2.21–2.10 (m, 4H, 2xCH_2 of *E*-isomer and 2H, CH_2 of *Z*-isomer), 2.16 (d, J = 1.1 Hz, 3H, C=CH_3 of *E*-isomer), 1.89 (d, J = 1.3 Hz, 3H, C=CH_3 of *Z*-isomer), 1.68 (s, 3H, CH_3), 1.61 and 1.62 (each s, 3H, CH_3); ^{13}C NMR (75 MHz, $CDCl_3$): δ (ppm) = 167.2 and 166.7 (C=O), 160.5 and 160.1 (C_q), 132.5 and 132.1 (C_q), 123.6 and 122.9 (CH), 115.8 and 115.2 (CH), 50.7 (OCH_3), 40.9, 33.4, 26.8, 26.0 (2xCH_2 of 2 isomers), 25.6 and 25.3 (CH_3), 18.8 (CH_3), 17.64 and 17.57 (CH_3); IR (ATR): $\tilde{\nu}$ [cm^{-1}] = 2974 (w), 2951 (w), 2912 (w), 2847 (w), 1717 (m), 1649 (m), 1606 (w), 1435 (m), 1377 (w), 1358 (w), 1322 (w), 1280 (w), 1238 (m), 1221 (m), 1188 (w), 1144 (s), 1107 (w), 1061 (w), 987 (w), 920 (w), 853 (m), 818 (w), 734 (w); GC-MS (Std 50): t_R = 10.0 min (*Z*-isomer), m/z = 182 (M^+), 151, 139, 123, 114, 107, 91, 83, 69 (100%), 53 and t_R =10.3 min (*E*-isomer), m/z = 182 (M^+), 151, 139, 123, 114, 107, 91, 83, 69 (100%), 53.

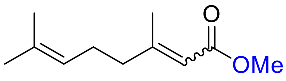

5dx

Methyl 3-methylbut-2-enoate (5ex). Colorless liquid; ^{1}H NMR (300 MHz, $CDCl_3$): δ (ppm) = 5.68 (m, 1H, CH), 3.68 (s, 3H, OCH_3), 2.17 (s, 3H, CH_3), 1.90 (s, 3H, CH_3); ^{13}C NMR (75 MHz, $CDCl_3$): δ (ppm) = 167.2 (C=O)), 156.9 (C_q), 115.8 (CH), 50.8 (OCH_3), 27.5 (CH_3), 20.3 (CH_3); IR (ATR): $\tilde{\nu}$ [cm^{-1}] = 2984 (w), 2950 (w), 2917 (w), 1719 (s), 1661 (m), 1447 (m), 1435 (m), 1379 (w), 1350 (w), 1279 (w), 1229 (s), 1188 (w), 1146 (s), 1078 (m), 1009 (w), 918 (w), 849 (m), 737 (w); GC-MS: t_R (Std 50) = 3.5 min, *m/z* = 114 (M^+), 83 (100%), 55.

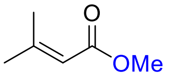

5ex

Propyl 3-methylbut-2-enoate (5ey). Colorless liquid; ^{1}H NMR (300 MHz, $CDCl_3$): δ (ppm) = 5.68 (m, 1H, CH), 4.05 (t, *J* = 6.7 Hz, 2H, OCH_2), 2.17 (d, *J* = 1.3 Hz, 3H, CH_3), 1.89 (d, *J* = 1.4 Hz, 3H, CH_3), 1.67 (sext, *J* = 7.3 Hz, 1H, CH_2), 0.95 (t, *J* = 7.4 Hz, 3H, CH_3); ^{13}C NMR (75 MHz, $CDCl_3$): δ (ppm) = 167.0 (C=O), 156.4 (C_q), 116.3 (CH), 65.3 (OCH_2), 27.5 (CH_3), 22.2 (CH_2), 20.3 (CH_3), 10.6 (CH_3); IR (ATR): $\tilde{\nu}$ [cm^{-1}] = 2970 (w), 2940 (w), 2882 (w), 1717 (s), 1649 (m), 1447 (w), 1391 (w), 1377 (w), 1348 (w), 1273 (w), 1225 (s), 1144 (s), 1076 (s), 1042 (w), 1003 (w), 986 (w), 907 (w), 851 (m), 827 (w), 770 (w), 729 (w)); GC-MS: t_R (Std 50) = 7.5 min, *m/z* = 142 (M^+), 100, 83 (100%), 55.

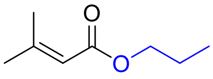

5ey

Methyl 2-methyleneoctanoate (5fx). Colorless liquid; ^{1}H NMR (300 MHz, $CDCl_3$): δ (ppm) = 6.22–6.05 (m, 1H, C=C*H*H), 5.52 (d, *J* = 1.4 Hz, 1H, C=CH*H*), 3.75 (s, 3H, OCH_3), 2.40–2.21 (m, 2H, CH_2), 1.47 (d, *J* = 7.2 Hz, 2H, CH_2), 1.29 (s, 6H, 3xCH_2), 1.00–0.76 (m, 3H, CH_3); ^{13}C NMR (75 MHz, $CDCl_3$): δ (ppm) = 167.8 (C=O), 140.8 (C_q), 124.4 (=CH_2), 51.7 (OCH_3), 31.9 (CH_2), 31.6 (CH_2), 28.9 (CH_2), 28.3 (CH_2), 22.6 (CH_2), 14.0 (CH_3); IR (ATR): $\tilde{\nu}$ [cm^{-1}] = 2953 (m), 2928 (m), 2859 (m), 1722 (s), 1630 (w), 1458 (w), 1437 (m), 1333 (w), 1290 (w), 1254 (m), 1196 (s), 1148 (s), 1113 (w), 1094 (w), 999 (w), 939 (m), 816 (m), 727 (w), 684 (w); GC-MS: t_R (Std 50) = 9.2 min, *m/z* = 170 (M^+), 139, 127, 110, 101 (100%), 95, 88, 81, 69, 55.

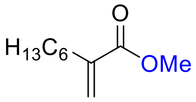

5fx

Methyl 3,7-dimethyl-2-methyleneoct-6-enoate (5gx). Colorless liquid; ^{1}H NMR (300 MHz, $CDCl_3$): δ (ppm) = 6.16 (s, 1H, C=CH*H*), 5.51 (s, 1H, C=CH*H*), 5.18–5.01 (m, 1H, C=CH), 3.75 (s, 3H, OCH_3), 2.68 (sext, *J* = 6.9 Hz, 1H, C*H* CH_3), 1.95 (m, 2H, CH_2), 1.68 (s, 3H, CH_3), 1.60–1.50 (m, 1H, C*H*H), 1.58 (s, 3H, CH_3), 1.44–1.29 (m, 1H, CH*H*), 1.08 (d, *J* = 6.9 Hz, 3H, CHC*H*$_3$); ^{13}C NMR (75 MHz, $CDCl_3$): δ (ppm) = 167.9 (C=O), 145.9 (C_q), 131.5 (C_q), 124.3 (=CH), 122.8 (=CH_2), 51.7 (OCH_3), 35.9 (CH_2), 34.4 (CH), 25.8 (CH_2), 25.7 (CH_3), 19.9 (CH_3), 17.6 (CH_3); IR (ATR): $\tilde{\nu}$ [cm^{-1}] = 2965 (m), 2926 (m), 2916 (m), 2857 (w), 1721 (s), 1626 (w), 1437 (m), 1375 (m), 1321 (w), 1273 (m), 1223 (w), 1192 (m), 1175 (m), 1142 (s), 1103 (m), 1082 (w), 1057 (w), 991 (w), 941 (m), 818 (m) 748 (w), 687 (w); GC-MS: t_R (Std 50) = 10.3 min, *m/z* = 196 (M^+), 181, 164, 154, 136, 121, 107, 93, 82, 67, 55 (100%).

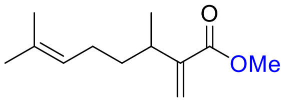

5gx

Methyl 2-benzylacrylate (5hx). Colorless liquid; ^{1}H NMR (300 MHz, $CDCl_3$): δ (ppm) = 7.33–7.24 (m, 2H, ArH), 7.24–7.15 (m, 3H, ArH), 6.28–6.19 (m, 1H, =C*H*H), 5.45 (q, *J* = 1.4 Hz, 1H, =CH*H*), 3.72 (s, 3H, OCH_3), 3.63 (s, 2H, CH_2); ^{13}C NMR (75 MHz, $CDCl_3$): δ (ppm) = 167.4 (C=O), 140.1 (C_q), 138.7 (ArC), 129.1 (2xArCH), 128.5 (2xArCH), 126.4 (ArCH), 126.3 (=CH_2), 51.9 (OCH_3), 38.1 (CH_2);IR (ATR): $\tilde{\nu}$ [cm^{-1}] = 3027 (w), 2950 (w), 1717 (s), 1632 (w), 1602 (w), 1495 (w), 1452 (w), 1437 (m), 1317 (w), 1304 (w), 1281 (m), 1254 (m), 1202 (m), 1134 (s), 1072 (w), 1029 (w), 995 (w), 947 (m), 829 (m), 814 (m), 748 (m), 700 (s); GC-MS: t_R (Std 50) = 10.4 min, *m/z* = 176 (M^+), 144, 116 (100%), 91, 65.

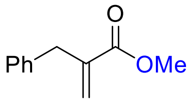

5hx

Methyl (1*R*,5*S*)-6,6-dimethylbicyclo[3.1.1]hept-2-ene-2-carboxylate (5ix). Pale yellow liquid; ^{1}H NMR (300 MHz, $CDCl_3$): δ (ppm) = 6.81 (m, 1H, C=CH), 3.72 (s, 3H, OCH_3), 2.80 (td, *J* = 5.7, 1.4 Hz, 1H, CH), 2.52–2.39 (m, 3H, CH_2+C*H*H), 2.13 (m, 1H, CH), 1.33 (s, 3H, CH_3), 1.11 (d, *J* = 9.0 Hz, 1H, CH*H*), 0.79 (s, 3H, CH_3); ^{13}C NMR (75 MHz, $CDCl_3$): δ (ppm) = 166.9 (C=O), 140.2 (=C_q), 136.6 (CH), 51.6 (OCH_3), 41.4 (CH), 40.4 (CH), 37.8 (C_q), 32.3 (CH_2), 31.5 (CH_2), 26.0 (CH_3), 21.1 ((CH_3); IR (ATR): $\tilde{\nu}$ [cm^{-1}] = 2980 (w), 2949 (w), 2918 (w), 2886 (w), 2821 (w), 1713 (s), 1626 (w), 1470 (w), 1435 (m), 1423 (w), 1366 (w), 1306 (w), 1350 (s), 1221 (w), 1209 (m), 1200 (m), 1144 (m), 1090 (m), 1072 (s), 1057 (m), 1040 (m), 966 (w), 947 (w), 889 (w), 802 (w), 752 (s), 727 (w); GC-MS: t_R (Std 50) = 10.2 min, *m/z* = 180 (M^+), 165, 149, 137, 121, 105 (100%), 91, 77, 65, 59, 51.

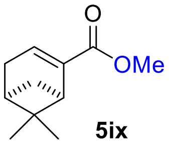

5ix

Methyl 2-methylpent-2-enoate (5jx). Colorless liquid; ^{1}H NMR (300 MHz, $CDCl_3$): δ (ppm) = 6.75 (m, 1H, C=CH), 3.73 (s, 3H, OCH_3), 2.19 (m, 2H, CH_2), 1.83 (m, 3H, CH_3), 1.05 (t, *J* = 7.6 Hz, 3H, CCH_3); ^{13}C NMR (75 MHz, $CDCl_3$): δ (ppm) = 168.9 (C=O), 144.2 (=CH), 127.1 (=C_q), 51.8 (OCH_3), 22.1 (CH_2), 22.1 (CH_3), 12.3 (CH_3); IR (ATR): $\tilde{\nu}$ [cm^{-1}] = 2968 (w), 2951 (w), 2876 (w), 1713 (s), 1649 (w), 1458 (w), 1435 (m), 1388 (w), 1357 (w), 1308 (w), 1271 (m), 1244 (s), 1190 (m), 1144 (s), 1094 (s), 1069 (w), 1040 (w), 966 (w), 953 (w), 897 (w), 825 (w), 743 (m), 660 (w); GC-MS: t_R (Std 50) = 6.4 min, *m/z* = 128 (M^+), 113, 97, 69 (100%), 67, 59, 53.

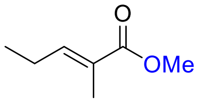

5jx

Methyl 2-ethylhex-2-enoate (5kx). Colorless liquid; ^{1}H NMR (300 MHz, $CDCl_3$): δ (ppm) = 6.73 (t, *J* = 7.5 Hz, 1H, CH=C), 3.74 (s, 3H, OCH_3), 2.32 (q, *J* = 7.5 Hz, 2H, CH_2), 2.17 (q, *J* = 7.4 Hz, 2H, CH_2), 1.48 (sext, *J* = 7.4 Hz, 2H, CH_2), 1.01 (t, *J* = 7.5 Hz, 3H, CH_3), 0.95 (t, *J* = 7.4 Hz, 3H, CH_3); ^{13}C NMR (75 MHz, $CDCl_3$): δ (ppm) = 142.2 (C=O), 133.8 (=C_q), 51.5 (OCH_3), 30.3 (CH_2), 22.1 (CH_2), 20.0 (CH_2), 13.9 (2xCH_3); IR (ATR): $\tilde{\nu}$ [cm^{-1}] = 2963 (w), 2936 (w), 2874 (w), 1736 (m), 1723 (s), 1654 (w), 1458 (w), 1435 (m), 1379 (w), 1290 (m), 1242 (s), 1219 (s), 1190 (m), 1146 (s), 1101 (m), 1047 (w), 1016 (w), 1005 (w), 968 (w), 916 (w), 795 (w), 760 (m), 714 (w), 642 (w); GC-MS: t_R (Std 50) = 8.5 min, *m/z* = 156 (M^+), 141, 125, 109, 95, 81, 67, 55 (100%).

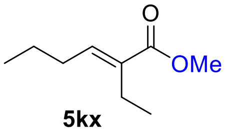

5kx

5. Gas chromatograms for the determination of the enantiomeric composition of (*S*)-3-(*tert*-butyldimethylsilyloxy)-2-methylpropanal (1n)

Column: Supelco Beta Dex 225; conditions: 63 °C, 80 min; 10 °C/min to 200 °C, 5 min; flow (N_2), 0.9 mL/min; t_R of *(R)*-enantiomer = 68.5 min and t_R of *(S)*-enantiomer = 70.3 min.

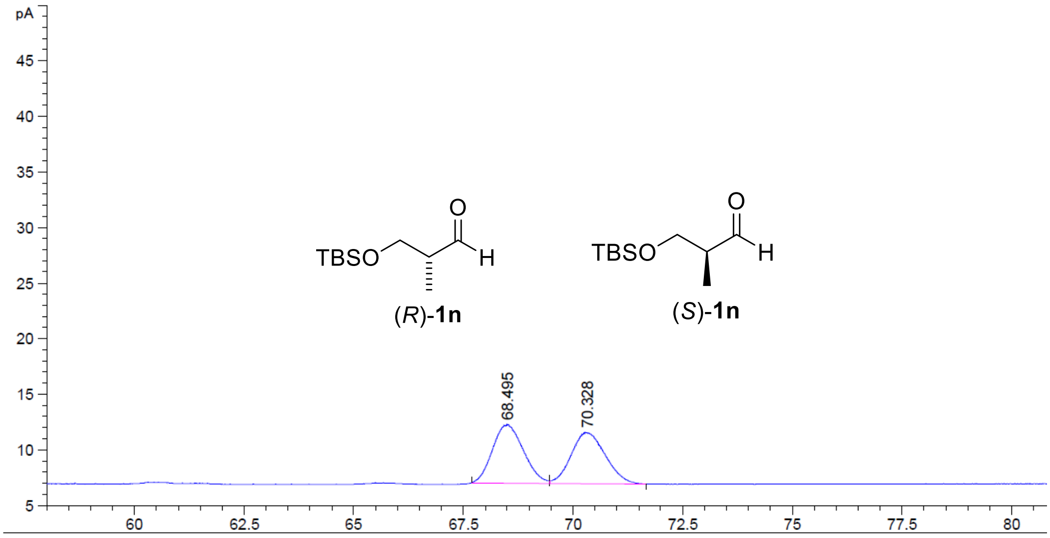

Peak #	RetTime [min]	Type	Width [min]	Area [pA*s]	Height [pA]	Area %
1	68.495	BV	0.5730	259.54944	5.31206	50.89892
2	70.328	VB	0.6335	250.38167	4.63305	49.10108

Figure S5. Gas chromatogram of *rac*-**1n**.

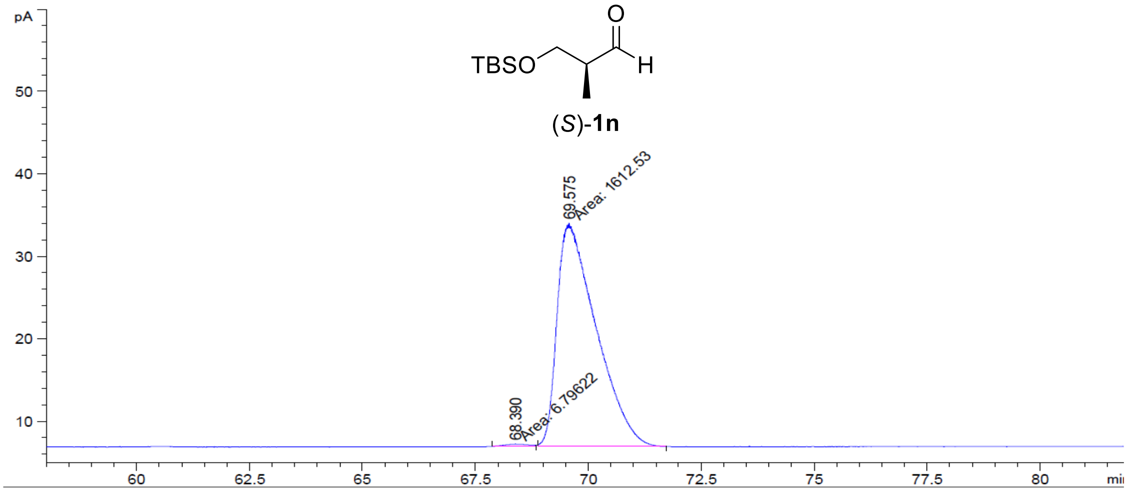

Peak #	RetTime [min]	Type	Width [min]	Area [pA*s]	Height [pA]	Area %
1	68.390	MM	0.5051	6.79622	2.24268e-1	0.41969
2	69.575	MM	0.9920	1612.52917	27.09250	99.58031

Figure S6. Gas chromatogram of *(S)*-**1n** with 99% *ee*, used as the substrate in NHC-catalysed oxidative esterification to give (*S*)-**3nx**.

6. Gas chromatograms for the determination of the enantiomeric composition of methyl (*S*)-3-(*tert*-butyldimethylsilyloxy)-2-methylpropanoate (3nx)

Column: CP-Chirasil-Dex CB; conditions: 65 °C, 85 min; 10 °C/min to 180 °C, 10 min; flow (N_2), 1.0 mL/min; t_R of (*S*)-enantiomer = 68.0 min and t_R of (*R*)-enantiomer = 73.2 min.

.

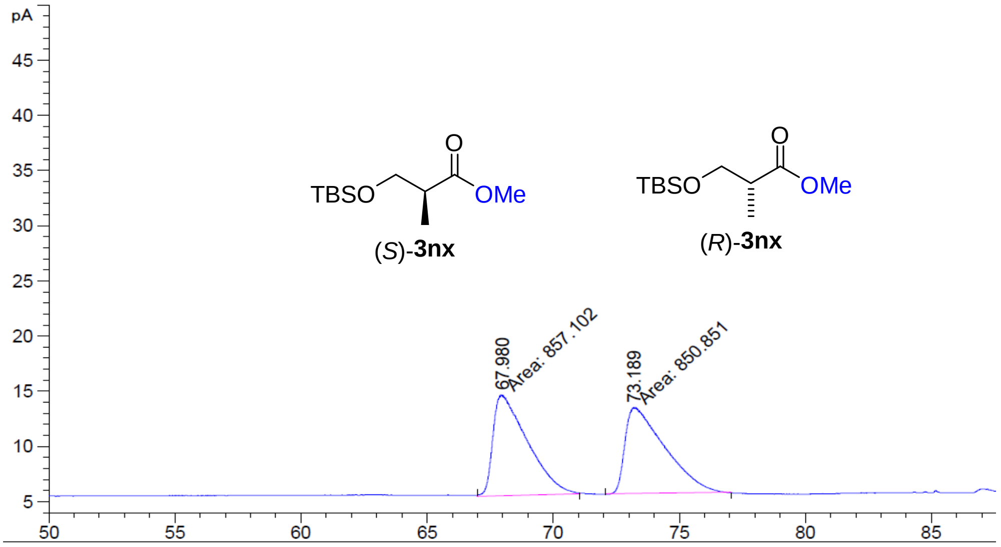

Peak #	RetTime [min]	Type	Width [min]	Area [pA*s]	Height [pA]	Area %
1	67.980	MM	1.5517	857.10236	9.20605	50.18300
2	73.189	MM	1.8217	850.85132	7.78453	49.81700

Figure S7. Gas chromatogram of *rac*-**3nx**.

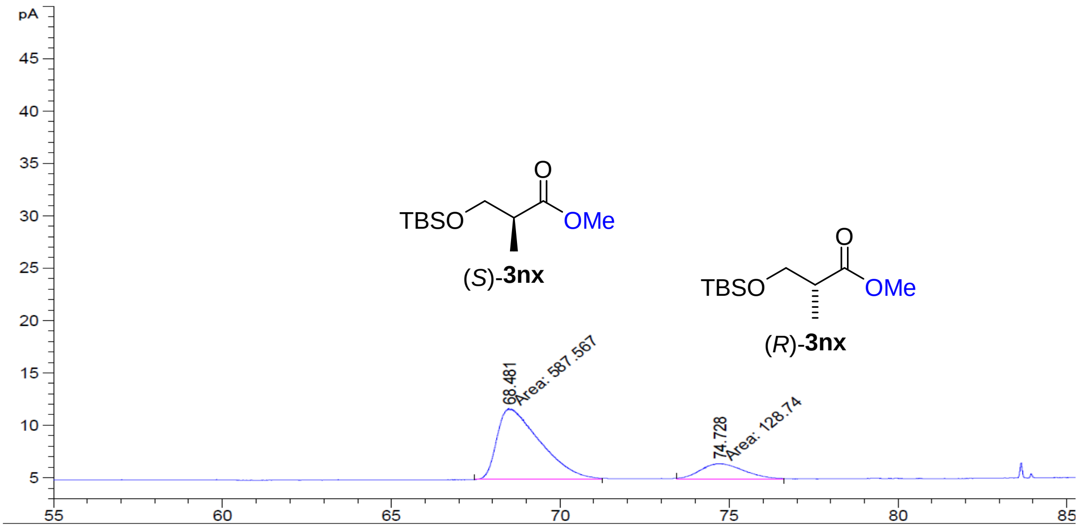

Peak #	RetTime [min]	Type	Width [min]	Area [pA*s]	Height [pA]	Area %
1	68.481	MM	1.4526	587.56671	6.74165	82.02730
2	74.728	MM	1.4652	128.73961	1.46438	17.97270

Figure S8. Gas chromatogram of (*S*)-**3nx** with 64% ee, obtained from NHC-catalysed oxidative esterification of (*S*)-**1n** (99% *ee*).

7. Investigations on the effect of BzOH on acyl azolium and azolium enolate intermediates in the esterification

7.1 Effect of BzOH on the oxidative esterification of benzaldehyde: acyl azolium intermediate

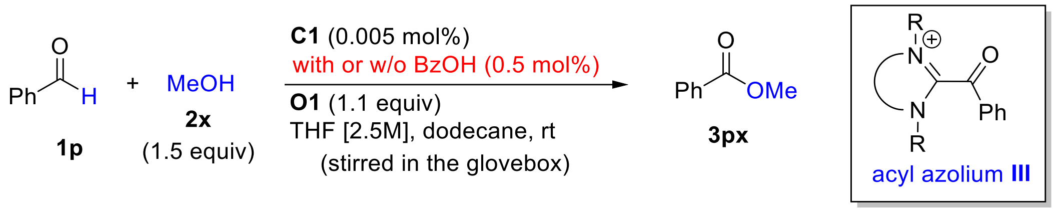

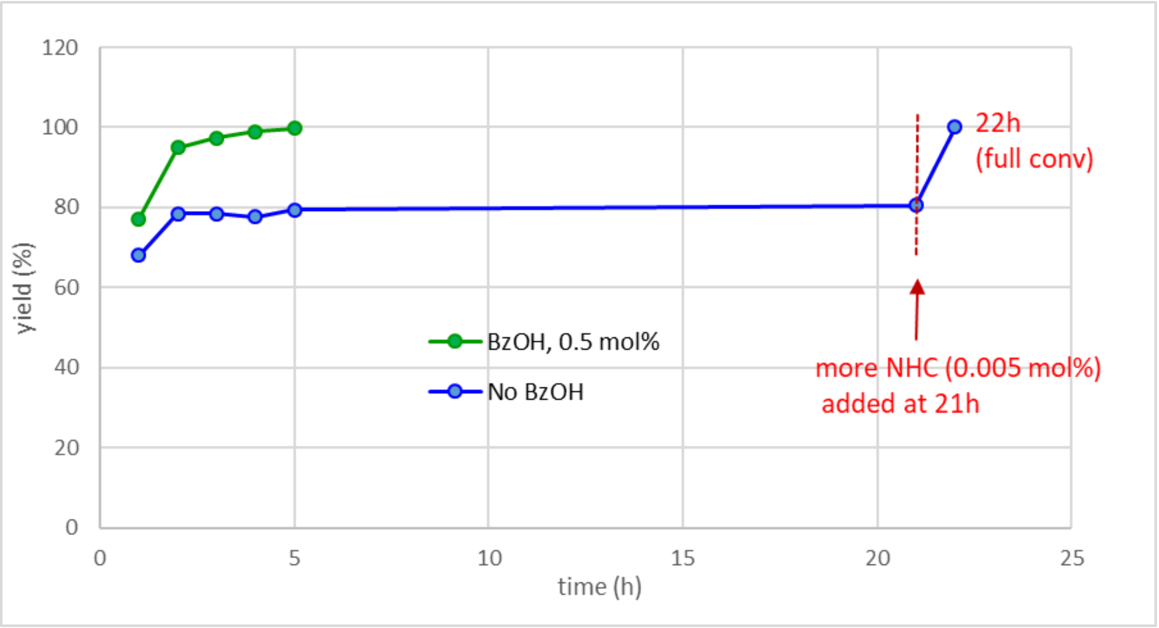

Figure S9. Kinetic study on the role of BzOH for the esterification of benzaldehyde (**1p**) with methanol (**2x**) to form **3px**. The data suggest that (i) BzOH is crucial in preventing catalyst decomposition and (ii) has little effect on the reaction rate. Yields were determined by GC using dodecane as the internal standard.

7.2 NMR studies on the reactivity of the acyl azolium triflate 6a towards benzyl alcohol in the presence of BzOH and DMAP

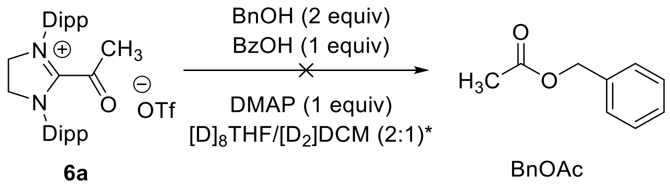

*Due to the low solubility of **6a** in $[D]_8$THF, $[D_2]$DCM was added for the complete dissolution

In a glovebox, the acyl azolium triflate **6a** was prepared according to the literature procedure.[6] An NMR tube was charged with 5 mg (8.6 µmol, 1.0 equiv) of **6a** and benzyl alcohol (**2a**, 1.8 µL, 2 equiv) in $[D_8]$THF/$[D]_2$DCM (0.4:0.2 mL) . A solution of benzoic acid (1 mg, 1 equiv) in 0.05 mL of $[D_8]$THF/$[D]_2$DCM (2:1) was added by means of a syringe, and the reaction was monitored by ^{1}H NMR spectroscopy. No transformation of the acyl azolium salt **6a** was observed up to 30 min. After 30 min, a solution of DMAP (1 mg, 1 equiv) in 0.05 mL $[D_8]$THF/$[D]_2$DCM (2:1) was added to the reaction mixture and ^{1}H NMR monitoring was continued. Even after 1.5 h, no formation of benzyl acetate could be observed (Figure S10).

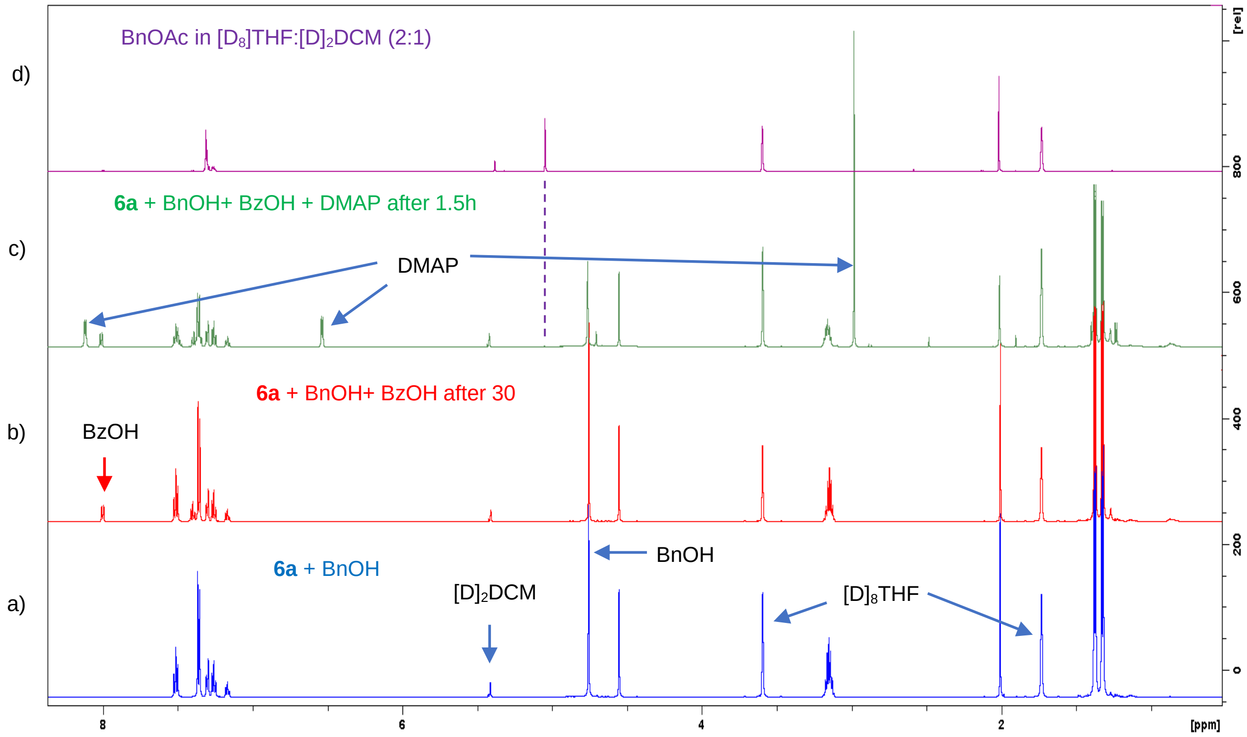

Figure S10. ^{1}H NMR (600 MHz, 298 K, [D8]THF/[D]2DCM = 2:1) spectra of a mixture of **6a** and BnOH (trace a) and of the reaction mixture after the addition of BzOH and DMAP (traces b,c). Trace d is the ^{1}H NMR (600 MHz, 298 K, [D8]THF/[D]2DCM = 2:1) spectrum of an authentic sample of benzyl acetate.

7.3 NMR studies on the reactivity of the acyl azolium chloride 6b towards methanol in the presence of BzOH

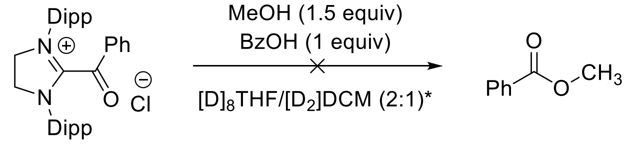

6b **3px**

*Due to the low solubility of **6b** in [D]8THF, [D2]DCM was added for the complete dissolution

In a glovebox, the acyl azolium chloride **6b** was prepared as described in the literature (see Figures S11, S12 for NMR spectra).[7] An NMR tube was charged with 10 mg (18.8 µmol, 1.0 equiv.) of **6b** in [D8]THF:[D]2DCM (0.4:0.2 mL) mixture. Methanol (1.1 µL, 1.5 equiv) was added to the solution by means of a syringe, and the mixture was monitored by ^{1}H NMR spectroscopy for 3 h at RT, no reaction was observed. A solution of benzoic acid (2.3 mg, 1 equiv) in 0.05 mL [D8]THF:[D]2DCM (2:1) was added by means of a syringe, and the mixture was again monitored by ^{1}H NMR spectroscopy. Up to three days, no formation of **3px** could be observed (Figure S13).

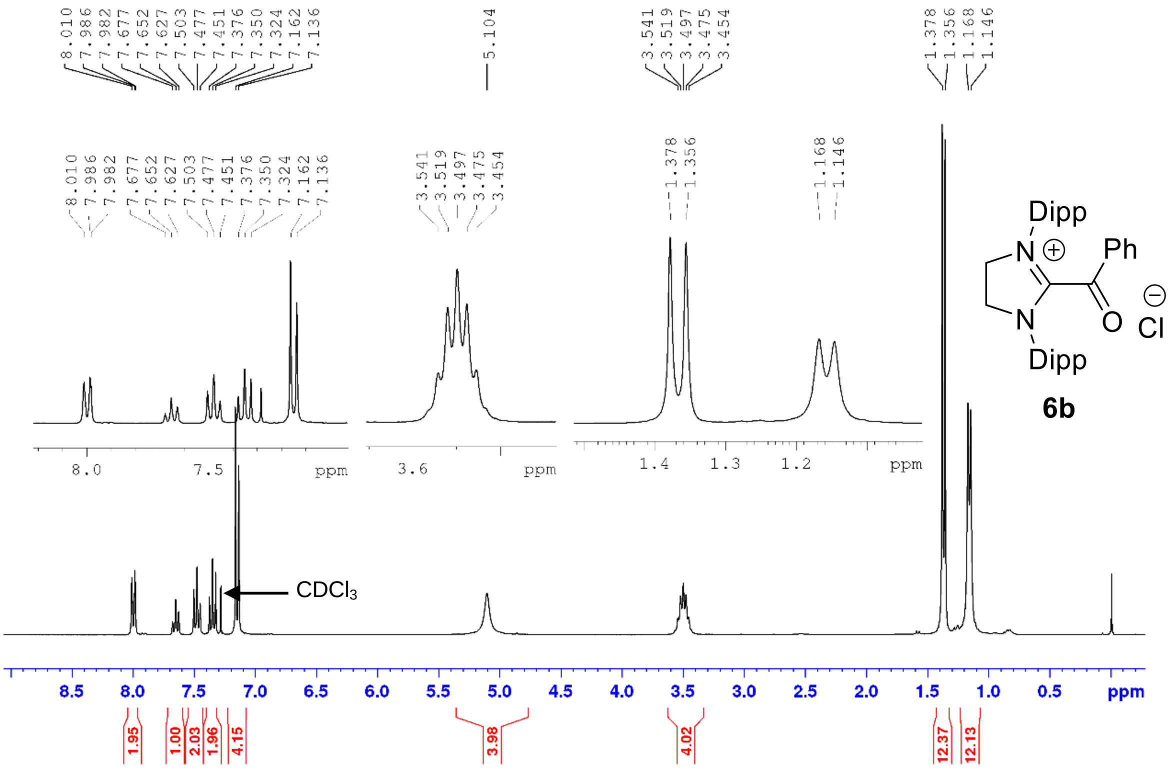

Figure S11. ^{1}H NMR (300 MHz, 298 K, $CDCl_3$) of **6b**.

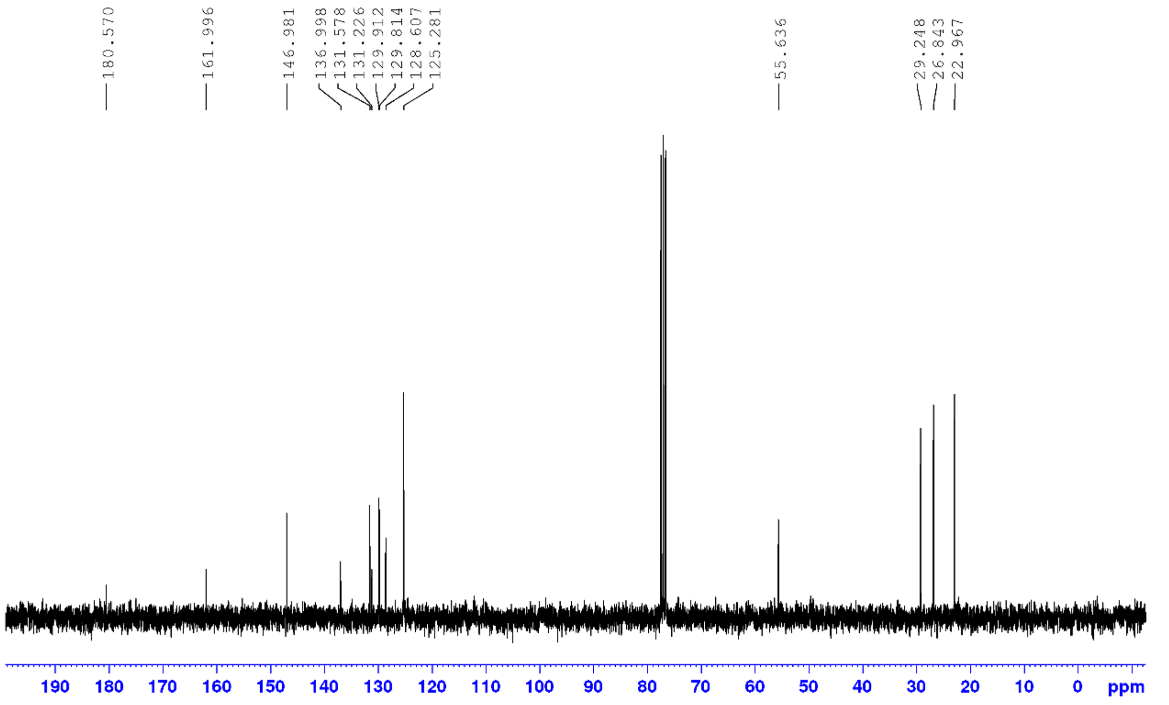

Figure S12. ^{13}C NMR (75 MHz, 298 K, $CDCl_3$) of **6b**.

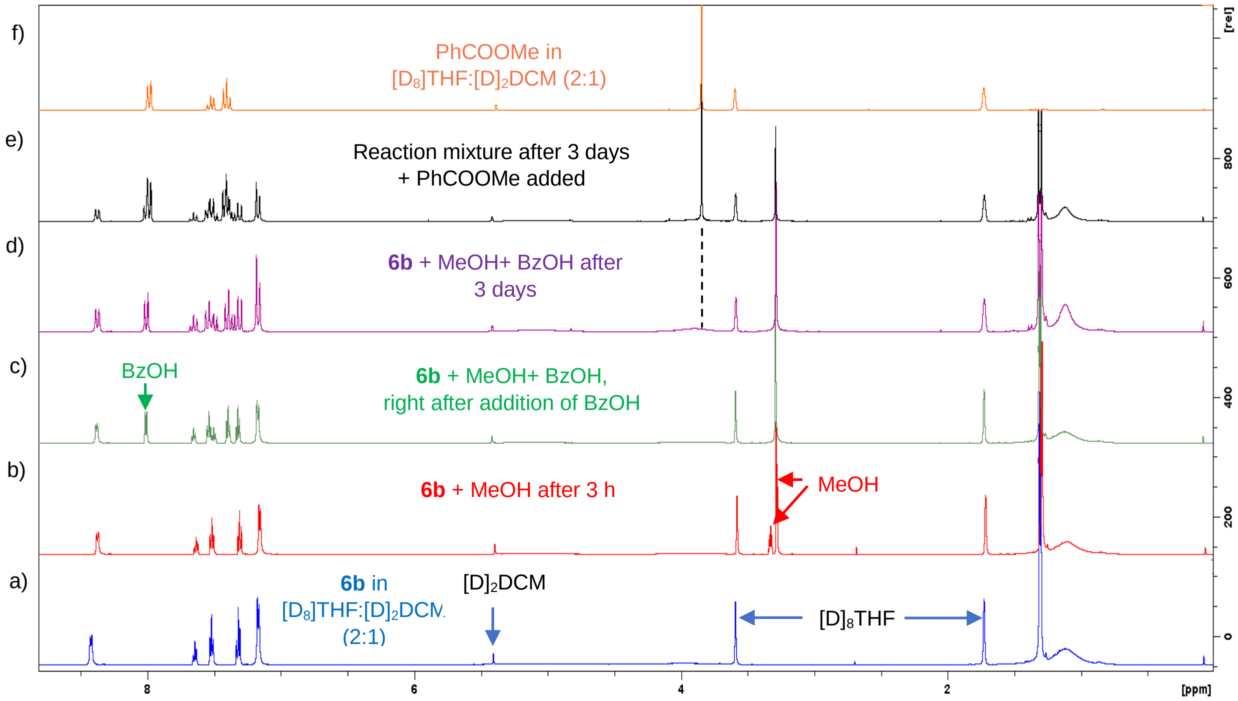

Figure S13. ^{1}H NMR (600 MHz, 298 K, [D$_8$]THF/[D]$_2$DCM = 2:1) spectra of **6b** (trace a) and of the reaction mixture after the addition of MeOH and BzOH (traces b-c). Traces d and e are the ^{1}H NMR (300 MHz, 298 K, [D$_8$]THF/[D]$_2$DCM = 2:1) spectra of the reaction mixture after 3 days and after the addition of the product methyl benzoate (**3px**), respectively. Trace f is the ^{1}H NMR (300 MHz, 298 K, [D$_8$]THF/[D]$_2$DCM = 2:1) spectrum of an authentic sample of methyl benzoate.

7.4 NMR studies on the reactivity of the azolium enolate 7 towards benzyl alcohol in the presence of benzoic acid

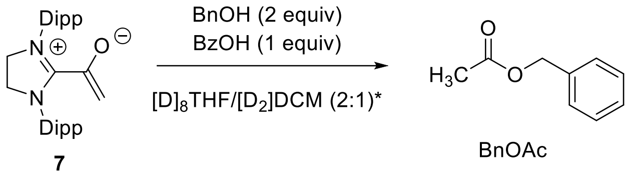

*In order to compare the reactivity of **7** to that of **6a**, [D]$_8$THF/[D$_2$]DCM (2:1) was used

In a glovebox, the azolium enolate **7** was generated and crystallized according to the literature procedure.[6] An NMR tube was charged with 7 mg (16 µmol, 1.0 equiv) of **7** in [D$_8$]THF:[D]$_2$DCM (0.4:0.1 mL) and sealed with a septum. Benzyl alcohol (2 equiv, 3.3 µL) and a solution of benzoic acid (1 equiv, 1.9 mg in 0.1 mL [D]$_2$DCM) were added simultaneously by means of syringes. The reaction was then followed by ^{1}H NMR spectroscopy, and the rapid formation of benzyl acetate could be observed (Figure S14).

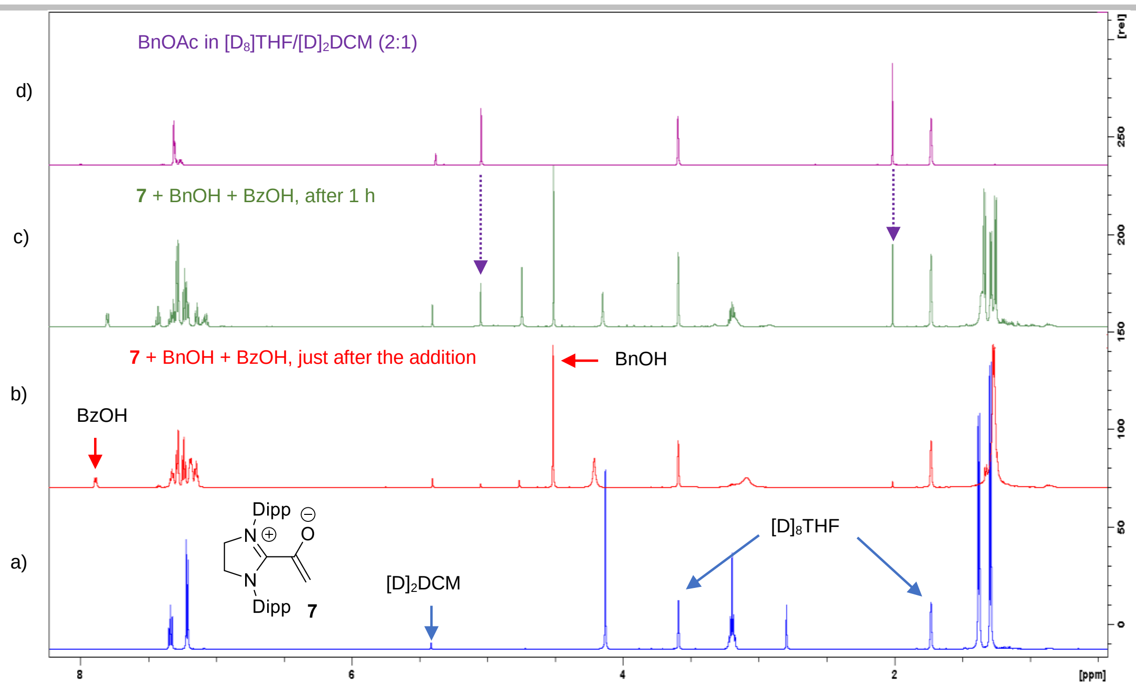

Figure S14. ^{1}H NMR (600 MHz, 298 K, $[D_8]THF/[D]_2DCM$ = 2:1) spectra of **7** (trace a), of the reaction mixture right after the addition of BnOH and BzOH (trace b), and after 1 h reaction time (trace c). Trace d is the ^{1}H NMR (600 MHz, 298 K, $[D_8]THF/[D]_2DCM$ = 2:1) spectrum of an authentic sample of benzyl acetate.

7.5 Kinetics of the reaction of the azolium enolate 7 with benzyl alcohol

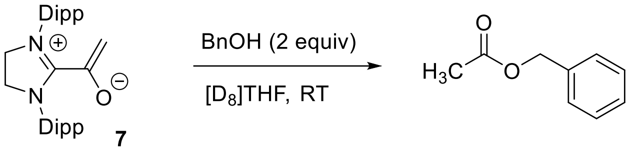

In a glovebox, the azolium enolate **7** was generated and crystallized according to the literature procedure.[6] An NMR tube was charged with 7 mg (16 µmol, 1.0 equiv) of **7** in $[D_8]THF$ (0.6 mL) and sealed with a septum. Benzyl alcohol (3.3 µL, 2 equiv) was added by means of a syringe, and the reaction was followed by ^{1}H NMR spectroscopy. Slow formation of benzyl acetate was observed (Figure S15, Table S1).

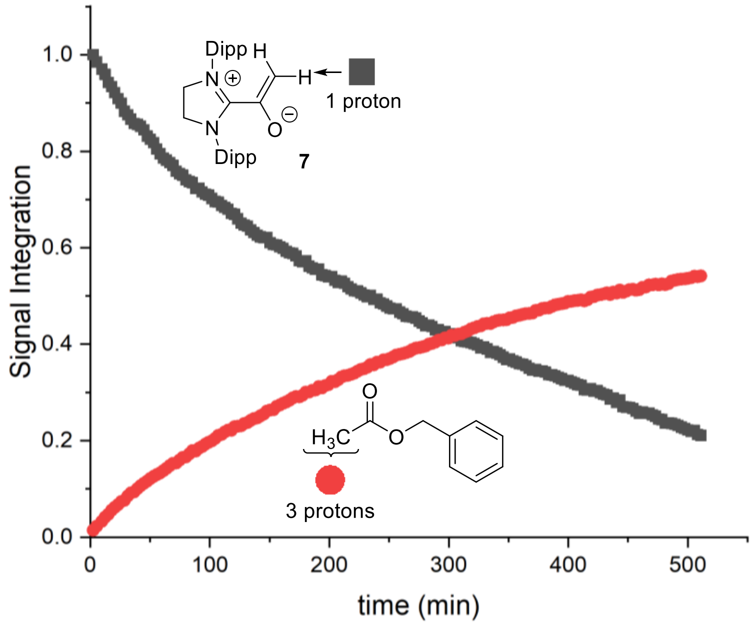

Figure S15. Kinetics of BnOAc formation from **7** and BnOH in the absence of BzOH, by monitoring the time course of the proton integral of one of the olefinic protons of **7** (black squares) and CH_3 of benzyl acetate (red dots).

Table S1. First oder rate constants (*k*) for **7** and BnOAc in the reaction of **7** and BnOH in the absence of BzOH, according to Figure S15.

Peak designation	δ [ppm]	Rate constant, *k* [1/min]	Error R^2
**7** ■	2.873	2.89E-3	1E-6
BnOAc ●	2.010	2.99E-3	5E-7

7.6 Kinetics of the reaction of the azolium enolate 7 with benzyl alcohol in the presence of benzoic acid

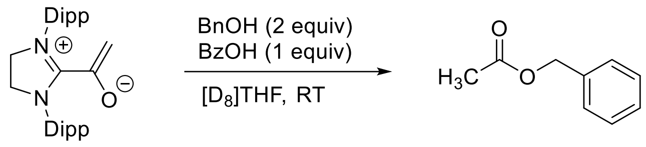

7

In a glovebox, the azolium enolate **7** was generated and crystallized according to the literature procedure.[6] An NMR tube was charged with 7 mg (16 µmol, 1.0 equiv) of **7** in [D_8]THF (0.5 mL) and sealed with a septum. Benzyl alcohol (3.3 µL, 2 equiv) and a solution of benzoic acid (1.9 mg, 1 equiv) in 0.1mL [D_8]THF were added simultaneously by means of syringes. The reaction was then followed by ^{1}H NMR spectroscopy, and the rapid formation of benzyl acetate could be observed (Figure S16, Table S2).

.

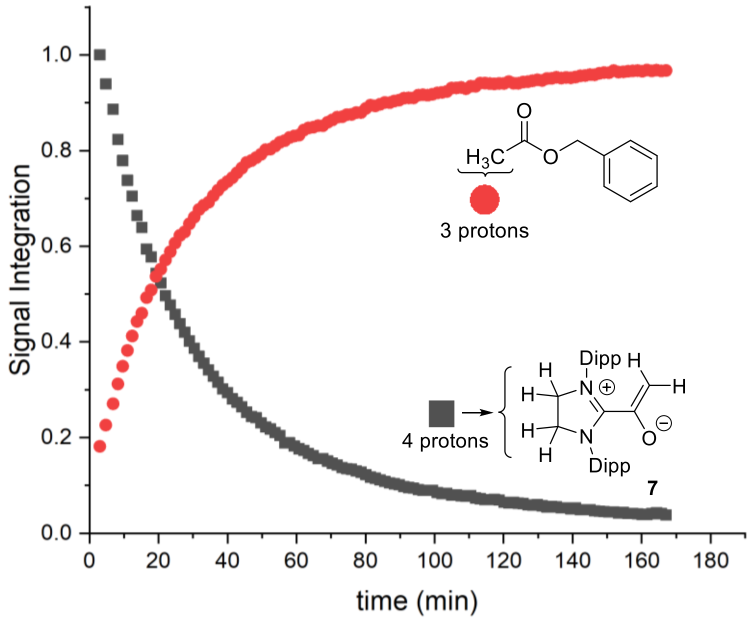

Figure S16. Kinetics of BnOAc formation from **7** and BnOH in the presence of BzOH (1 equiv), by monitoring the time course of the proton integral of the CH_2CH_2 protons of **7** (black squares) and CH_3 of benzyl acetate (red dots).

.

Table S2. First order rate constants (*k*) for **7** and BnOAc in the reaction of **7** and BnOH in the presence of BzOH, according to Figure S16.

Peak designation	δ [ppm]	Rate constant, *k* [1/min]	Error R^2
**7** ■	4.327	35.9E-3	4E-6
BnOAc ●	2.012	32.6E-3	4E-6

8. NMR spectra

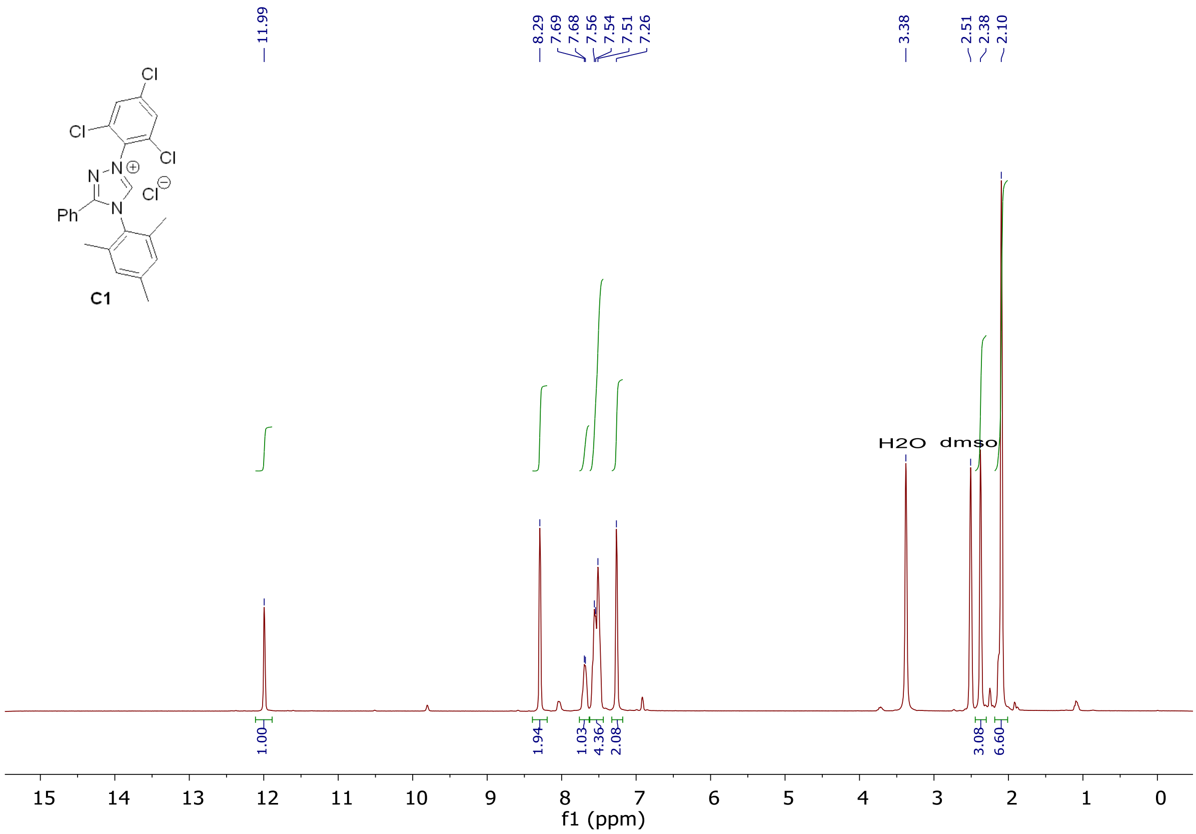

Figure S17. ^{1}H NMR (300 MHz, DMSO-*d6*) of **C1**

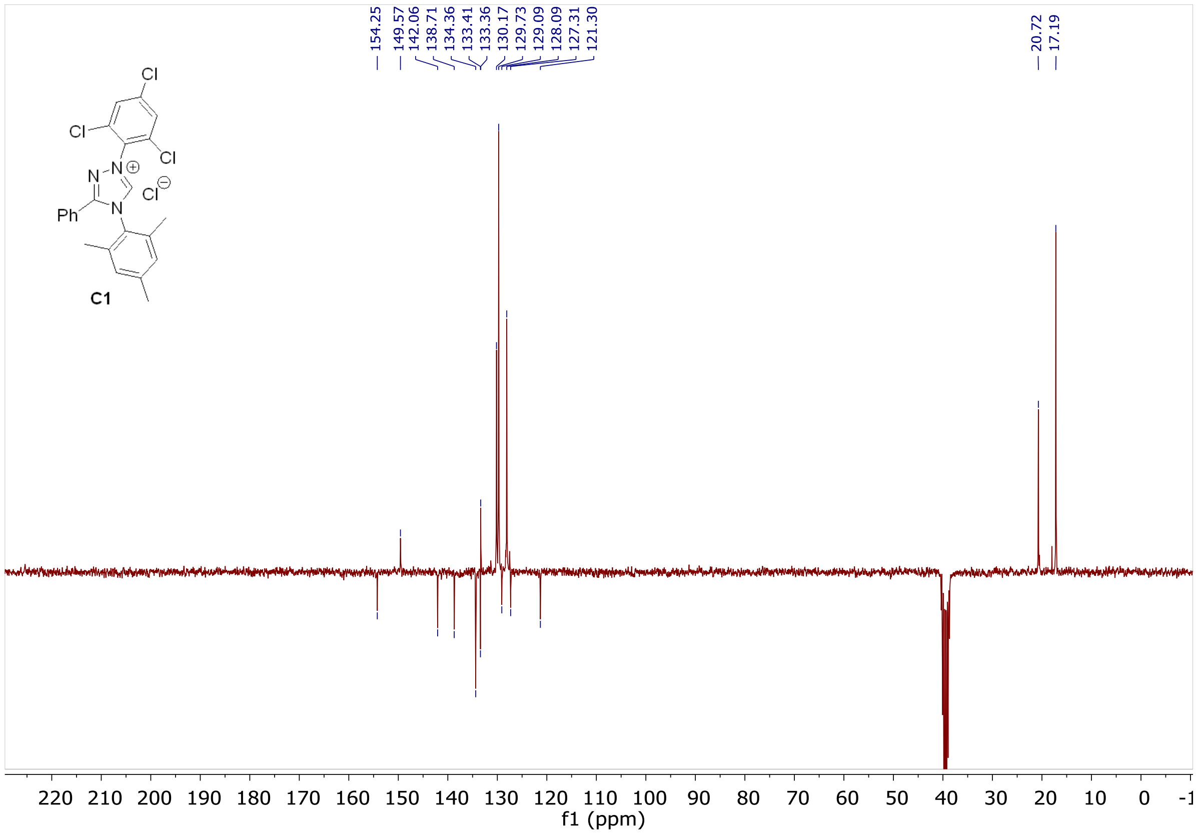

Figure S18. ^{13}C NMR (75 MHz, APT, DMSO-*d6*) of **C1**

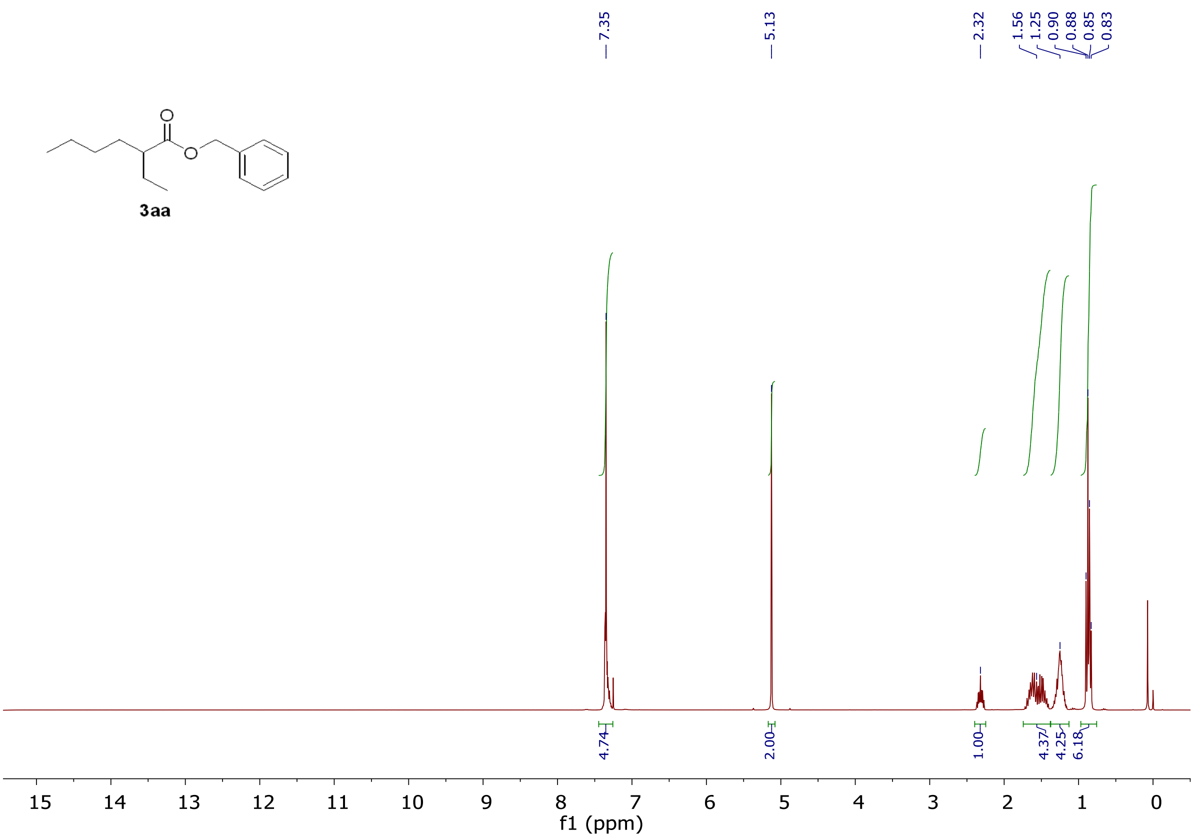

Figure S19. ^{1}H NMR (300 MHz, $CDCl_3$) of **3aa**

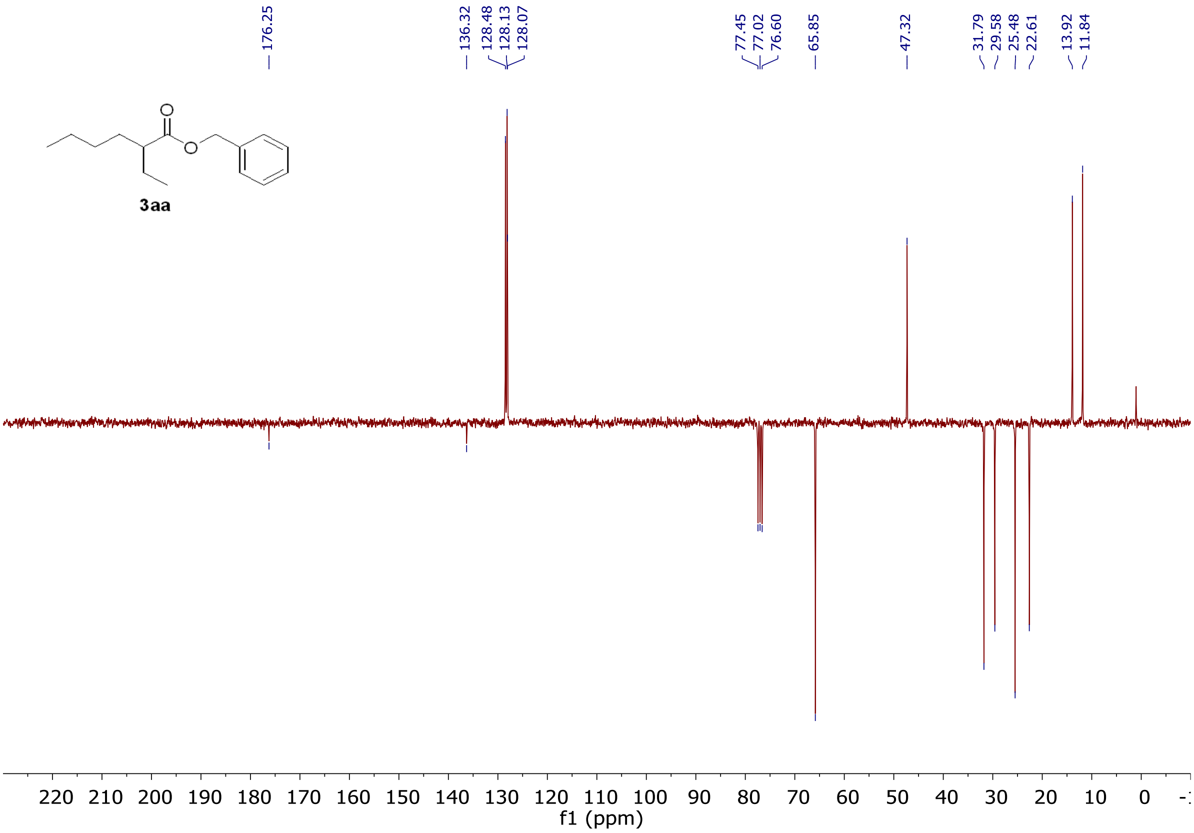

Figure S20. ^{13}C NMR (75 MHz, APT, $CDCl_3$) of **3aa**

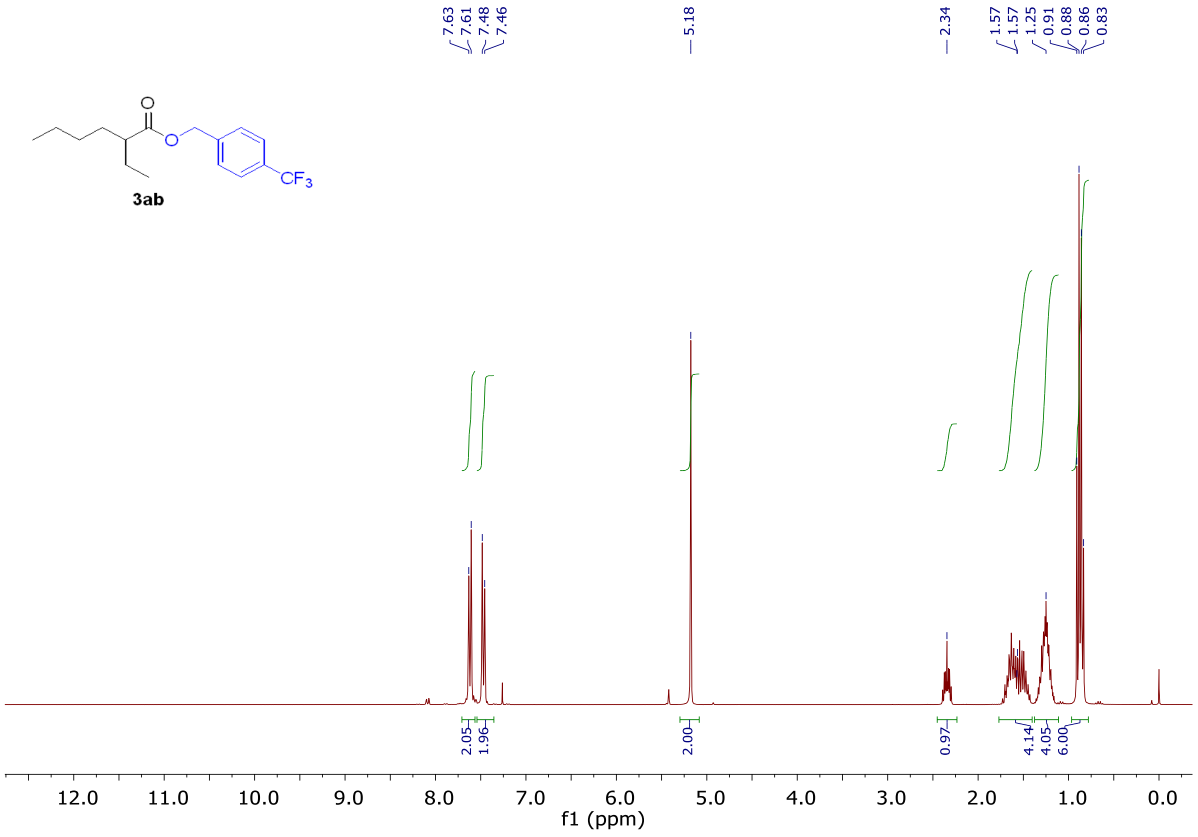

Figure S21. ^{1}H NMR (300 MHz, $CDCl_3$) of **3ab**

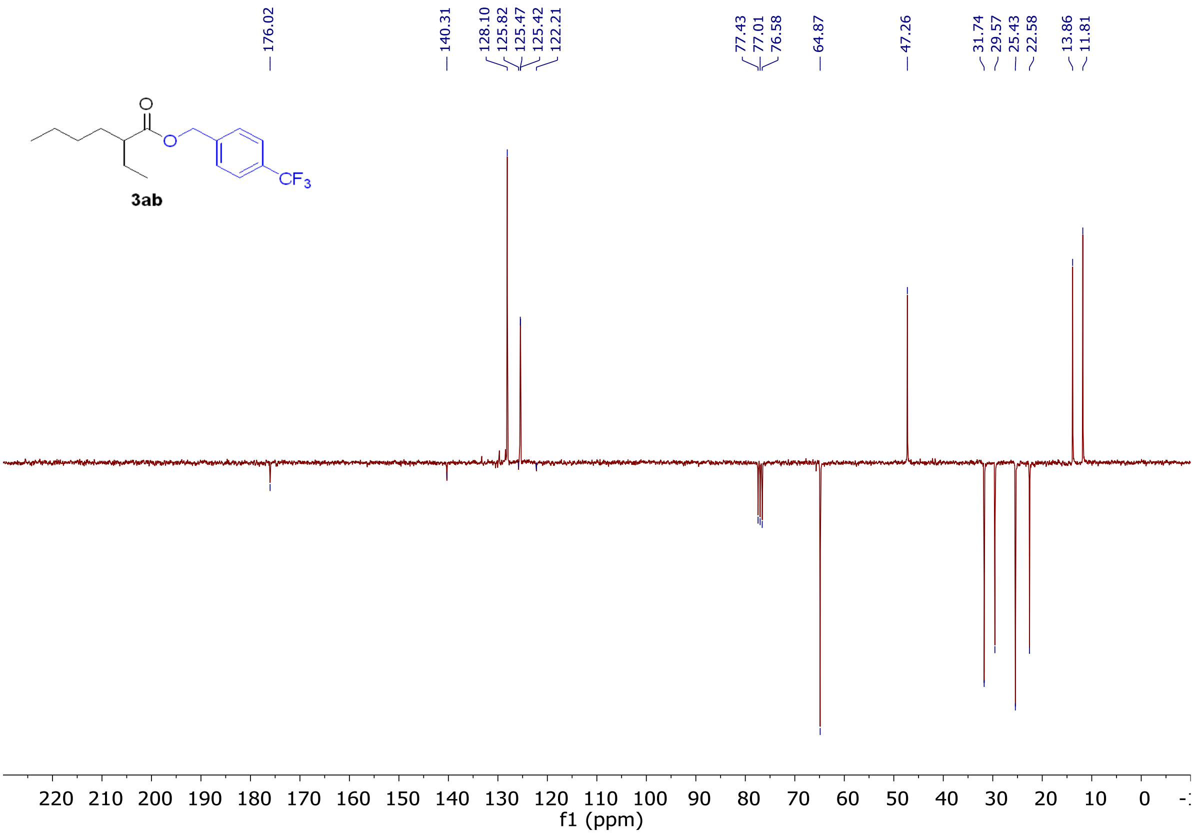

Figure S22. ^{13}C NMR (75 MHz, APT, $CDCl_3$) of **3ab**

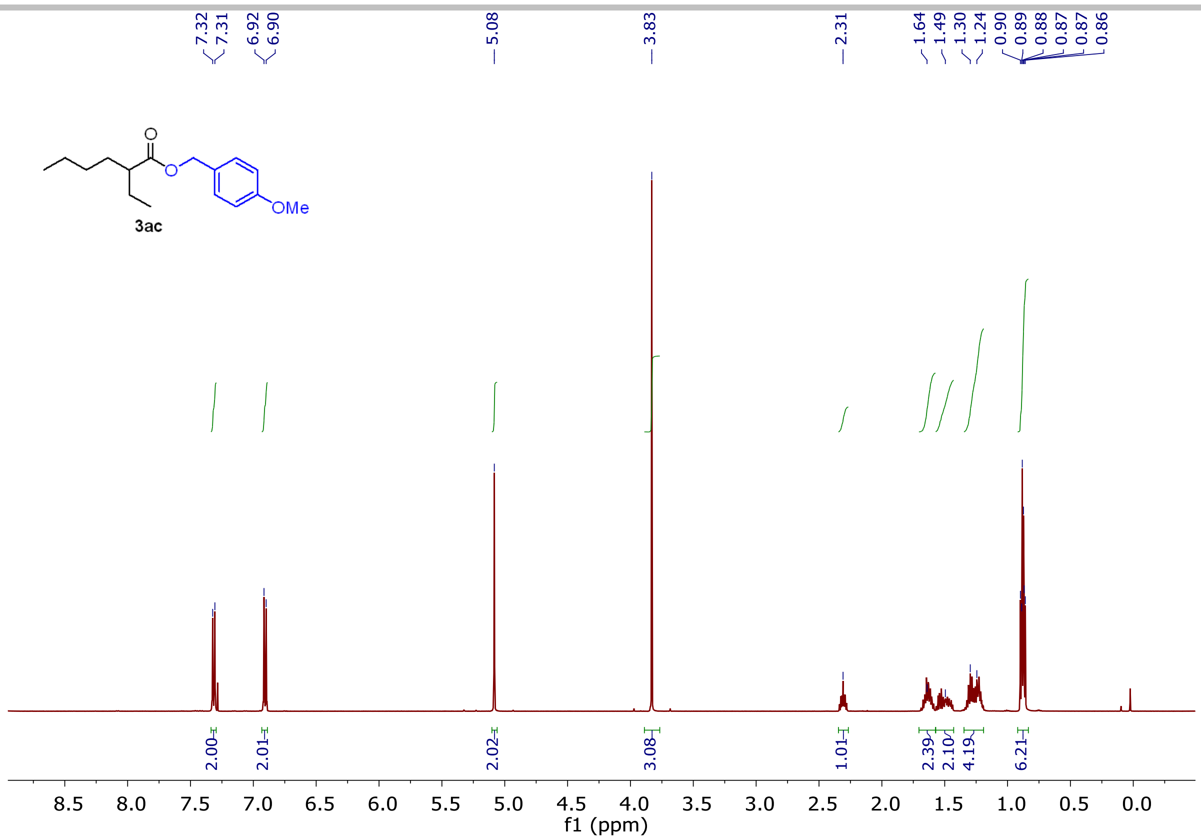

Figure S23. ^{1}H NMR (500 MHz, $CDCl_3$) of **3ac**

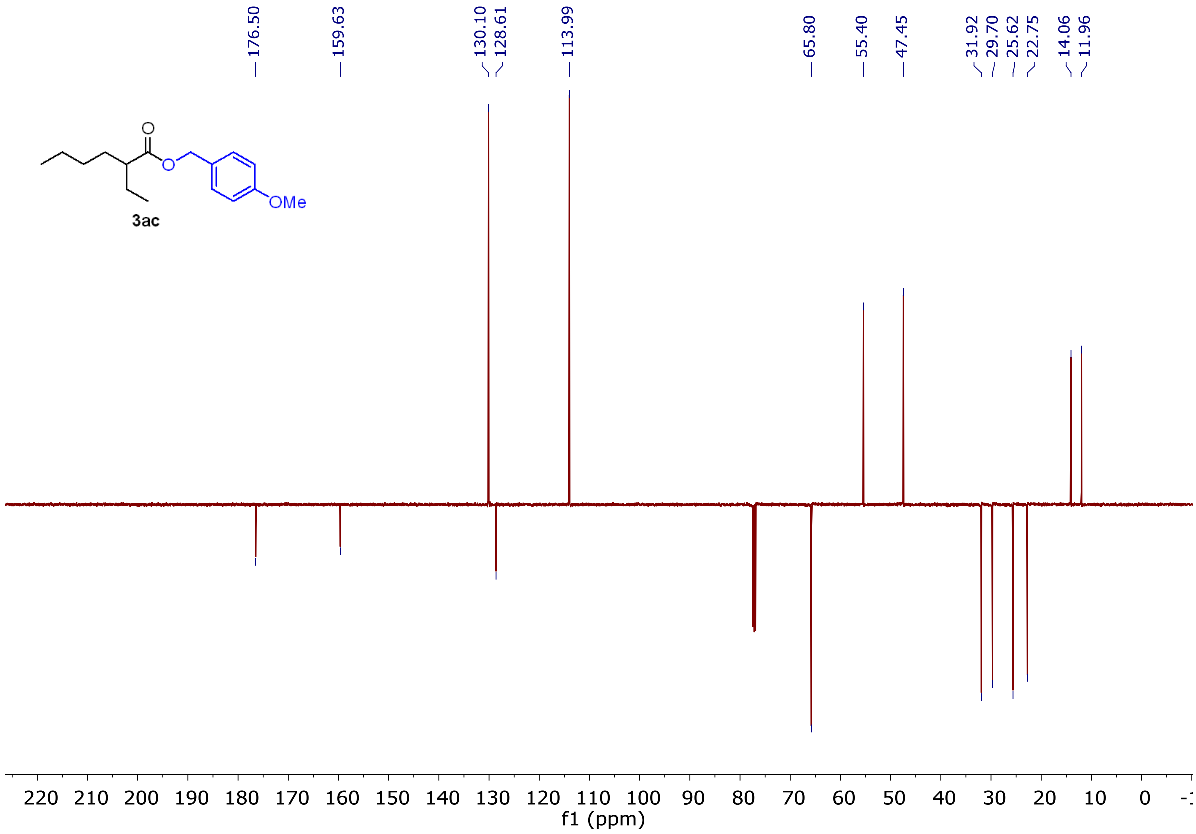

Figure S24. ^{13}C NMR (125 MHz, APT, $CDCl_3$) of **3ac**

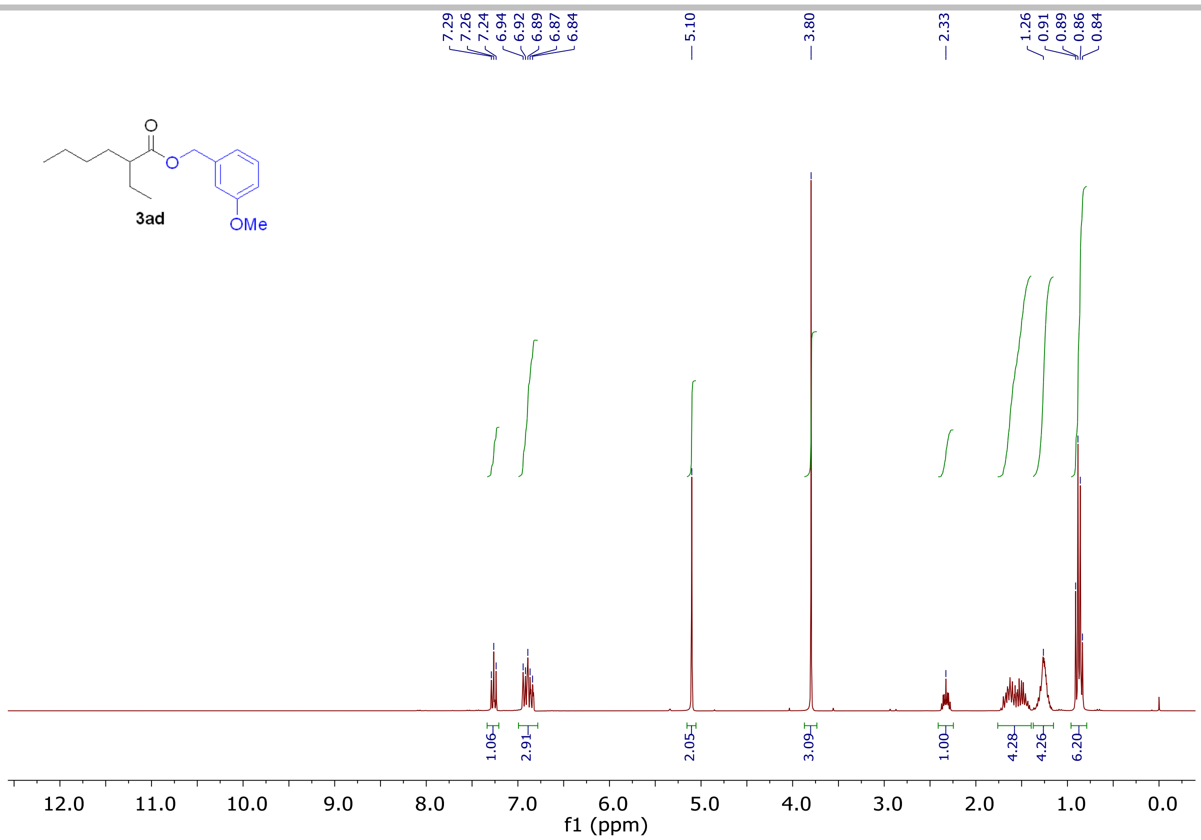

Figure S25. ^{1}H NMR (300 MHz, $CDCl_3$) of **3ad**

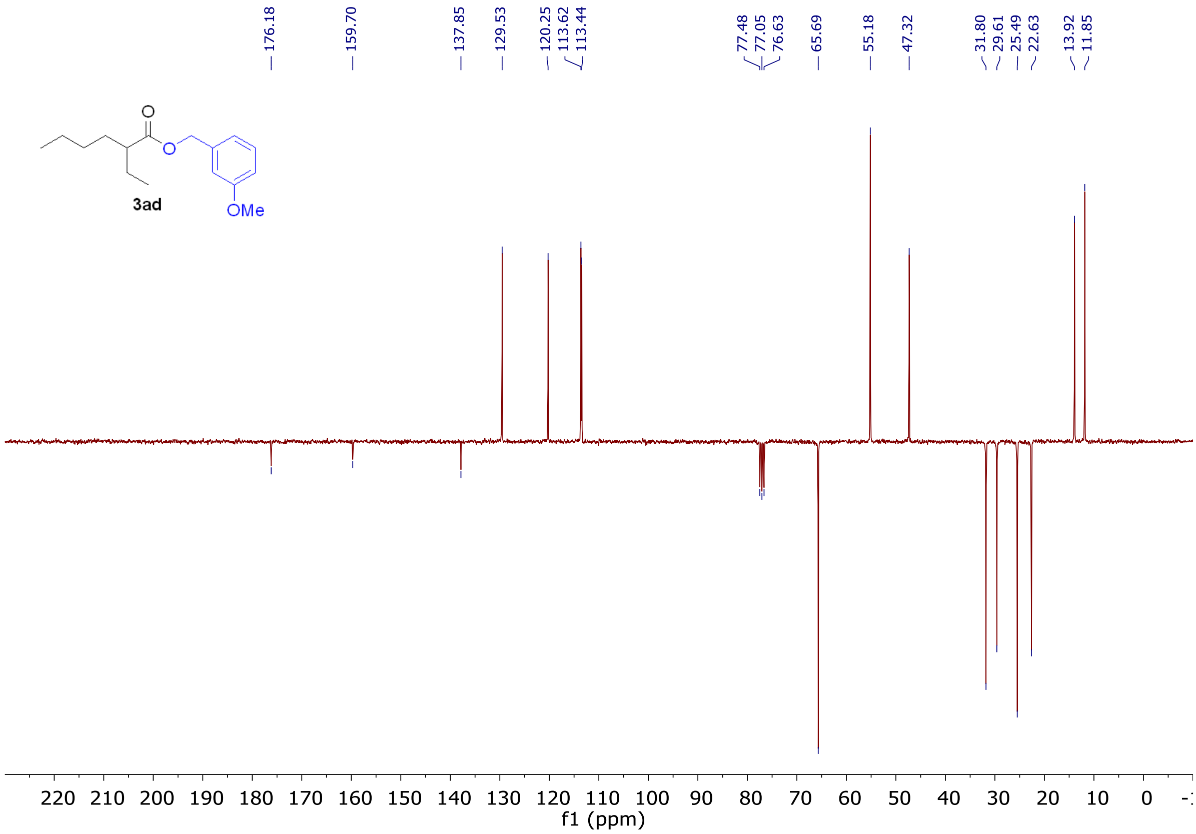

Figure S26. ^{13}C NMR (75 MHz, APT, $CDCl_3$) of **3ad**

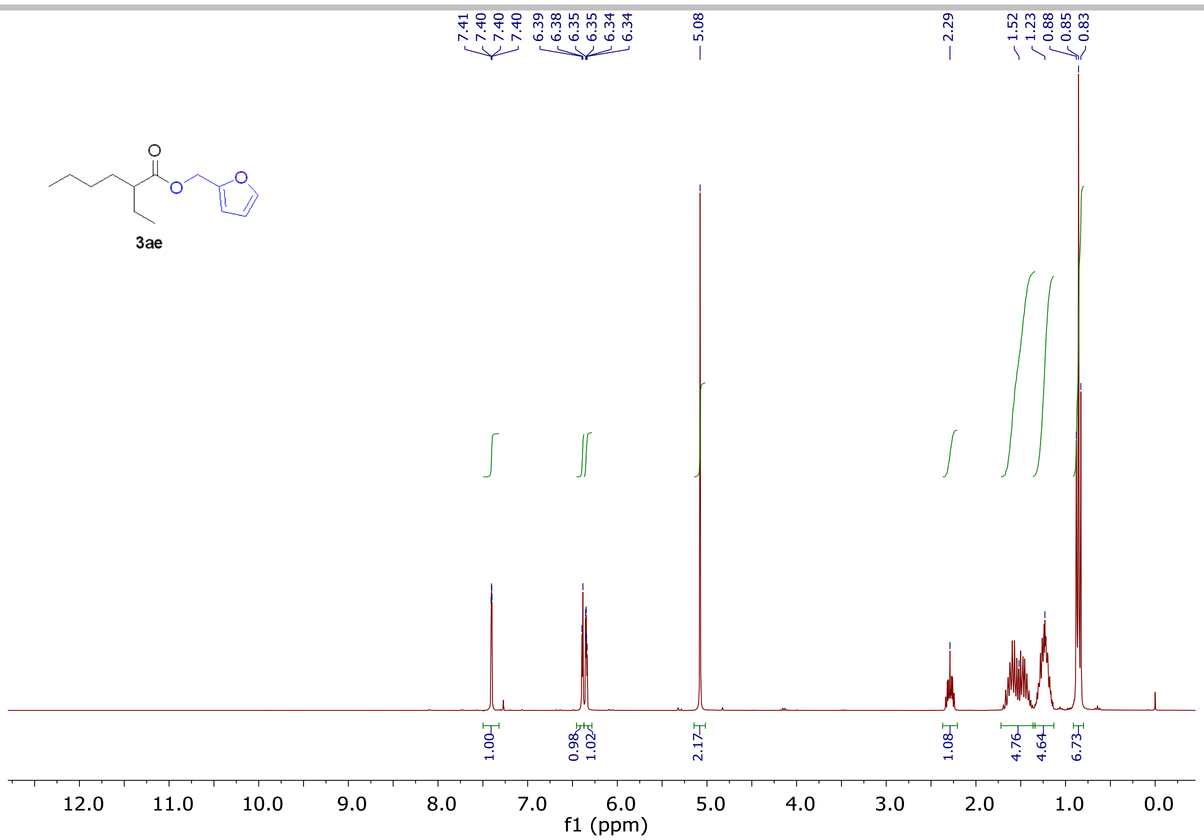

Figure S27. ^{1}H NMR (300 MHz, $CDCl_3$) of **3ae**

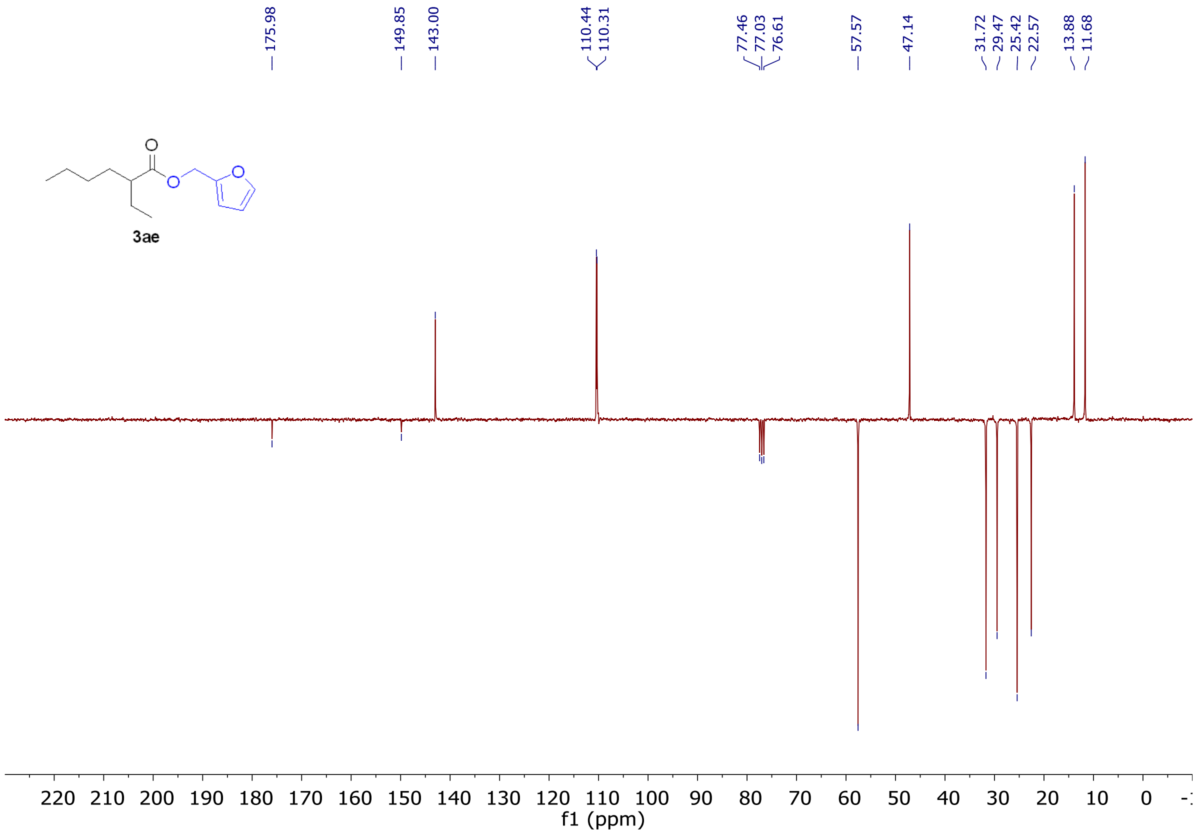

Figure S28. ^{13}C NMR (75 MHz, APT, $CDCl_3$) of **3ae**

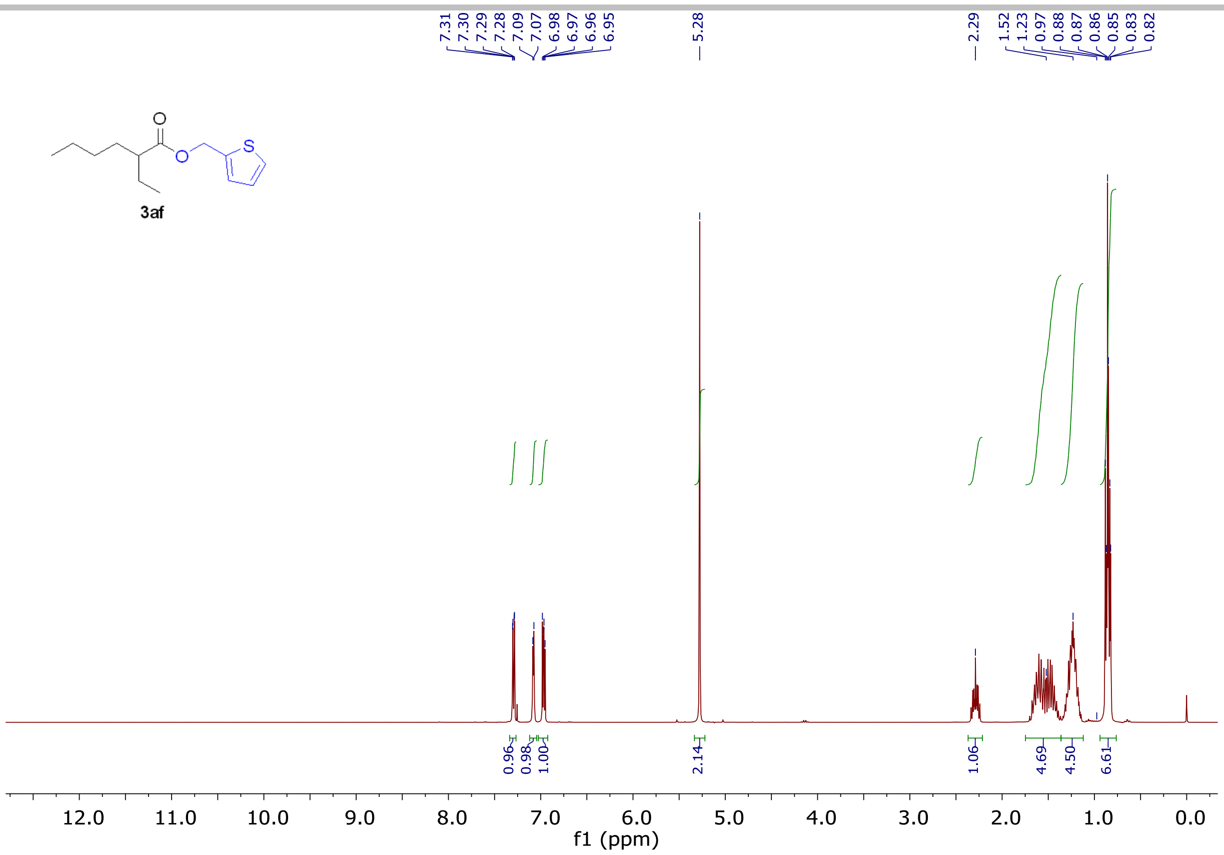

Figure S29. ^{1}H NMR (300 MHz, $CDCl_3$) of **3af**

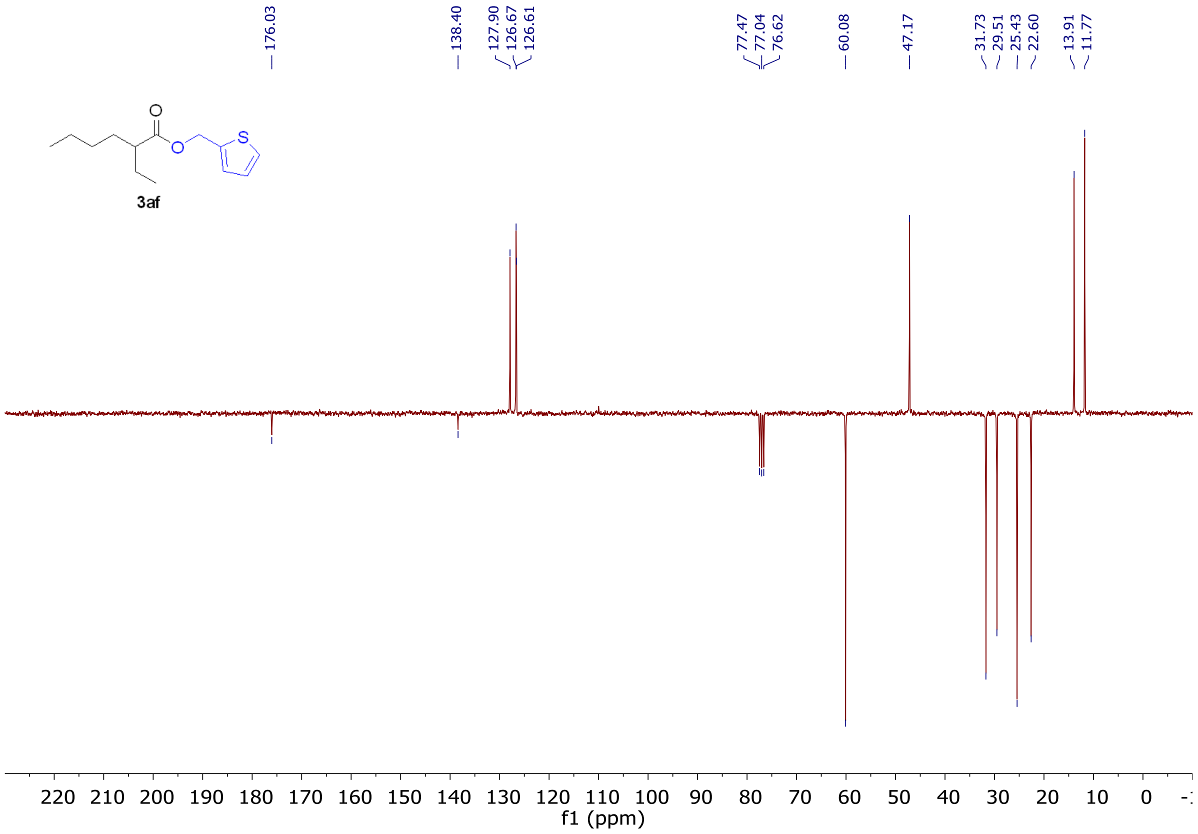

Figure S30. ^{13}C NMR (75 MHz, APT, $CDCl_3$) of **3af**

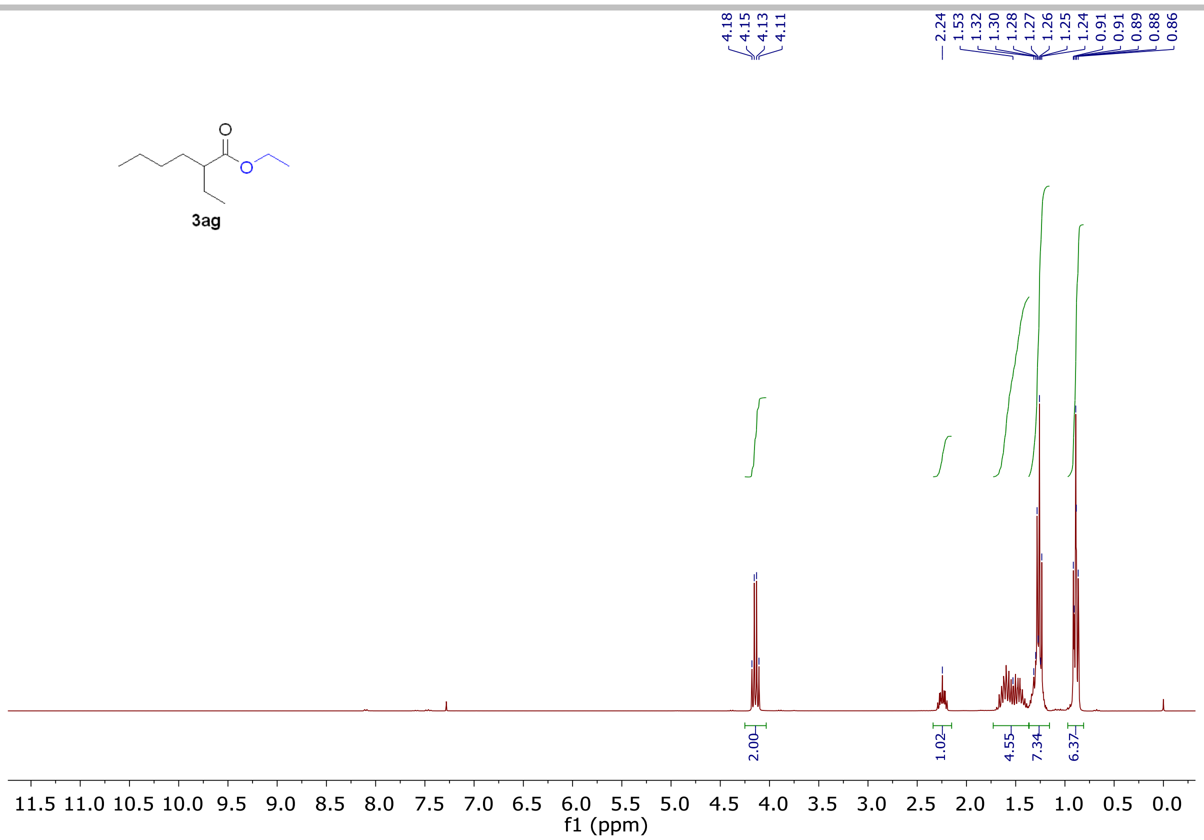

Figure S31. ^{1}H NMR (300 MHz, $CDCl_3$) of **3ag**

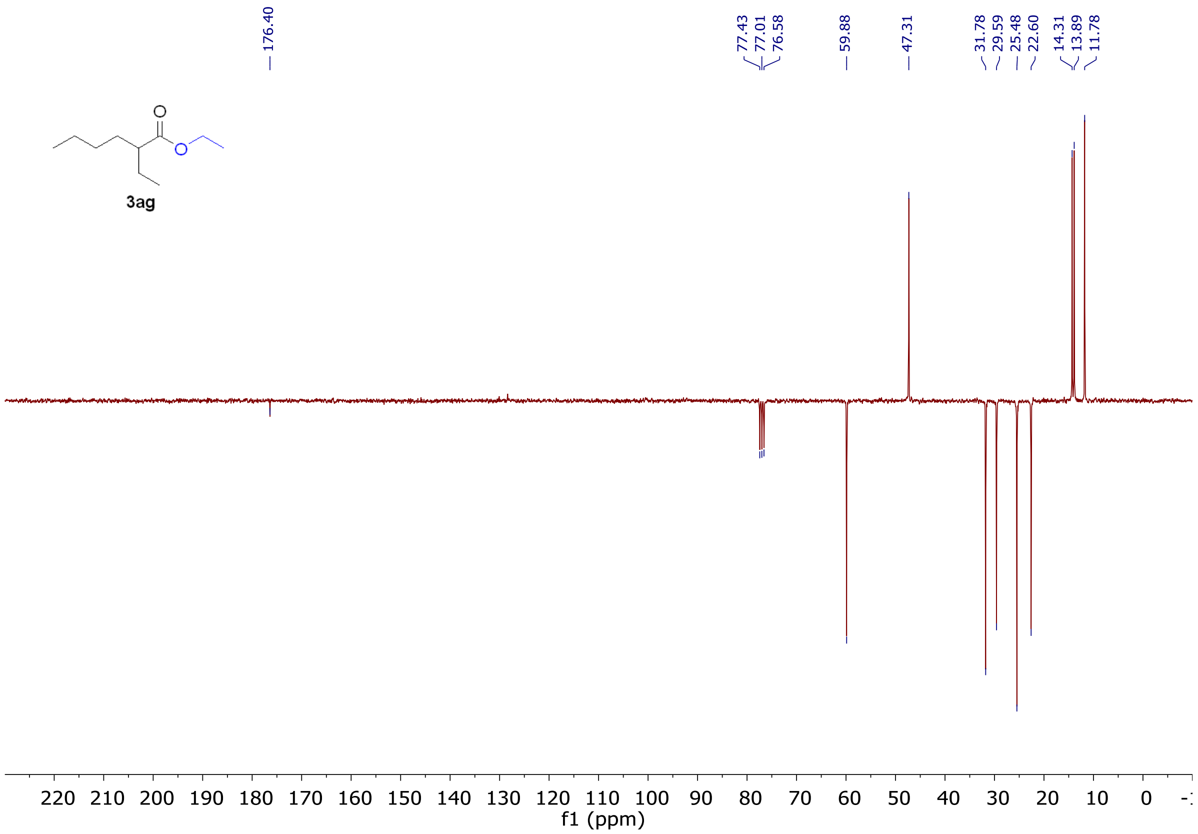

Figure S32. ^{13}C NMR (75 MHz, APT, $CDCl_3$) of **3ag**

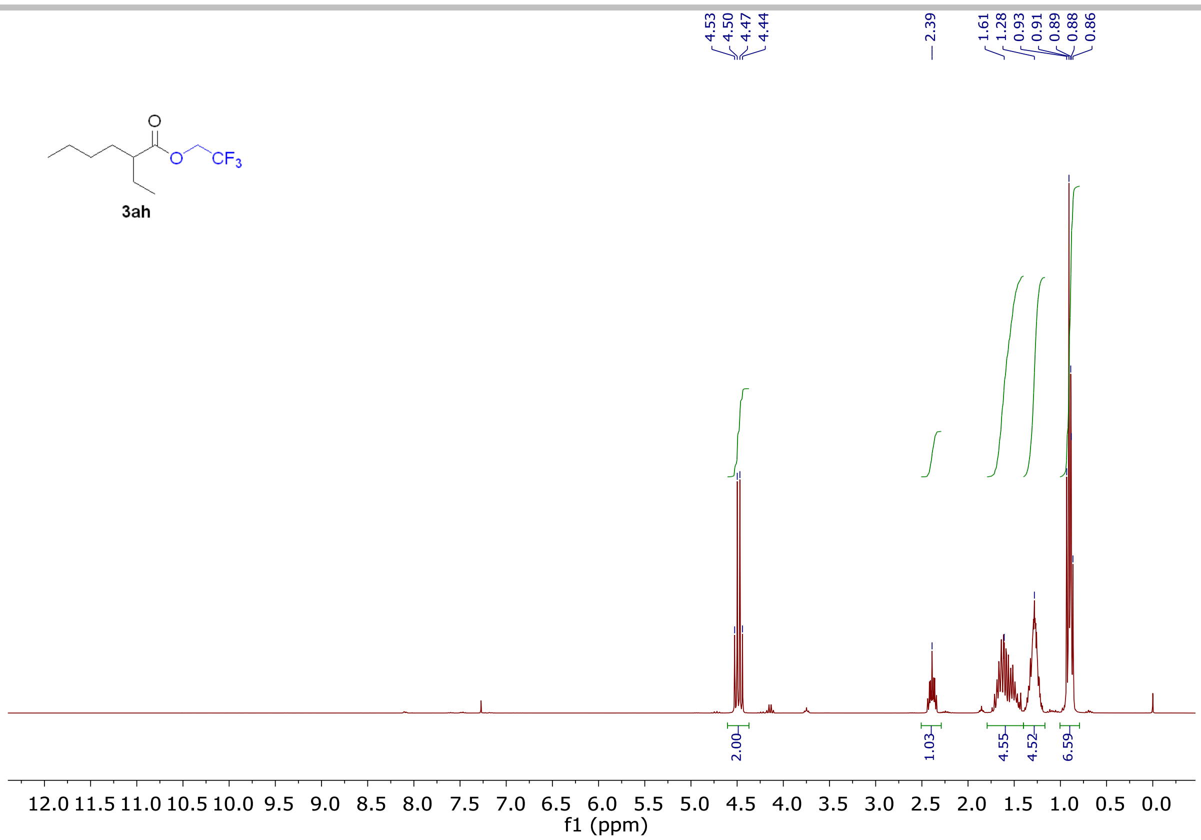

Figure S33. ^{1}H NMR (300 MHz, $CDCl_3$) of **3ah**

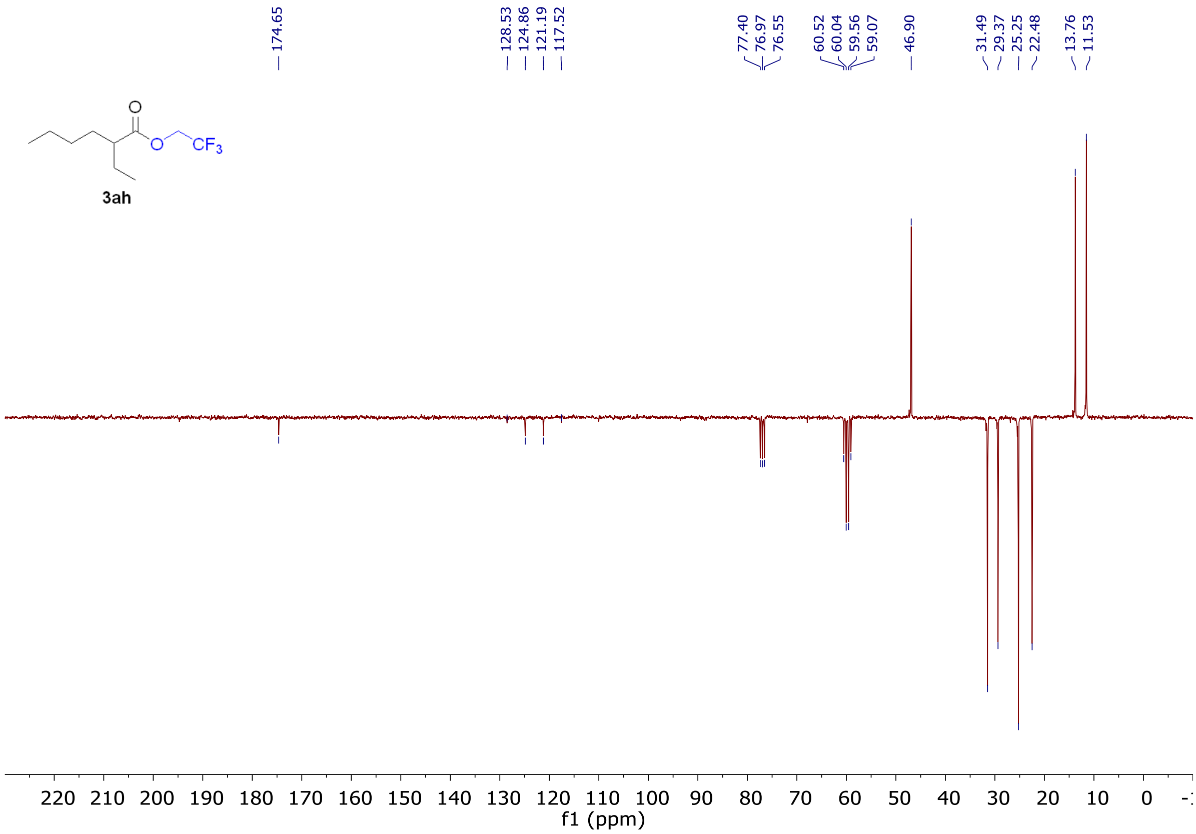

Figure S34. ^{13}C NMR (75 MHz, APT, $CDCl_3$) of **3ah**

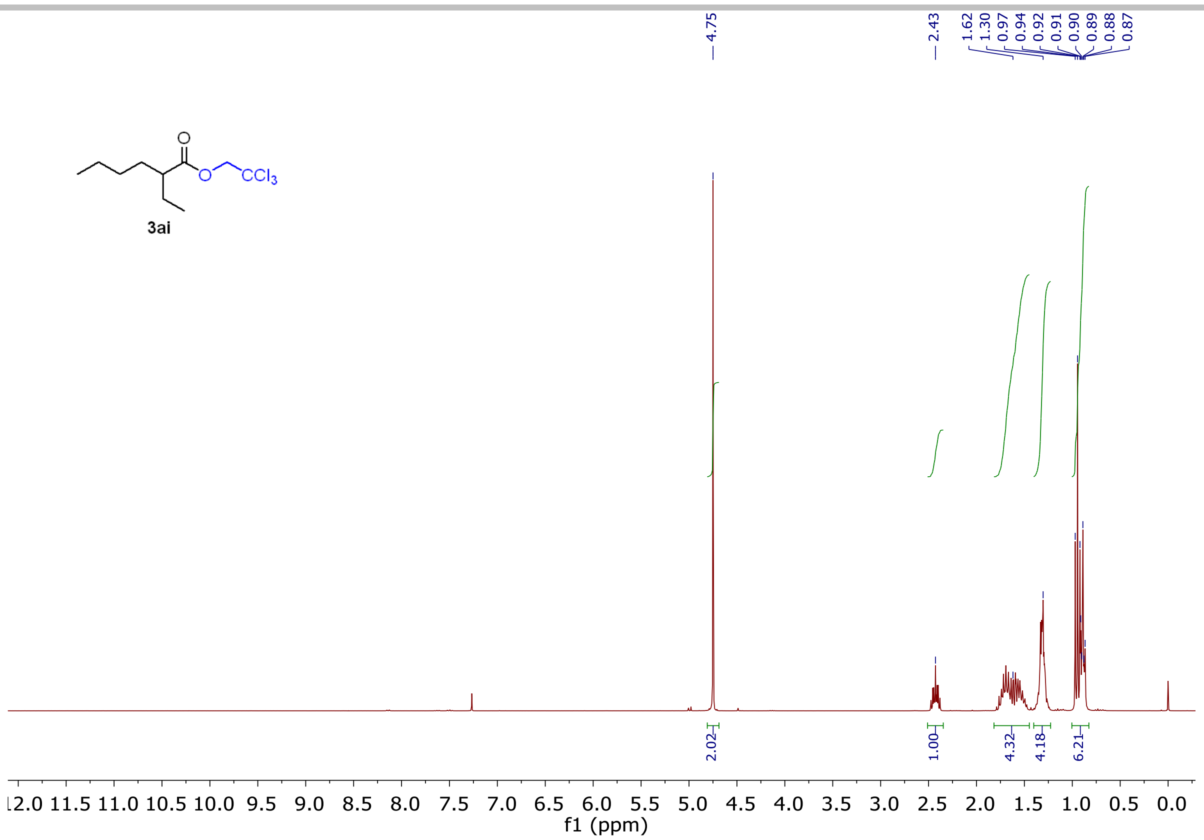

Figure S35. ^{1}H NMR (300 MHz, $CDCl_3$) of **3ai**

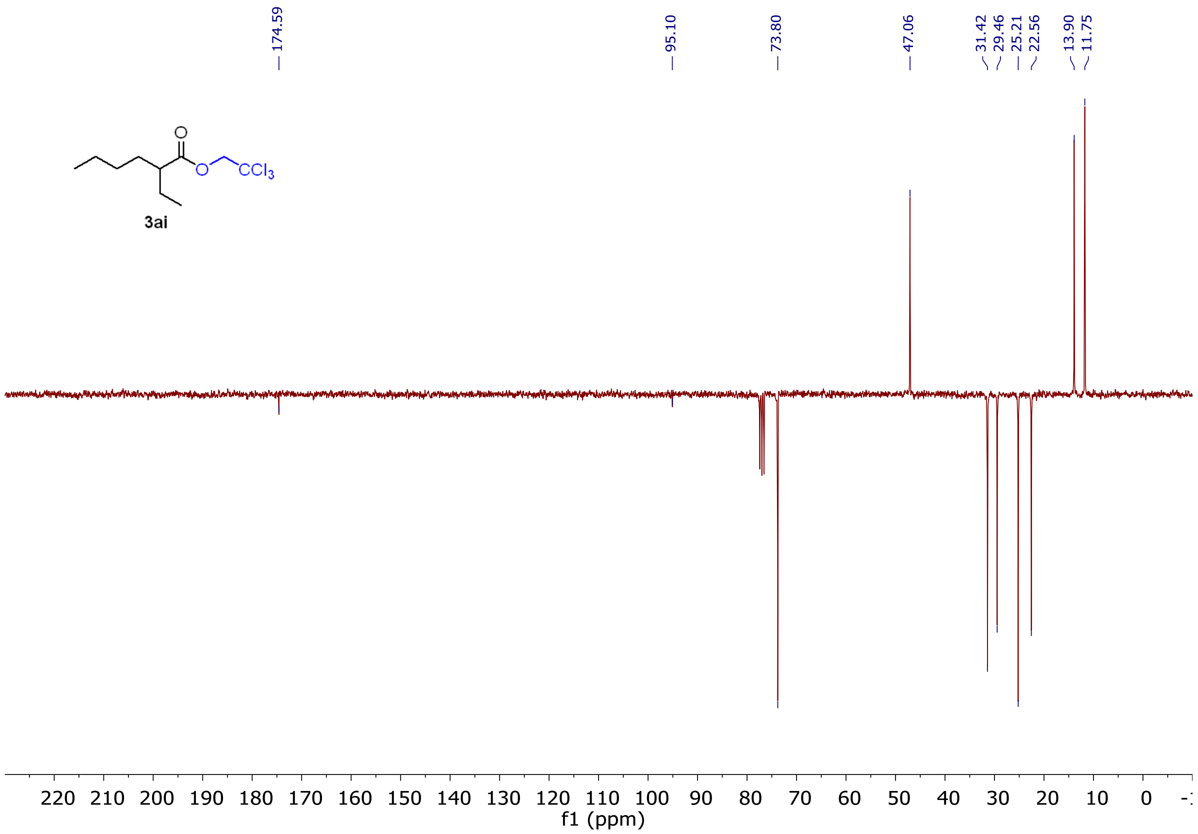

Figure S36. ^{13}C NMR (75 MHz, APT, $CDCl_3$) of **3ai**

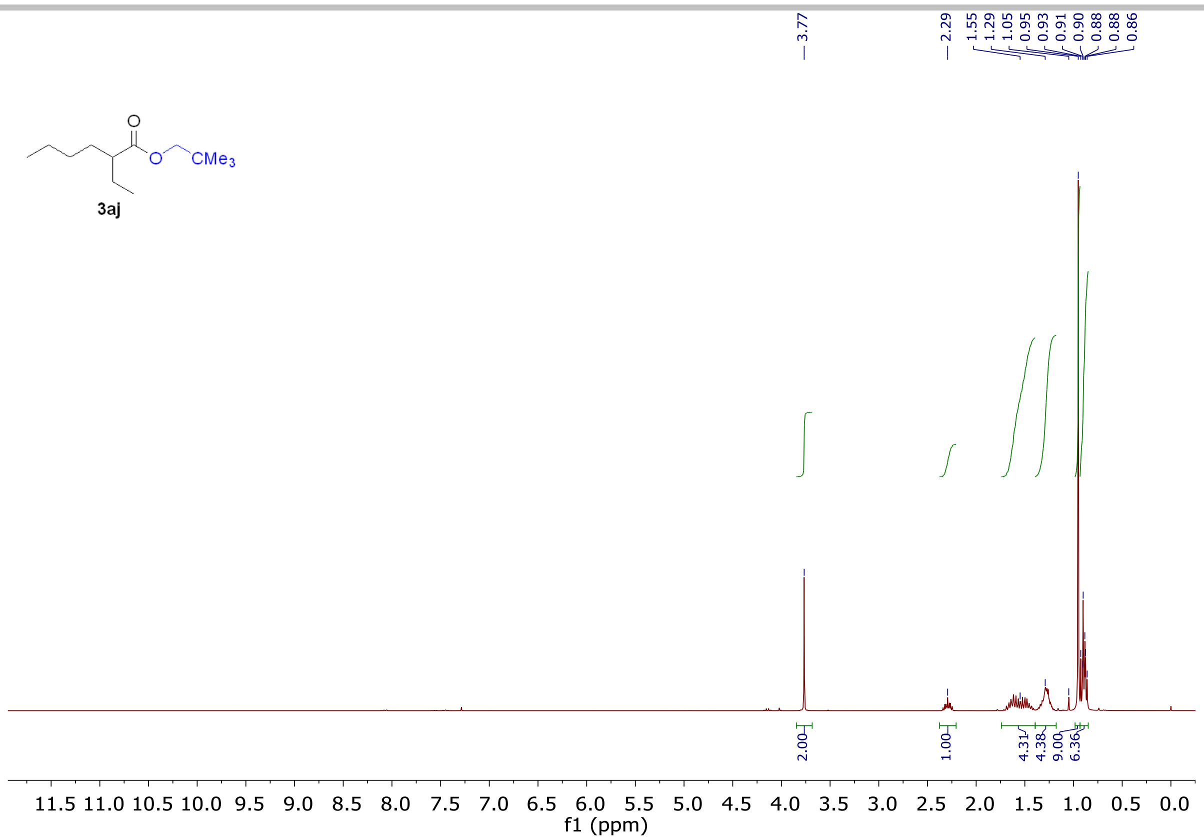

Figure S37. ^{1}H NMR (300 MHz, $CDCl_3$) of **3aj**

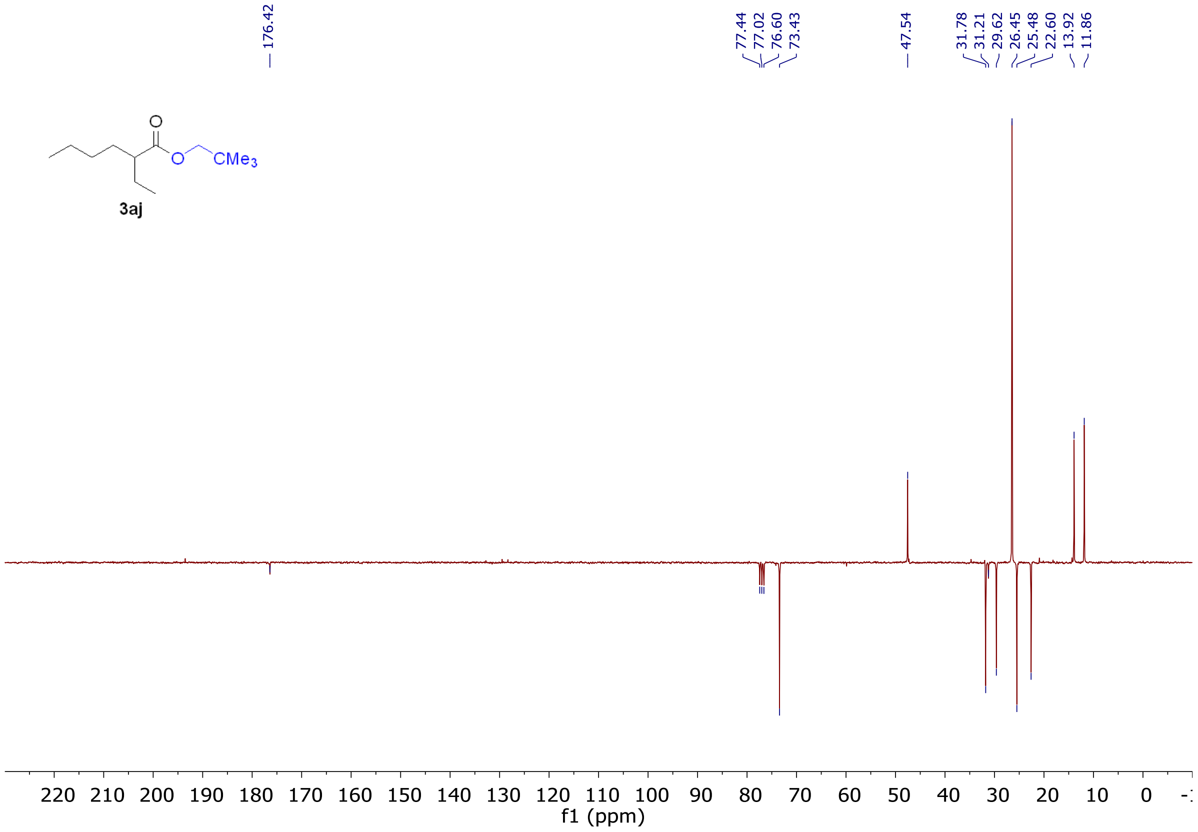

Figure S38. ^{13}C NMR (75 MHz, APT, $CDCl_3$) of **3aj**

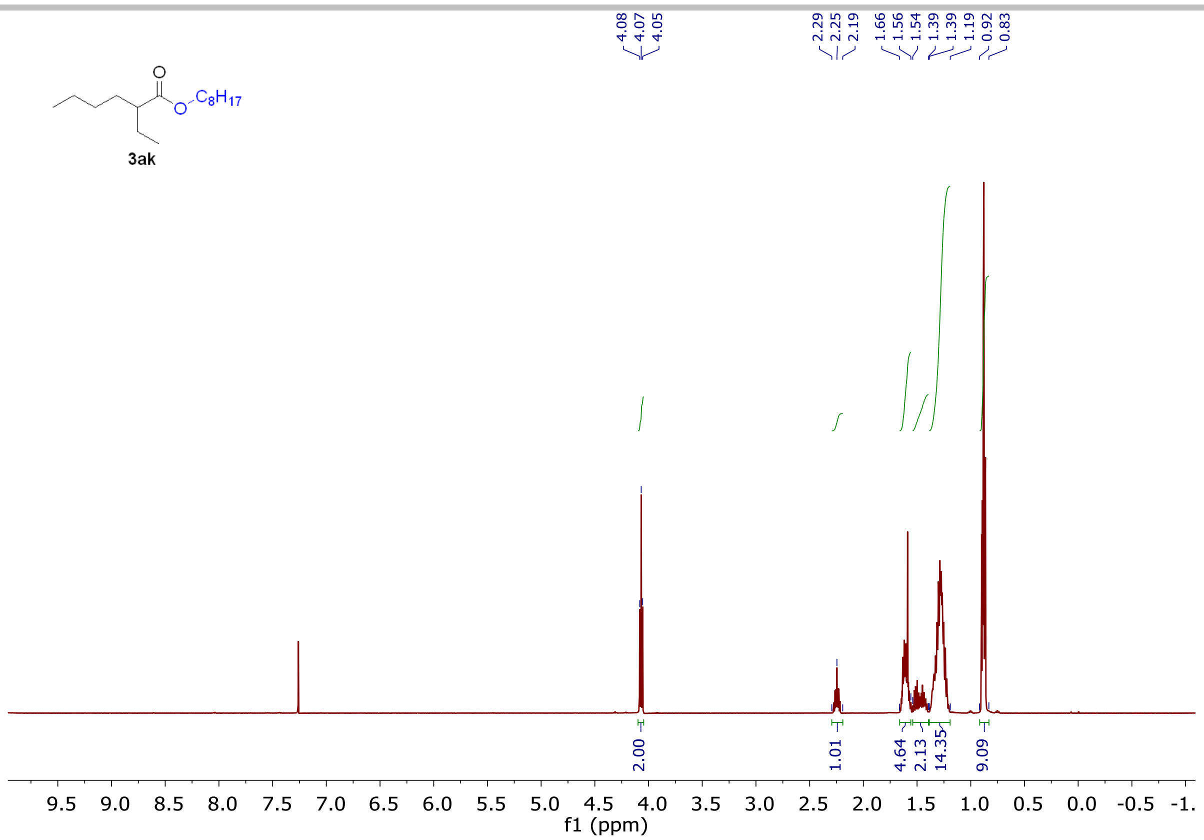

Figure S39. ^{1}H NMR (500 MHz, $CDCl_3$) of **3ak**

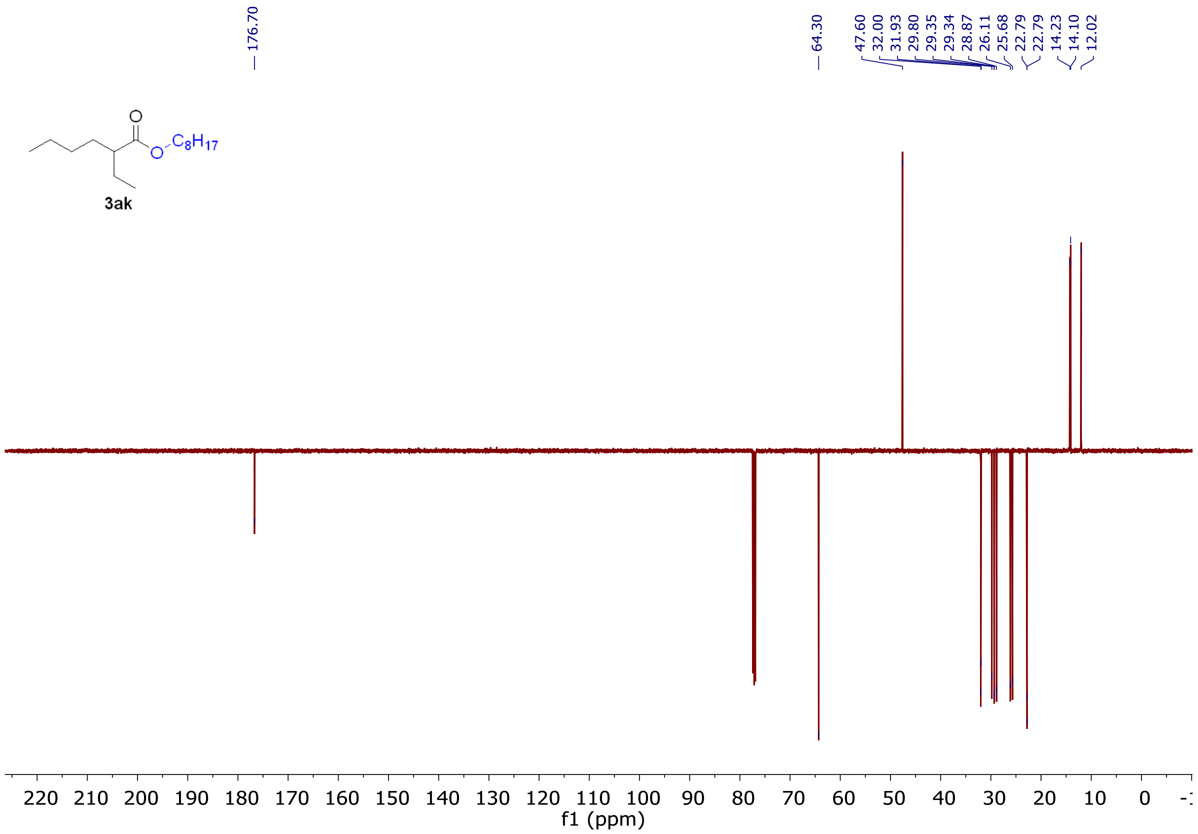

Figure S40. ^{13}C NMR (126 MHz, APT, $CDCl_3$) of **3ak**

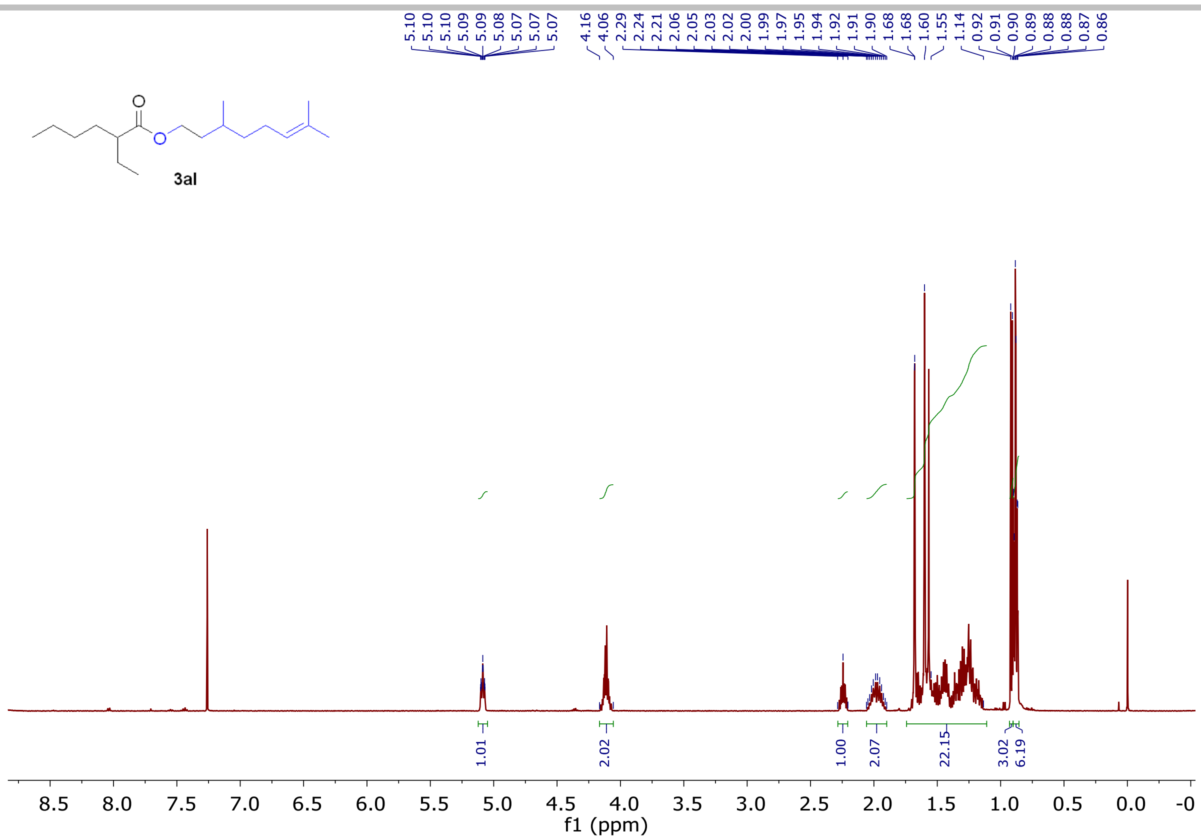

Figure S41. ^{1}H NMR (300 MHz, $CDCl_3$) of **3al**

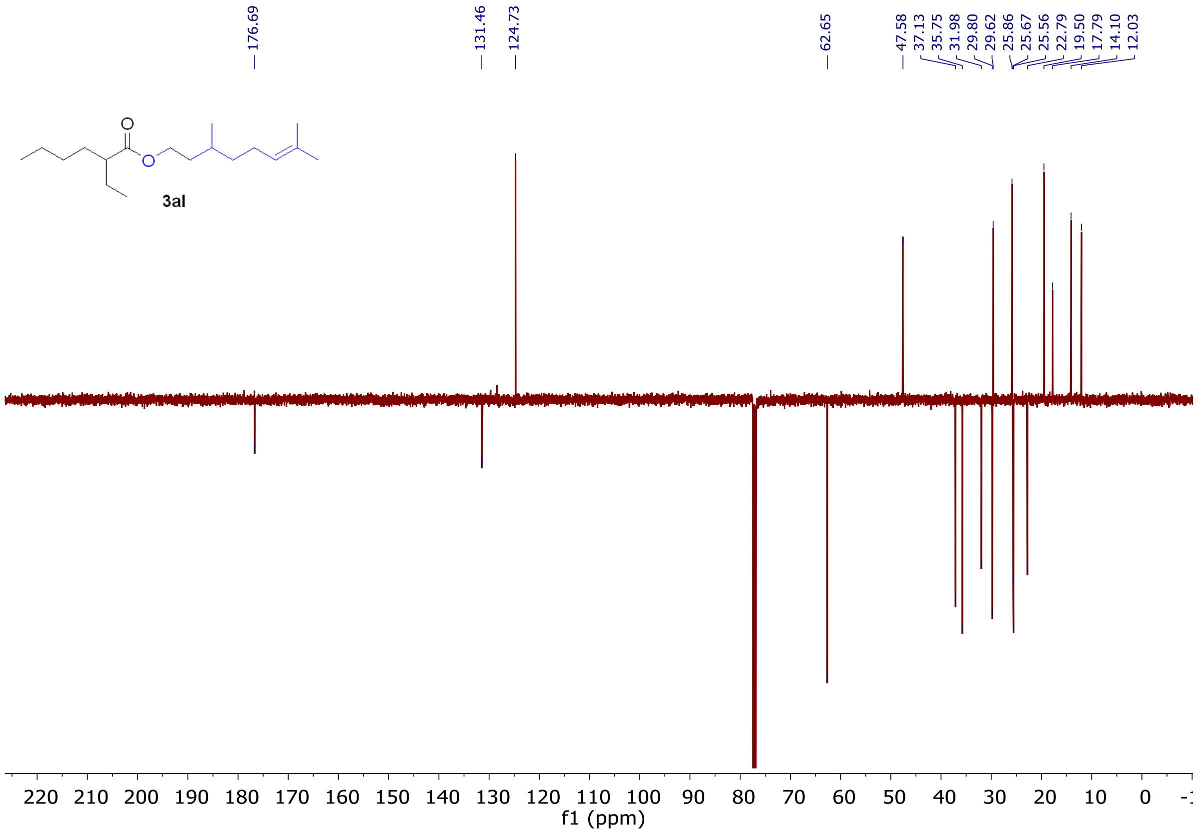

Figure S42. ^{13}C NMR (75 MHz, APT, $CDCl_3$) of **3al**

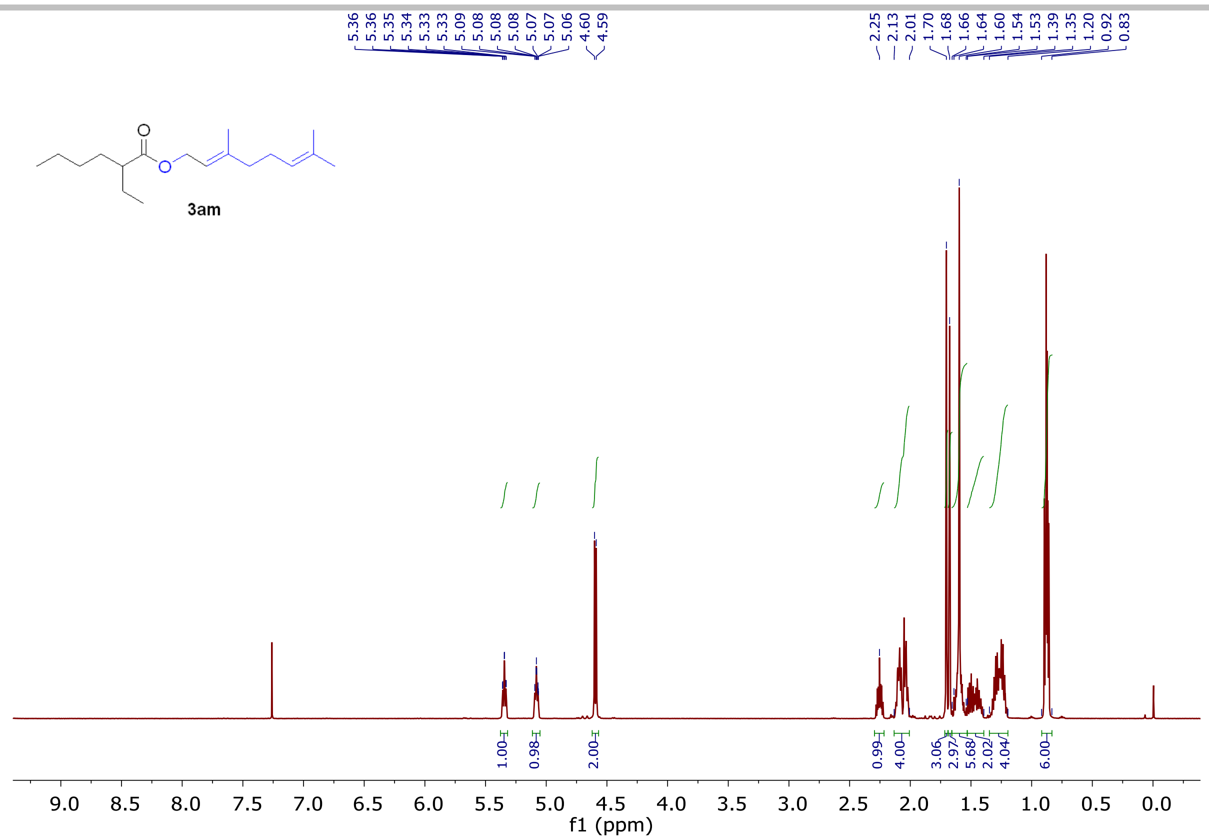

Figure S43. ^{1}H NMR (300 MHz, $CDCl_3$) of **3am**

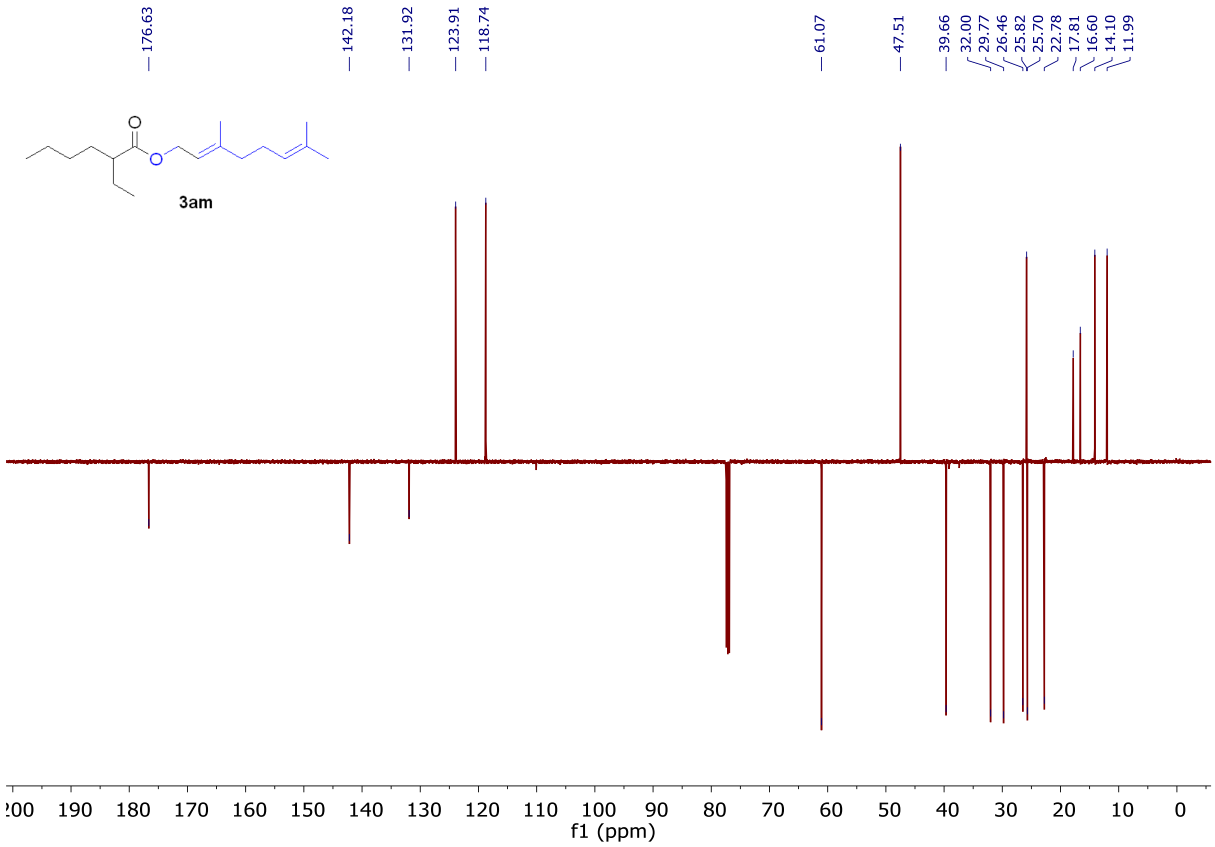

Figure S44. ^{13}C NMR (75 MHz, APT, $CDCl_3$) of **3am**

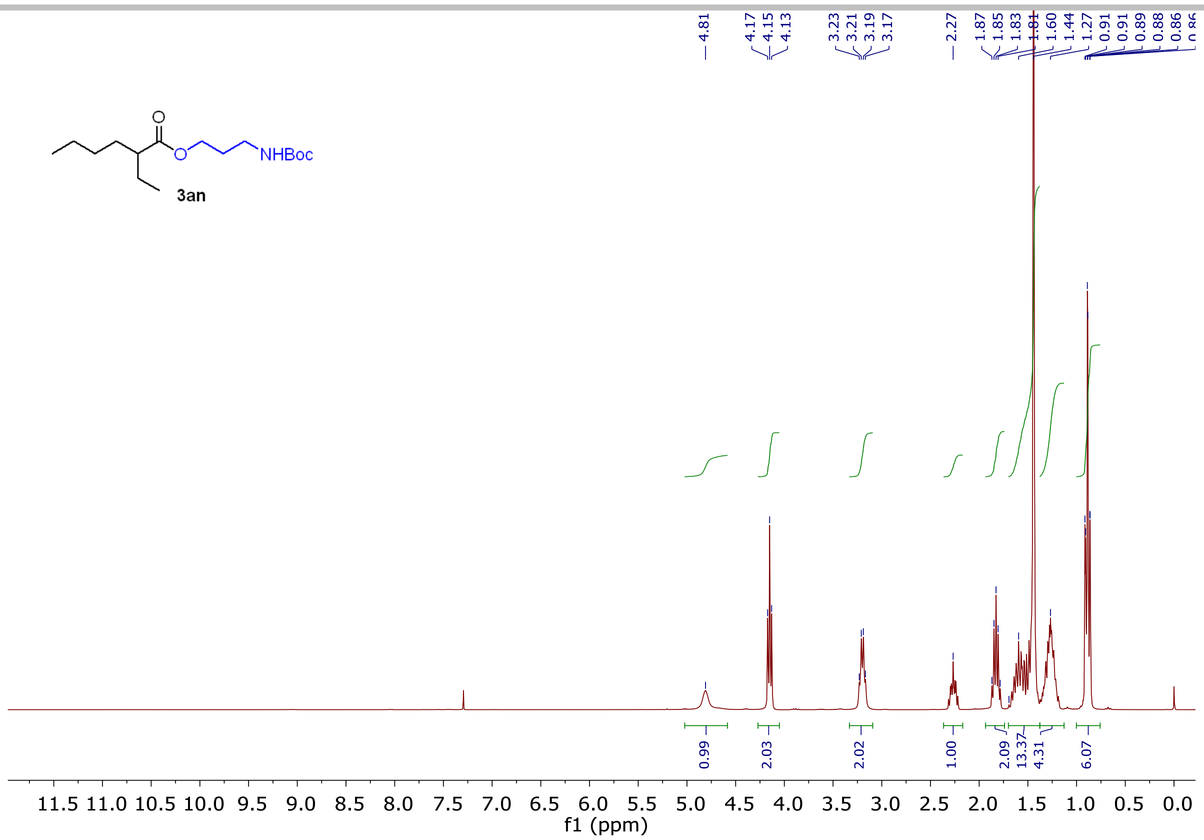

Figure S45. ^{1}H NMR (300 MHz, $CDCl_3$) of **3an**

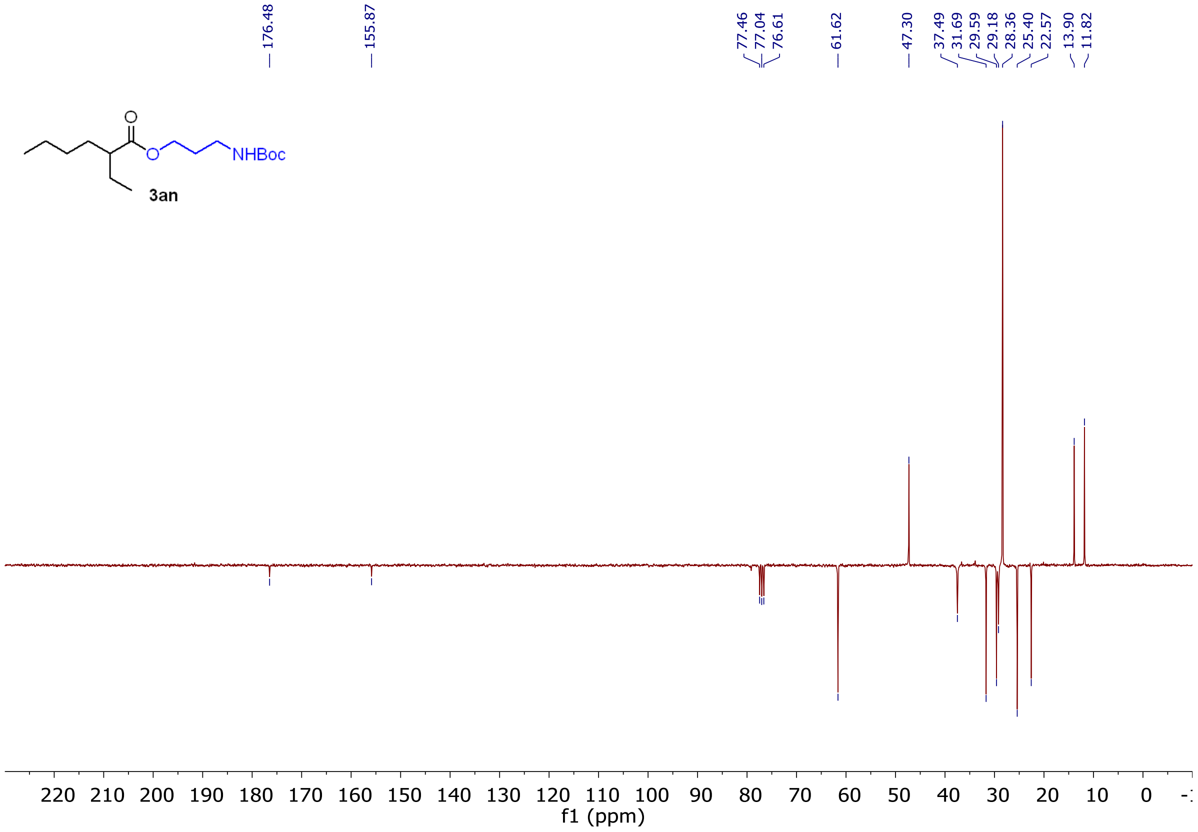

Figure S46. ^{13}C NMR (75 MHz, APT, $CDCl_3$) of **3an**

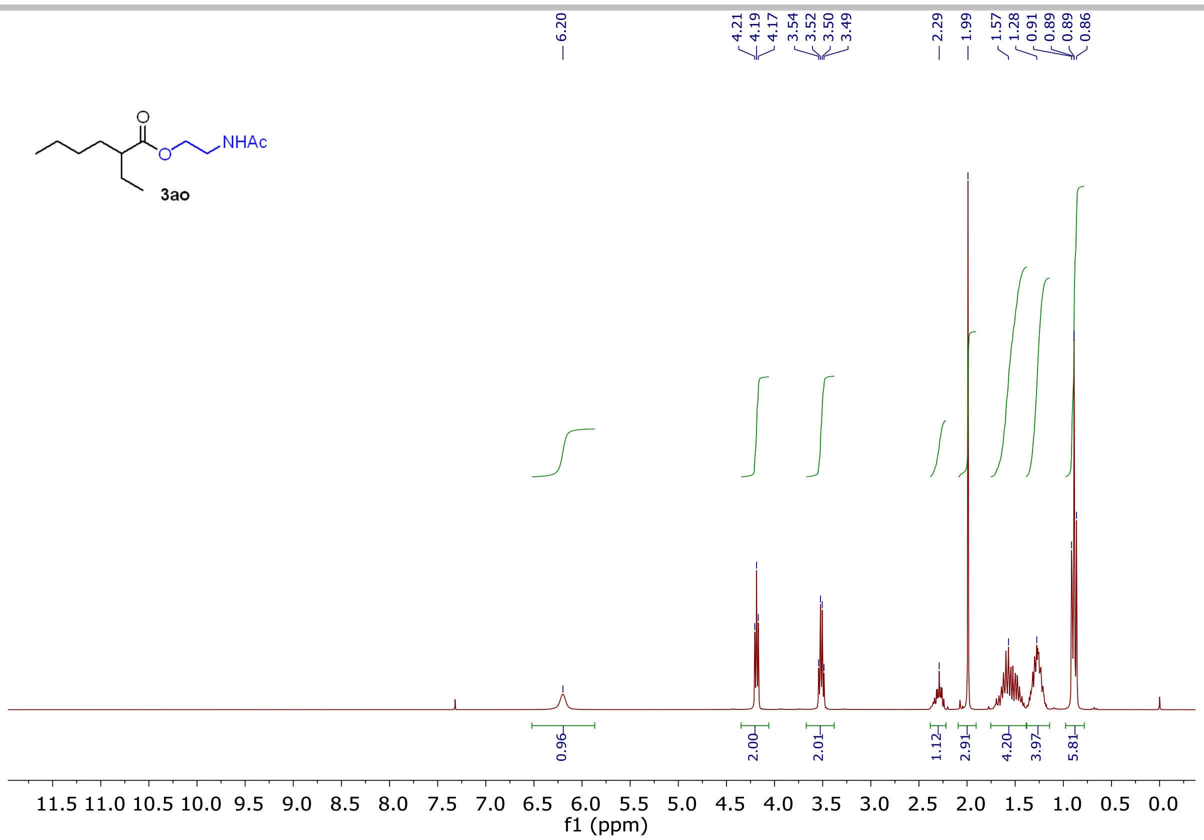

Figure S47. ^{1}H NMR (300 MHz, $CDCl_3$) of **3ao**

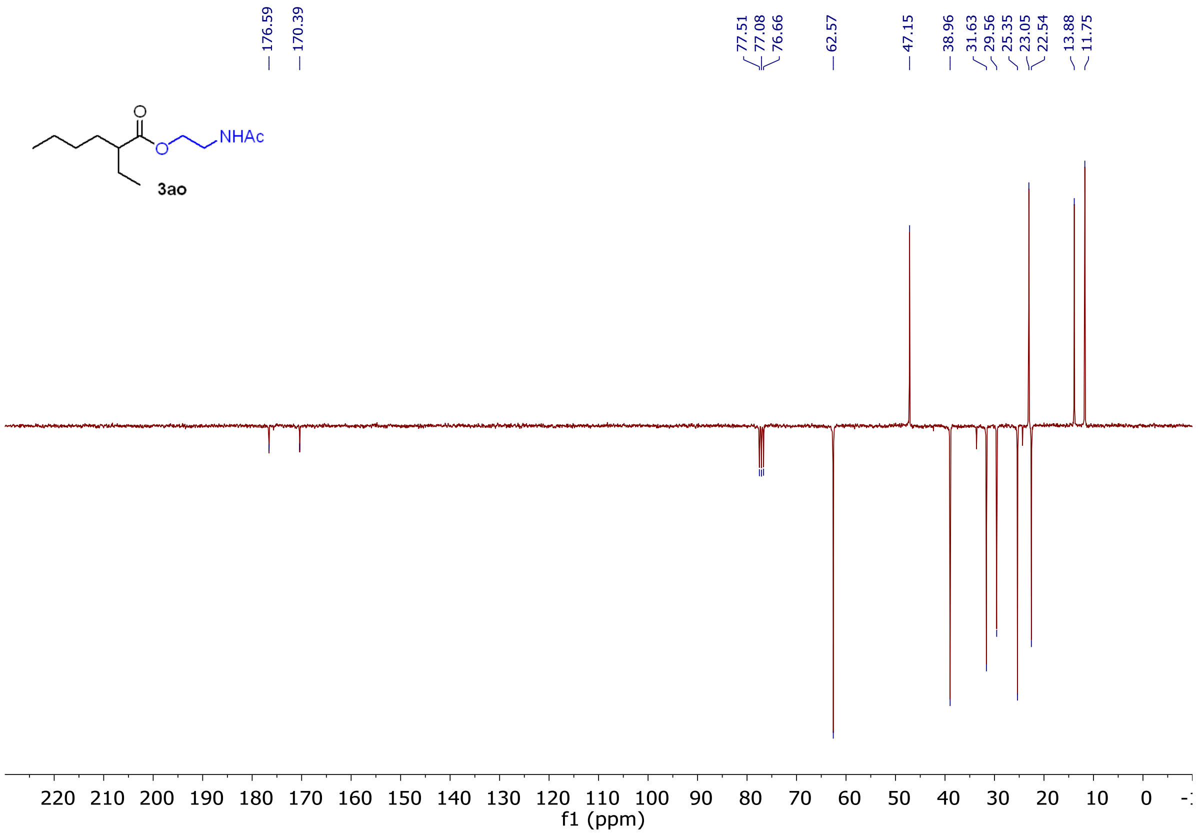

Figure S48. ^{13}C NMR (75 MHz, APT, $CDCl_3$) of **3ao**

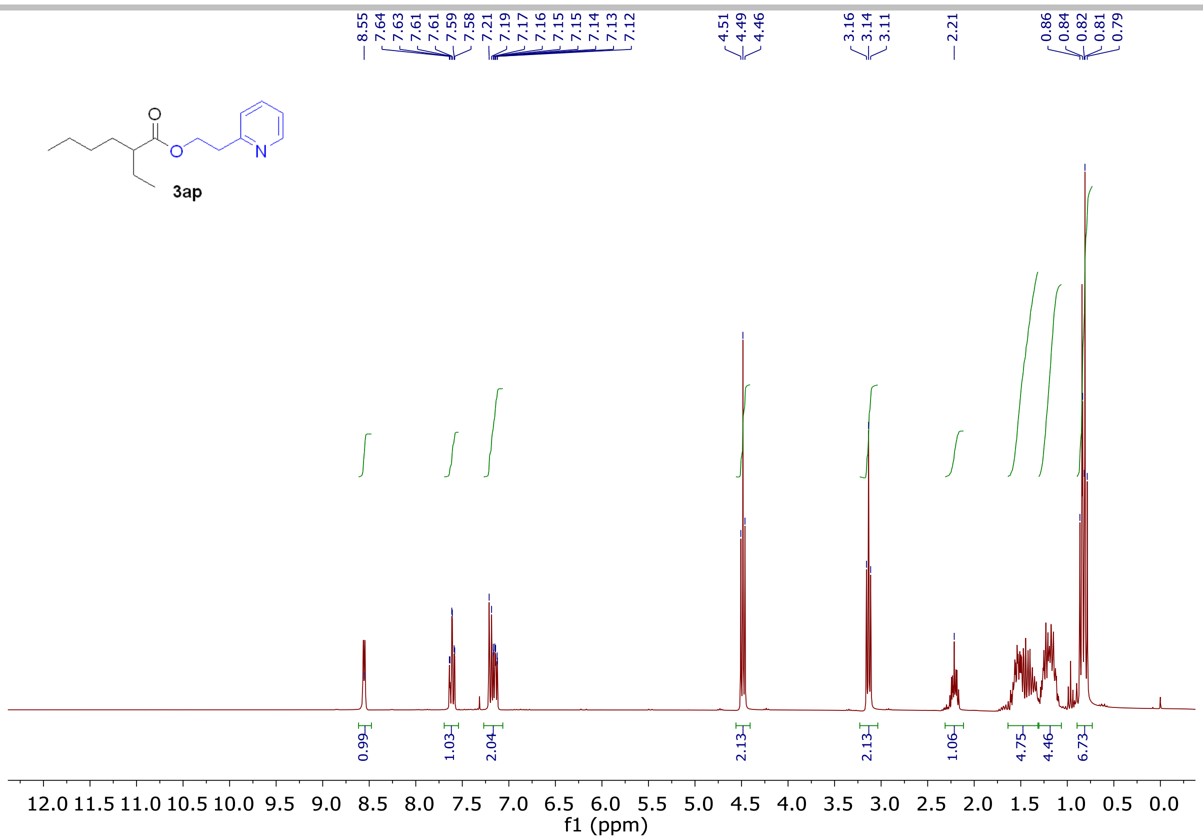

Figure S49. ^{1}H NMR (300 MHz, $CDCl_3$) of **3ap**

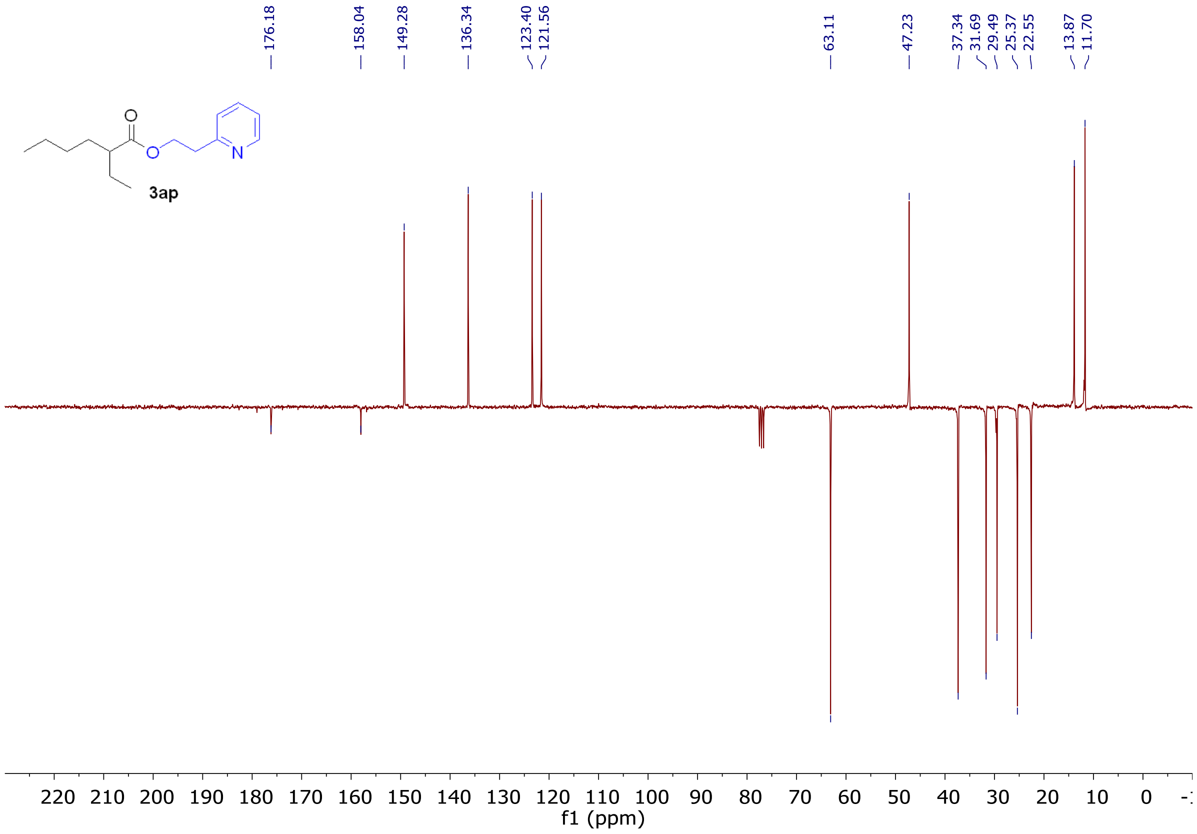

Figure S50. ^{13}C NMR (75 MHz, APT, $CDCl_3$) of **3ap**

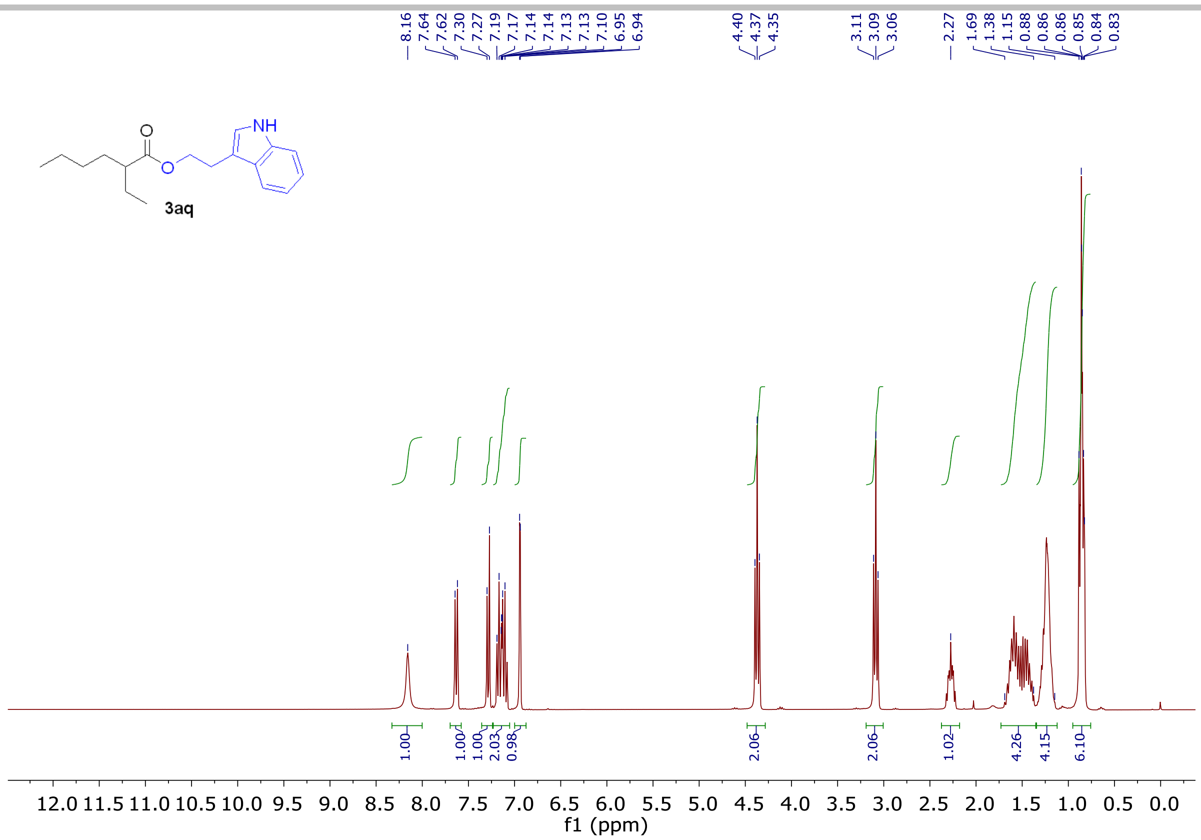

Figure S51. ^{1}H NMR (300 MHz, $CDCl_3$) of **3aq**

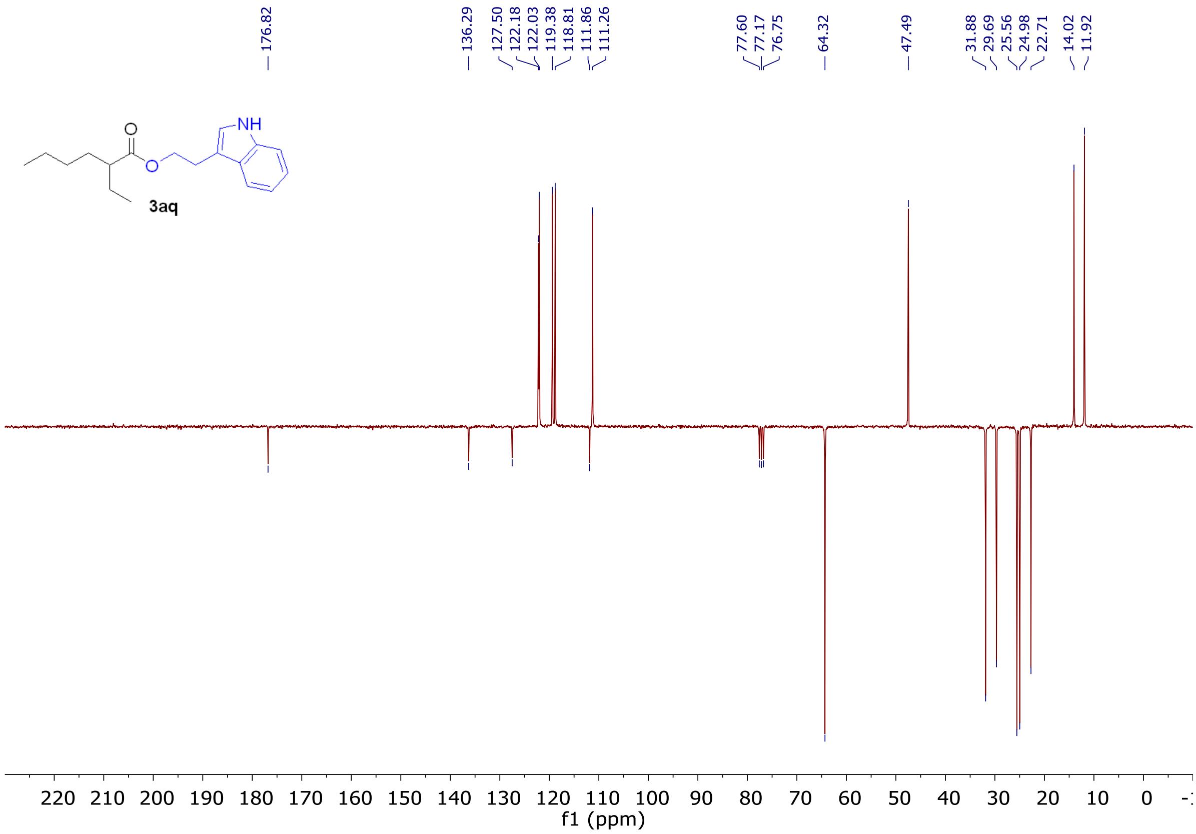

Figure S52. ^{13}C NMR (75 MHz, APT, $CDCl_3$) of **3aq**

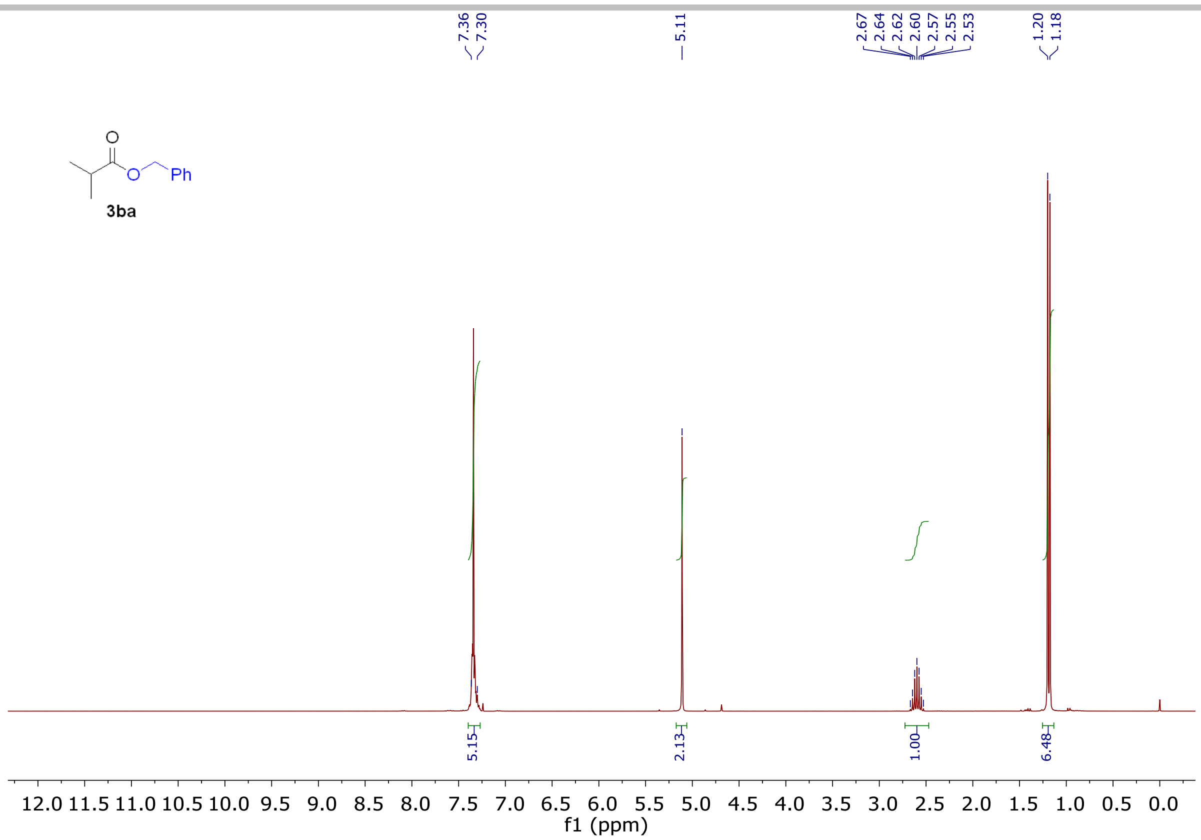

Figure S53. ^{1}H NMR (300 MHz, $CDCl_3$) of **3ba**

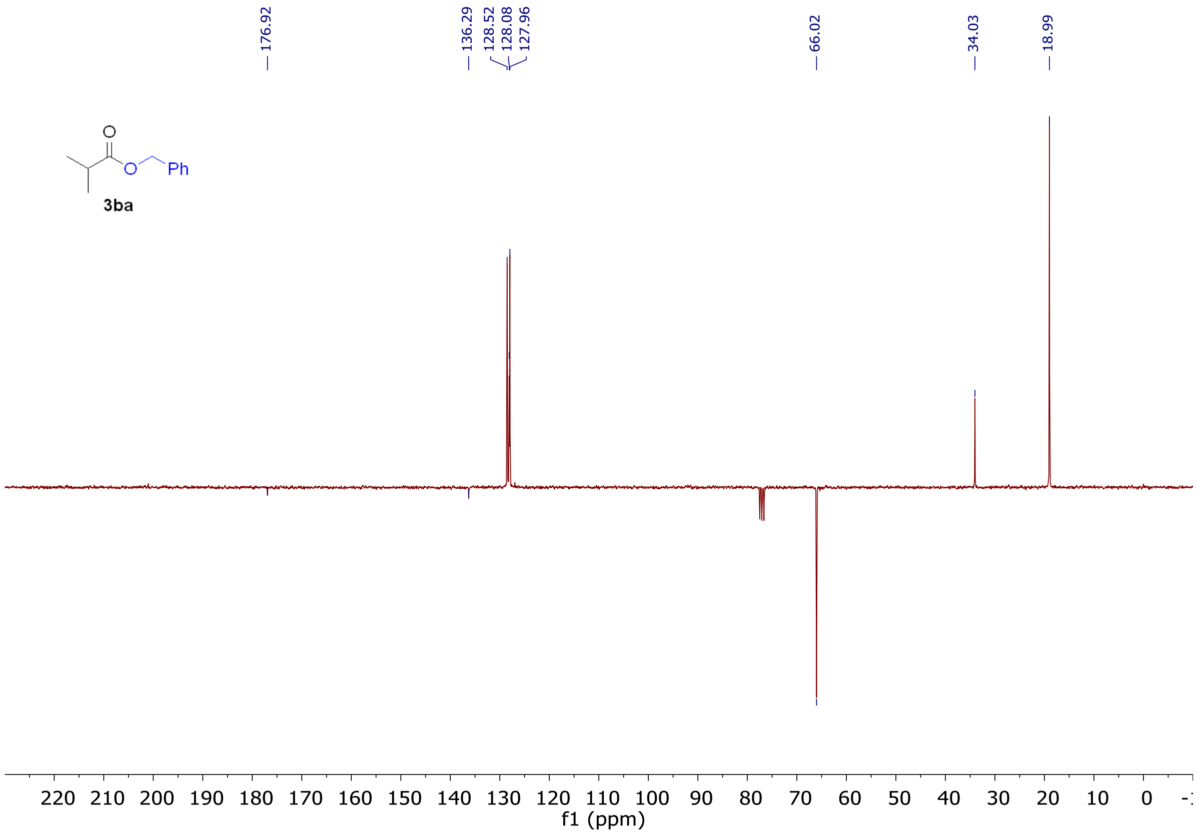

Figure S54. ^{13}C NMR (75 MHz, APT, $CDCl_3$) of **3ba**

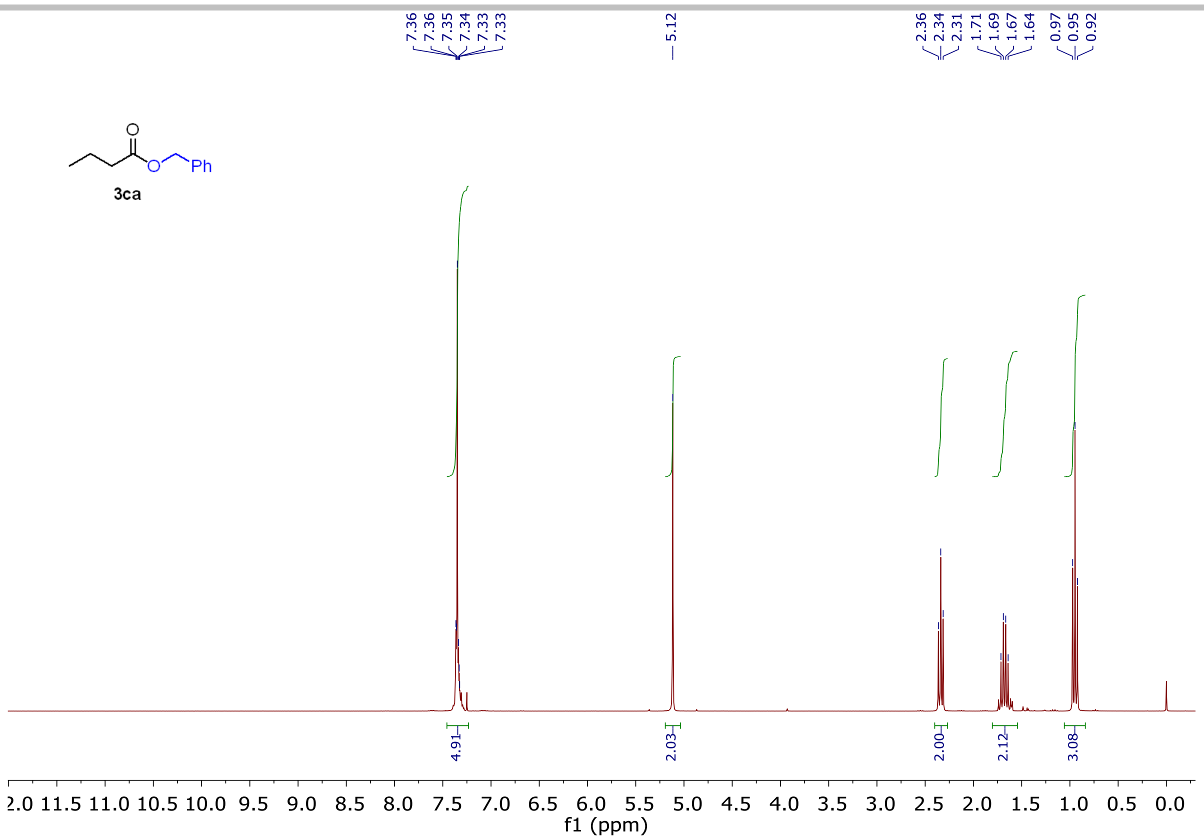

Figure S55. ^{1}H NMR (300 MHz, $CDCl_3$) of **3ca**

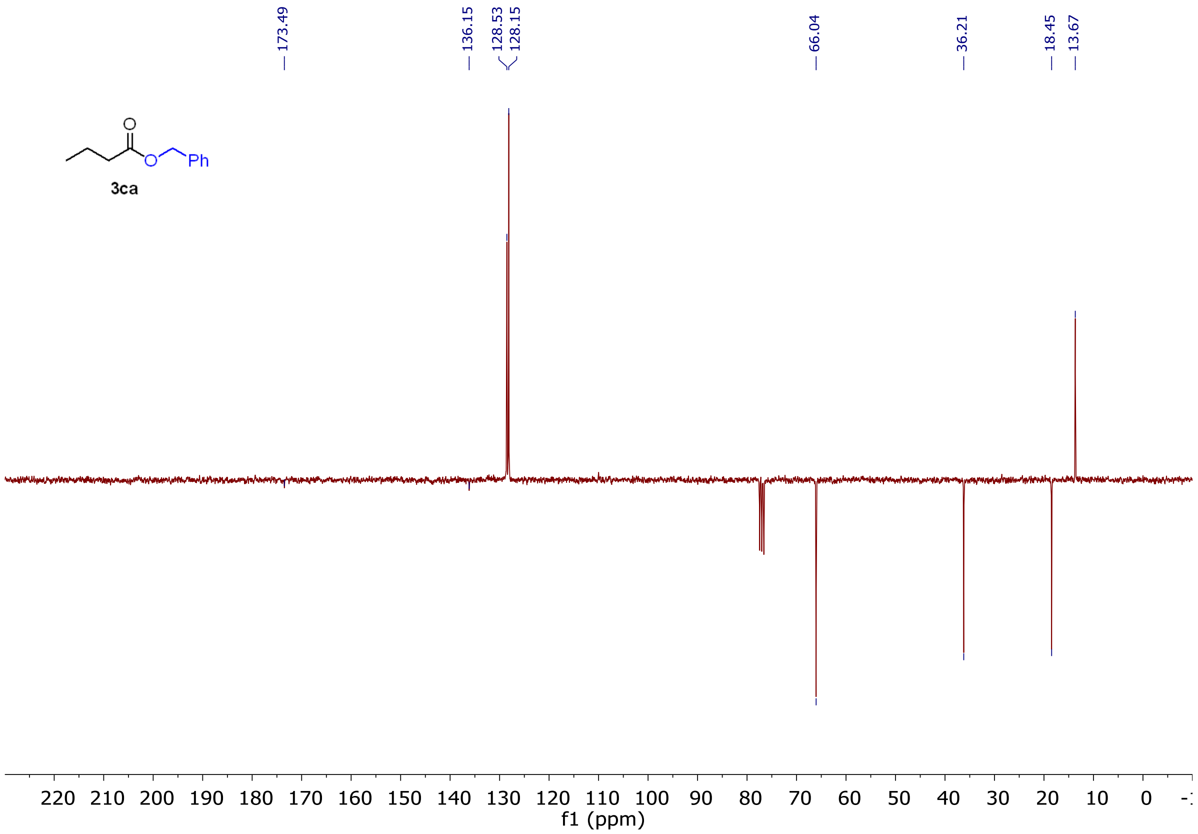

Figure S56. ^{13}C NMR (75 MHz, APT, $CDCl_3$) of **3ca**

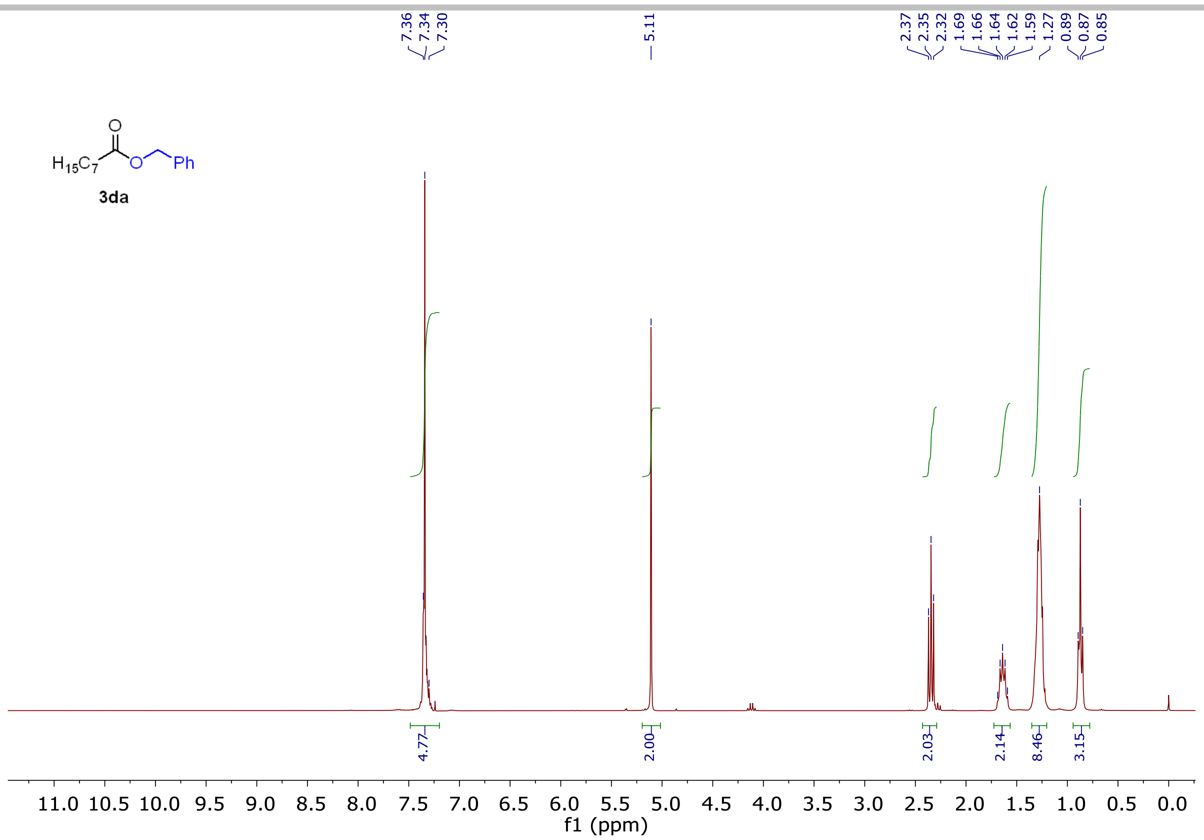

Figure S57. ^{1}H NMR (300 MHz, $CDCl_3$) of **3da**

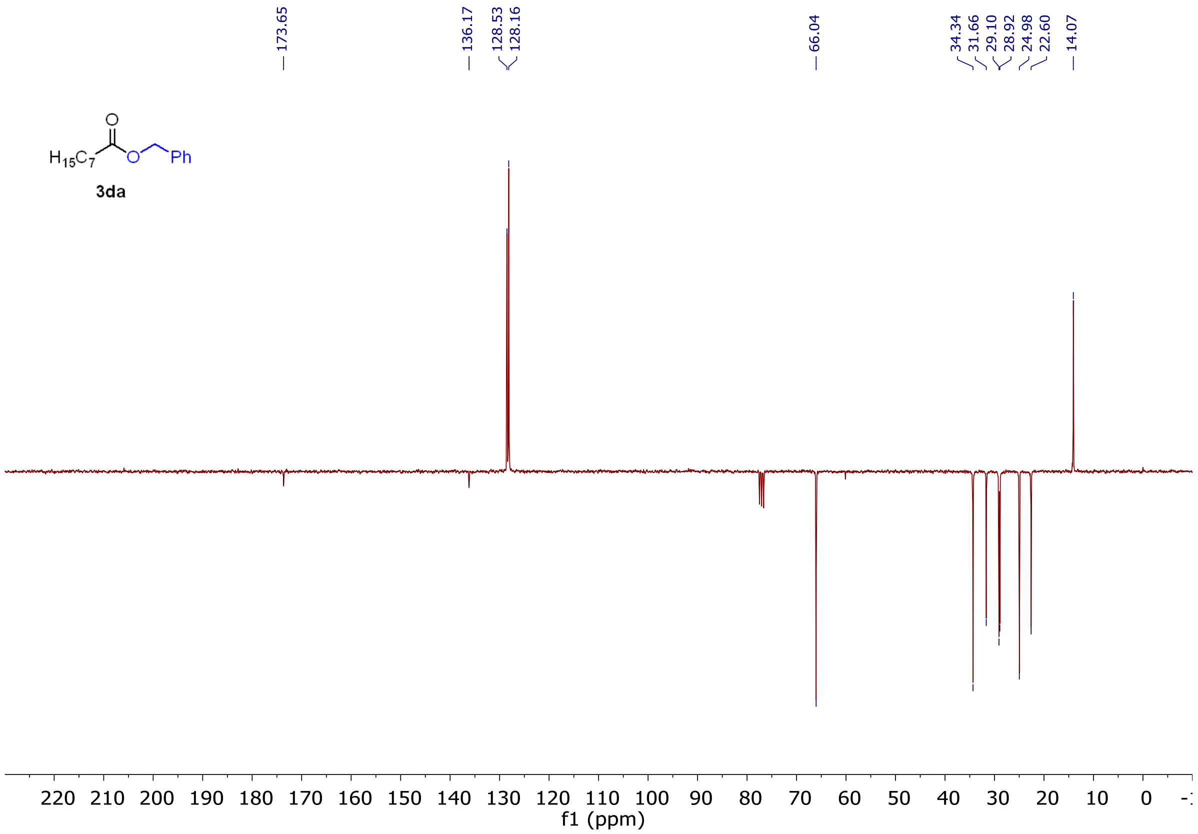

Figure S58. ^{13}C NMR (75 MHz, APT, $CDCl_3$) of **3da**

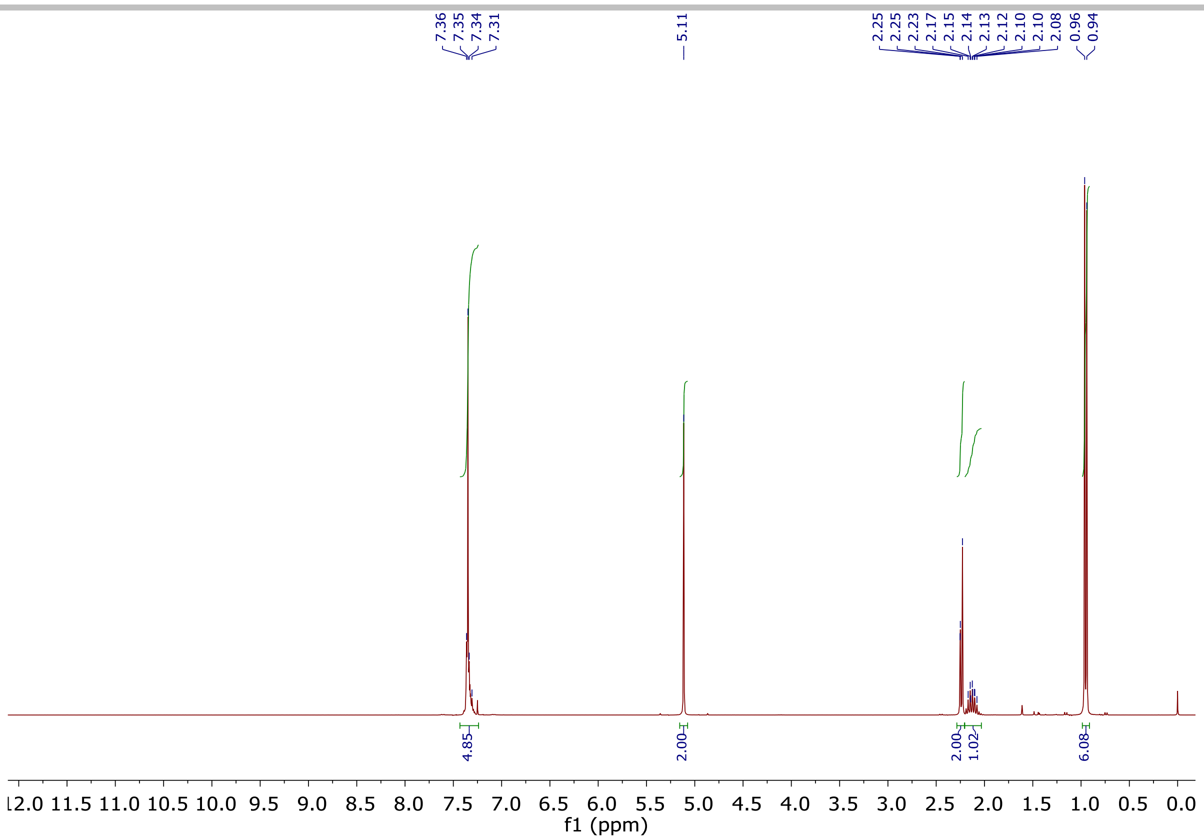

Figure S59. ^{1}H NMR (300 MHz, $CDCl_3$) of **3ea**

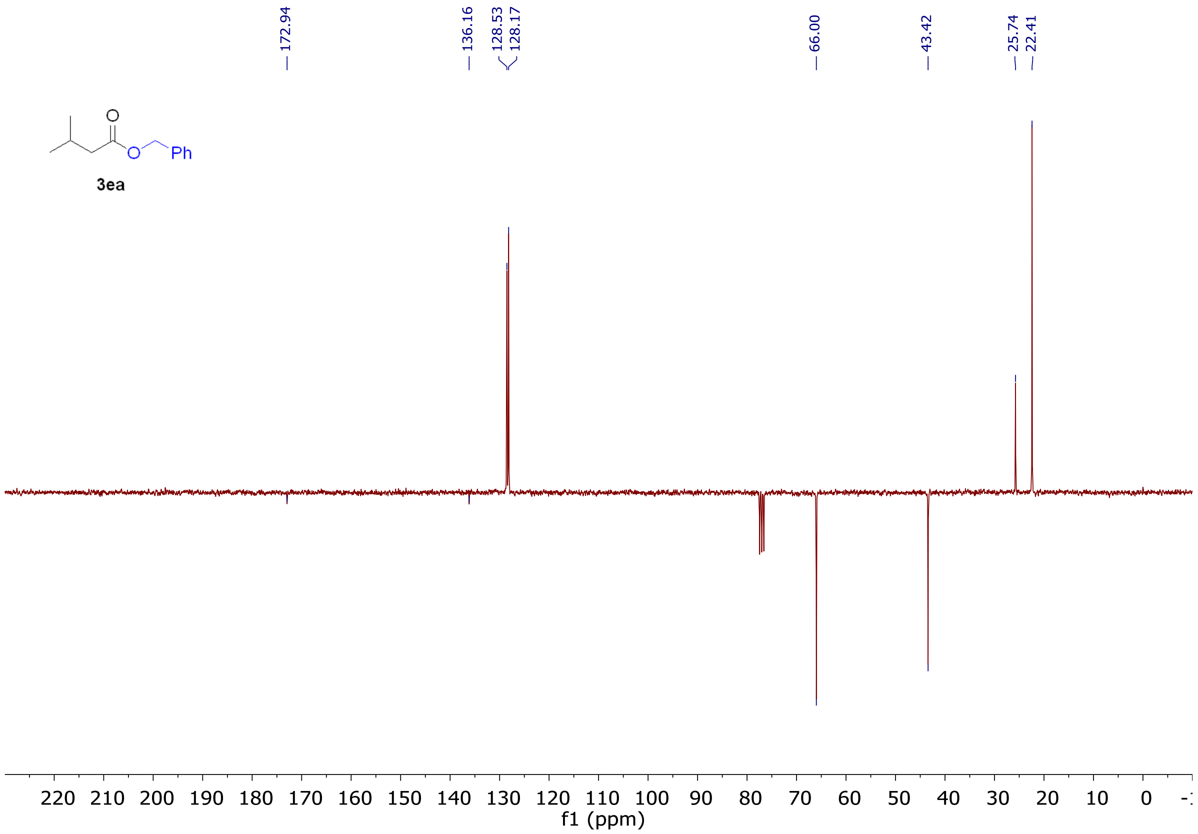

Figure S60. ^{13}C NMR (75 MHz, APT, $CDCl_3$) of **3ea**

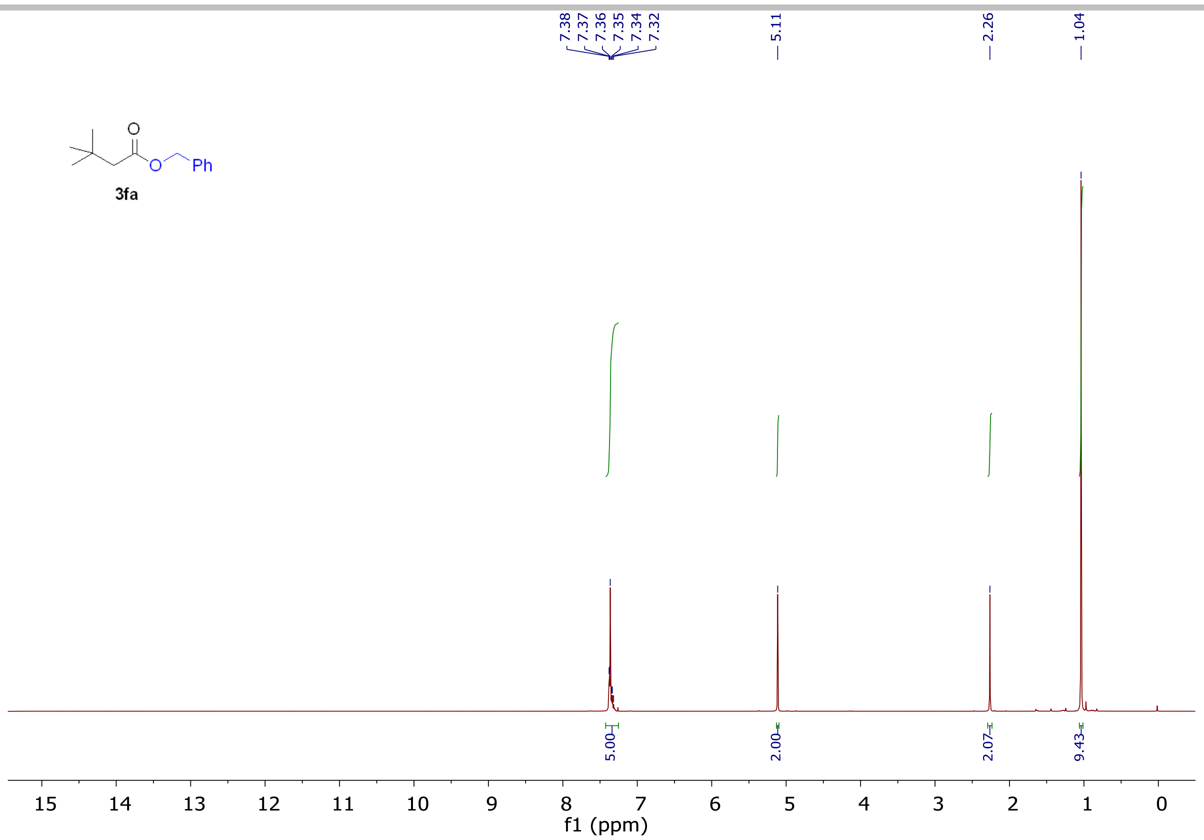

Figure S61. ^{1}H NMR (300 MHz, $CDCl_3$) of **3fa**

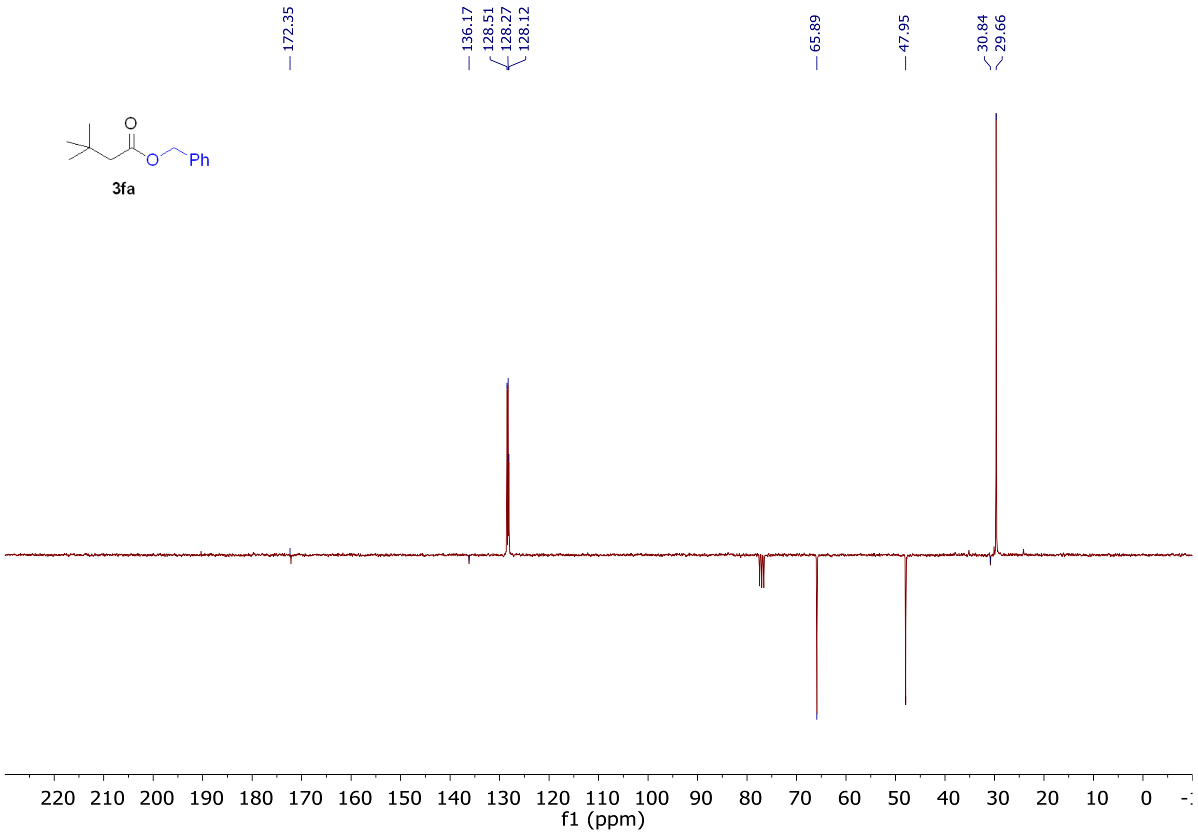

Figure S62. ^{13}C NMR (75 MHz, APT, $CDCl_3$) of **3fa**

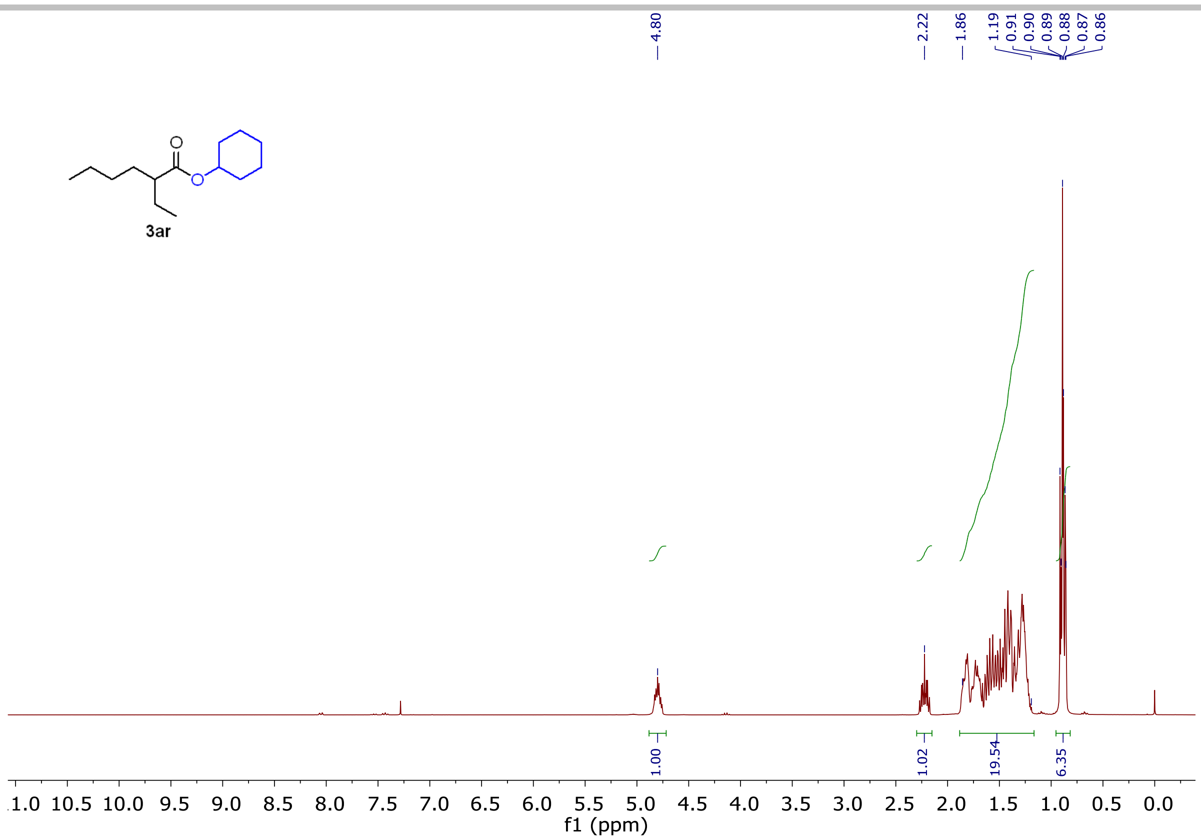

Figure S63. ^{1}H NMR (300 MHz, $CDCl_3$) of **3ar**

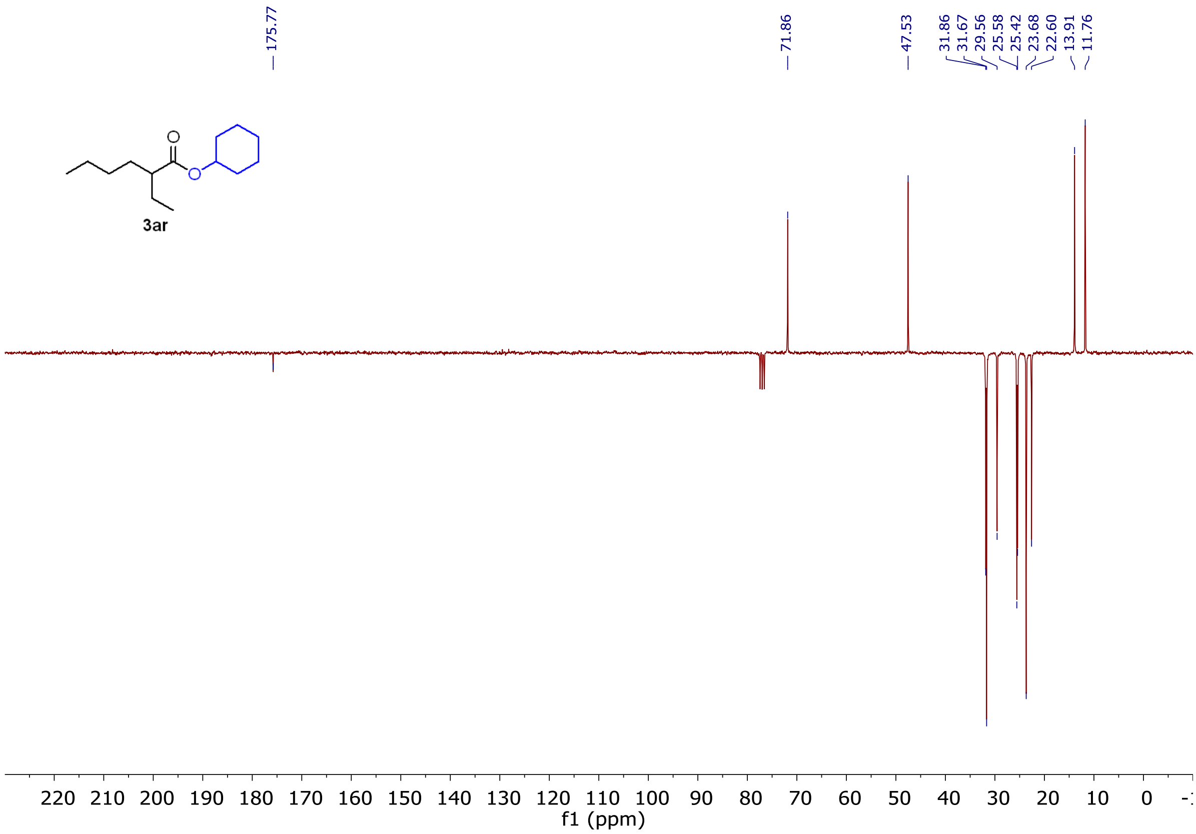

Figure S64. ^{13}C NMR (75 MHz, APT, $CDCl_3$) of **3ar**

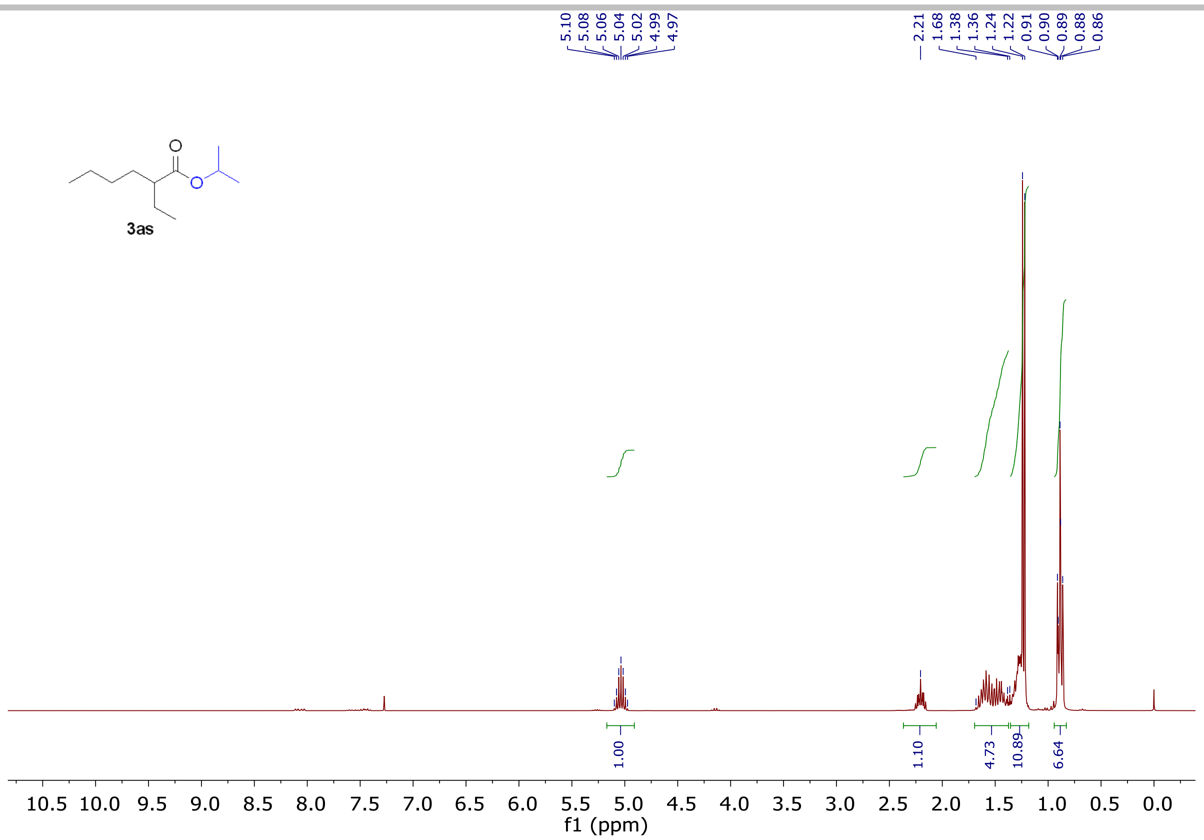

Figure S65. ^{1}H NMR (300 MHz, $CDCl_3$) of **3as**

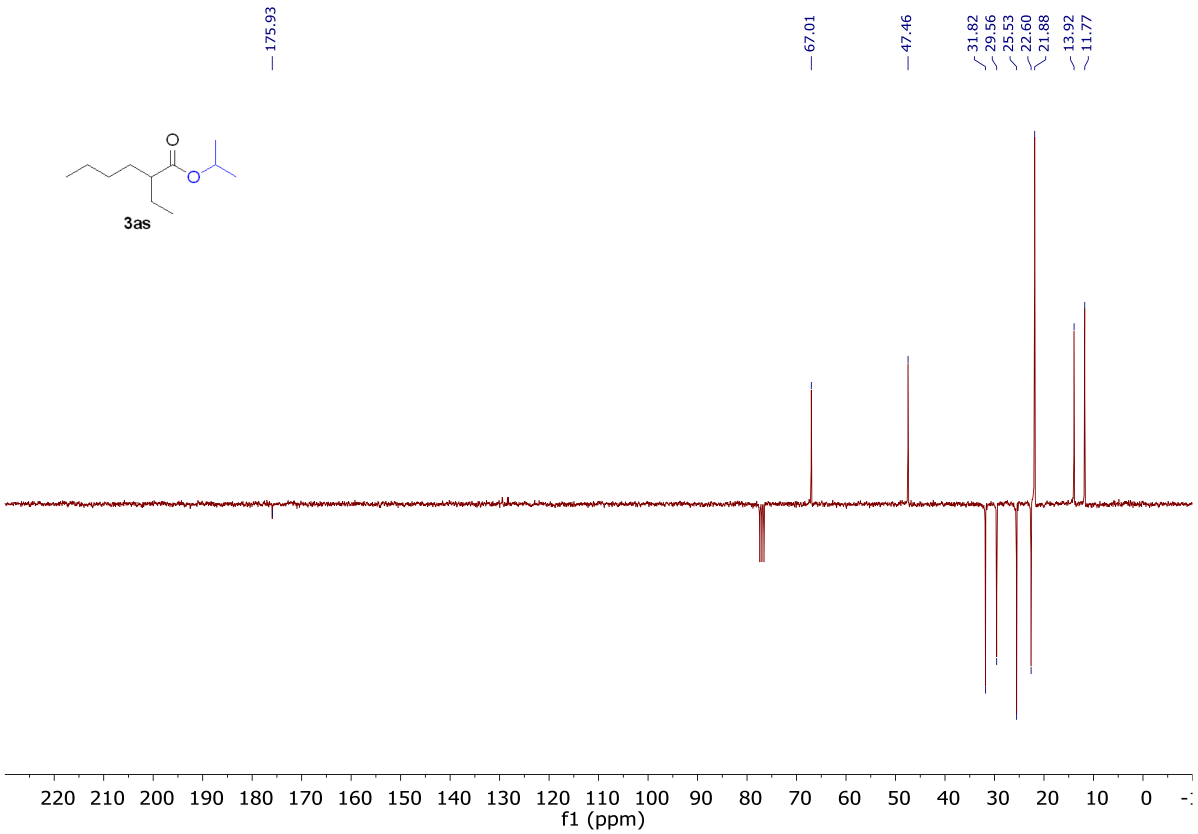

Figure S66. ^{13}C NMR (75 MHz, APT, $CDCl_3$) of **3as**

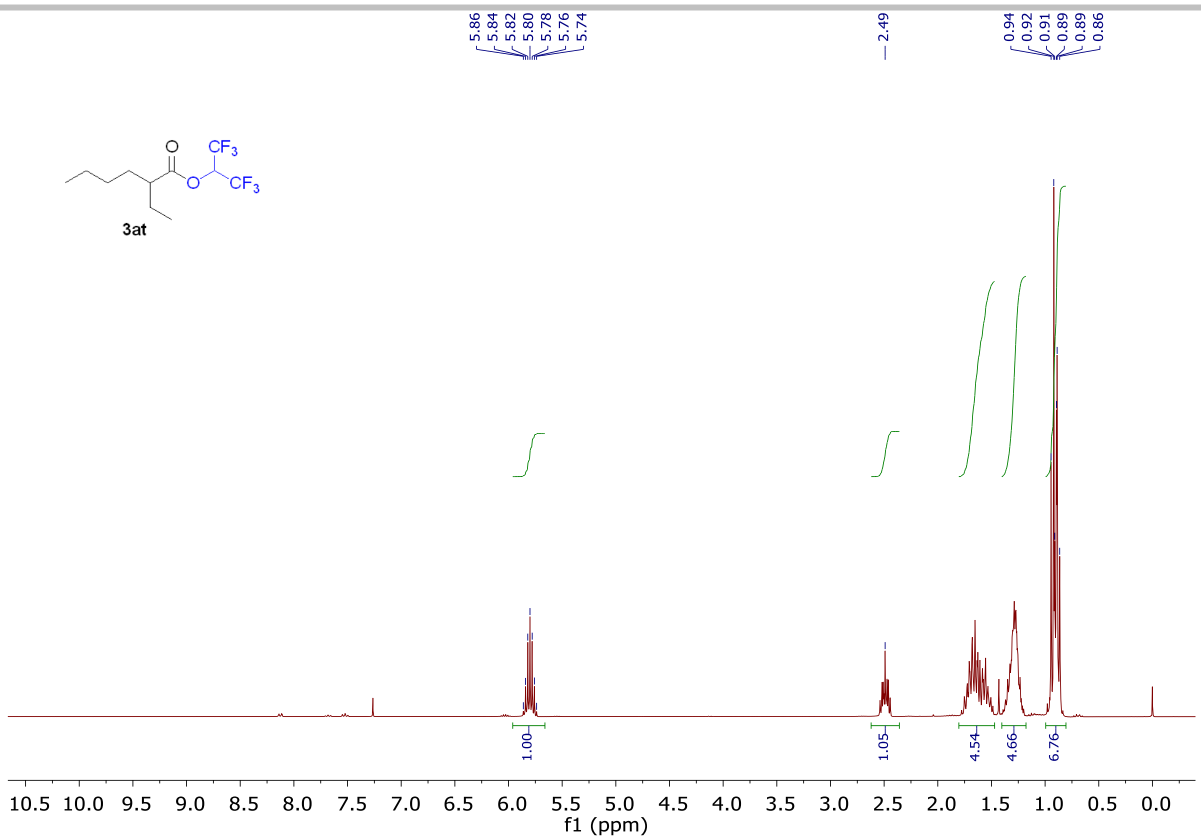

Figure S67. ^{1}H NMR (300 MHz, $CDCl_3$) of **3at**

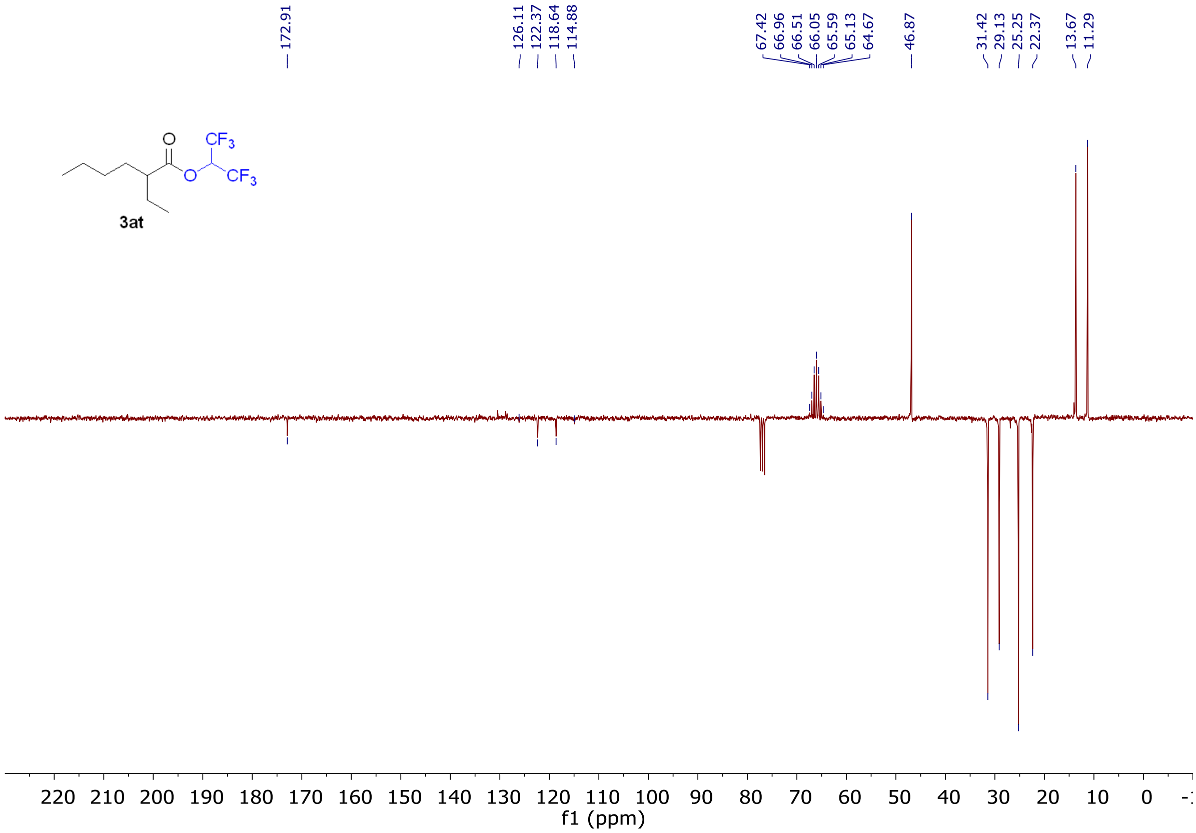

Figure S68. ^{13}C NMR (75 MHz, APT, $CDCl_3$) of **3at**

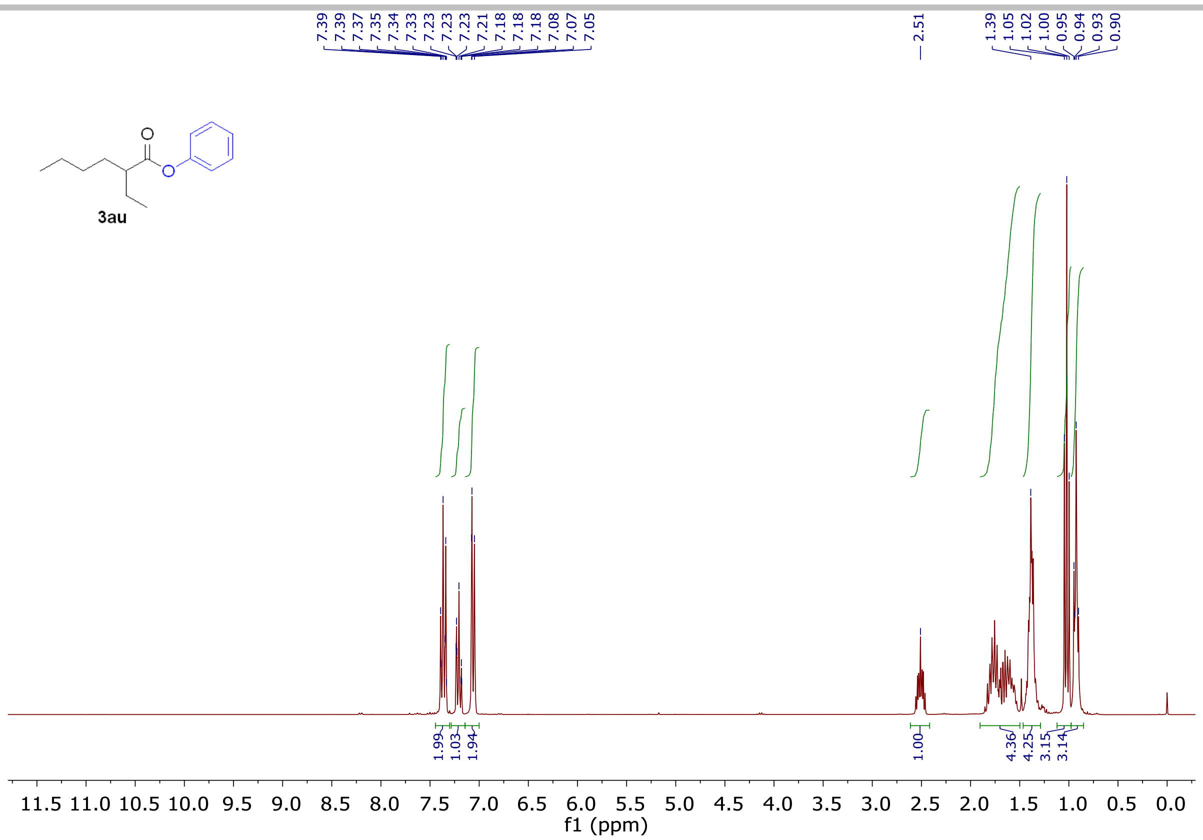

Figure S69. ^{1}H NMR (300 MHz, $CDCl_3$) of **3au**

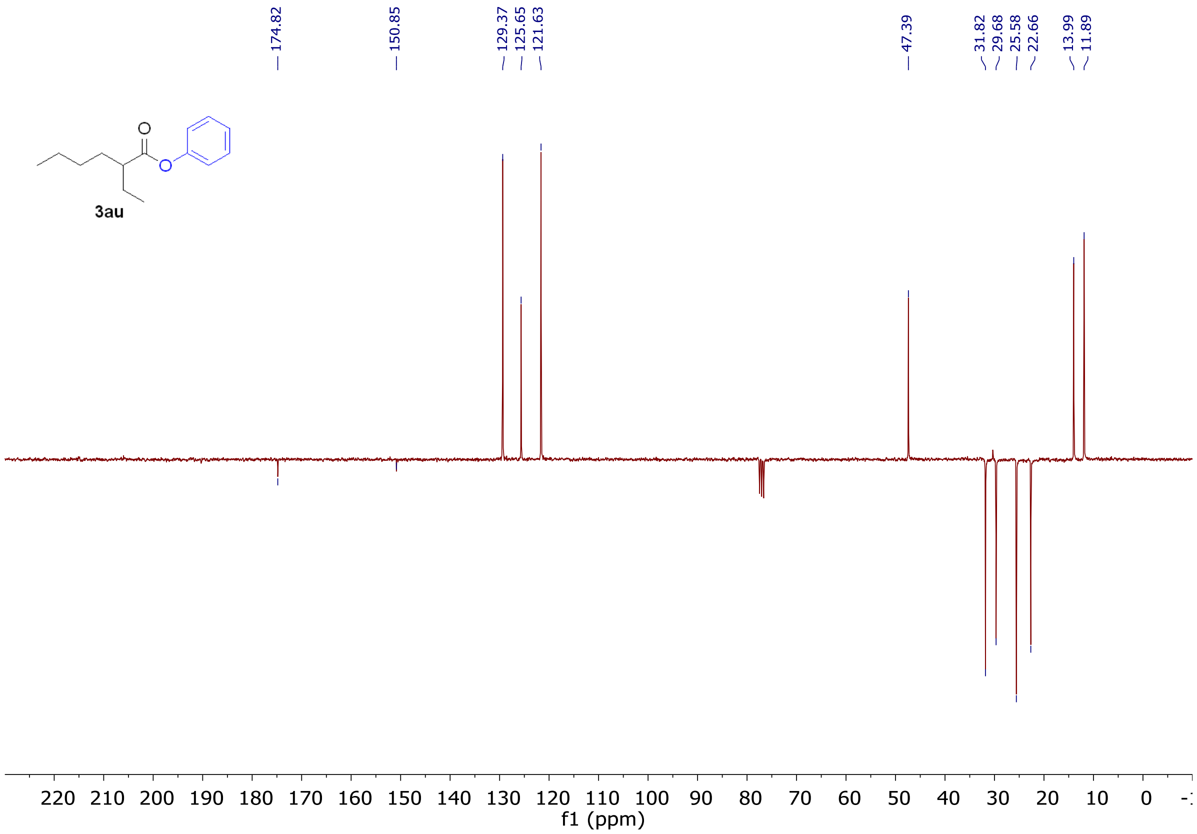

Figure S70. ^{13}C NMR (75 MHz, APT, $CDCl_3$) of **3au**

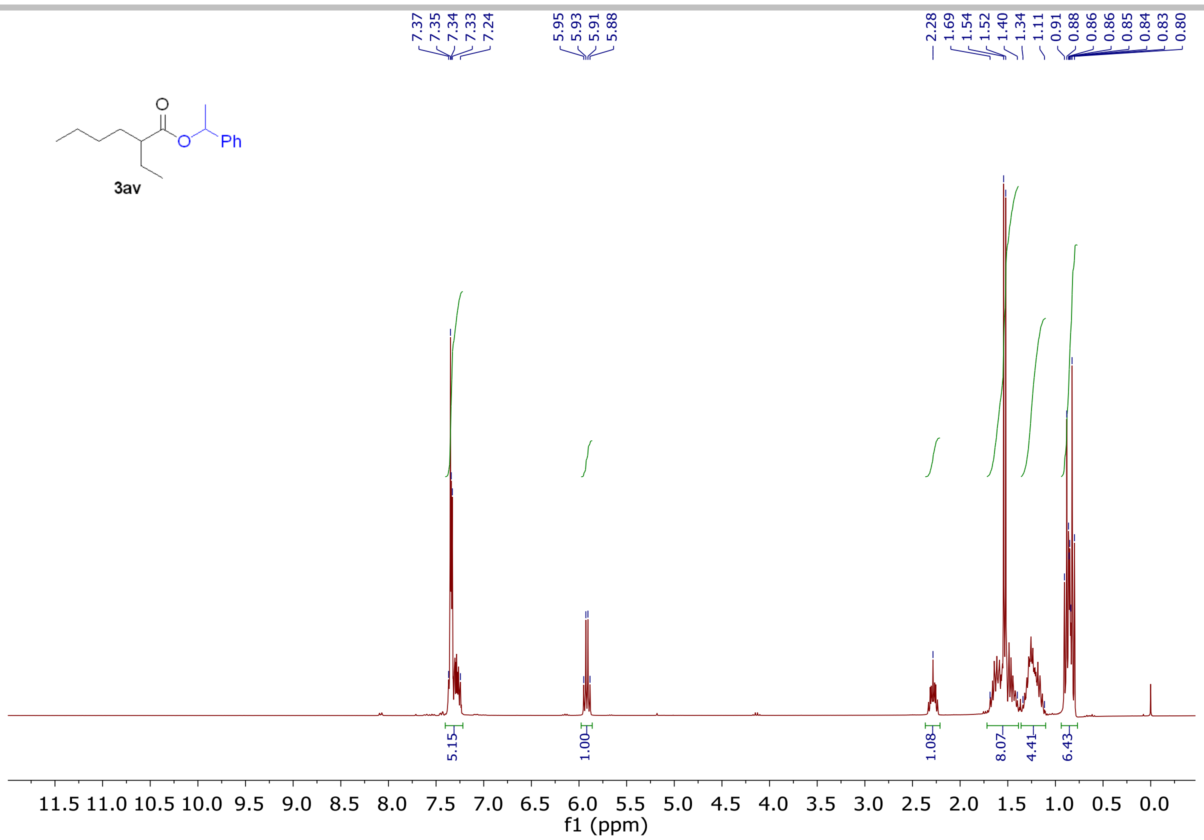

Figure S71. ^{1}H NMR (300 MHz, $CDCl_3$) of **3av**

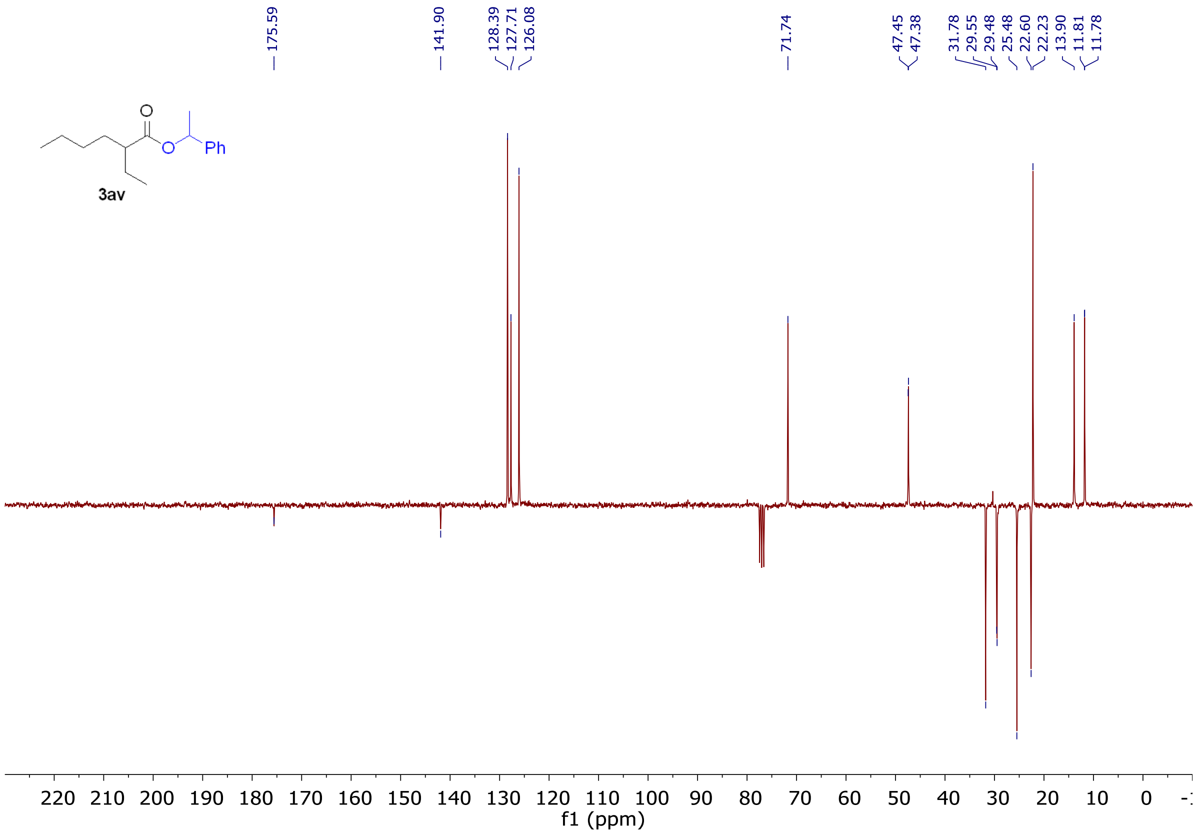

Figure S72. ^{13}C NMR (75 MHz, APT, $CDCl_3$) of **3av**

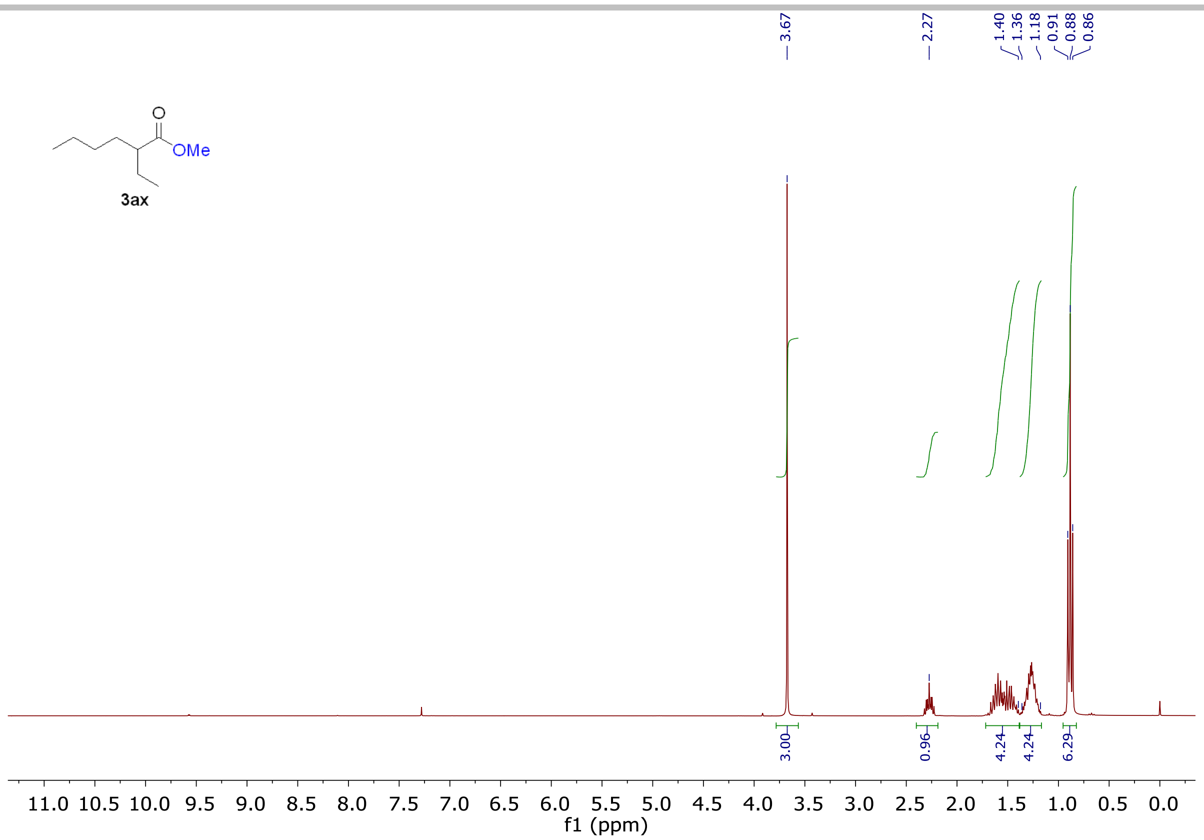

Figure S73. ^{1}H NMR (300 MHz, $CDCl_3$) of **3ax**

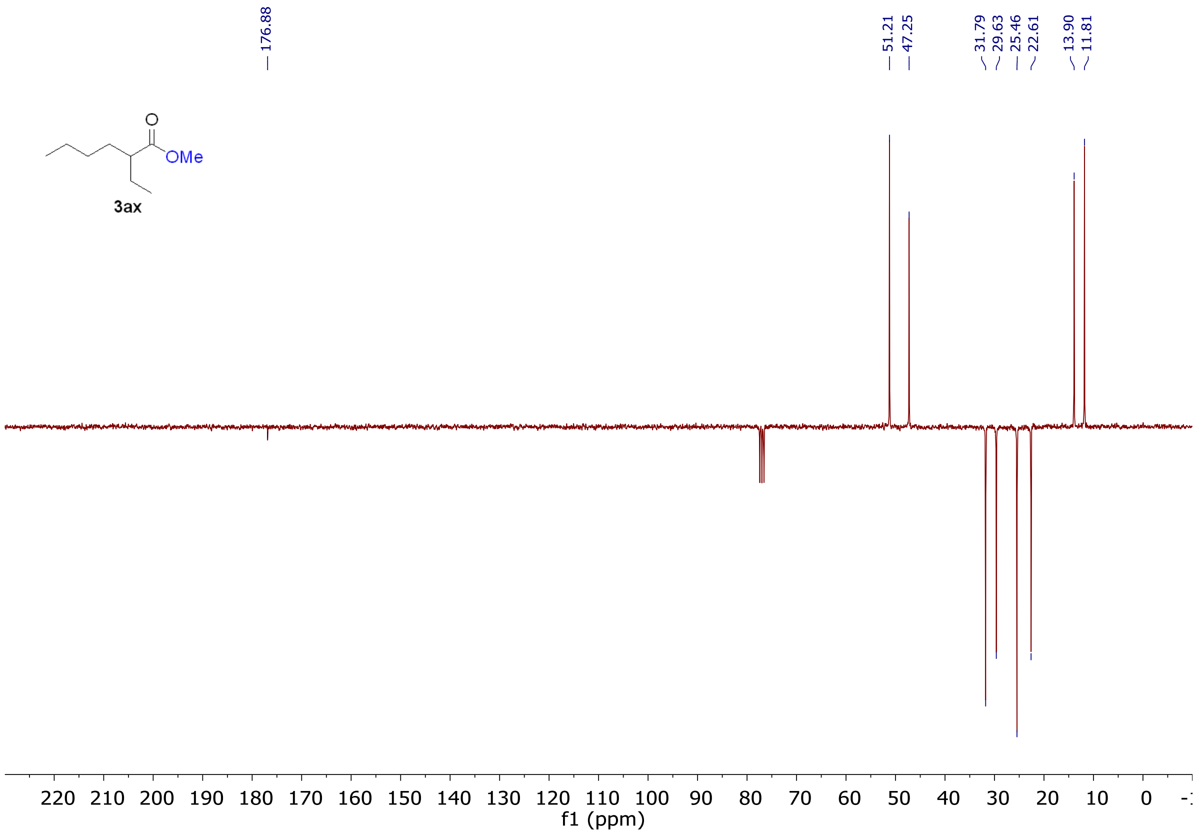

Figure S74. ^{13}C NMR (75 MHz, APT, $CDCl_3$) of **3ax**

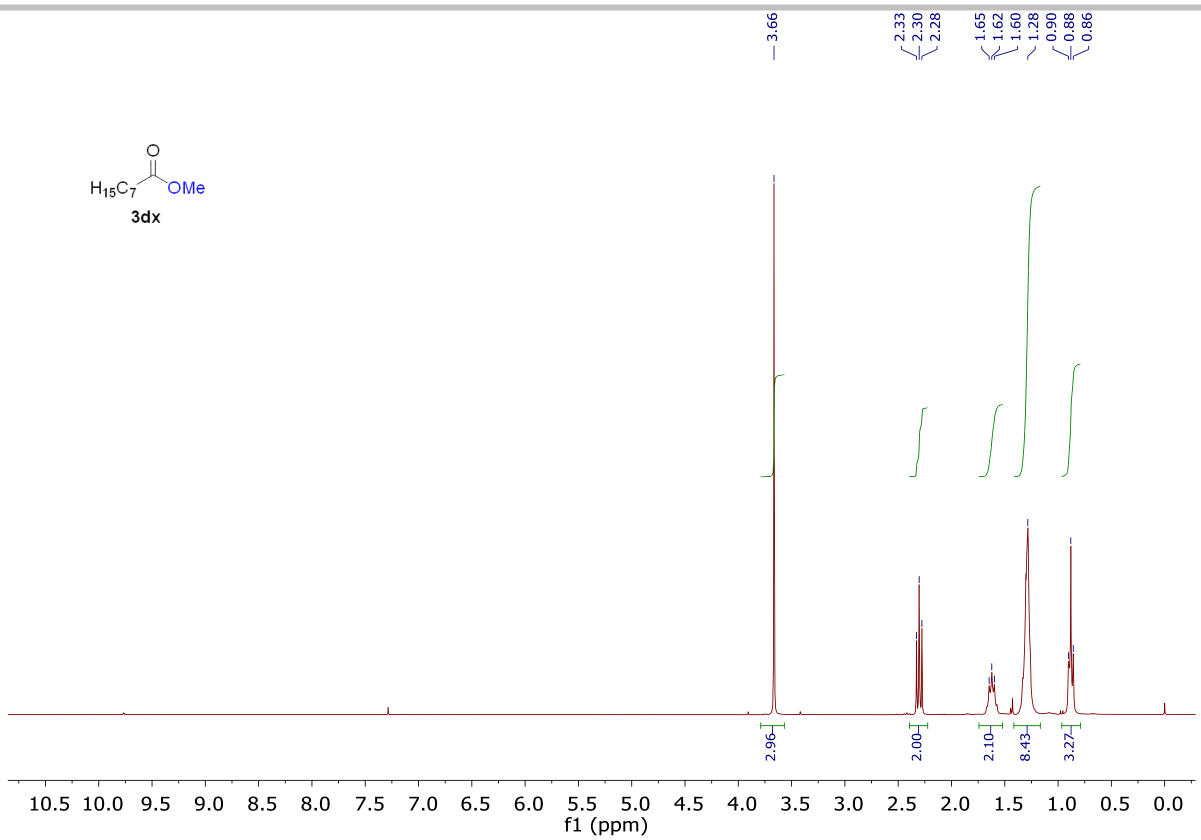

Figure S75. ^{1}H NMR (300 MHz, $CDCl_3$) of **3dx**

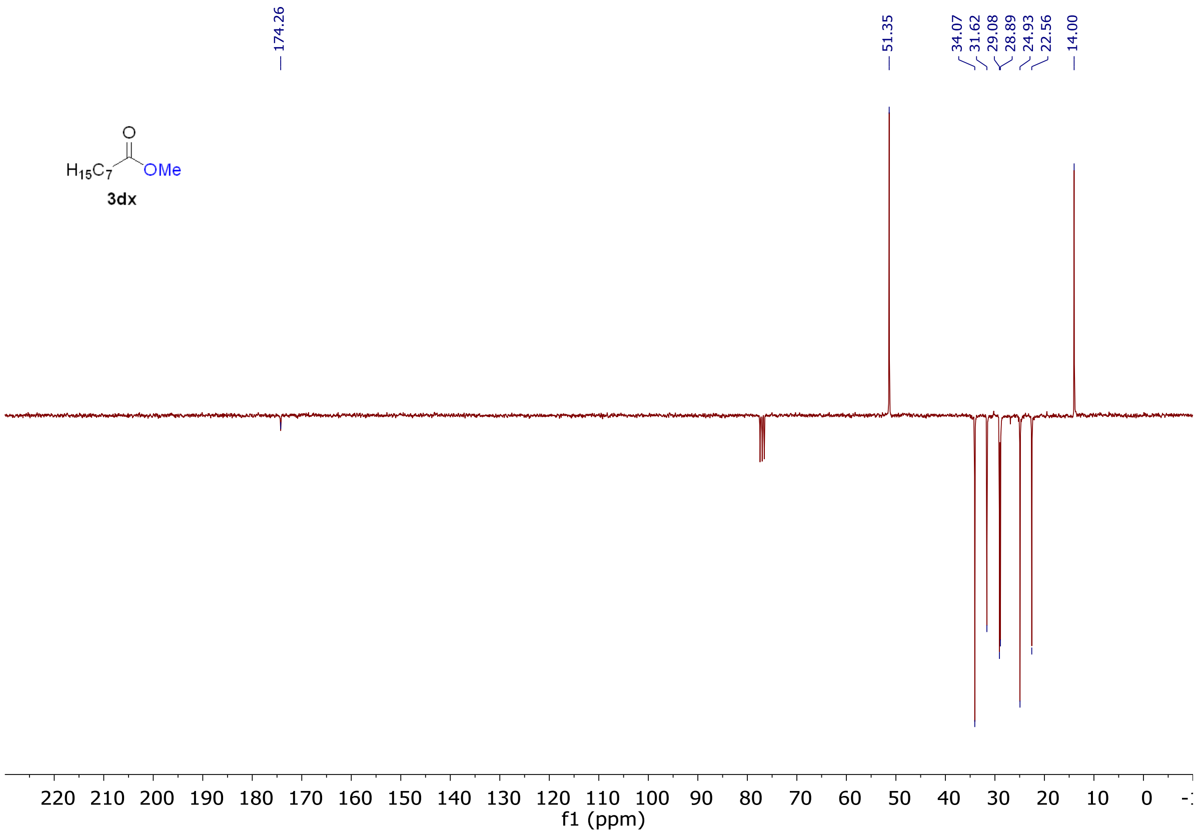

Figure S76. ^{13}C NMR (75 MHz, APT, $CDCl_3$) of **3dx**

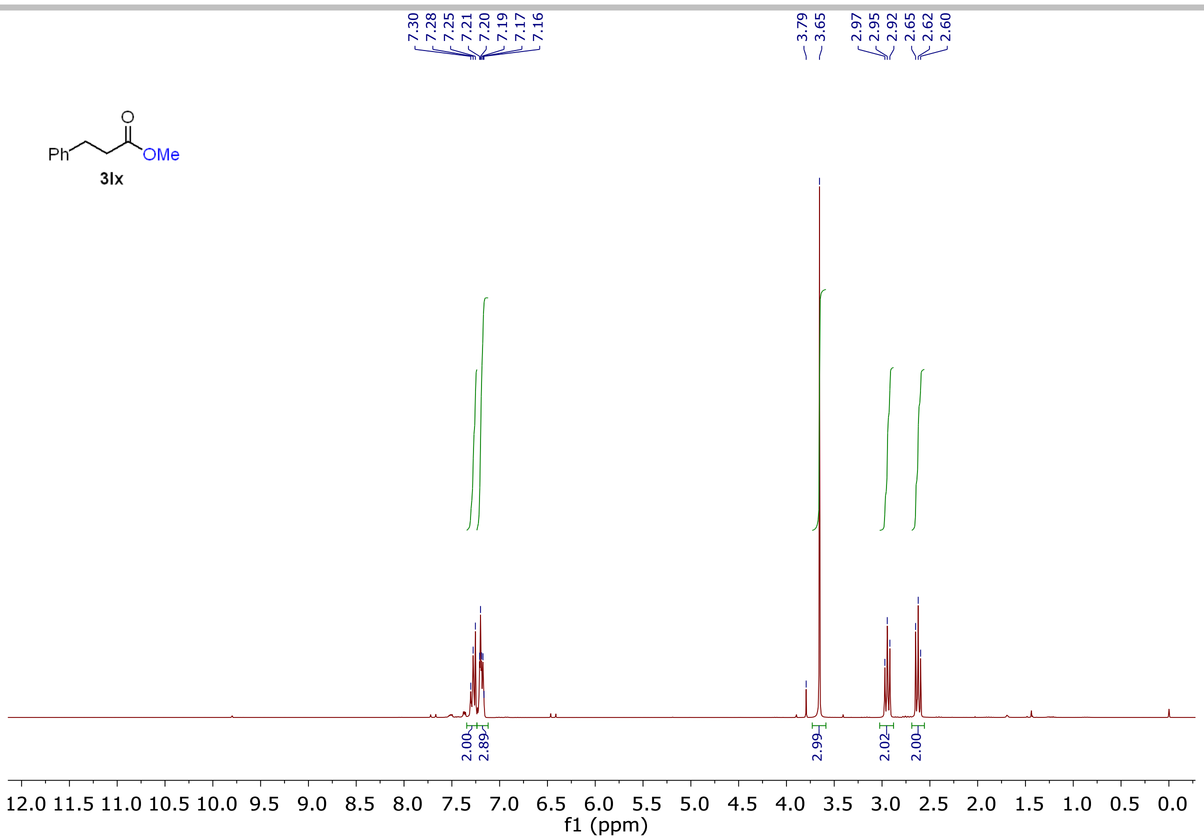

Figure S77. ^{1}H NMR (300 MHz, $CDCl_3$) of **3gx**

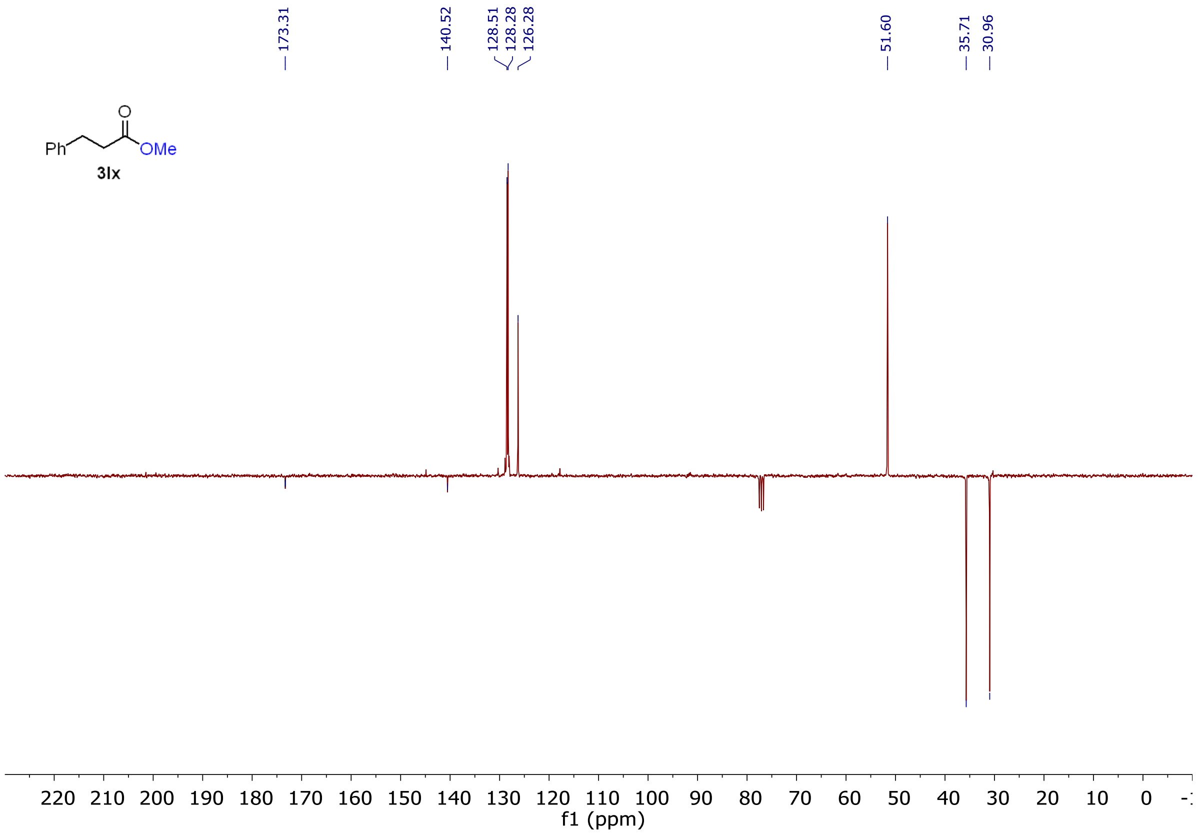

Figure S78. ^{13}C NMR (75 MHz, APT, $CDCl_3$) of **3gx**

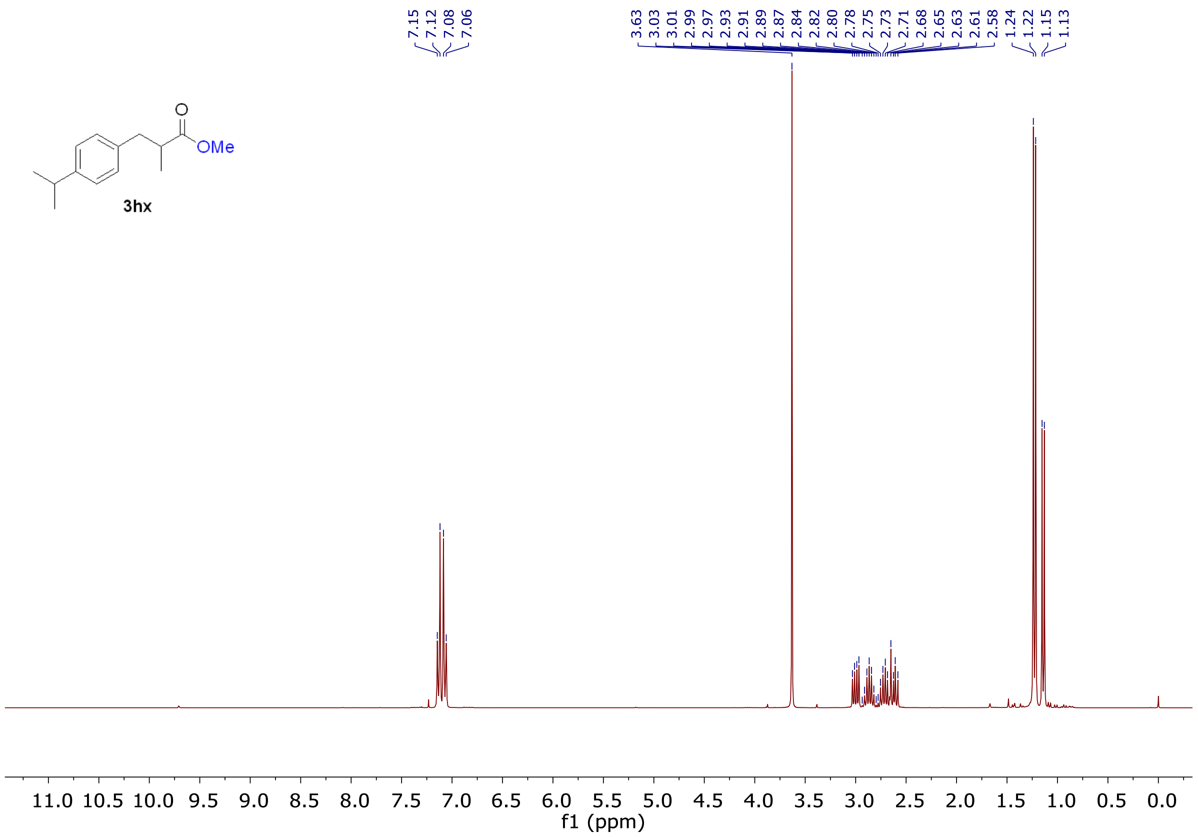

Figure S79. ^{1}H NMR (300 MHz, $CDCl_3$) of **3hx**

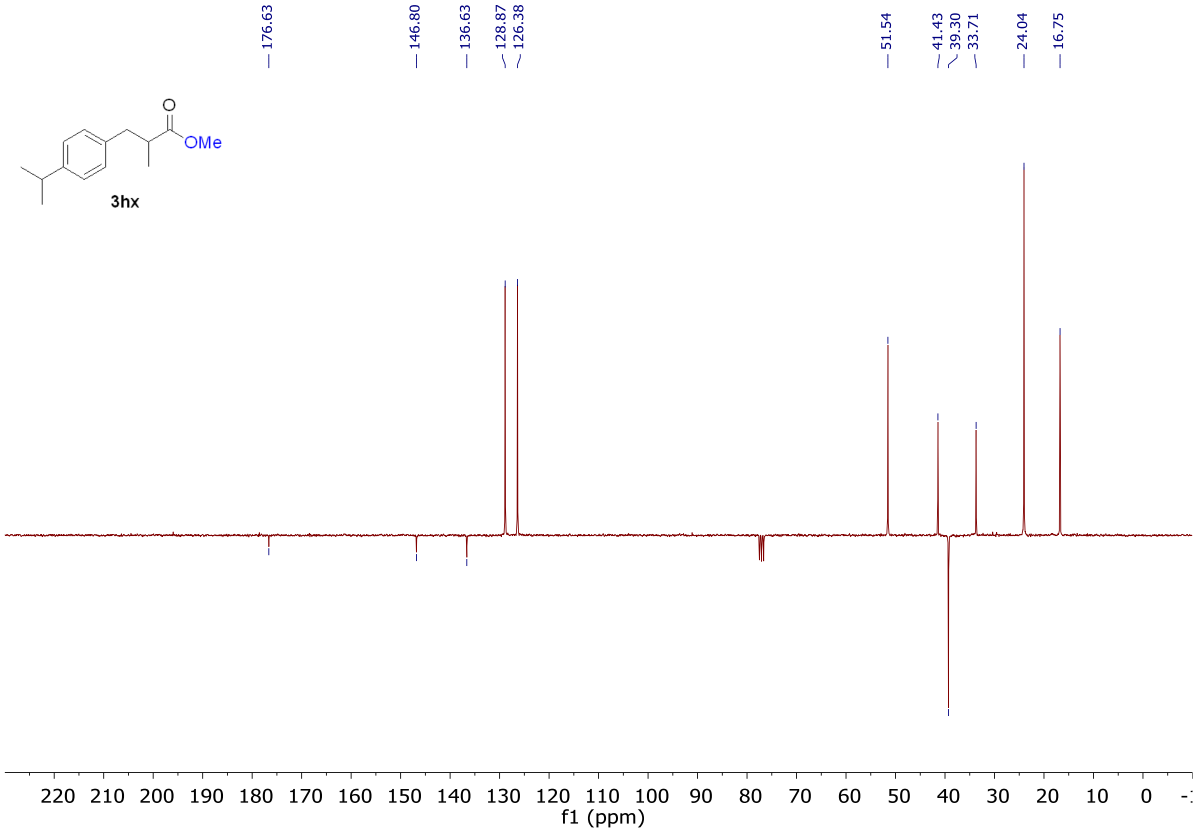

Figure S80. ^{13}C NMR (75 MHz, APT, $CDCl_3$) of **3hx**

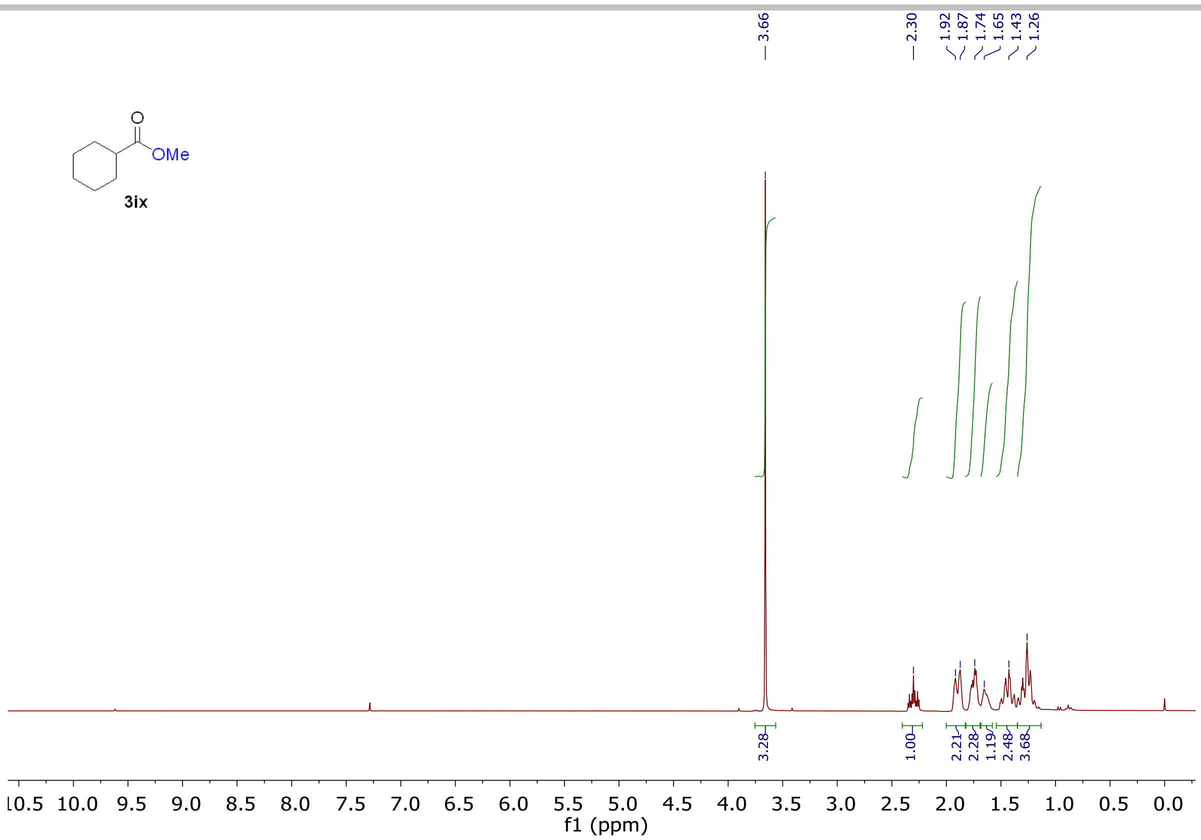

Figure S81. ^{1}H NMR (300 MHz, $CDCl_3$) of **3ix**

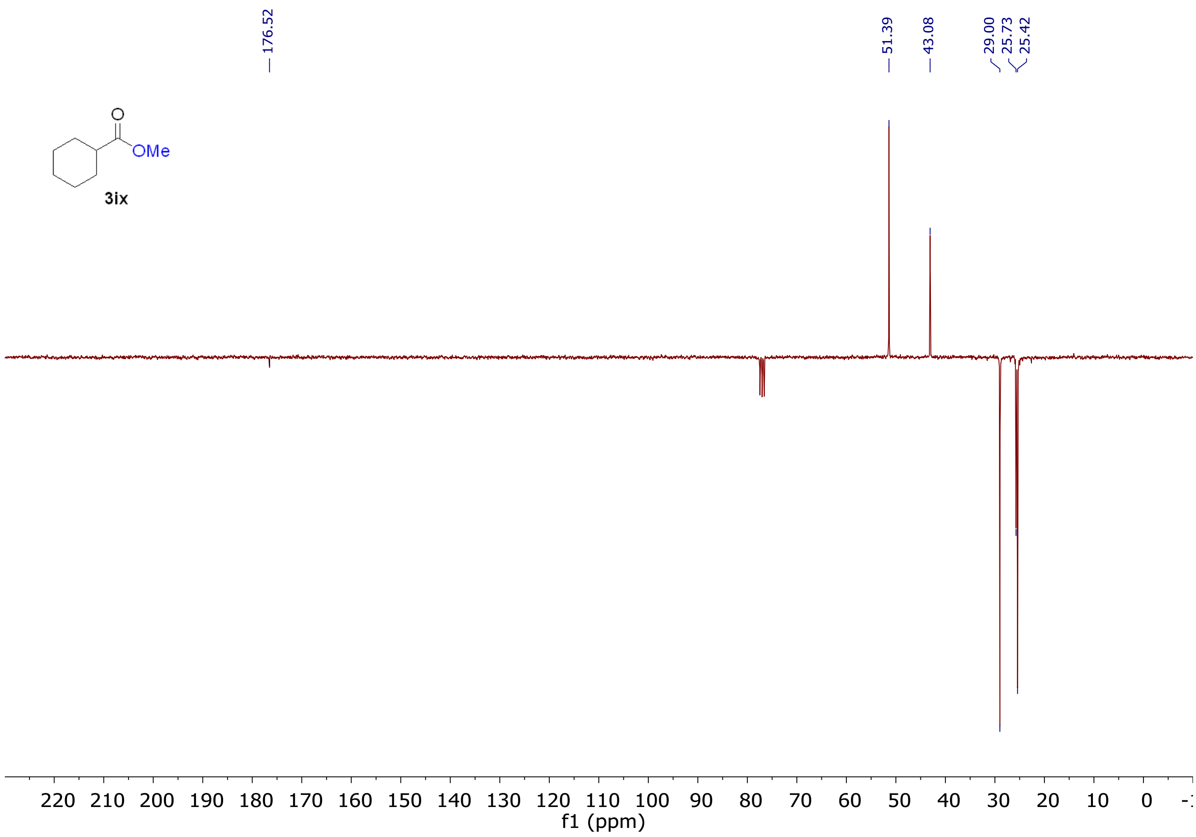

Figure S82. ^{13}C NMR (75 MHz, APT, $CDCl_3$) of **3ix**

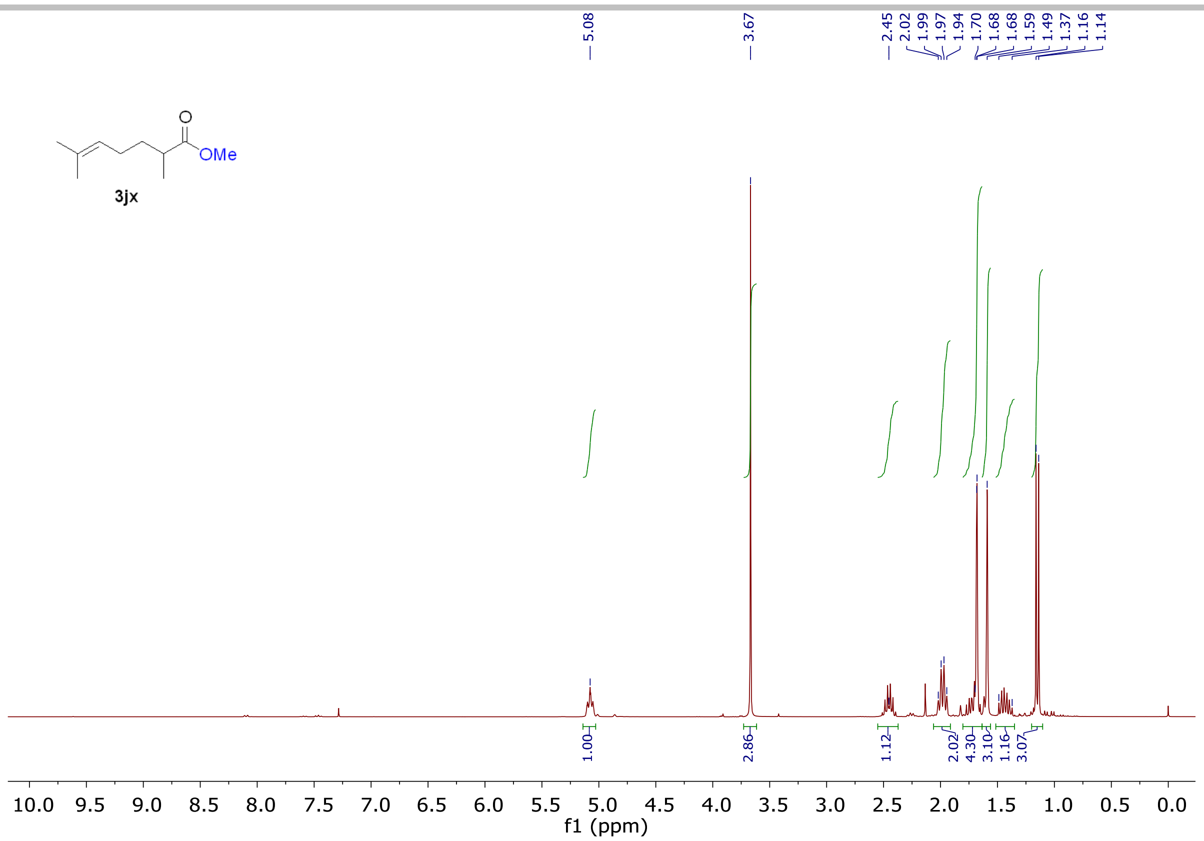

Figure S83. ^{1}H NMR (300 MHz, $CDCl_3$) of **3jx**

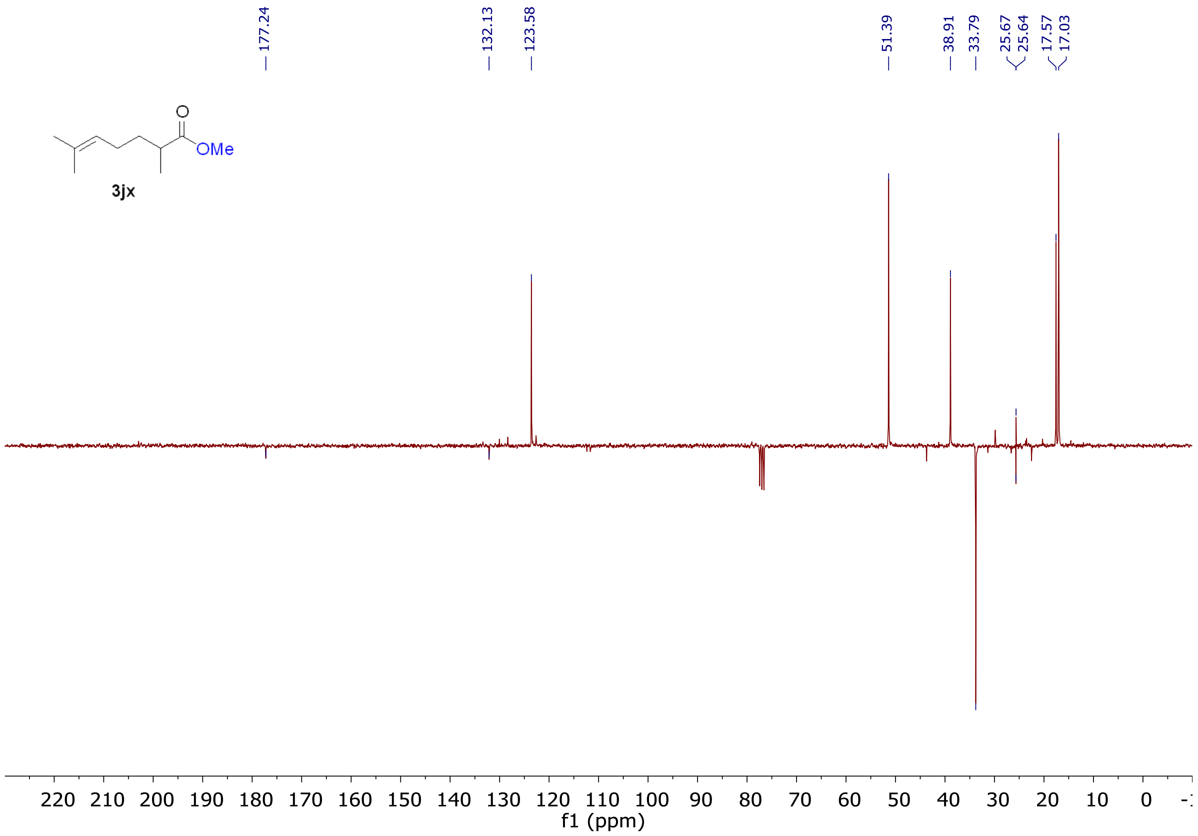

Figure S84. ^{13}C NMR (75 MHz, APT, $CDCl_3$) of **3jx**

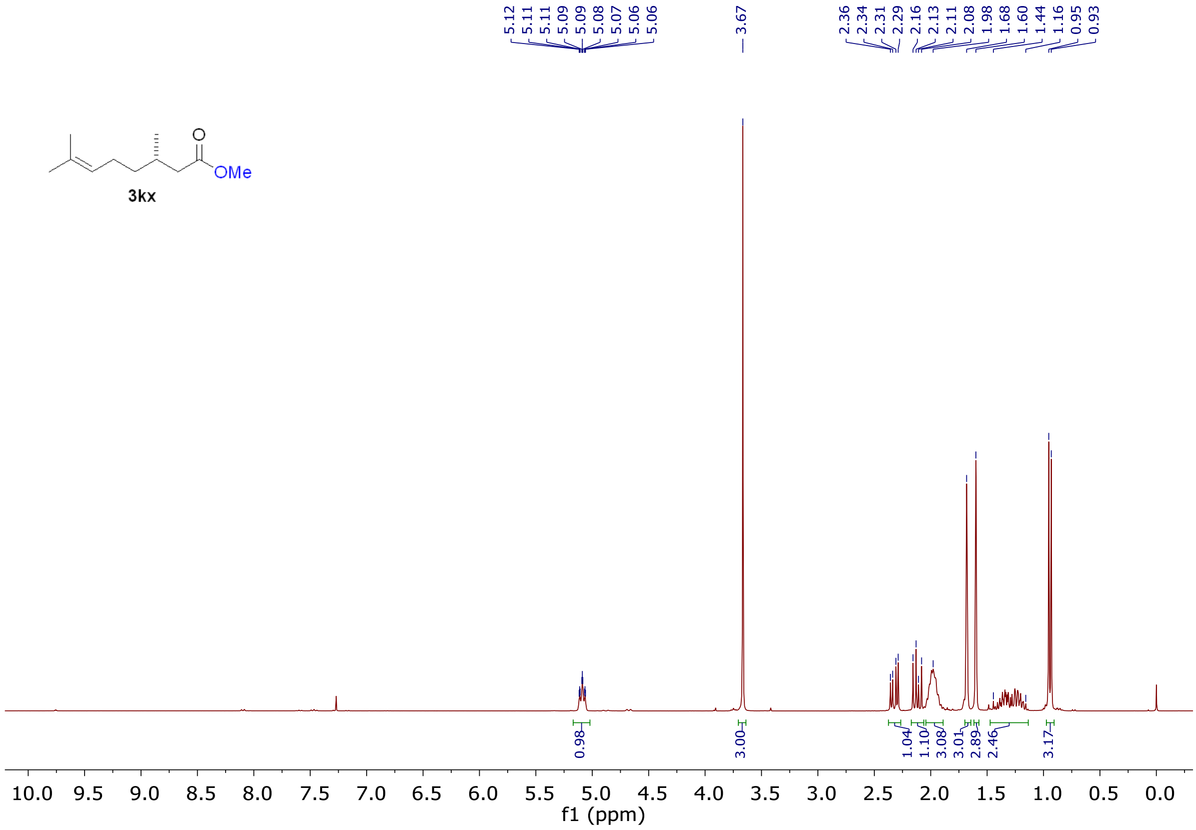

Figure S85. ^{1}H NMR (300 MHz, $CDCl_3$) of **3kx**

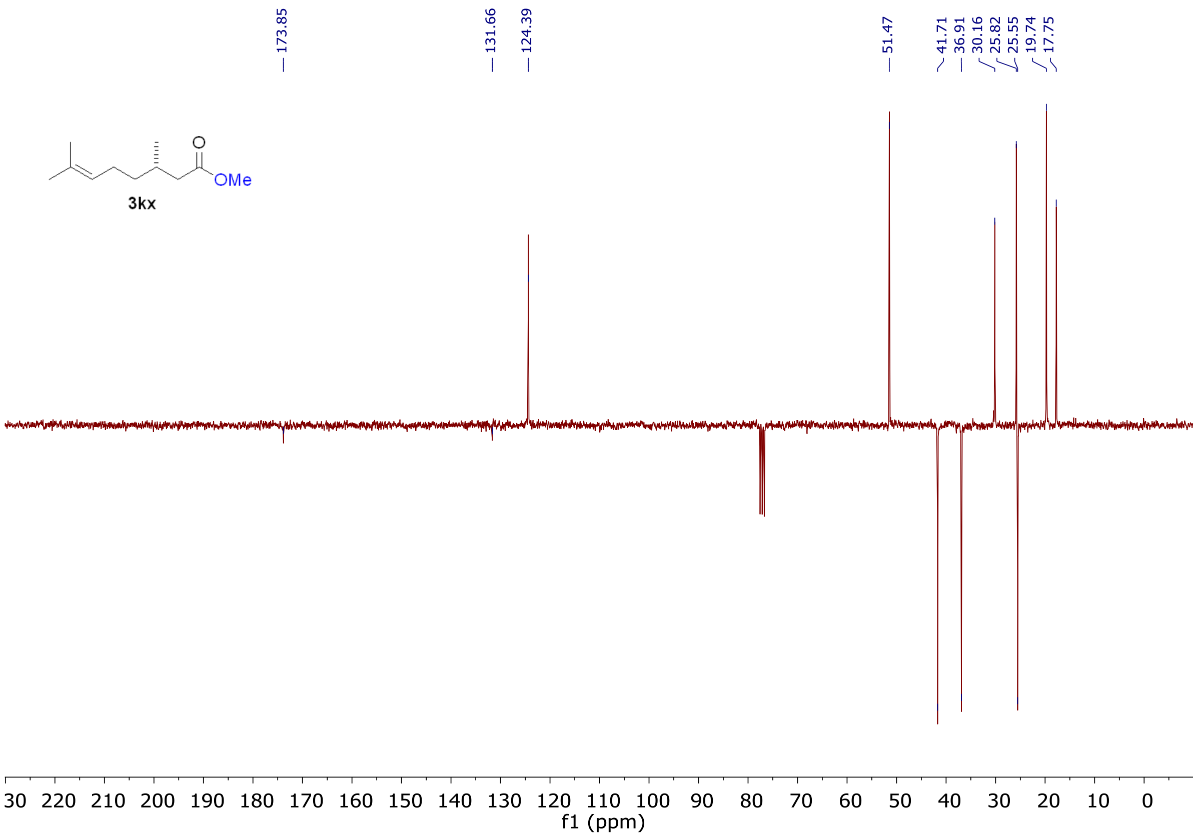

Figure S86. ^{13}C NMR (75 MHz, APT, $CDCl_3$) of **3kx**

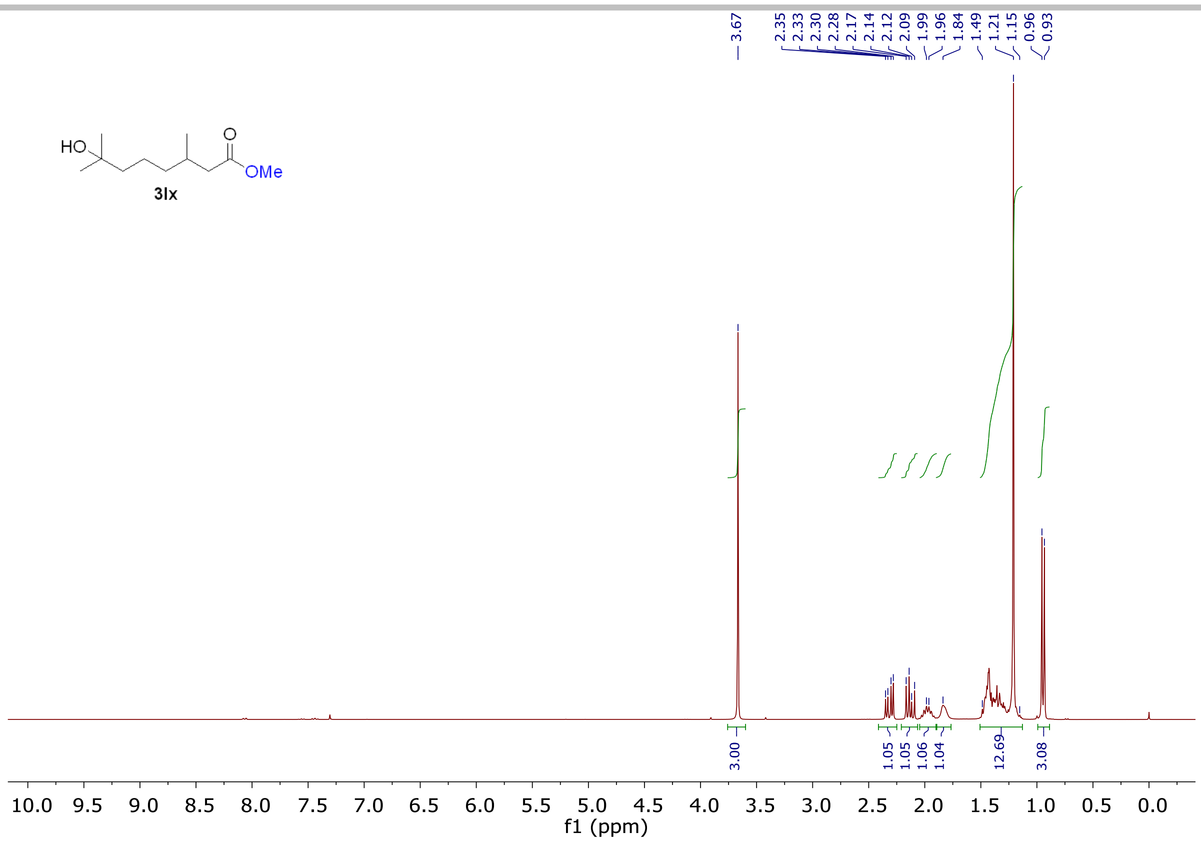

Figure S87. ^{1}H NMR (300 MHz, $CDCl_3$) of **3lx**

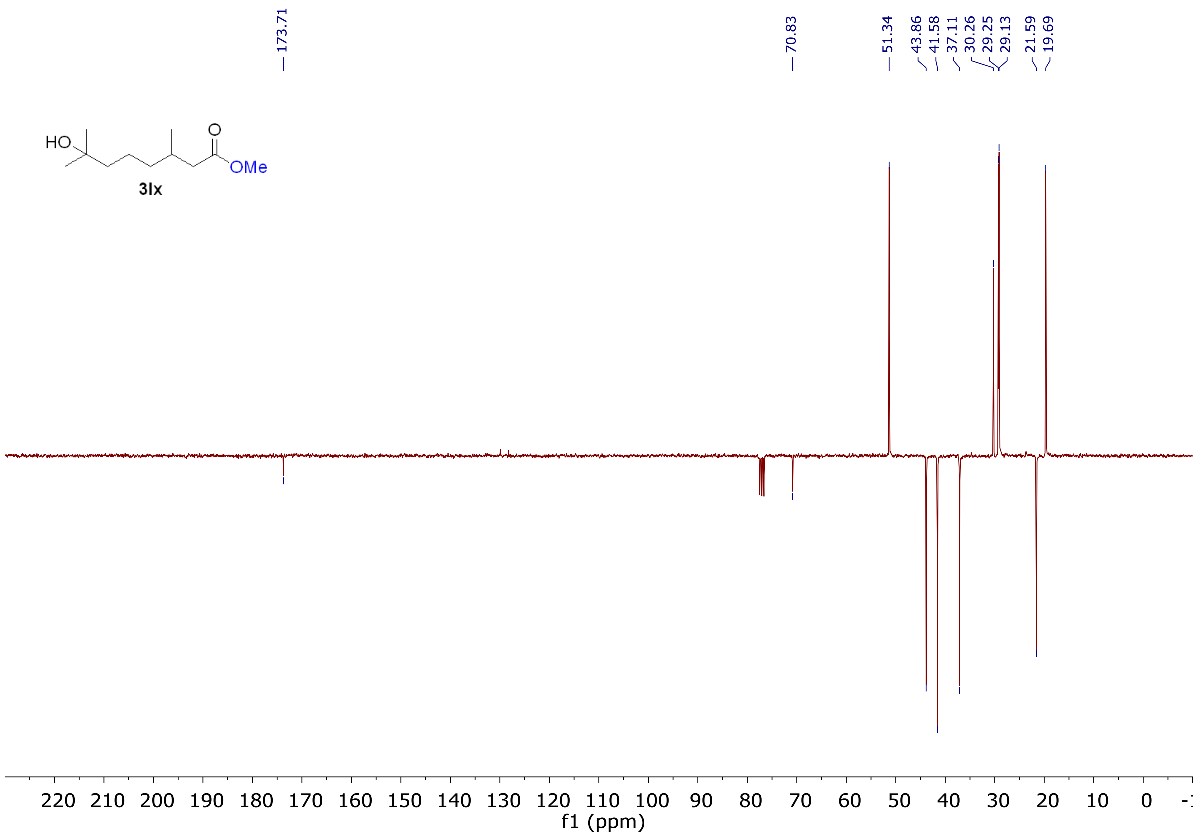

Figure S88. ^{13}C NMR (75 MHz, APT, $CDCl_3$) of **3lx**

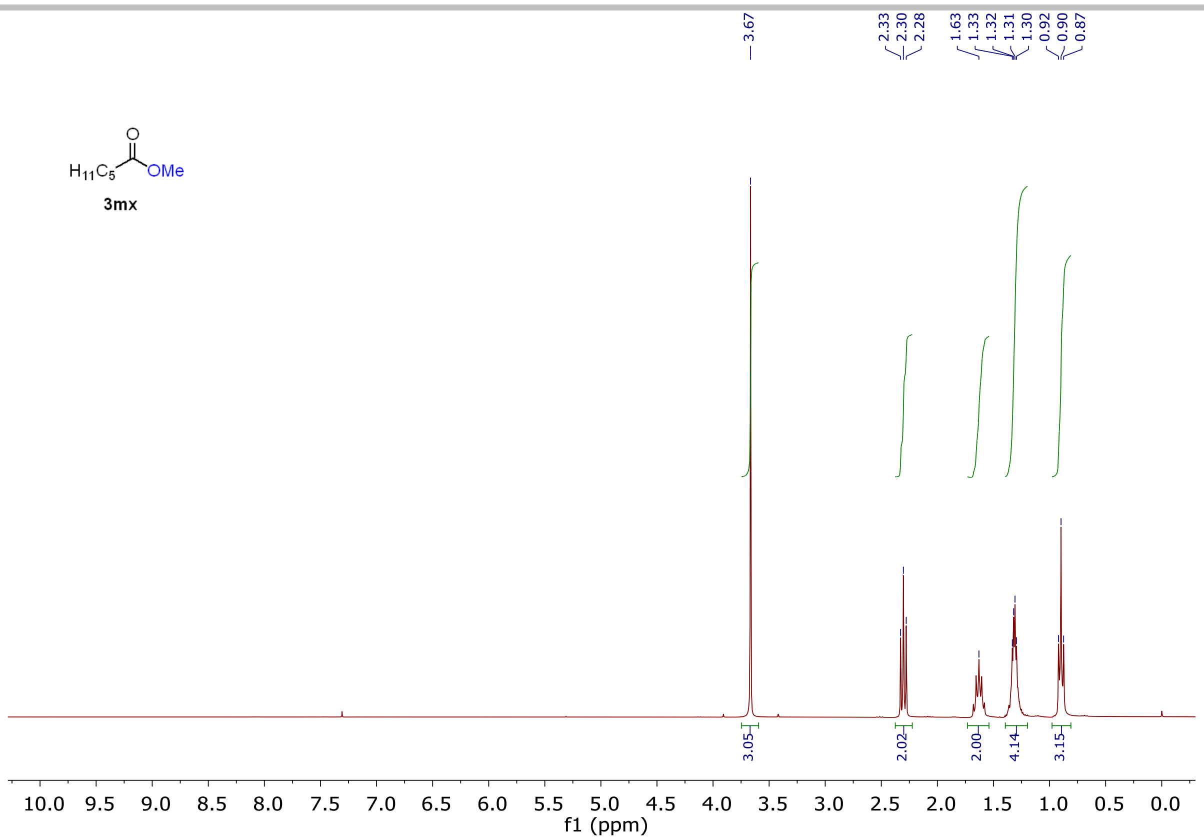

Figure S89. ^{1}H NMR (300 MHz, $CDCl_3$) of **3mx**

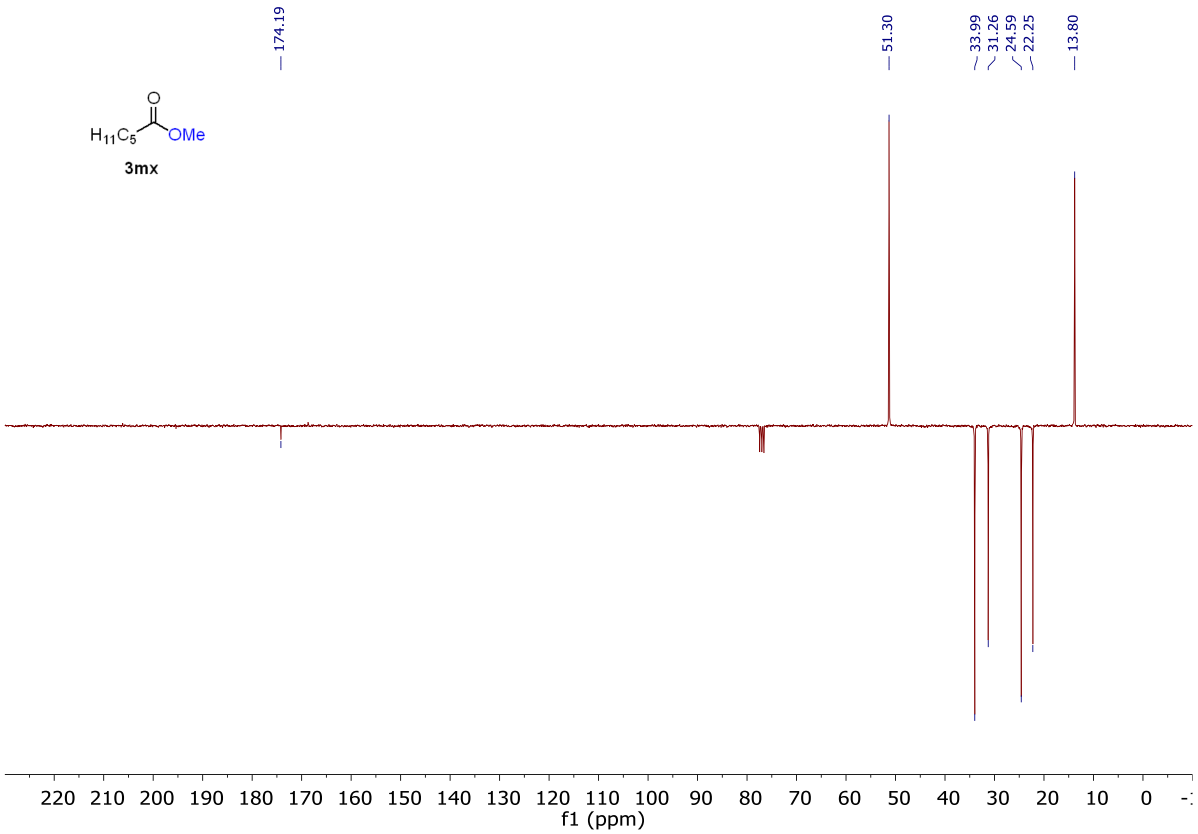

Figure S90. ^{13}C NMR (75 MHz, APT, $CDCl_3$) of **3mx**

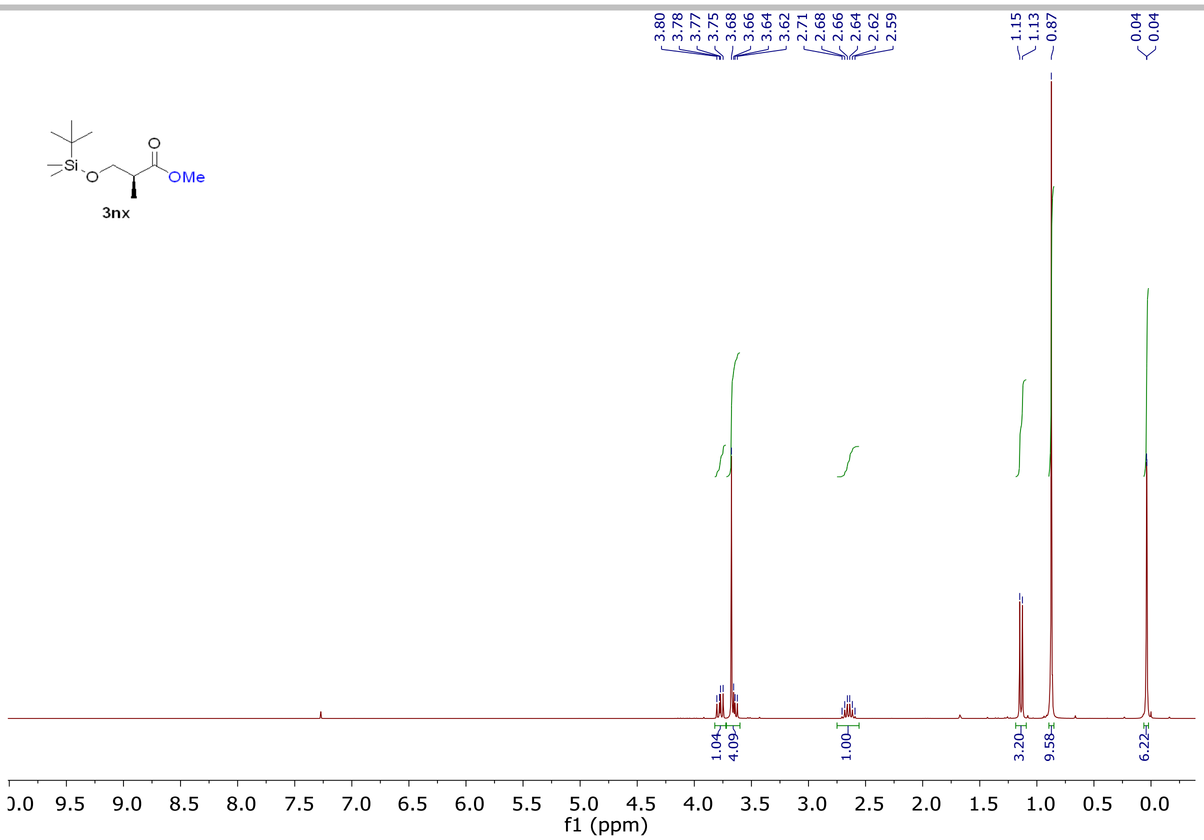

Figure S91. ^{1}H NMR (300 MHz, $CDCl_3$) of **3nx**

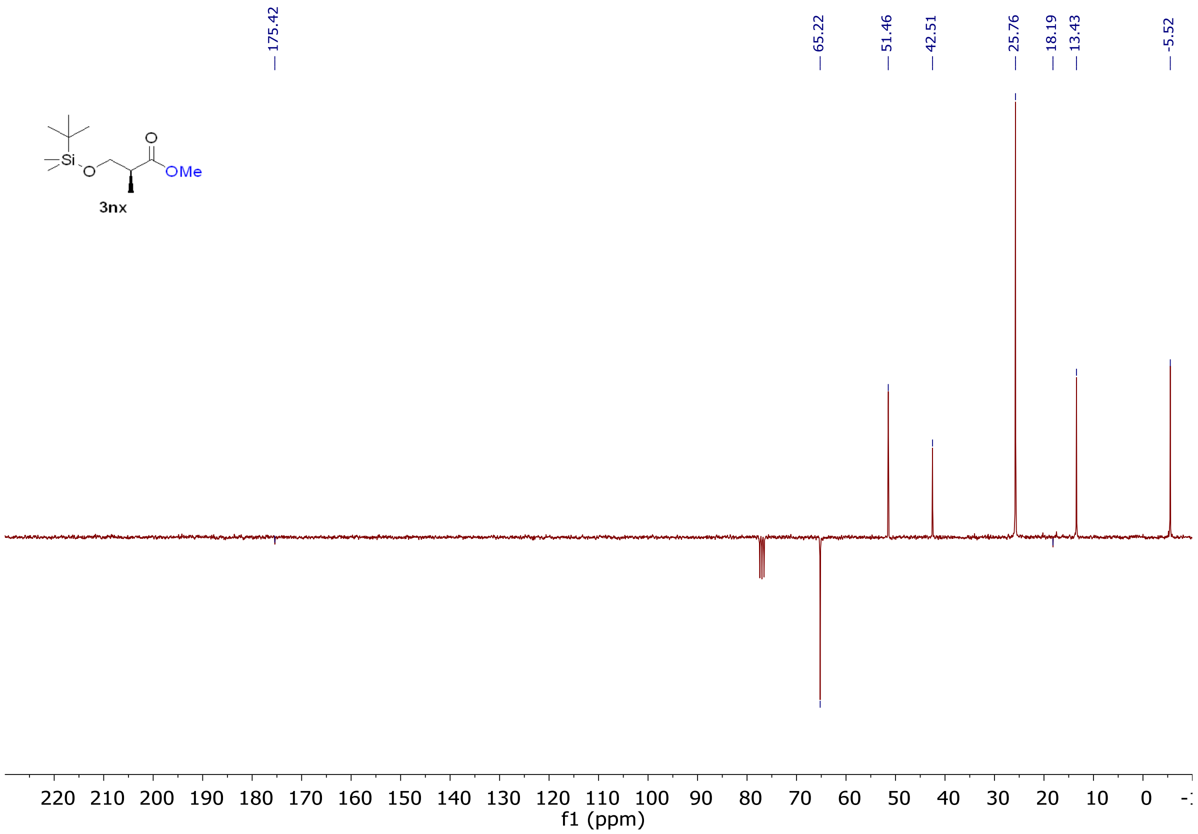

Figure S92. ^{13}C NMR (75 MHz, APT, $CDCl_3$) of **3nx**

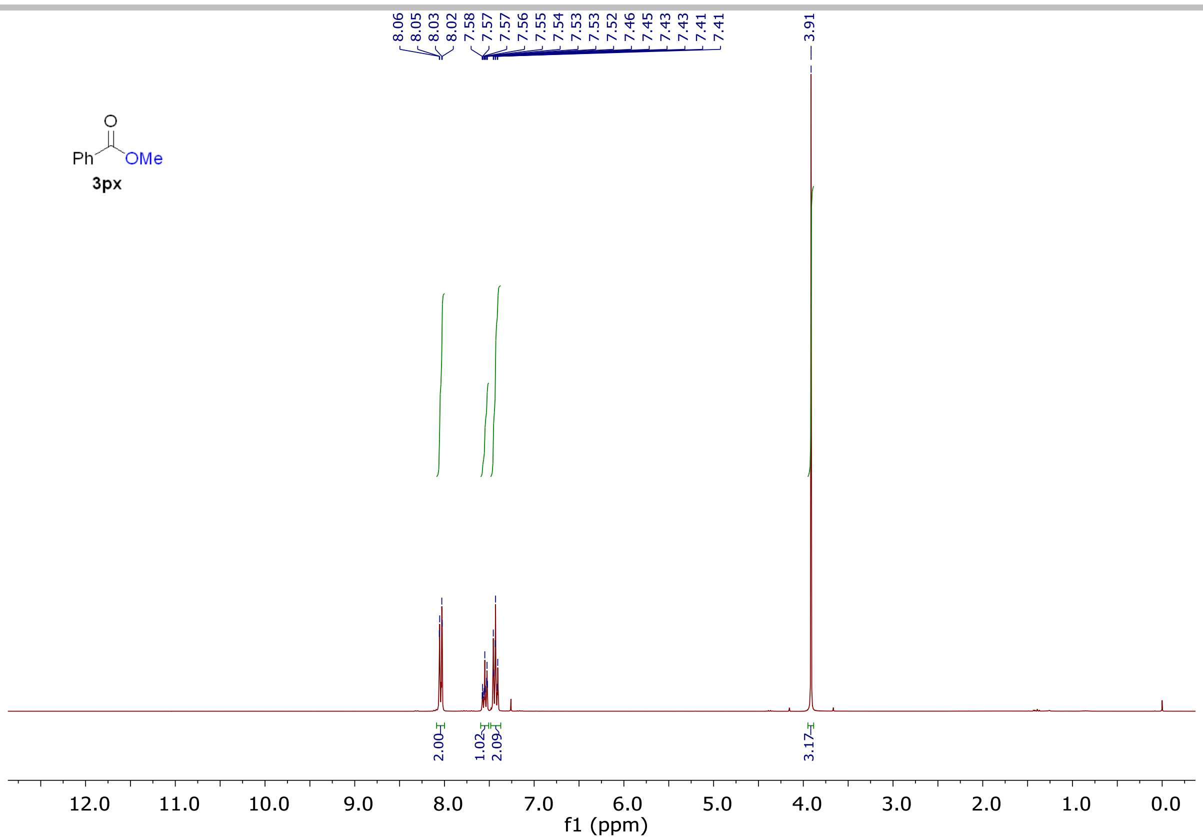

Figure S93. ^{1}H NMR (300 MHz, $CDCl_3$) of **3px**

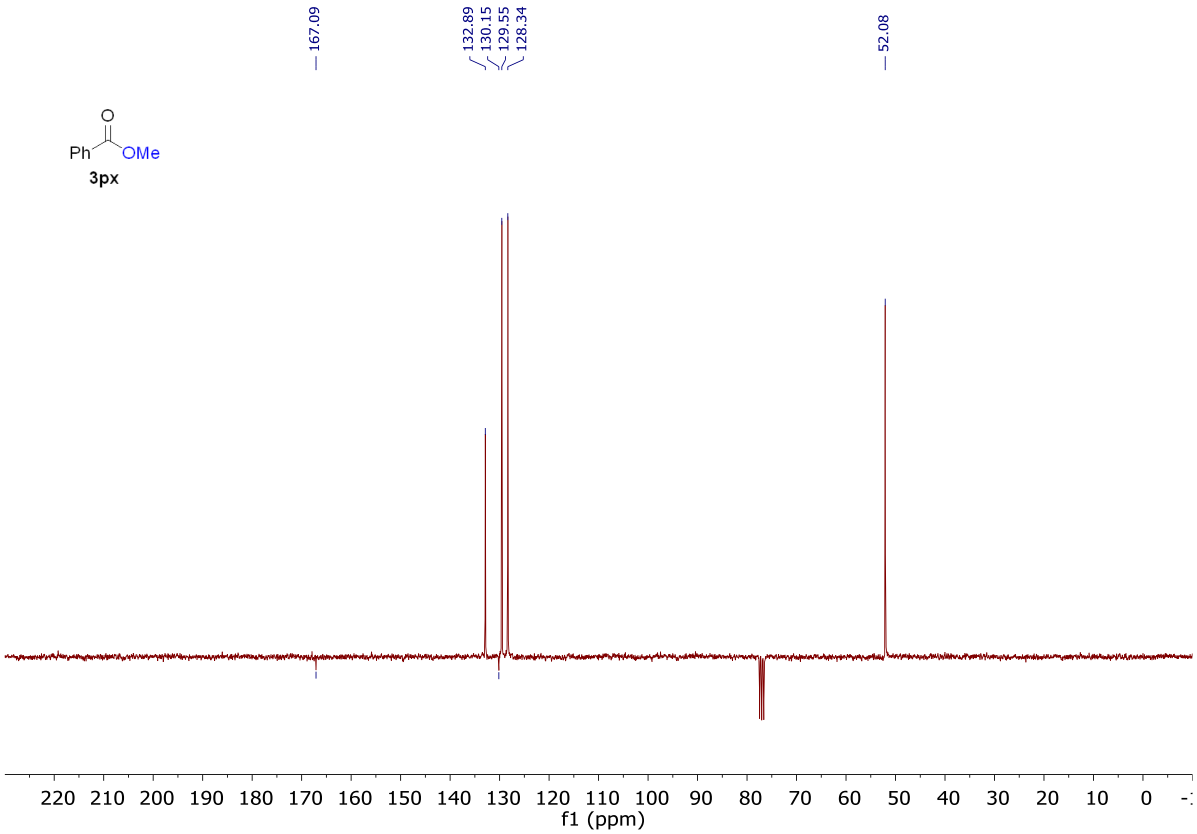

Figure S94. ^{13}C NMR (75 MHz, APT, $CDCl_3$) of **3px**

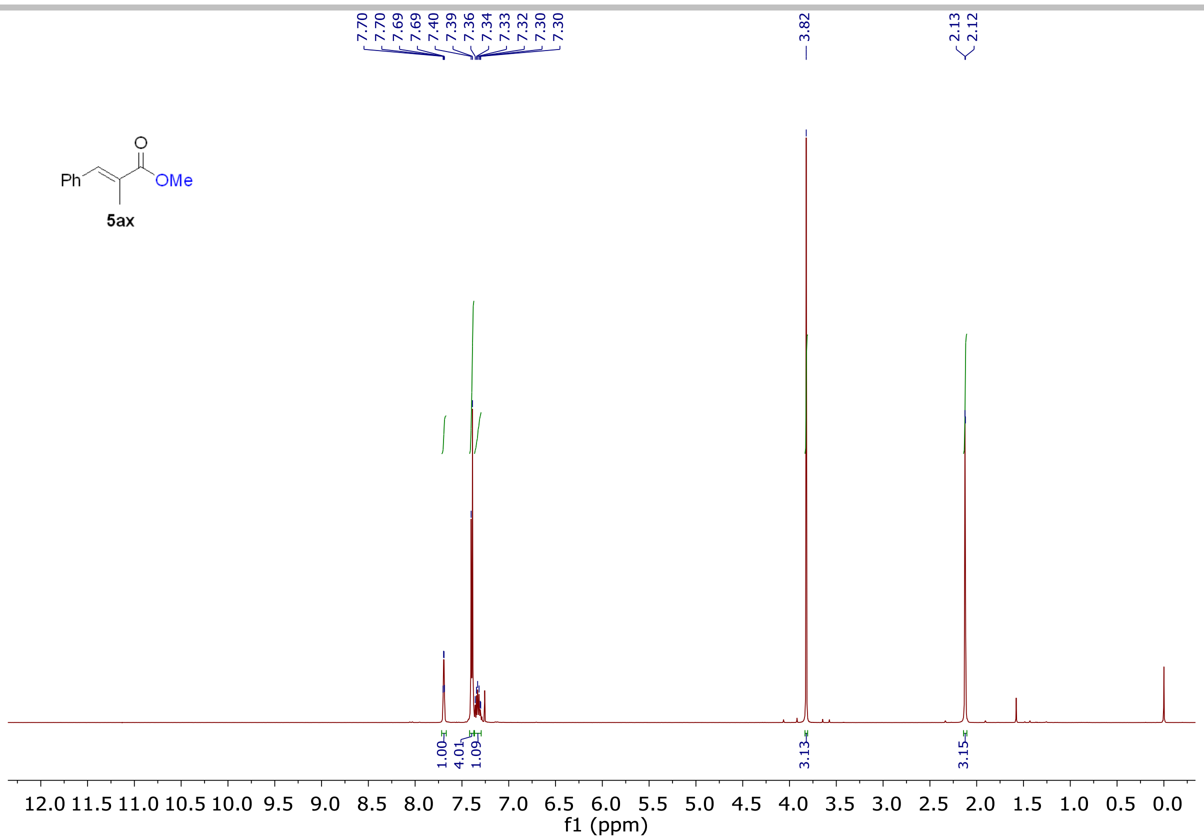

Figure S95. ^{1}H NMR (300 MHz, $CDCl_3$) of **5ax**

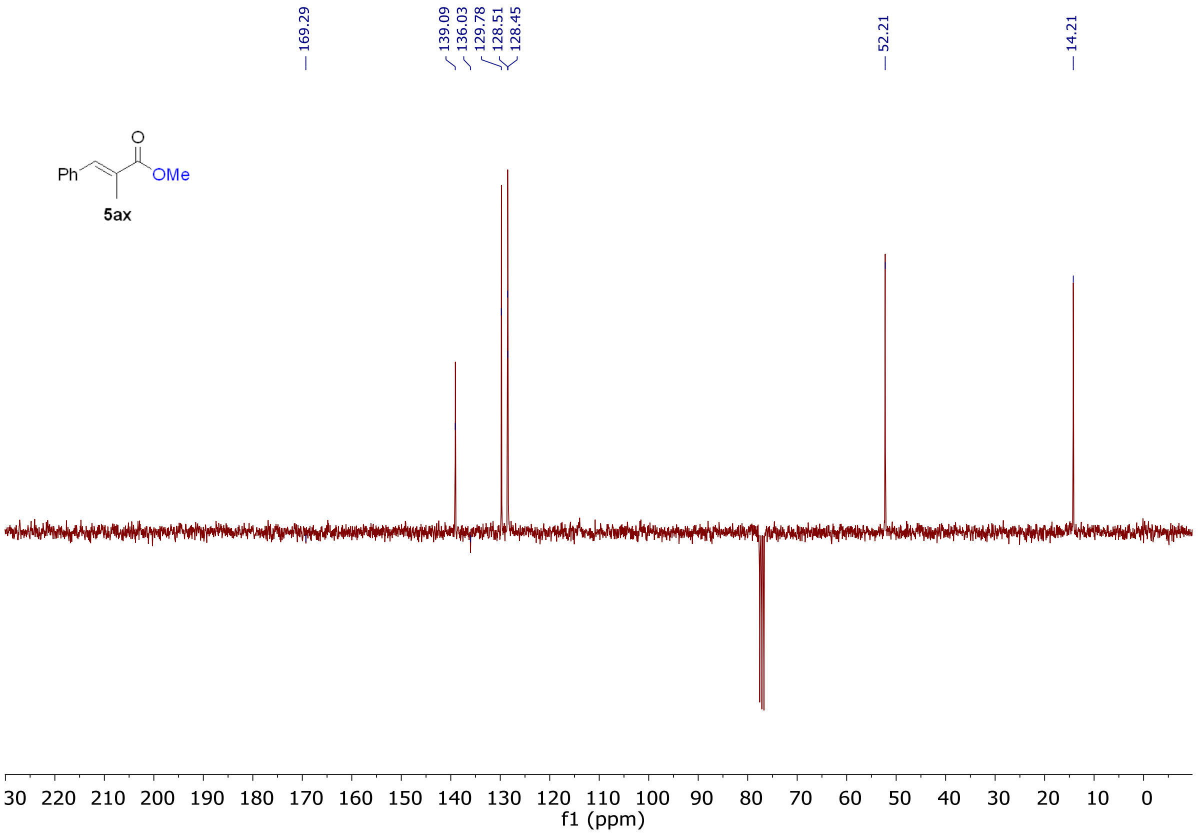

Figure S96. ^{13}C NMR (75 MHz, APT, $CDCl_3$) of **5ax**

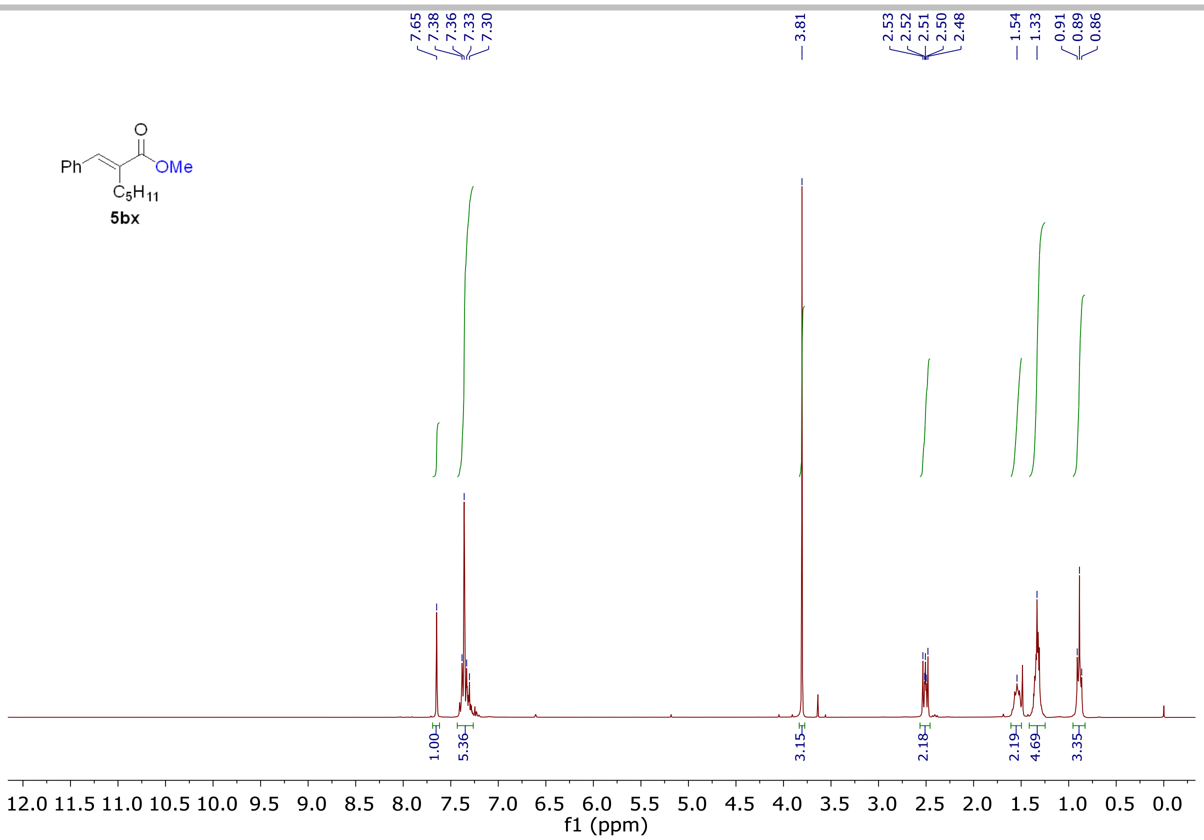

Figure S97. ^{1}H NMR (300 MHz, $CDCl_3$) of **5bx**

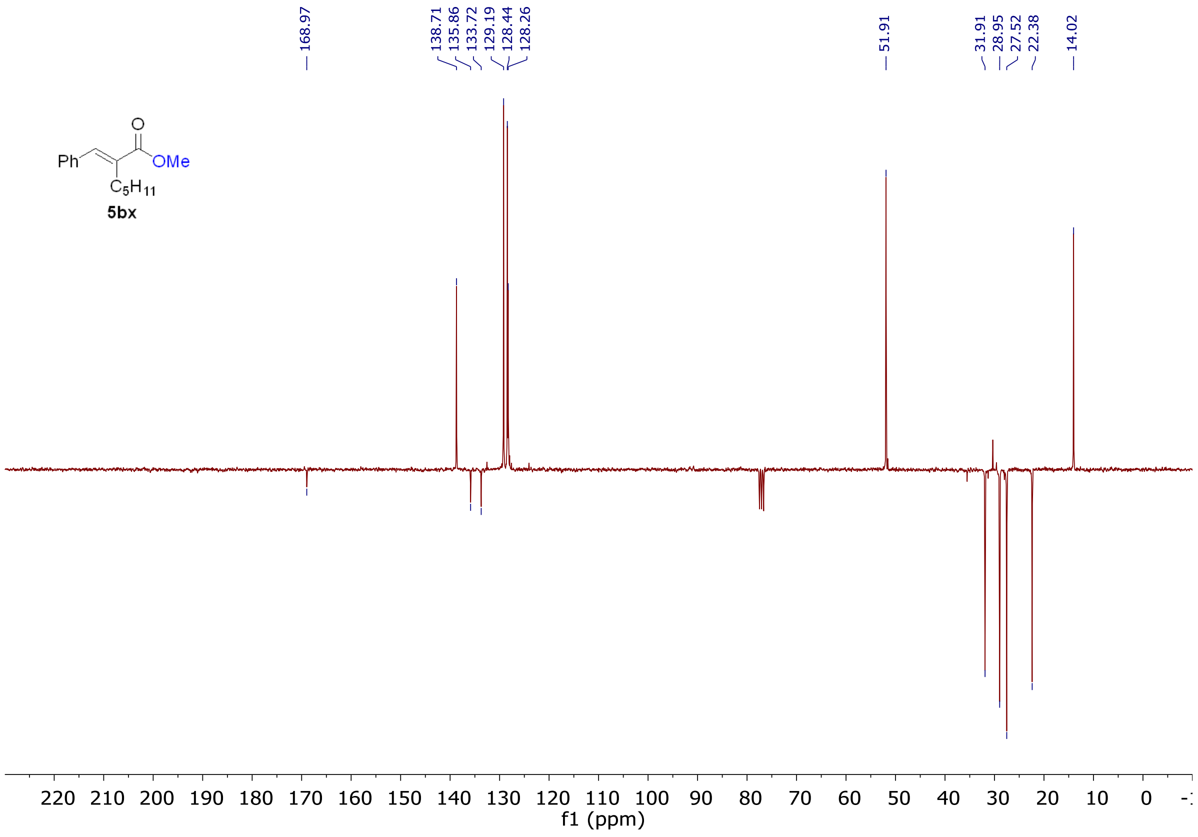

Figure S98. ^{13}C NMR (75 MHz, APT, $CDCl_3$) of **5bx**

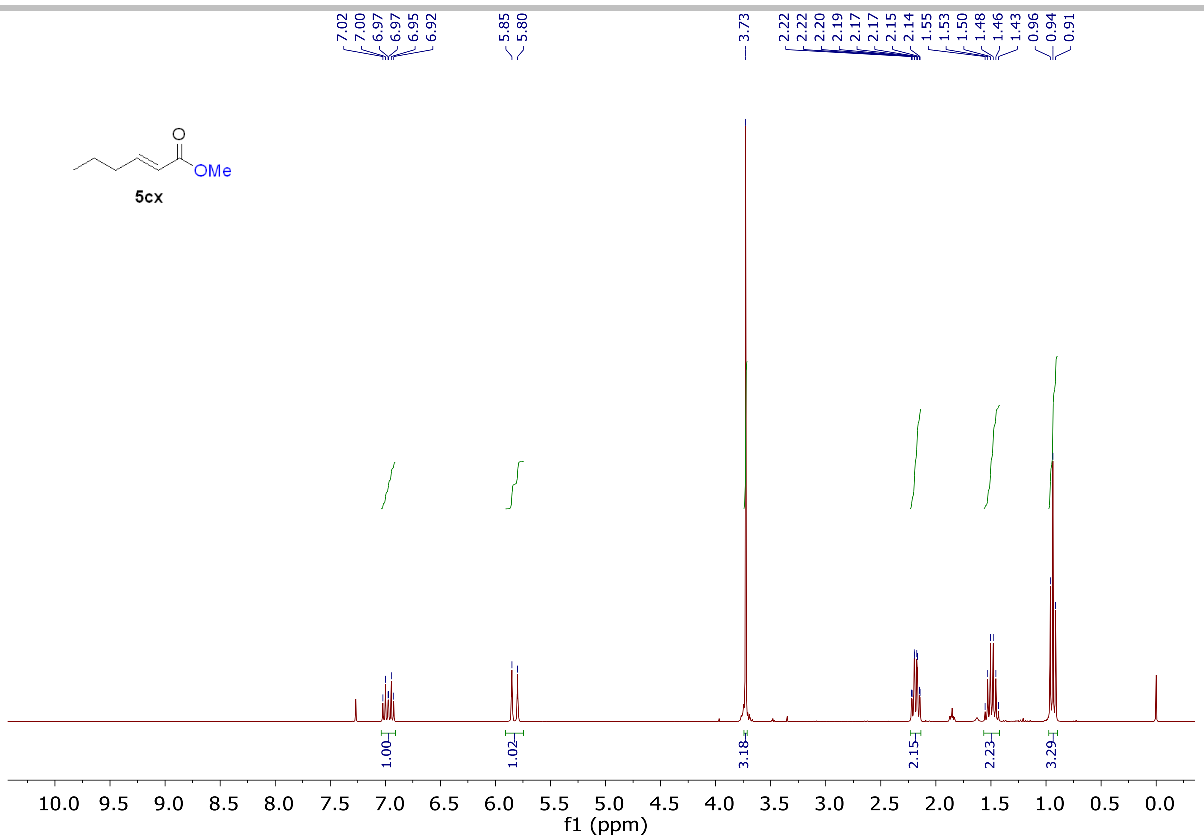

Figure S99. ^{1}H NMR (300 MHz, $CDCl_3$) of **5cx**

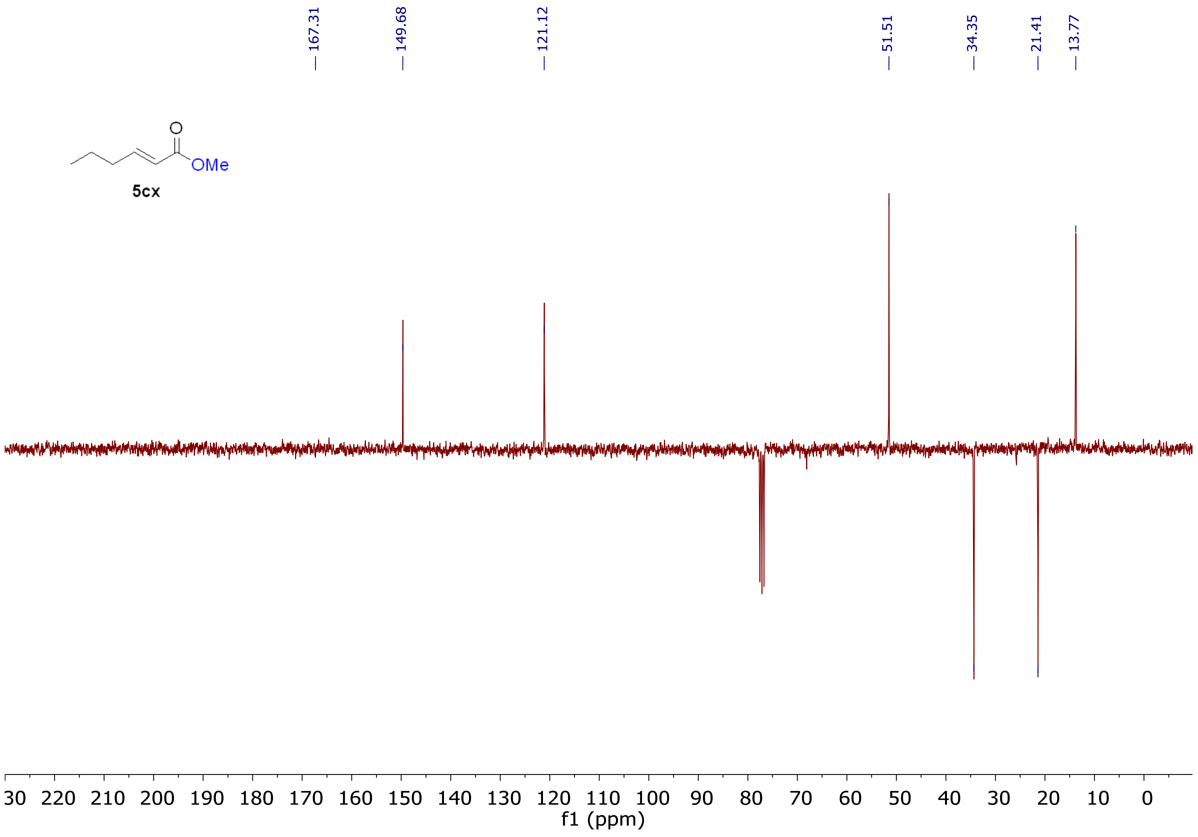

Figure S100. ^{13}C NMR (75 MHz, APT, $CDCl_3$) of **5cx**

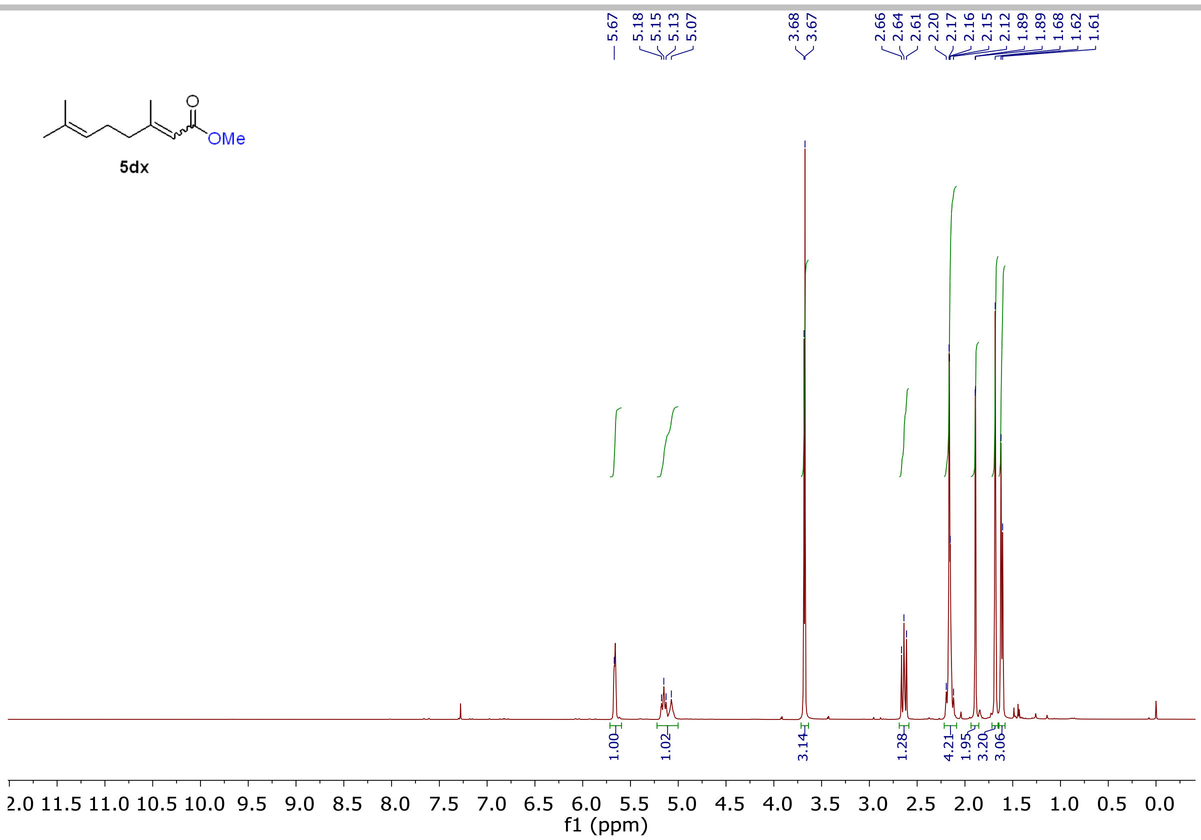

Figure S101. ^{1}H NMR (300 MHz, $CDCl_3$) of **5dx**

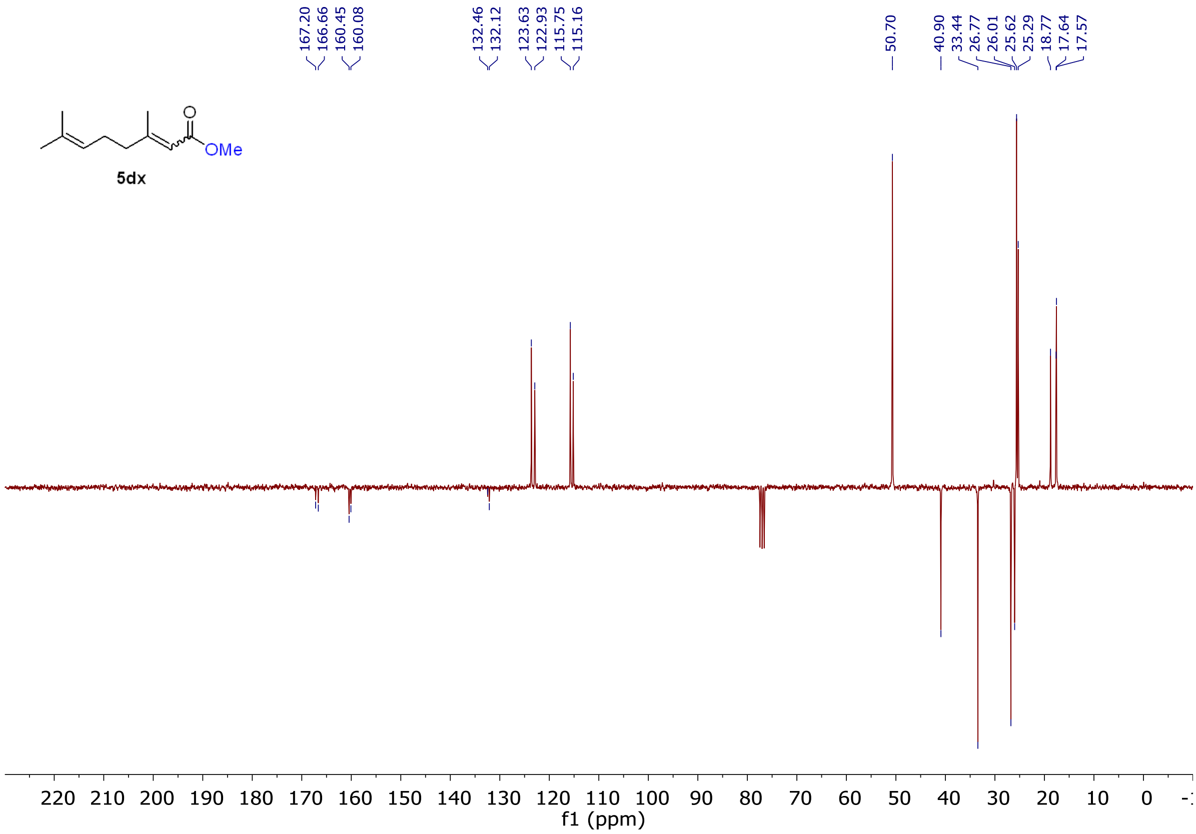

Figure S102. ^{13}C NMR (75 MHz, APT, $CDCl_3$) of **5dx**

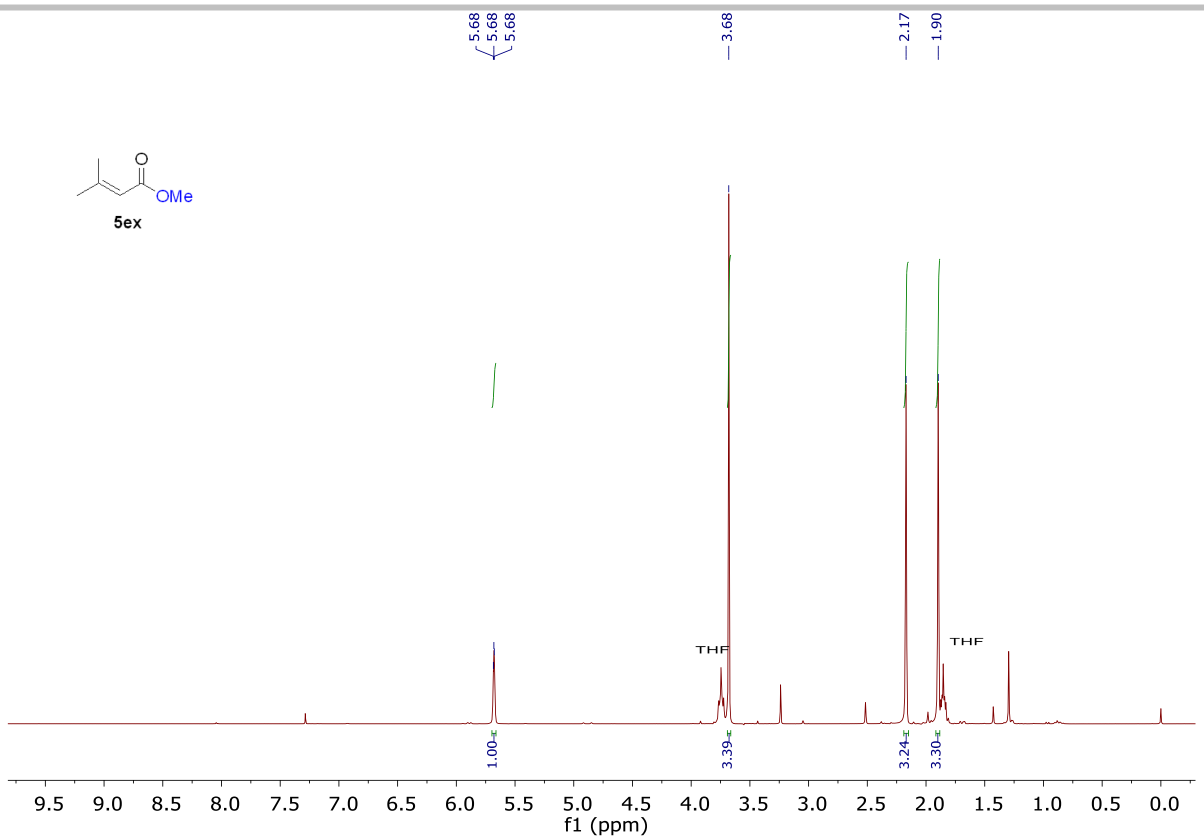

Figure S103. ^{1}H NMR (300 MHz, $CDCl_3$) of **5ex**

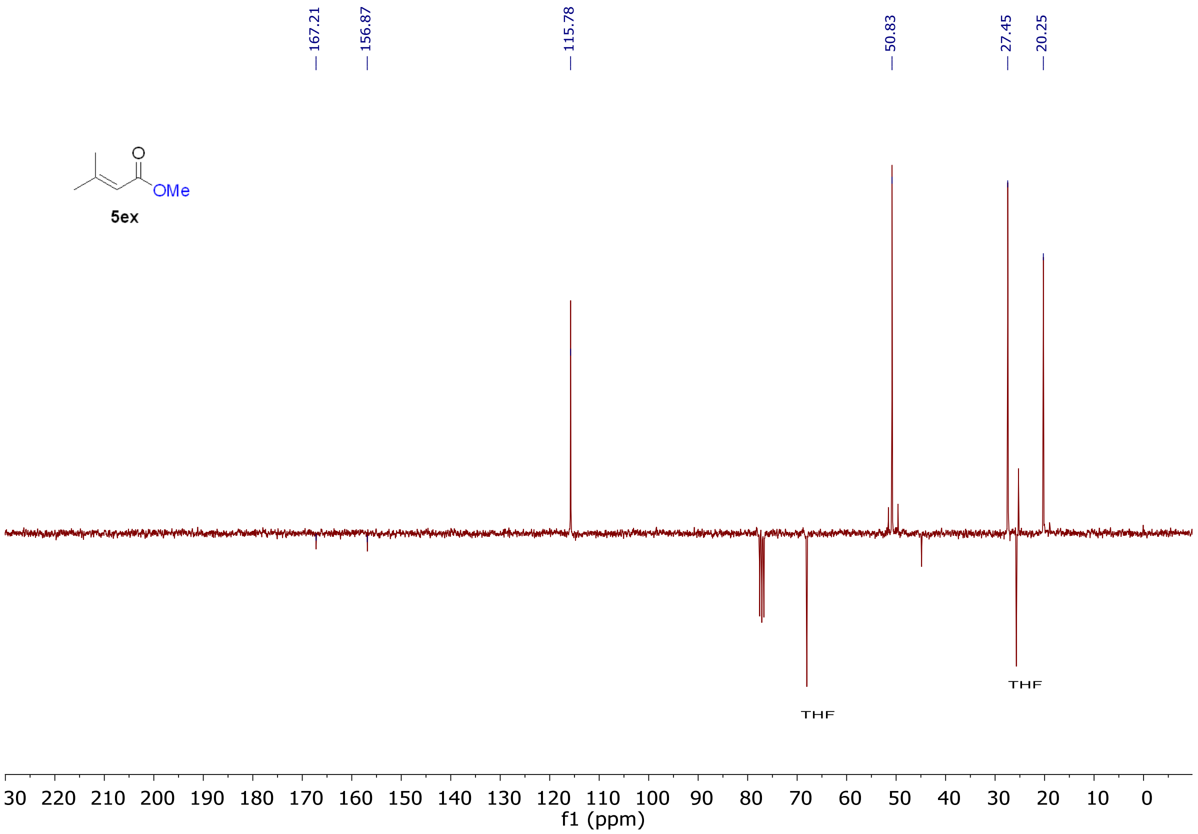

Figure S104. ^{13}C NMR (75 MHz, APT, $CDCl_3$) of **5ex**

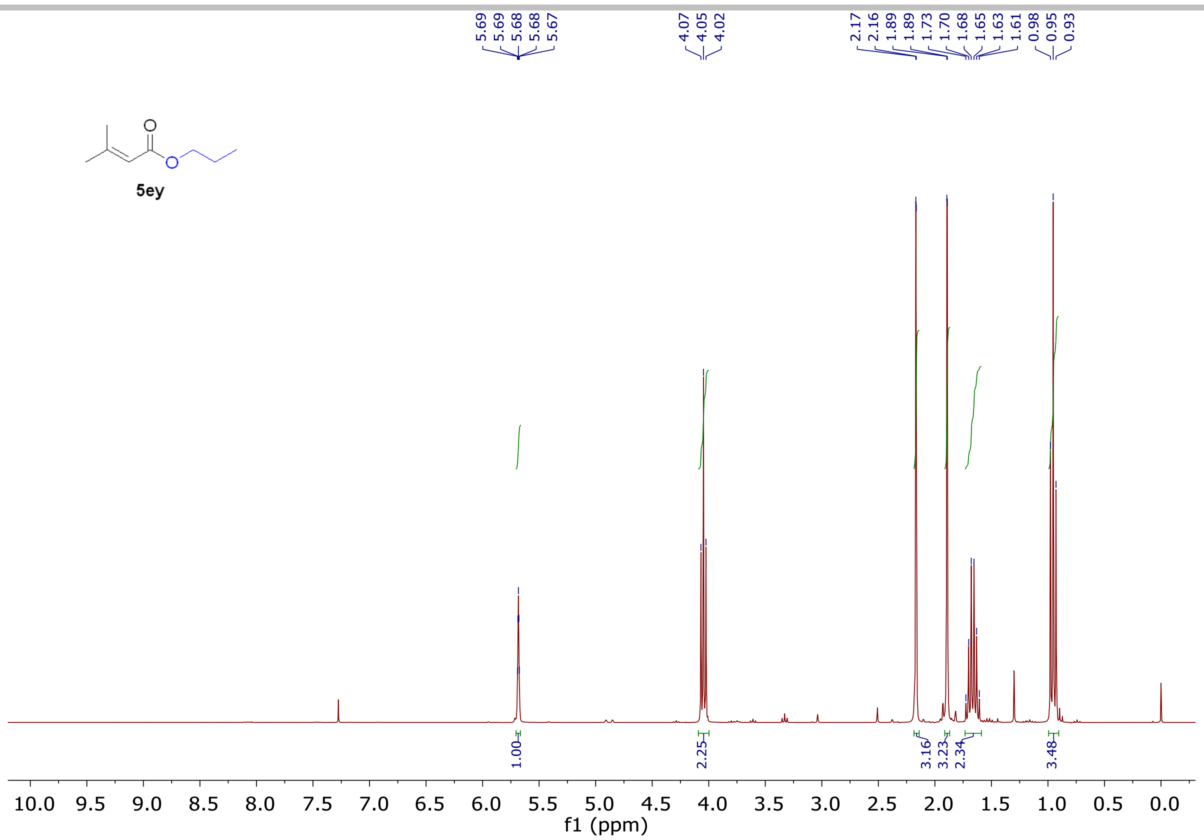

Figure S105. ^{1}H NMR (300 MHz, $CDCl_3$) of **5ey**

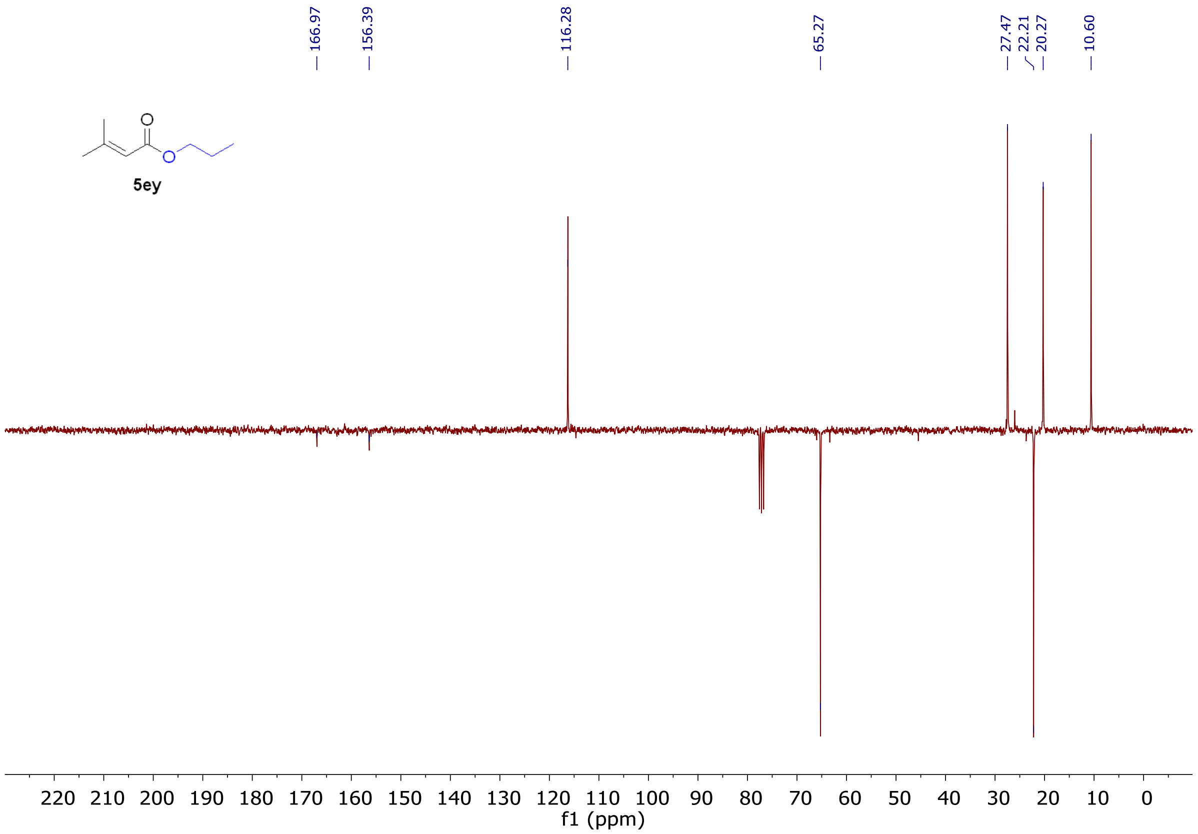

Figure S106. ^{13}C NMR (75 MHz, APT, $CDCl_3$) of **5ey**

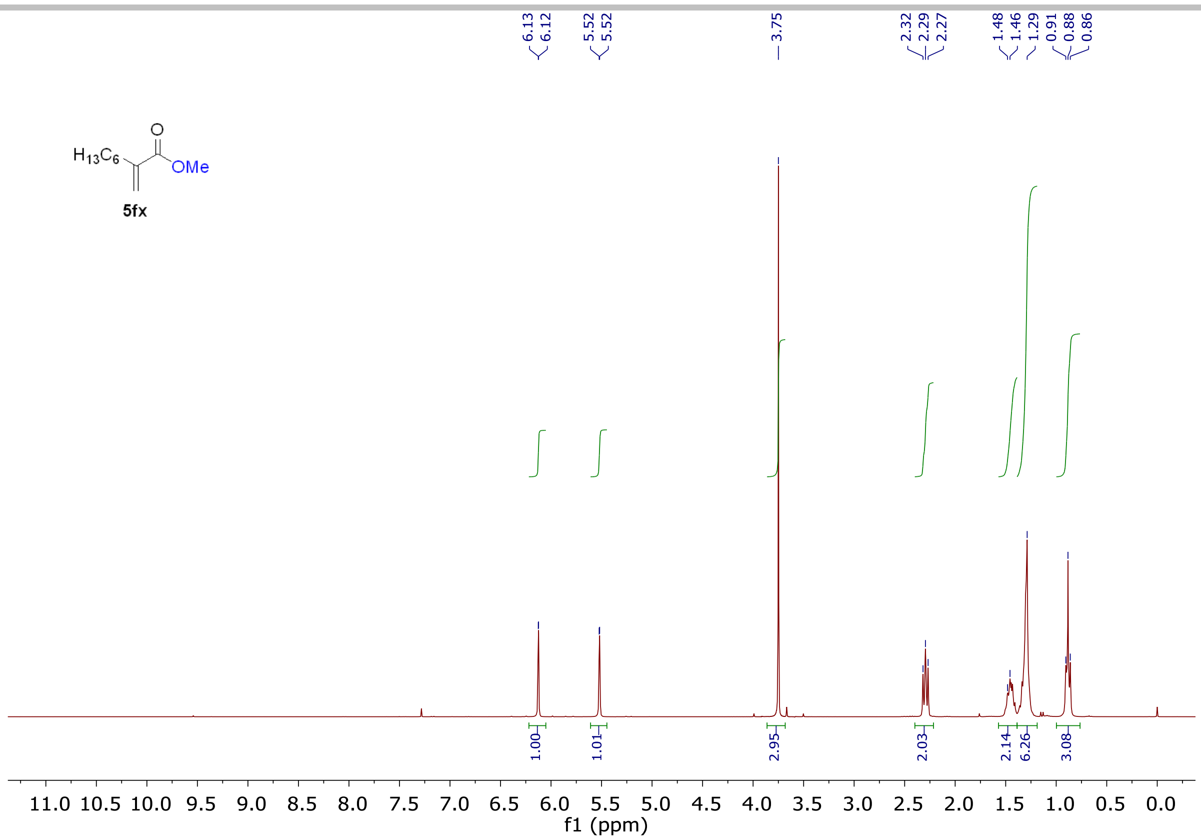

Figure S107. ^{1}H NMR (300 MHz, $CDCl_3$) of **5fx**

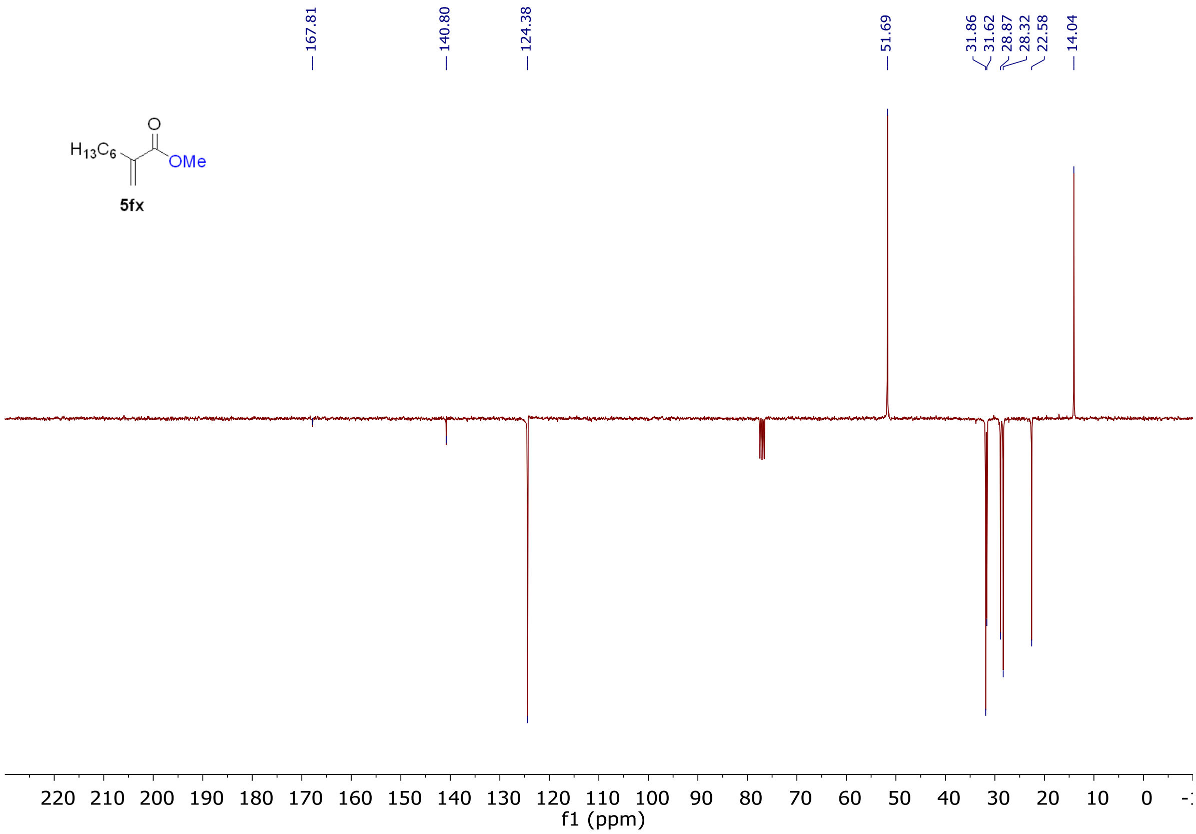

Figure S108. ^{13}C NMR (75 MHz, APT, $CDCl_3$) of **5fx**

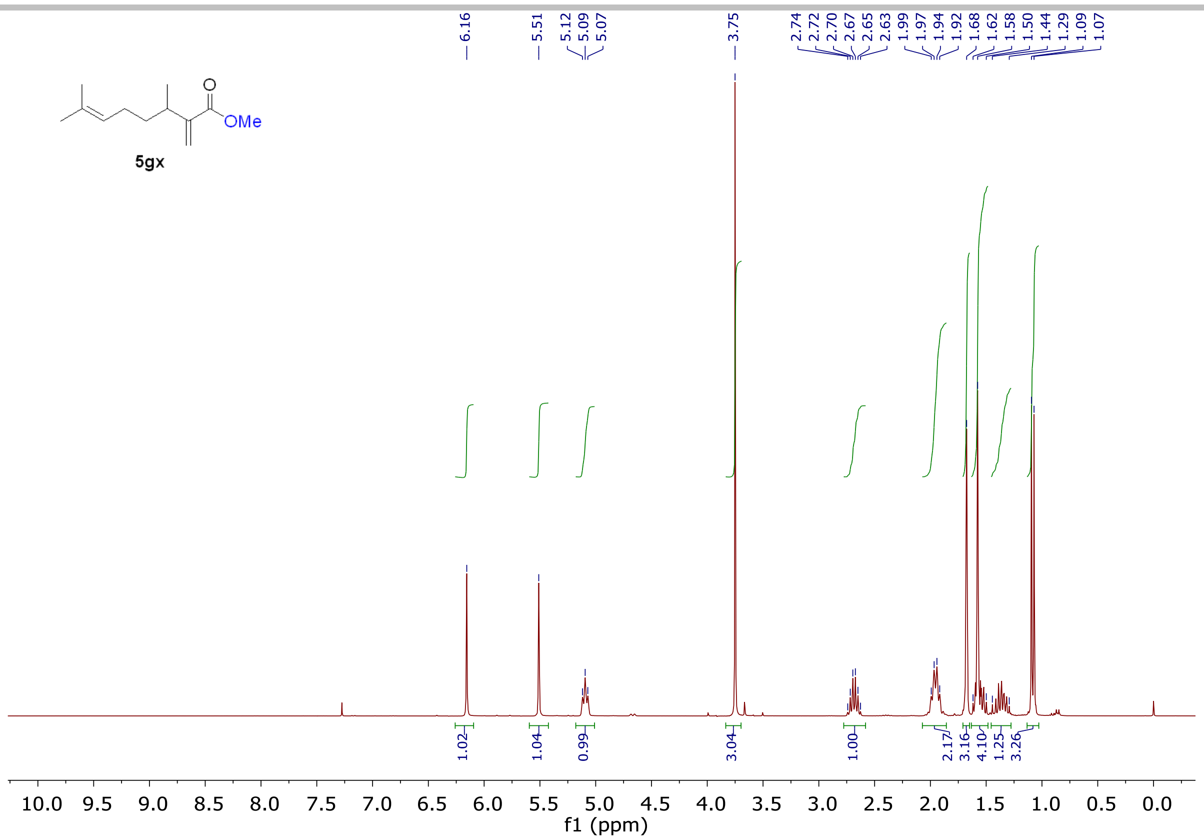

Figure S109. ^{1}H NMR (300 MHz, $CDCl_3$) of **5gx**

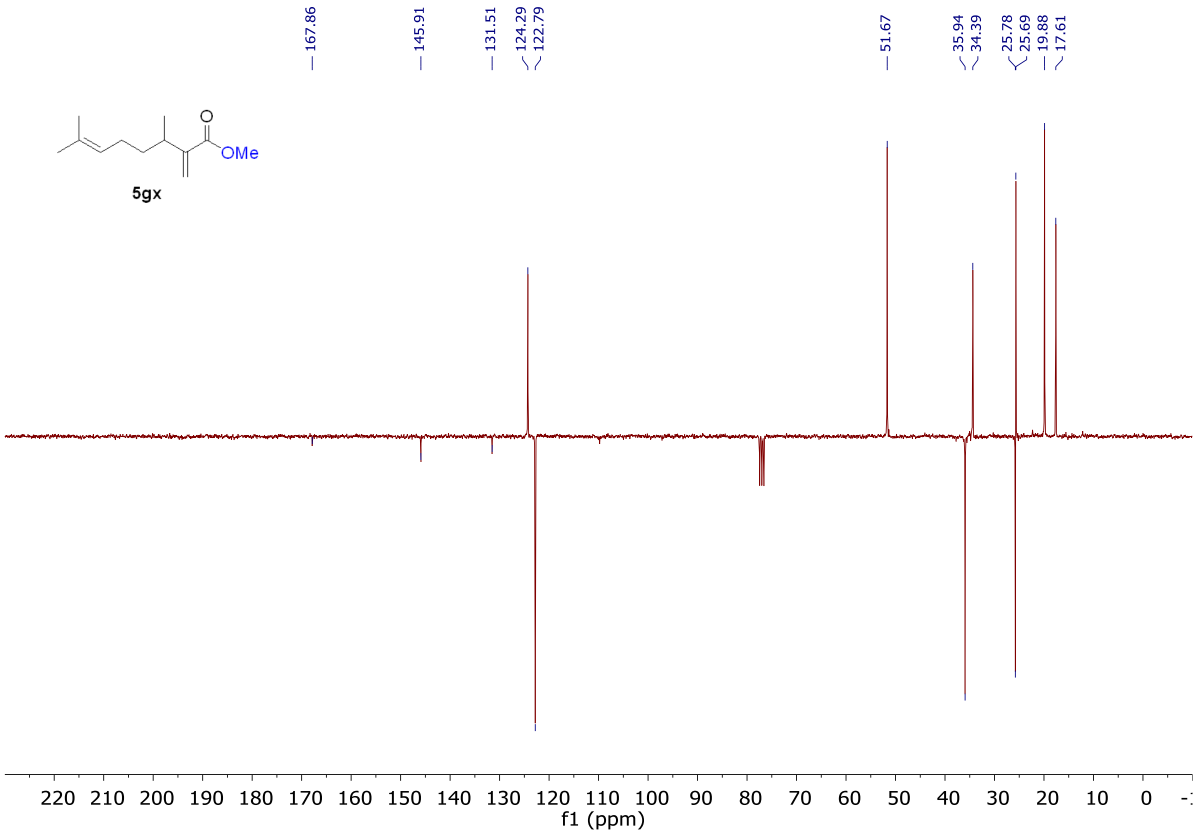

Figure S110. ^{13}C NMR (75 MHz, APT, $CDCl_3$) of **5gx**

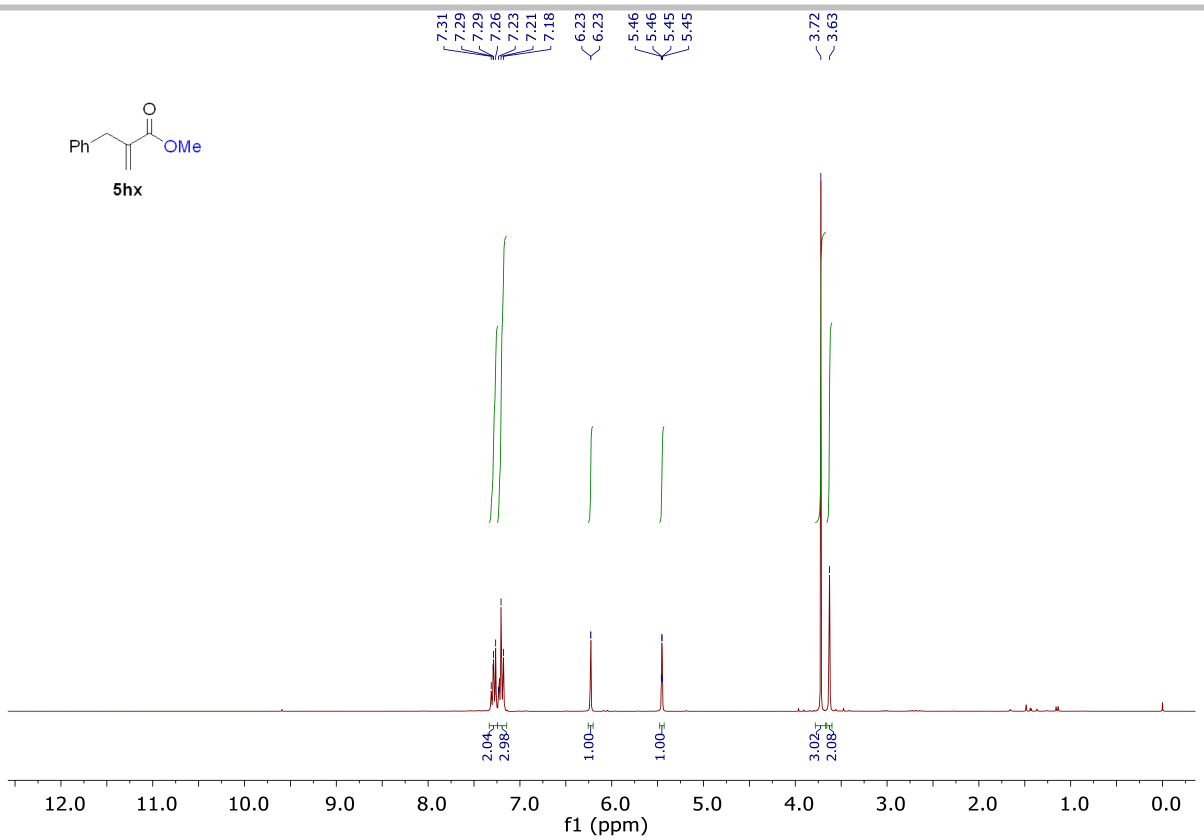

Figure S111. ^{1}H NMR (300 MHz, $CDCl_3$) of **5hx**

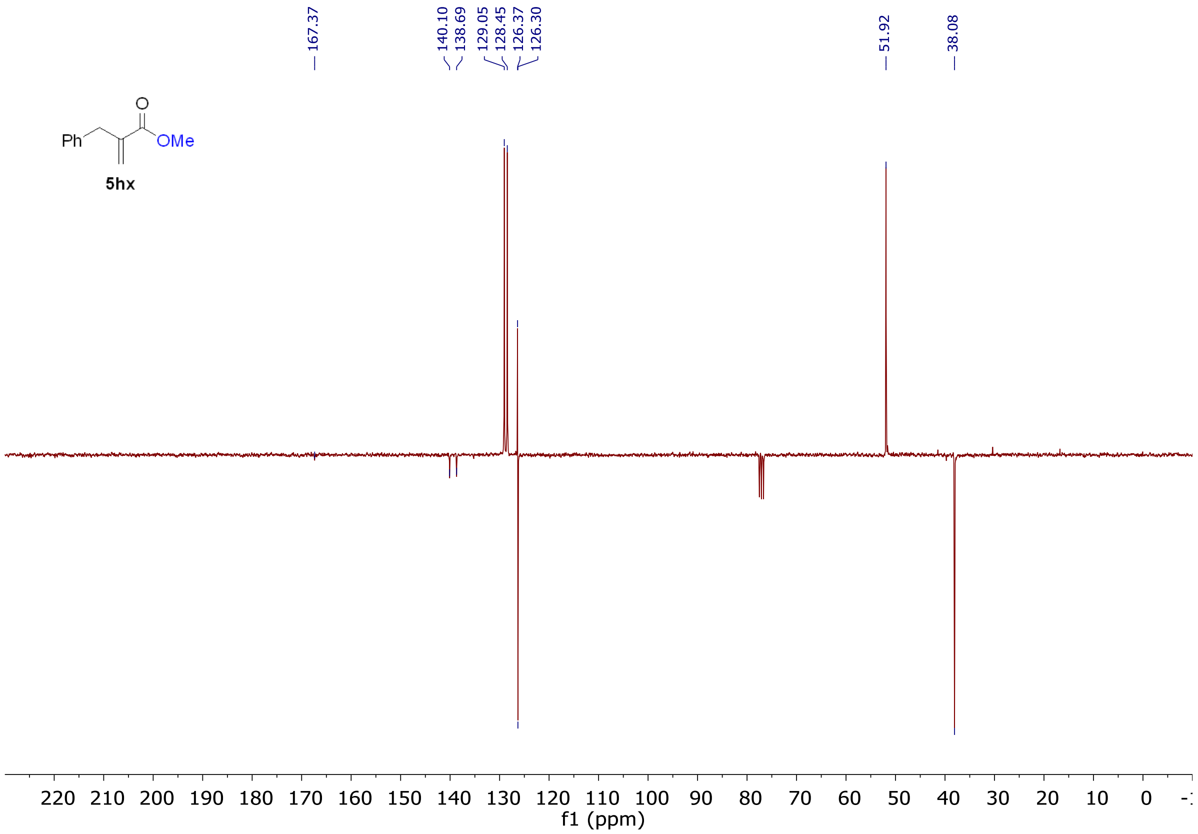

Figure S112. ^{13}C NMR (75 MHz, APT, $CDCl_3$) of **5hx**

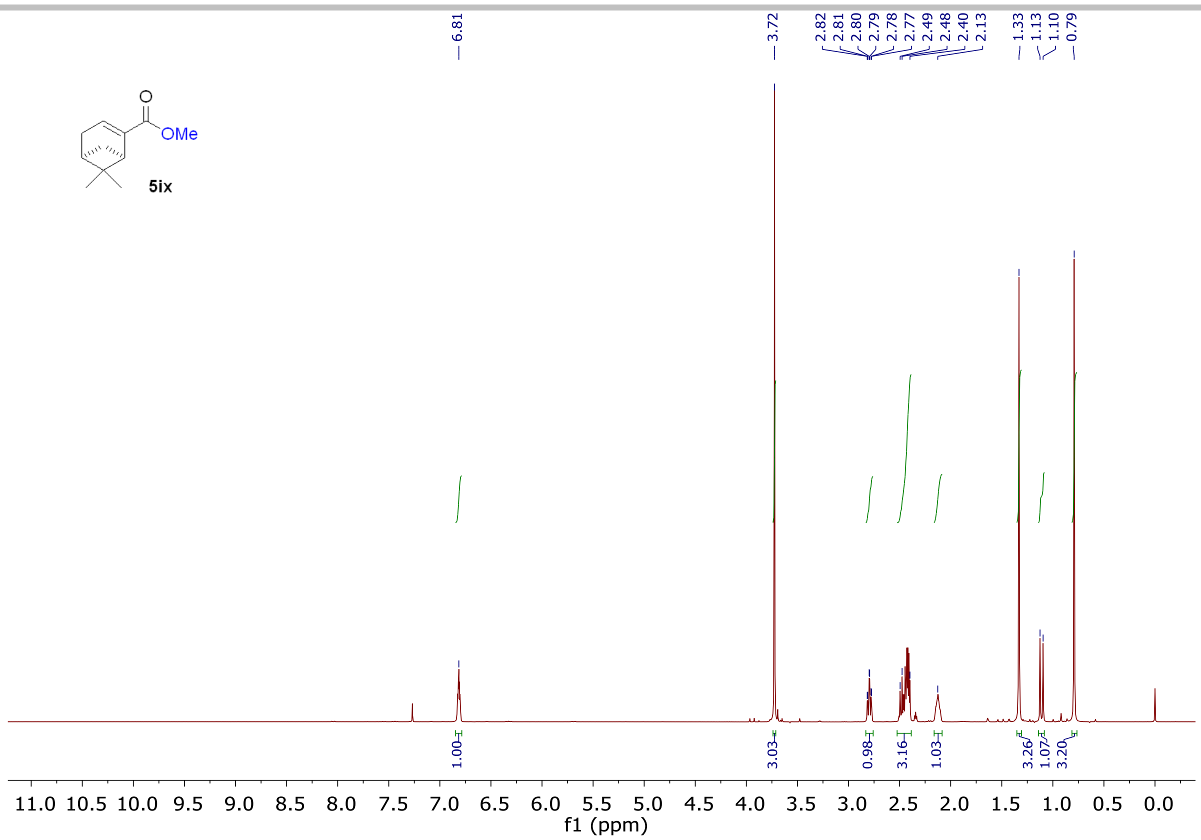

Figure S113. ^{1}H NMR (300 MHz, $CDCl_3$) of **5ix**

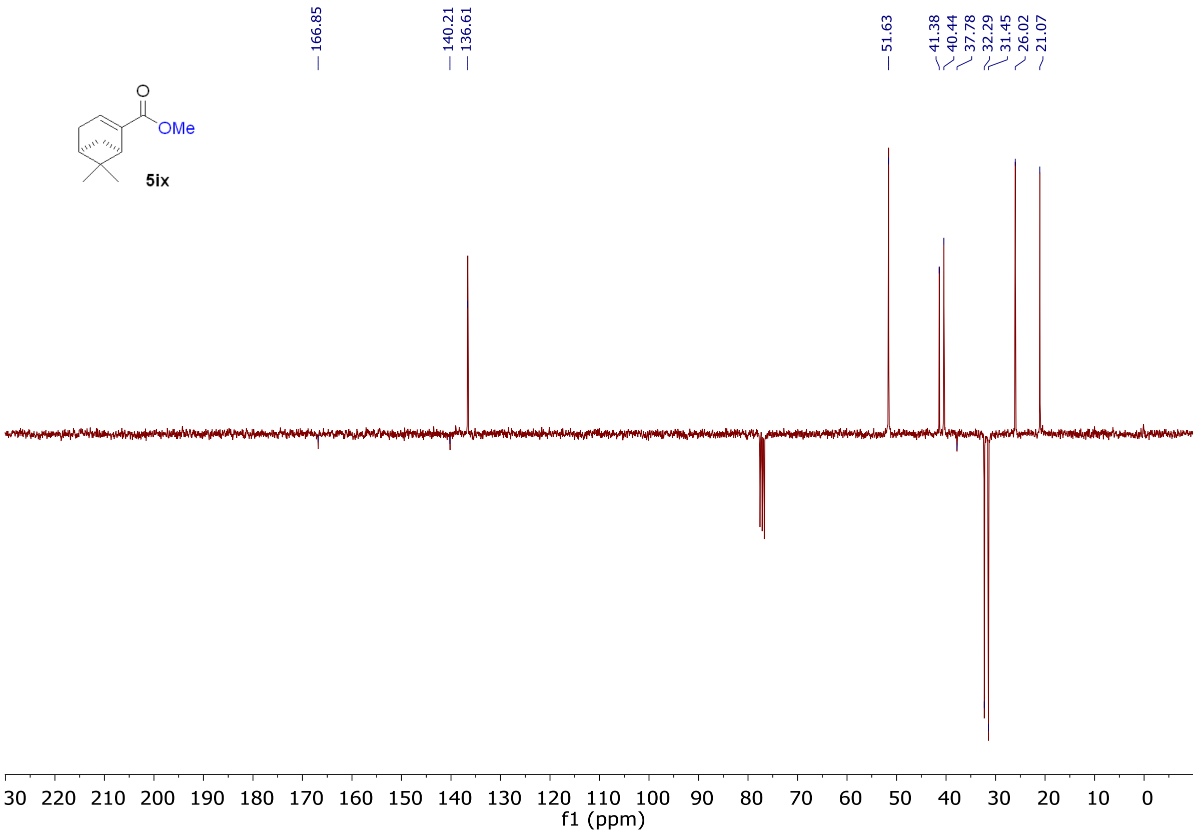

Figure S114. ^{13}C NMR (75 MHz, APT, $CDCl_3$) of **5ix**

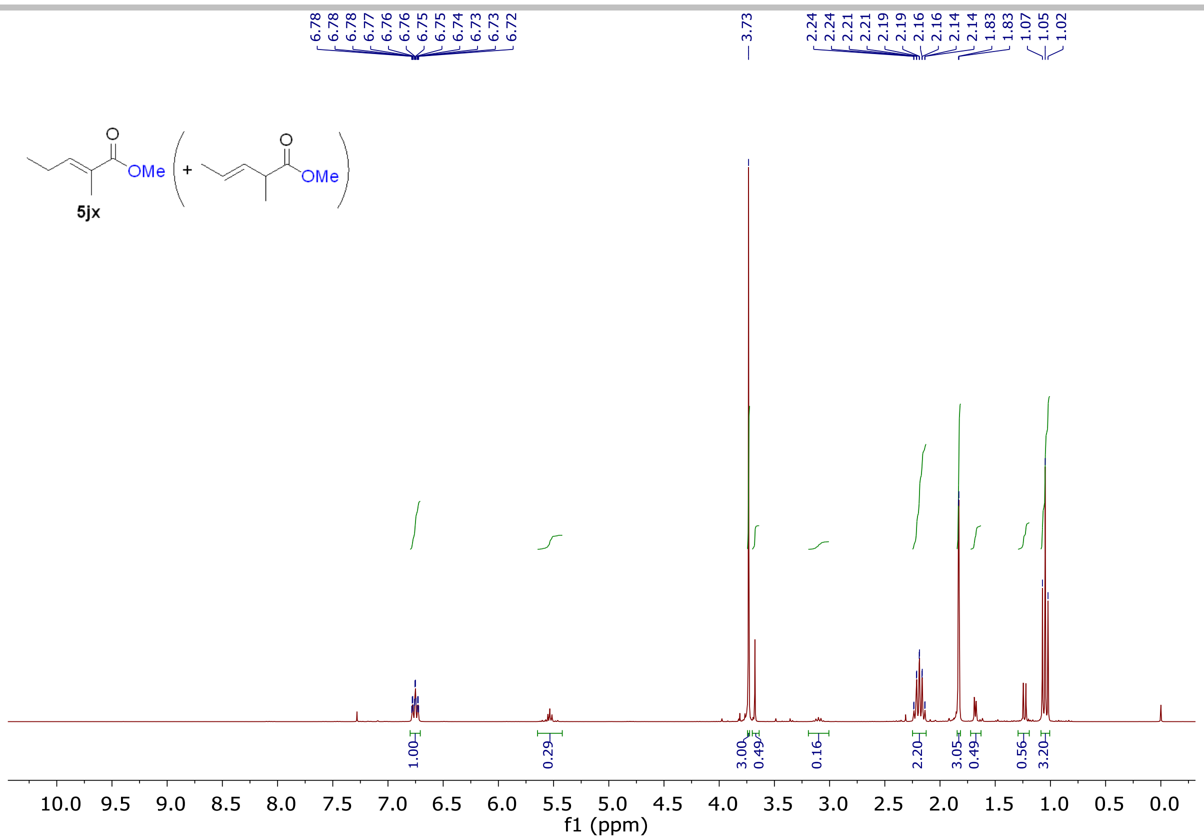

Figure S115. ^{1}H NMR (300 MHz, $CDCl_3$) of **5jx**

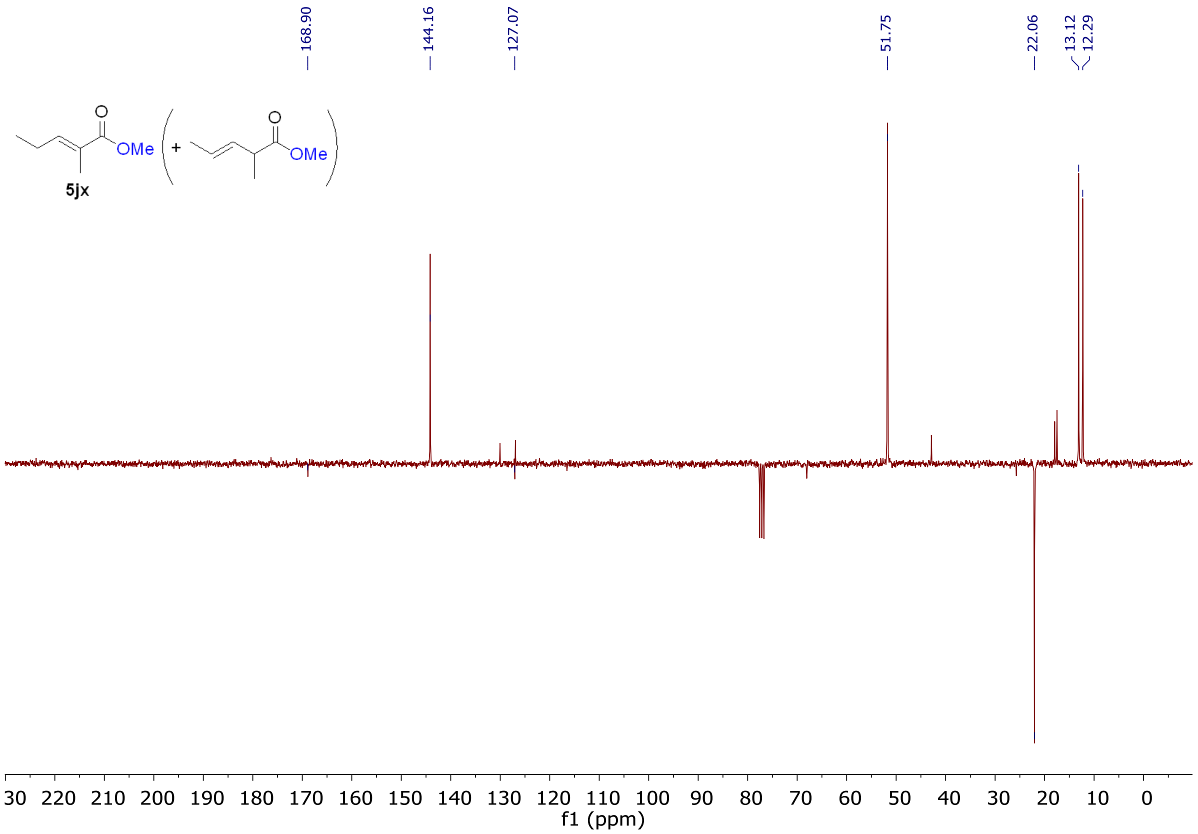

Figure S116. ^{13}C NMR (75 MHz, APT, $CDCl_3$) of **5jx**

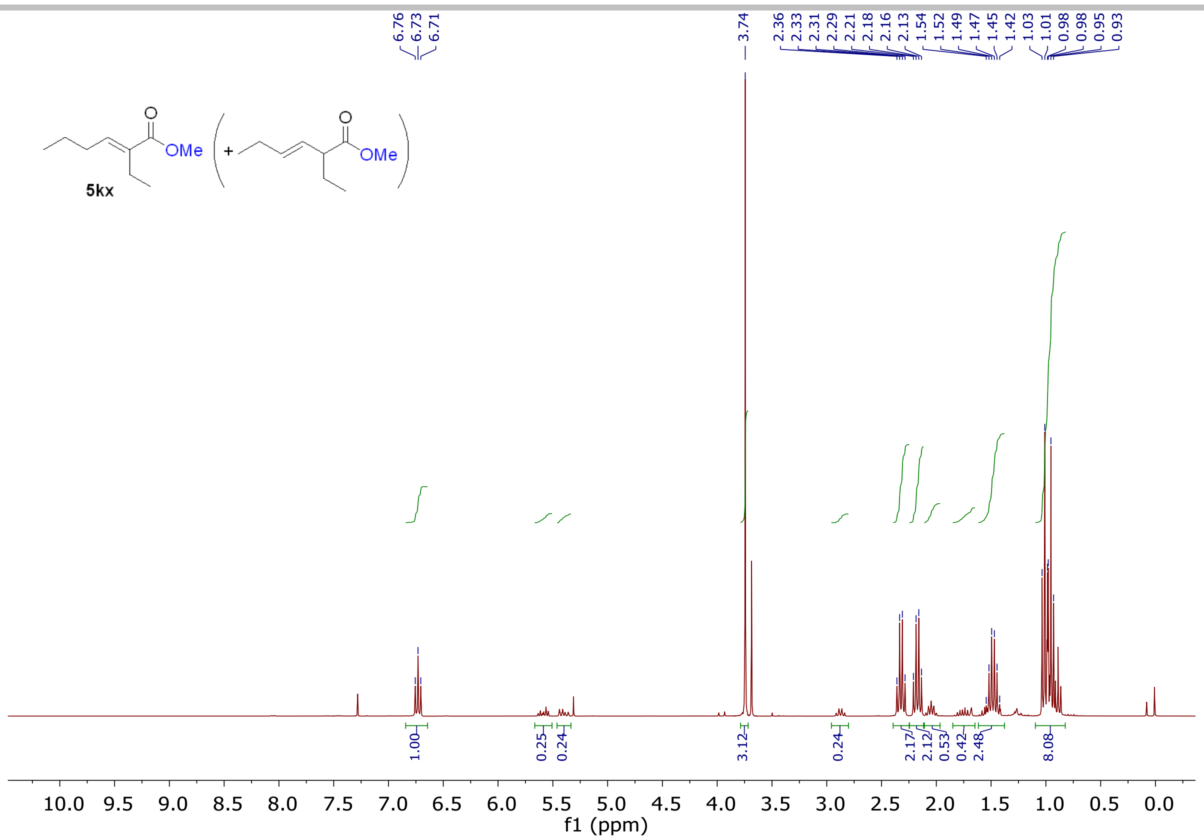

Figure S117. ^{1}H NMR (300 MHz, $CDCl_3$) of **5kx**

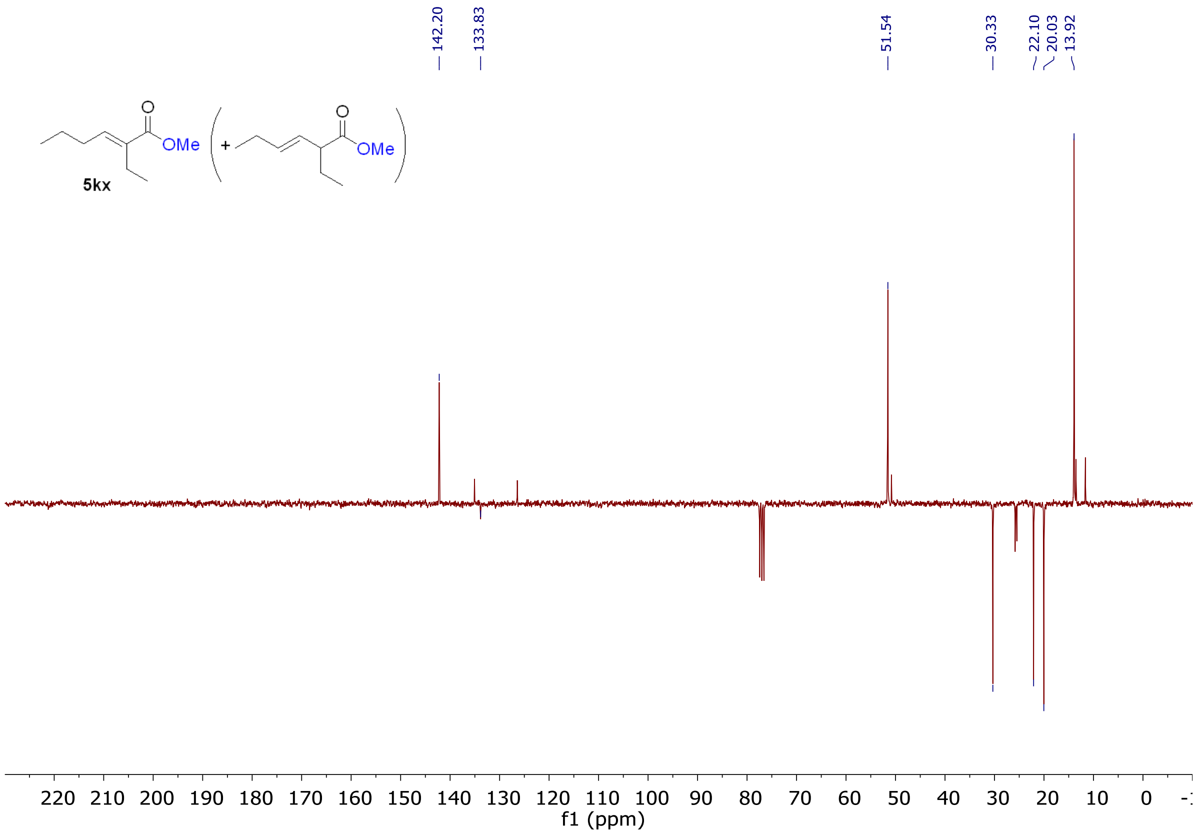

Figure S118. ^{13}C NMR (75 MHz, APT, $CDCl_3$) of **5kx**

7. References

[1] M. Carrera, M. de la Viuda, A. Guijarro, *Synlett* **2016**, *27*, 2783–2787.
[2] E. C. Izgu, A. C. Burns, T. R. Hoye, *Org. Lett.* **2011**, *13*, 703-705.
[3] a) A. Erkkilä, P. M. Pihko, *J. Org. Chem.* **2006,** *71*, 2538-2541; b) A. Erkkilä, P. M. Pihko, *Eur. J. Org. Chem.* **2007,** 4205-4216.
[4] W. Harnying, J.-M. Neudörfl, A. Berkessel, *Org. Lett*. **2020**, *22*, 386−390.
[5] J.-T. Ren, J.-X. Wang, H. Tian, J.-L. Xu, H. Hu, M. Aslam, M. Sun**,** *Org. Lett*. **2018**, *20*, 6636−6639.
[6] A. Biswas, J.-M. Neudörfl, N. E. Schlörer, A. Berkessel, *Angew. Chem. Int. Ed.* **2021**, *60*, 4507−4511; *Angew. Chem.* **2021**, *133*, 4557−4561.
[7] J. K. Mahoney,R. Jazzar, G. Royal, D. Martin, G. Bertrand, *Chem. Eur. J.* **2017**, *23*, 6206–6212.
